# Inferring the relation between transcriptional and posttranscriptional regulation from expression compendia

**DOI:** 10.1186/1471-2180-14-14

**Published:** 2014-01-27

**Authors:** Sandra Van Puyvelde, Jos Vanderleyden, Kathleen Marchal

**Affiliations:** 1 Center of Microbial and Plant Genetics, Kasteelpark Arenberg 20, B-3001 Leuven, Belgium; 2 Department of Plant Biotechnology and Bioinformatics, Ghent University, 9052 Gent, Belgium; 3 Department of Information Technology, Ghent University, IMinds, VIB, 9052 Gent, Belgium

**Keywords:** sRNA, Gene, Module network, Network inference, *Escherichia coli*

## Abstract

**Background:**

Publicly available expression compendia that measure both mRNAs and sRNAs provide a promising resource to simultaneously infer the transcriptional and the posttranscriptional network. To maximally exploit the information contained in such compendia, we propose an analysis flow that combines publicly available expression compendia and sequence-based predictions to infer novel sRNA-target interactions and to reconstruct the relation between the sRNA and the transcriptional network.

**Results:**

We relied on module inference to construct modules of coexpressed genes (sRNAs). TFs and sRNAs were assigned to these modules using the state-of-the-art inference techniques LeMoNe and Context Likelihood of Relatedness (CLR). Combining these expressions with sequence-based sRNA-target interactions allowed us to predict 30 novel sRNA-target interactions comprising 14 sRNAs. Our results highlight the role of the posttranscriptional network in finetuning the transcriptional regulation, e.g. by intra-operonic regulation.

**Conclusion:**

In this work we show how strategies that combine expression information with sequence-based predictions can help unveiling the intricate interaction between the transcriptional and the posttranscriptional network in prokaryotic model systems.

## Background

Transcriptional regulation plays a predominant role in prokaryotic organisms. Although the transcription factor (TF) - mediated network of *Escherichia coli (E. coli)* is amongst the best documented network, recent advances in next-generation sequencing unveiled the unprecedented role of sRNA-mediated posttranscriptional regulation by *trans*-acting sRNAs [1-3]. In *E. coli* more than 80 sRNA genes have been identified [4]. Most of the currently known sRNAs are *trans*-acting and assumed to be dependent on the chaperone Hfq. Hfq-dependent sRNAs have a short and imperfect binding region with their mRNA targets (10–25 bases) [5] and they execute their regulatory mechanism by direct base pairing with the mRNA of their target genes. The outcome of this interaction can be negative, for example by blocking ribosome entry (translational repression) or positive, where positive regulation can be mediated by a plethora of possible mechanisms, for example by melting inhibitory secondary structures, stabilizing the transcript or by sequestration of endonucleolytic cleavage [6,7]. Regulation is often coupled to nuclease mediated cleavage of the mRNA [8,9]. Another small number of posttranscriptional regulatory RNAs also act through antisense RNA base pairing with their targets, but in general in an Hfq independent way. This base pairing usually involves a longer stretch of complementary DNA. Targets of those sRNAs are positioned at their exact same location in the genome (cis location), but on the opposite DNA strand.

Although it is known that sRNAs are involved in a complex regulation network, it is still unclear how this sRNA-mediated posttranscriptional network relates to the TF-mediated regulatory network. Publicly available expression compendia that measure both mRNAs and sRNAs provide useful information to infer such interactions. Modi et al., 2011 [10] have already exploited the potential of network inference to compile an updated sRNA-regulatory network of *E. coli* using a well-known network inference approach CLR [11]. Compared to their work we relied on an alternative module inference framework (rather than a single gene based approach) to simultaneously assign TFs and sRNAs to target genes. In addition, we combined interactions inferred from expression compendia with sequence-based predictions to infer novel sRNA-target interactions. This integrative suite allowed us to both infer the sRNA-target network and to reconstruct the relation between the sRNA and the transcriptional network.

## Results

### Overview of the analysis flow

**Figure 1:**
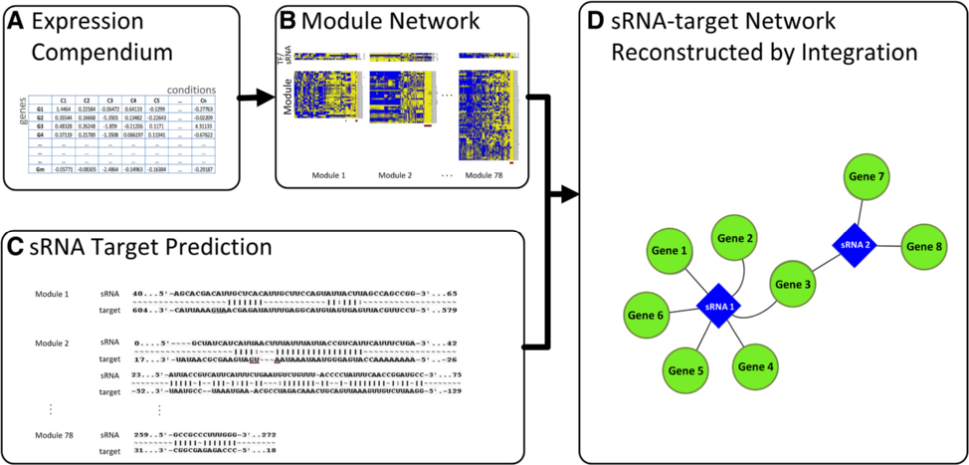
**Reconstructing the combined transcriptional-sRNA network.** An expression compendium compiled from publicly available microarray data is used as input (showed in Panel **A**). Using this compendium coexpression modules were constructed by means of biclustering. To each of those modules a regulatory program was assigned using either LeMoNe or CLR (Panel **B**). For genes in the modules we tested whether they contained a region in their sequence that shows complementarity to any of the sRNA sequences assigned to the module (Panel **C**). Integration of both the module network (modules with their regulatory programs) and the sequence-based predictions results in a final sRNA-target network (Panel **D**).

The analysis flow used to reconstruct the combined transcriptional-sRNA network from the expression compendium is depicted in Figure 1. We first inferred a module network from the expression compendium (Panel A and B). A module consists of a set of genes that is co-expressed, and the conditions under which these genes are co-expressed. Because genes in a module behave similarly, we assume they might be co-regulated either at the transcriptional or post-transcriptional level. Possible TFs or sRNAs that could explain their co-expression behavior were assigned to each of the obtained modules using expression-based network inference methods that assess whether there exists a similarity in the profile of the assigned TF/sRNA and that of the genes in the module to which the TF/sRNA is assigned. Because it has been shown that network inference approaches differing in their underlying principles often give complementary predictions [12], we used a combination of two different methods (LeMoNe [13] and CLR [11]) to make our final predictions.

Expression-based inference methods cannot distinguish whether the regulators affect the modules to which they are assigned in a direct versus an indirect way, i.e. whether the assigned regulators directly interact with the target genes in the modules to affect their regulation or whether they affect another regulator which on its turn physically interacts with the targets in the module. To infer for the assigned sRNAs direct from indirect modes of regulation, we complemented the expression-based inferences with sequence-based information (Panel C): direct interactions as summarized in the sRNA-target interaction network (Panel D) were inferred by identifying genes in the module that contained a region in their sequence that was complementary to a region present in an sRNA assigned to the module (results obtained from IntaRNA [14] and TargetRNA [15,16]).

### Module inference

To infer modules, we relied on a previously developed global biclustering algorithm (ISA [17,18]). With ISA, we identified 78 modules in our dataset of which 57 were functionally enriched. All 78 modules contained at least one predicted sRNA target (based on IntaRNA and TargetRNA predictions (see Methods)) and 21 modules contained an experimentally validated sRNA target. For several modules which showed a clear functional overrepresentation, sRNA targets within the module had a function related to the functional category assigned to the module (see below for a more detailed description of those modules). An overview of the modules is given in Additional file 1: Table S1: Characteristics of module network as reconstructed by CLR and LeMoNe. 37 out of the 108 experimentally verified sRNA targets ended up in a module, while the remaining sRNA targets remained unclustered. In some cases, e.g. for OmrA, OmrB, OxyS, DsrA, GcvB targets of the same sRNA, were clustered together. For the other cases it seems that targets, despite being regulated by the same sRNA exhibit a profoundly different expression pattern (Additional file 2: Table S2: overview of the sRNAs in different modules). This indicates an intricate interaction between the sRNAs and the TF-mediated transcriptional network.

### Assigning a regulatory program

To map the interaction between the transcriptional and the posttranscriptional network, we reconstructed a module network by assigning to each of the modules a regulatory program that consists of a combination of sRNAs and TFs. Because a module expression profile is more robust than a single gene profile, we assigned regulatory programs to modules rather than to single genes (here referred to as module networks). To this end, we used two previously described inference tools that exploit expression information to reconstruct networks, CLR and LeMoNe [12,19]. Both tools assume that the expression profile of the regulator is a proxy for its activity. They thus assign a regulator to a module if the expression profiles of both entities show a relation. The first method, LeMoNe [13] is inherently module-based: it assigns a regulatory program to pregrouped gene sets (or module). LeMoNe first partitions for the selected gene set in a module, the conditions according to different levels of over (under) expression (multivariate distribution). Then it assigns to each module those regulators for which the expression profiles best fits all or part of the condition partitions in the module. CLR [11], on the other hand assigns a regulatory program based on the degree of mutual information between the expression profile of a regulator and that of each possible target gene. Although initially developed as a direct inference method that assigns a regulatory program on a gene by gene basis, we apply CLR here to assign a regulatory program at module level (see Methods). As input for both CLR and LeMoNe we used the gene selection of the 78 ISA modules, but instead of only using the conditions selected in the module, we used all conditions in the compendium to perform the regulatory assignments as both CLR and LeMoNe can weigh the conditions ‘according’ to their relevance for the assignment of the regulatory program. An overview of the assignment of a regulatory program to the different modules is shown in Additional file 1: Table S1.

### Complementarity between CLR and LeMoNe

Both CLR and LeMoNe assign a score to the TF/sRNA module assignments. To set the threshold for CLR, we used the FDR-based strategy described in the original paper [11]. For LeMoNe we relied on a previously optimized threshold [19]. These criteria resulted in assigning on average 2–3 regulators (with a regulator being defined as a TF or sRNA) per module for CLR (159 assignments comprising 75 regulators) and 1–2 for LeMoNe, (165 assignments comprising 89 regulators). To four modules no regulators were assigned, given the defined thresholds.

**Figure 2:**
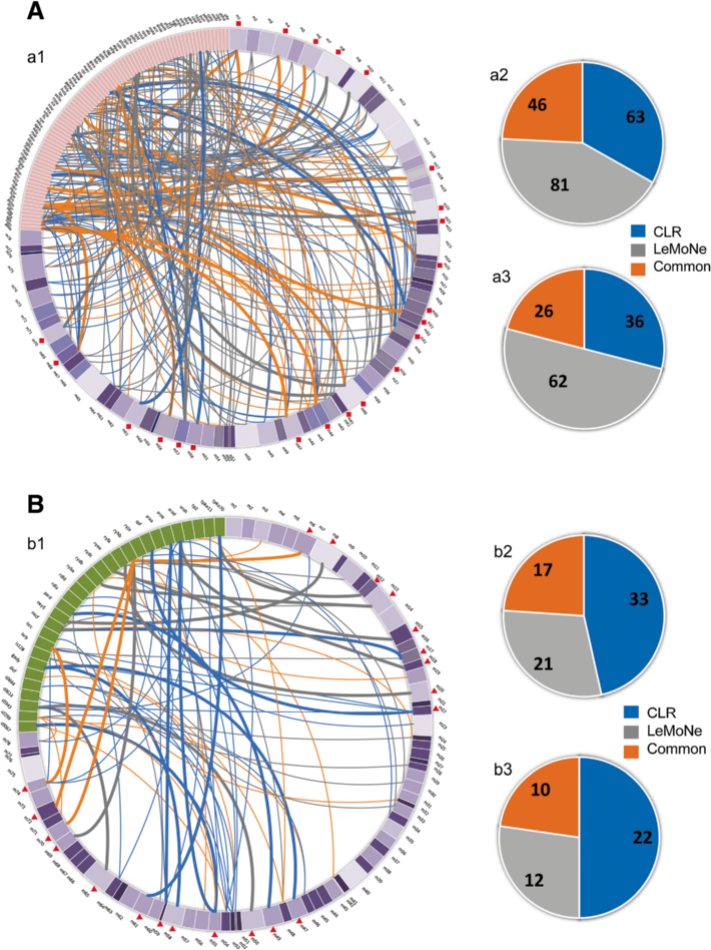
**TFs/sRNA to module assignments by LeMoNe and CLR.** Panel **A**: Assignments for TFs (according to the results in Additional file 1: Table S1). **a1**: relation between TF assignments and modules. Pink: TFs. Purple: modules, with a higher degree of shading corresponding to a smaller sized module. Red square: enrichment in targets of the assigned TF. Blue lines: assignments uniquely made by CLR. Gray lines: assignments uniquely made by LeMoNe. Orange lines: assignments made by both methods. Bold face lines: assignment confirmed by target enrichment analysis. **a2**: complementarity in the assignments made by CLR and LeMoNe from the assignment point of view. Gray: assignments uniquely made by LeMoNe. Blue: assignments uniquely made by CLR. Orange: assignments made by both methods. **a3**: complementarity in the assignments made by CLR and LeMoNe from the TF point of view. Gray: number of TFs uniquely assigned by LeMoNe. Blue: number of TFs uniquely assigned by CLR. Orange: number of TFs assigned by both methods. Panel **B**: Assignments for sRNAs (according to the results in Additional file 1: Table S1). **b1**: relation between sRNA assignments and modules. Legend as in Panel A a1 except for Green: sRNAs. Bold face line: the module to which the sRNA was assigned also contains a predicted or an experimentally verified target of the assigned sRNA. **b2**: complementarity in the assignments made by CLR and LeMoNe from the assignment point of view. Legend as in Panel A a2. **b3**: complementarity in the assignments made by CLR and LeMoNe from the sRNA point of view. Legend as in Panel A a3.

Figure 2 gives an overview of the TF and sRNA assignments by either LeMoNe or CLR to the different modules (Panel A). Figure 2 panel B and C summarizes quantitatively the complementarity between LeMoNe and CLR for respectively the TFs and sRNAs. Because in principle the same regulator (TF/sRNA) can be assigned to different modules, the number of assignments is larger than the number of assigned regulators. The complementarity between both methods is therefore displayed both from the perspective of the assignments and of the assigned number of TFs.

For TFs a total of 190 assignments, covering 84 TFs were made of which 127 assignments covering 71 TFs were made by LeMoNe and 109 assignments covering 49 TFs were made by CLR. Of the total number of assignments, 46 assignments covering 26 different TFs were consistent between CLR and LeMoNe. For about 39 assignments made by either CLR or LeMoNe covering 17 different TFs, the assignments were confirmed by target enrichment analysis: that is the module to which the TF was assigned indeed was overrepresented in known targets (according to RegulonDB) of the assigned TF (Figure 2 Panel A). 66.7% of these assignments that were confirmed by target enrichment analysis can be found in the intersection of targets predicted by both methods.

For the sRNAs, a total of 71 assignments for 30 different sRNAs were made. Of the 38 assignments for 18 sRNAs made by LeMoNe and the 50 assignments for 26 sRNAs made by CLR, 17 assignments for 10 sRNAs were consistently predicted by both algorithms. For 3 cases (one assignment for MicF (predicted by CLR), one assignment for Spf (predicted by CLR), and one assignment for RyhB (predicted by LeMoNe and CLR)), the module to which the sRNA was assigned also contained at least one experimentally verified target of this assigned sRNA. 21 out of the 43 modules to which an sRNA was assigned, contained either a predicted or an experimentally verified target for the assigned sRNA, indicating that probably in the other cases the assigned sRNAs are involved in the indirect rather than the direct regulation of the genes in the module e.g. by regulating the TFs that were assigned to the module rather than by regulating the genes in the module itself.

Regarding the complementarity between LeMoNe and CLR, results show that both methods tend to make more predictions for TFs than for sRNAs which indicates that the signal in the dataset is more pronounced for TFs than for sRNAs. This is to be expected as the probes in the used array platforms were designed to measure protein coding genes (and thus TFs), but not sRNAs. sRNAs are in general represented by very few probes and not necessarily on all platforms (see Methods). Other differences between the results obtained by either method can be explained by the method’s specificities. In general, CLR performs well if the profile of the assigned regulator matches the profile of the module for a minimal number of conditions of any type (it tests a global similarity rather than a condition specific profile). LeMoNe on the other hand assigns more importance in finding a fit between the expression profile of the regulator and that of the module for those condition partitions that are homogenous. The latter can contain a large part of the conditions in the dataset (in which case the interaction would also be recovered by CLR) or it can be restricted to only a subset of the conditions (in which case the interaction would only be recovered by LeMoNe). However, LeMoNe’s ability to assign regulators that only match part of the conditions in the dataset comes at the expense of also penalizing more small mismatches between the profiles of the regulators and those of the modules for the condition partitions of importance. So even if the global profile seems to match quite well (high CLR score), such mismatches can result in low LeMoNe scores. Both properties can explain why for the same thresholds used on both TFs and sRNAs, LeMoNe tends to find more unique assignments for TFs than for sRNAs compared to CLR (for which the opposite is true): because for TFs the signal is rather robustly measured, LeMoNe can also assign regulators for which the expression profile only matches the module’s profile for a subset of the conditions, whereas CLR can not assign those regulators, explaining why LeMoNe assigns relatively more TFs than CLR. However, in the case of sRNAs, the expression signal is much less robustly measured and small mismatches in the expression profile of the regulator and that of the module become a major limitation for LeMoNe. This results in CLR being able to assign more sRNAs than LeMoNe under those conditions.

The most reliable assignments of regulators (TF or sRNA) to modules evidently consist of the predictions that were made by both LeMoNe and CLR (indicated in bold in the Additional file 1: Table S1). A selection of interesting modules is described in more detail below.

### sRNA-target interaction network

**Figure 3:**
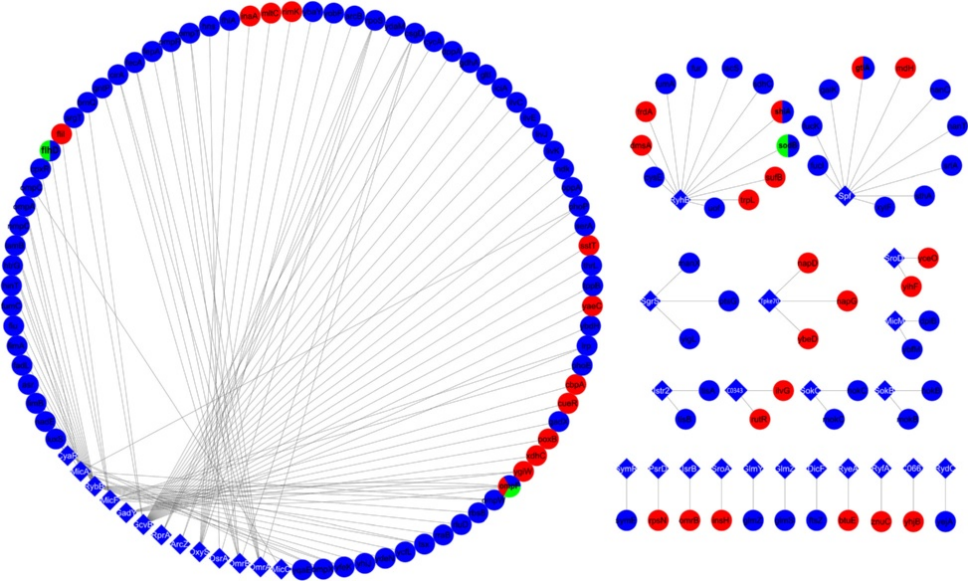
**sRNA-target interaction network as inferred by the network inference procedure.** sRNAs are indicated as diamonds and the targets of the associated sRNAs are indicated in circles. In the picture we show both targets predicted by our analysis (in red, circle or sector) and those present in our current benchmark set (in blue sector or in a circle) to illustrate the extent to which our targets overlap with the benchmark. Targets of the benchmark predicted by Modi et al. [10] are indicated in green (circle or sector). For both Modi et al. [10] and our approach we used those predictions obtained by combining the network-based assignments with the sequence-based predictions.

To derive from the sRNA-module assignments the underlying sRNA-target network, we defined as potential targets of a certain sRNA those module genes that also contained in their upstream regions a putative recognition site of the sRNA assigned to the module (based on the union of IntaRNA and TargetRNA). This resulted in 30 different sRNA-target interactions (corresponding to 33 sRNA-target assignments) comprising 14 sRNAs (out of the 72 for which we tried to make an assignment) regulating 30 genes (Figure 3).

Recently, Modi et al. [10] have also applied CLR to predict novel sRNA-target interactions for 24 sRNAs (which are all contained in the set we used in our analyses). Our approach is intrinsically quite different from the one of Modi et al. [10] in its experimental set up: whereas Modi et al. [10] assigns sRNAs to single genes, we assign sRNAs to modules (module-base inference). In addition both approaches, ours and the one of Modi et al. [10] use slightly different parameter settings for running CLR and the sequence-based sRNA-target predictions. We compared to what extent the results of our approach corresponded to those of Modi et al. [10] (Additional file 3: Table S3).

Whereas we integrate sequence-based predictions with the module-based sRNA assignments to infer the direct sRNA-target interaction network (Figure 3 and Additional file 3: Table S3 column f), Modi et al. [10] in their study used expression profile-based sRNA assignments to construct an sRNA-target interaction network which is thus composed of both direct and indirect targets. The original number of predictions made by Modi et al. [10] based on expression data only is shown column c of the table in the Additional file 3: Table S3. To make the results of both networks more comparable, we removed from the predictions of Modi et al. [10] the indirect interactions using the sequence-based prediction approach adopted in their original paper (column d).

Results on a benchmark dataset show that both methods ours and the one of Modi et al. [10] perform poorly in recovering true benchmark interactions and have a very low sensitivity. When considering the targets of Modi et al. [10], predicted based on high scoring CLR assignments, benchmark interactions could be recovered for 5 sRNAs (Additional file 3: Table S3 column e) (MicF (1 target)), GadY (1 target), GcvB (2 target), RyhB (1 targets)). Of those, three benchmark interactions (MicF, RyhB, GadY) were retained if the target was also required to contain a recognition site for the respective sRNA (column e between brackets). We had a similarly low sensitivity (column h between brackets) and could also only recover the known MicF target from the benchmark. Comparing columns d and g of Additional file 3: Table S3, containing respectively the number of direct sRNA targets predicted by Modi et al. [10] and our study by combining expression and sequence data shows that both methods predict novel targets for a very different set of sRNAs. Besides for MicF and RyhB for which both our method and Modi et al. [10] can predict targets, Modi et al. [10] predicts targets for GcvB whereas we do not. In contrast, with our approach we were able to predict targets for OxyS, GadY, RyeA, PsrD, SroD, Tpke70, SroA, IsrB and C0343 that were not detected by Modi et al. [10] (the last 7 sRNAs were not analyzed by Modi et al. [10]). This indicates the complementarity between single gene and module-based approaches [12].

### Description of interesting modules

An overview of the modules can be found in Additional file 1: Table S1. The more detailed content of the modules, together with their regulatory program can be found at Additional file 4: Module Overview.

#### Module 6

**Figure 4:**
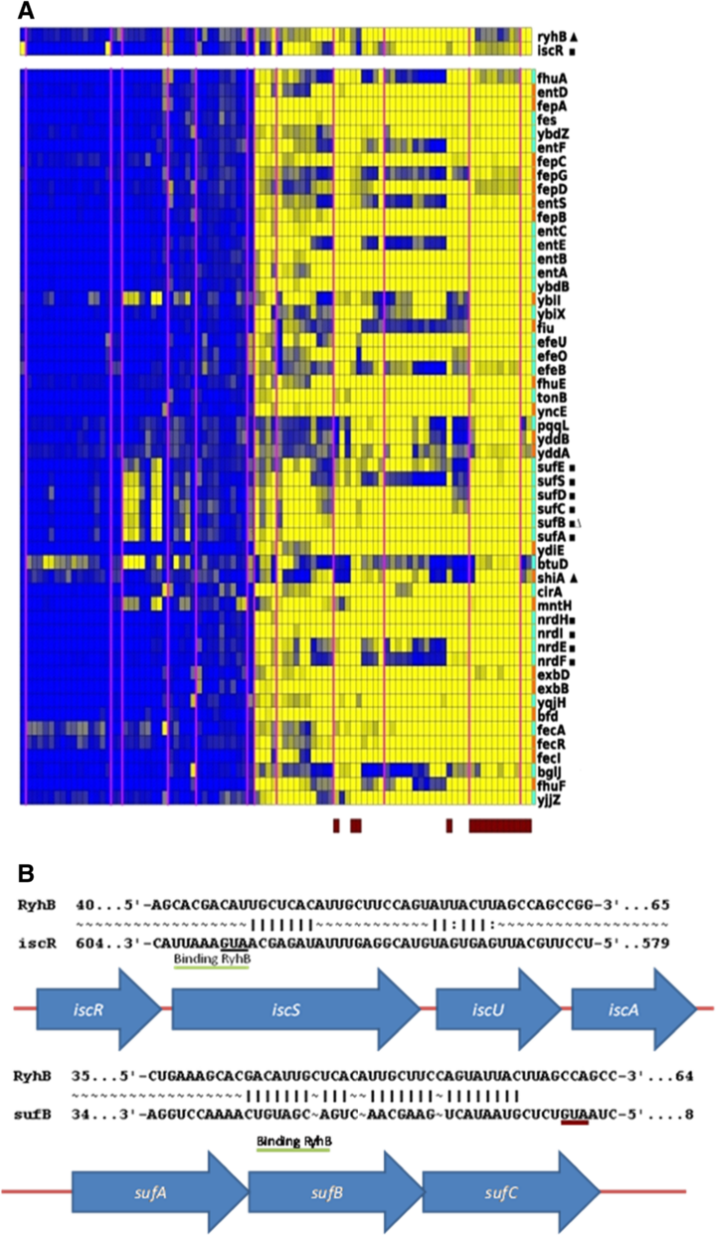
**Module 6 and its regulatory program.** Panel **A**: the regulatory program assigned to this module. The module to which the regulatory program was assigned; yellow indicates high expression levels and blue refers to low expression levels of genes in the module. The genes correspond to the genes present in the original module discovered by ISA. Conditions present in the original ISA module are indicated by a horizontal bar. As both LeMoNe and CLR use all conditions when assigning their respective regulatory program, we indicated also the additional compendium conditions that were relevant for assigning the respective regulatory programs. Genes in the module correspond to likely targets of the assigned regulators. Targets indicated by a square correspond to known targets of the assigned TF (s). Targets indicated by empty triangle correspond to predicted targets of the assigned sRNA, targets indicated by filled triangle correspond to known targets of the assigned sRNA. Panel **B**: sRNA-target interaction as predicted by the sequence-based analysis for both known and predicted targets of the sRNAs assigned to the module. Indicated sequence positions refer to the location of the recognition sequence relative to the translation start of the gene the sRNA is predicted to interact with (if ATG is indicated in red). If the ATG is indicated in black it refers to the start codon of a neighboring gene. In this case the underlined ATG corresponds to the start codon of IscS as the target site of RyhB is located in the intergenic region between the predicted target IscR and IscS.

Module 6 (Figure 4 panel A) is a rather large module being overrepresented for genes involved in iron transport and iron-sulfur cluster assembly. Two regulators, one TF (IscR) and one sRNA (RyhB) were assigned to this module with a high reliability (as their assignment was confirmed by both LeMoNe and CLR): IscR, a sulfur-cluster containing TF, known to regulate the expression of operons that encode components of a pathway of iron-sulfur cluster assembly, iron-sulfur proteins, anaerobic respiration enzymes and biofilm formation [20,21]. Module 6 contains two known targets being regulated by IscR (the operons *nrdHIEF* and *sufABCDES*, involved in iron-sulfur cluster assembly [22]). Interestingly, the regulator IscR belongs to a polycistronic mRNA *iscRSUA* known to be regulated by the sRNA RyhB which was also assigned to this module.

This assignment of RyhB was further confirmed by that fact that the module contained one known target of RyhB (*shiA*) [23]. In addition to this known target we also have one predicted RyhB target in the module, *sufB* (Figure 4 panel B), which is a component of the SufBC_2_D Fe-S cluster assembly scaffold complex [24] that is responsible for the synthesis of Fe-S clusters [24-26].

Down-regulation of proteins involved in assembly of Fe-S clusters by RyhB would make sense given the known function of RyhB during Fe homeostasis: RyhB is known to reduce iron consumption under low-iron conditions by downregulating expression of iron-containing proteins [27-29] and the Fur regulon, of which also several genes are present in the module and which is known to have a central role in iron metabolism. In addition the direct binding of RyhB to *sufB* seems likely as the interacting region identified in RyhB is located in an unstructured region and overlaps with the binding region of previously detected targets (see Additional file 1: Table S1).

Interestingly, both the ISC assembly system (*iscRSUA* operon*)*, which is responsible for Fe-S cluster production under normal conditions and the SUF assembly system (*sufABCDES* operon) responsible for Fe-S cluster production under oxidative stress conditions are encoded by polycistronic operons [30]. The polycistronic *iscRSUA* mRNA to which also IscR, the regulator assigned to the module belongs, is known to be processed by RyhB by inducing a cleavage of the operonic transcript between *iscR* and *iscSUA*[31]. Our predicted interaction between RyhB and *sufB*, the second gene of the *sufABCDES* operon suggests that RyhB would also interact with the *sufABCDES* operon through an intraoperonic regulation mechanism [31].

#### Module 17

Module 17 contains 31 genes most of which relate to membrane encoded transport systems. The module was predicted to be regulated by the TFs MalT, of which also the targets were found to be enriched in this module, CueR and YgiV. For the latter TFs no known targets were found in the module. For the sRNA MicF assigned to this module, one of its known targets, *ompF*, belongs to the module [9]. Although the module does not contain any other predicted targets of MicF, we found evidence of a MicF recognition site upstream the coding region of the *ygiV*, a TF also assigned to the module. The recognition size is located in the region from -102 to -52 bp upstream of *ygiV*, a region that comprises the short intergenic region between *ygiW* and *ygiV*, and the C terminal end of the coding region of *ygiW*. The region on MicF, predicted to bind with *ygiV,* although being quite large partially overlaps with the unstructured 5′end of MicF to which also the known MicF target OmpF binds (see Additional file 4: Modules overview). YgiV is known as a repressor of McbR (also known as YncC), a regulator of biofilm formation [32].

Also interesting is the assignment of the sRNA IsrB with unknown function to the same module. IsrB in *E. coli* has no documented targets yet, but its genomic location overlaps with the coding regions of *azu*-genes, a set of inner membrane encoding genes with unknown function. A link between IsrB and membrane encoded functions is plausible viewing the large subset of membrane related functionalities in this module. However, relying on our sequence-based sRNA target prediction, no direct target of IsrB was found to be present in this module so IsrB could be involved in the indirect regulation of this module (e.g. by regulating other regulators that on their turn regulate the genes in the module).

#### Module 58

Module 58, mainly expressed under stationary growth contains genes involved in acid response, amino acid starvation (purine salvage, amino acid uptake) and induction of microaerobiosis (represented by Ecocyc enrichment analysis). Three regulators were assigned to Module 58, GadE, CueR and the sRNA GadY, all of which are known targets of RpoS [33]. Module 58 was also found to be enriched in direct targets of GadE (see Additional file 1: Table S1), indicating that the assignment of GadE as a regulator to module 58 is true. GadE, the central activator of the acid response system controls genes involved in the maintenance of pH homeostasis through its direct targets involved in the glutamate-dependent acid resistance system (here represented by *gadA* and *gadBC* genes) and is involved in multidrug efflux (*mdtE*, *mdtF*) through controlling the expression of the TFs, GadW and GadX both related to acid resistance (of which only GadW was found in the module) [34]).

The sRNA GadY which is highly expressed during entry into stationary phase and regulated by low pH [35] is related to the GadE dependent acid response through an intricate network of interactions with GadW (also in module 58) and GadX (according to Regulon DB).

A last regulator assigned to module 58 was CueR “Cu efflux regulator”, which was also predicted to be a target of GadY using our sequence-based predictions. CueR, regulates genes related to the primary copper homeostasis system in response to the presence of copper, silver, or gold ions [36]. None of the known CueR targets related to its function in Cu^2+^ homeostasis were found in module 58. However, CueR being a target of GadY and also being assigned as a regulator to module 58 points towards a connection between Cu^2+^ and pH homeostasis, a link that has been suggested before. Yamamoto et al., for instance, showed that pH changes affect the genome-wide transcription pattern of copper-balance genes in the presence of CuSO_4_[37].

Besides CueR, module 58 contained three additional predicted targets of GadY (assuming that genes belonging to a module with an assigned sRNA as regulator that also contain a recognition sequence of that sRNA in their upstream region are direct targets of the sRNA). A first one, CbpA has a functionality related to the one of DnaJ and functions as a co-chaperone with DnaK. A second one, PoxB is pyruvate oxidase and the last one XdhA-XdhB-XdhC is a putative heterotrimericxanthine dehydrogenase [38]. How their functionalities link to the role of GadY is less clear. GadY is known to be an antisense binding sRNA that acts on its cis encoded target GadX. So far GadX is the only characterized target of GadY. However, it cannot yet be excluded that GadY would have additional targets encoded elsewhere on the genome [39], the more because GadY has been shown to share the Hfq binding property of transacting sRNAs [33]. The fact that the regions to which GadY would bind in its predicted targets CueR, DnaJ and PoxB are located quite far upstream of their respective annotated TSSs could explain why such non-conventional targets have largely been overlooked by computational predictions.

#### Module 8

Module 8 contains pathways which relate to oxidative membrane stress (osmotic stress response, efflux pumps, membrane remodeling). Three regulators have been assigned to this module by LeMoNe: MarA, Fur and OxyS. MarA, is a “multiple antibiotic resistance” regulator of which indeed part of its known regulon was found in the module. MarA is an outer membrane porin involved in the efflux of several hydrophobic and amphipathic molecules and is known to be involved in resistance to antibiotics and oxidative stress [40]. The module indeed contains MarA targets such as TolC, an outer membrane porin. Although the Fur regulon members are not well represented in this module, the autoregulated TF Fur has not only been assigned to the module, but also belongs to the module itself, further supporting its assignment. Besides its well documented role in iron homeostasis, Fur is also known to be involved in oxidative stress responses by downregulating iron uptake systems [41].

Next to these TFs also the sRNA OxyS known to play a regulatory role in the oxidative stress response [42] was assigned to this module. Three targets regulated by OxyS were predicted with our approach and were found in module 8, implying that OxyS regulates together with MarA the genes in module 8: RimK, a ribosomal protein S6 modification protein belonging to the *ybjC-nfsA-rimK-ybjN* operon, an operon which indeed is known to be regulated by (Rob/MarA/SoxS) and OxyR. So, the additional regulation of *rimK* (intraoperonic promotor site) by OxyS is plausible. InaA, a second predicted target of OxyS present in module 8 is pH-inducible protein involved in stress response [43,44]. A third target of OxyS which we could predict is *mltC*, a membrane-bound lytic murein transglycosylase C, known to be induced by oxidative stress via SoxS [45].

#### Module 20

Module 20 contains genes belonging to pathways involved in transport, oxidative stress response (Mar and Sox operons) and gluconate, ascorbate utilization. SoxR which was assigned to the module is also part of the module. The regulator IclR also assigned to the module is known to regulate the glyoxylate bypass operon [46,47].

According to our predictions, the sRNA assigned to this module RyfA, which has no assigned function yet would have one predicted target in the module, i.e. ZnuC, the ATP-binding component of an ABC transporter involved in high-affinity zinc uptake (ZnuABC). *znuC* transcripts were shown to disappear or markedly decreased at 5 min after zinc addition [37]. Such quick induction or repression of the zinc-responsive genes upon increasing environmental zinc levels suggests a regulation mechanism mediated by sRNAs. Some of the enzymes being expressed in the module are indeed known to depend upon a Zn^2+^ containing active site (e.g. UlaE [48]). In literature we found an indirect relation between SoxS and RyfA through the regulation of the predicted target ZnuC. SoxS is known to increase the expression of the zinc uptake system ZnuACB in *E. coli,* although no direct binding of SoxS to the promoter of *znuACB* has been observed [49].

#### Module 61

Module 61 (44 genes) contains genes related to oxidation-reduction, electron transport and energy generation. Three TFs, AdiY and YahA were assigned to this module by LeMoNe and one CdaR by CLR. To our knowledge the role of YahA, a c-di-GMP-specific phosphodiesterase is yet unknown (YahA contains an EAL domain close to an N-terminal putative DNA-binding domain). AdiY was previously shown to be strongly upregulated after a rapid decrease in external pH [50]. Its known target, the arginine decarboxylase system (*adi*) is known to be induced in rich medium, under anaerobic conditions, and at low pH [50,51] conditions under which genes present in the module are also known to be expressed. CdaR, regulates genes involved in the uptake and metabolism of galactarate and glucarate and is also found to be one of the regulators for which the targets are enriched in the module. Besides these TFs also Tpke70 a sRNA of approximately 40 nt in length with yet unknown function was assigned to this module [52]. Module 61 also contains two predicted targets of Tpke70 that is NapG and NapD (predicted using sequence-based methods) both parts of the periplasmic nitrate reductase system in *E. coli*[53-55].

## Methods

### Microarray compendium

The *E. coli* compendium used in this study consisted of 348 contrasts taken from an initial compendium of 610 contrasts, covering 4311 genes and 78 sRNAs and profiling a diverse set of conditions such as (an)aerobic growth, growth in ethanol, different pH levels, various *E. coli* strains included, etc. In an initial test we used the full compendium to generate our results (610 contrasts). We noted that a subset of 262 contrasts (row 19 in Additional file 5: Table S4), corresponding to the previous compendium of [11] did not exhibit a large variability across conditions and did therefore bias our results. That is why we excluded this dataset for further analysis (resulting in a total of 348 remaining contrasts). The final compendium was composed of 31 experiments performed on 21 Affymetrix platforms AffymetrixGene Chip *E. coli* Antisense Genome Array [Ecoli_ASv2], 12 AffymetrixGeneChip *E. coli* Genome 2.0 Array [E_coli_2] (Additional file 5: Table S4: Description of expression compendium). The platform design files did not yet contain probe annotation for sRNAs, but some of the ‘intergenic’ probes corresponded to regions containing sRNAs. By blasting the probe sequences against a more recent annotation of the *E. coli* genome, we could link 1331 (1323 probes for sRNAs from Additional file 6: Table S5: Probe Information) probes to 72 sRNAs that were covered by all 3 Affymetrix platforms (a detailed list with probe annotation can be found in the supplementary information Additional file 6: Table S5).

### sRNA target prediction

sRNA target prediction was based on IntaRNA [14] and TargetRNA [15]. For both IntaRNA and TargetRNA, we searched for sRNA interaction sites in a region close to the start codon (ranging from 50 bp upstream to the ATG until -150 downstream from the ATG as this range showed to have the best sensitivity in recovering known sRNA-target interactions). For both algorithms the seed length was set to 8 nucleotides. Other settings had default values TargetRNA was performed without scores accounting for GU pairs, without thermodynamic information and without orthology information).

Target predictions were considered selected stringently if they were in the top 25 list of both methods (stringent selection criterium). If they were only predicted in the top 25 list of one of the two methods, they were considered less reliable (and we referred to this as the non-stringent selection criterium). Using the stringent selection criteria approximately 12% of the known targets for sRNAs could be retrieved with an average PPV of 19%. With the non-stringent criteria these numbers are respectively 18% with a PPV of 1%. However, as we could recover so few benchmark interactions with the stringent criterium, we relied on the non-stringent criterium, assuming that we would compensate for the lower PPV by integrating the sequence-based predictions with those obtained from the expression-based assignments (Additional file 7: Performance tests to optimize sequence-based sRNA-target predictions). For a full list of predictions we refer to Additional file 8: Table S6: Predictions of sRNA targets based on intaRNA and TargetRNA.

To identify putative interactions between TFs and sRNAs assigned to the same modules, we screened the sequences of the assigned regulators for recognition sites of the sRNAs assigned to the same module in a region near the start codon. Here we used a more relaxed screening to identify putative interactions that is we extended the screened region from -150 bp upstream to the ATG to 200 downstream from the ATG and screened an additional region near the transcript end (200 bp upstream of the stopcodon to the stopcodon).

To further validate the sequence-based predictions of the most promising sRNA-target interactions (those described in the main text), 1) we tested to what extent the location of the recognition region of sRNA-target interactions in the respective sRNAs overlapped with an unstructured regions [56] and, with the binding region of previously described targets of the same sRNAs, 2) we tested to what extent the location of the recognition region of the respective sRNA-target interactions in the target sequences were positioned relative to the start codon and transcription start positions of those targets. Results of this analysis are displayed Additional file 4: Module overview.

### Module detection

Modules (biclusters) were generated by running ISA [17,18] obtained from ISA website ( http://www2.unil.ch/cbg/index.php?title=ISA) on the *E. coli* expression compendium mentioned above, using default thresholds on the minimum number of genes (3) and on the minimum number of conditions (2). The ctrh parameter for checking convergence was set to be 50. The default number of 100 random seeds needed to initiate the global biclustering were generated using generate.seeds(). Running ISA on the *E. coli* compendium resulted in 78 modules.

### Inferring module networks

To assign a regulatory program to the 78 modules, we used two previously described inference methods CLR [11] and LeMoNe [12,19]. As input for both CLR and LeMoNe we used the gene selection of the 78 ISA modules, but instead of only using the conditions selected in the module, we used all conditions in the compendium to perform the regulatory assignments as both CLR and LeMoNe. Both tools require as input a regulator list to reconstruct the regulatory program. To this end we compiled a list of 311 potential regulators (transcription factors and sRNA regulators) from RegulonDB release 8.1 (RegulonDB version 7.0) [57], Ecocyc [58], RFAM [59], and literature (see Additional file 9: Table S7: List of Regulators). These regulators consisted of 170 annotated TFs (RegulonDB), 69 predicted transcription factors predicted (Ecocyc), 72 annotated sRNAs (RFAM), covering about 50% of all TFs in *E. coli*[60] and 30% of all estimated sRNAs (based on the analysis of all the non-coding transcripts in *E. coli*[61] ). LeMoNe was obtained from source website ( http://bioinformatics.psb.ugent.be/software/details/lemone). It is an unsupervised, module-based method that assigns regulators (TFs or sRNAs) to a an expression module (here our ISA modules) by first fitting a multivariate normal distribution to the expression profiles of all genes in the module such that the module conditions are regrouped in partitions with a coherent expression value (being either over or underexpressed according to a multivariate normal distribution that fits all genes in the module). It subsequently searches for the set of regulators for which the expression profiles best fit to part or all of the condition partitions in the module. LeMoNe was run with the default parameter settings. Regulators assigned by LeMoNe were ranked according to their regulator score (we only withheld regulators which had a score higher than 100). In our previous work we showed that this threshold value was shown to be well suited for assigning TFs as regulators to modules in *E. coli*[19]. CLR was obtained from the developer [11]. CLR was run with default parameter settings except for the statistical mode settings (we selected NORMAL mode which is recommended on larger networks). CLR was initially developed as a direct inference method [12], that assigns a regulator to an individual gene if the expression profiles of the gene and its assigned regulator show a sufficiently high mutual information. CLR was initially not developed to explicitly exploit modularity. To be able to use CLR also at module level, we calculated for each module its mean expression profile (over all conditions of the compendium). We assigned regulators to both individual gene expression profiles and module mean profiles. We observed that CLR was able to more confidently assign regulators to the mean module profiles than to the individual gene expression profiles (op scoring assignments made by CLR almost always involved mean module profiles when we used as input to CLR a mix of mean profiles and the profiles of the individual genes). This indicates that using modules instead of individual profiles helps reducing the noise and makes the assignments more robust. As a final threshold for CLR we selected a cut-off that corresponds to an FDR < 0.05 using the FDR (false discovery rate) calculation described in the original paper.

### Calculating functional enrichment

Functional enrichment was based on GO (category Biological process) [62] using a hypergeometric test and using false discovery rate (FDR, based correction for multiple testing (Benjamini and Hochberg correction), as implemented in BinGO [63]. Categories overrepresented with a significance level <0.05 were withheld. Testing whether coexpression modules were enriched for known targets of TFs (according to RegulonDB) was also based the hypergeometric test with FDR correction (p <0.05 were considered significant). Because of the low number of known sRNA targets, the enrichment value of expression modules in sRNA targets was unreliable and therefore replaced by indicating for each expression module the number of known or predicted targets it contained for the assigned sRNA.

Ecocyc pathways functional enrichment was based on pathways description [58] using a hypergeometric test and using false discovery rate (FDR, i.e. the expected proportion of false positives among the positively identified tests), based correction for multiple testing (Benjamini and Hochberg correction, build-in Genecodis tool [64]). Categories overrepresented with a significance level <0.001 were withheld.

### Benchmarks

We compiled a benchmark of 118 experimentally verified sRNA-target interactions in *E. coli* covering 25 different sRNAs (both Hfq-dependent and non Hfq-dependent) from curated databases (RegulonDB [55], Ecocyc [58]) and literature. A full table of curated interactions can be found on the supplementary table (Additional file 10: Table S8: Description of our benchmark of known sRNA-target interactions). This benchmark is an updated version of the one described in Modi et al. [10] with more recently validated interactions [65-68].

Compared to Modi et al. [10] our benchmark contained 71 additional interactions for 25 sRNAs (containing known interactions for 5 sRNAs that were not covered by Modi et al. [10], and 65 additional interactions for 20 sRNAs that were covered in the initial benchmark of Modi et al. [10]).

### Comparison with the predicted sRNA network of Modi et al. 2011

The CLR based predictions made by Modi et al. [10] were derived from its supplementary file (st02.doc). Sequence-based predictions on sRNA-target interactions were not available as such from the original paper. To generate these predictions in a way that was described in the original paper we did the following: we used the TargetRNA [69] with the same default settings [15] mentioned also above, but including a default setting for the seed length (9 nucleotides), sRNA sequences were used from the source TargetRNA server [69], or, if not available on this server from RFAM or Ecocyc. We searched for sRNA recognition sites in a region close to the start codon, ranging from -30 bp upstream of the ATG to 20 bp downstream of the ATG. Target predictions were considered selected stringently if they had a p-value less than 0.01. Predictions on sRNA-target interactions prior to and after the sequence-based filtering were compared for both our analysis and the analysis of Modi et al. [10] on our updated benchmark dataset (Additional file 3: Table S3).

## Discussion

This work studied the relation between the transcriptional and the posttranscriptional network by applying two complementary module-based inference frameworks LeMoNe and CLR to simultaneously assign TFs and sRNAs to coexpression modules. In general, we observed that in contrast to targets of the same TF, targets of the same sRNA rarely show a similar coexpression behavior, indicating that the level of posttranscriptional regulation has been evolved towards an additional layer of regulation that is largely involved in finetuning gene-specific expression behavior. This is further confirmed by the observation that for several of our novel detected sRNA-target interactions, the recognition region of the interaction is located within an operon (e.g. MicF–*ygiV*, RyhB–*sufB*, GadY-*xdhC*), pointing towards an extended role of these sRNAs in intraoperonic regulation [70]. We also observed that in contrast to TFs, sRNAs assigned to a module were most frequently only involved indirectly in the regulation of the module genes that is by modulating the expression of the TF responsible for the coexpression behavior of the genes in the module.

Combining sequence-based target predictions with the module-based sRNA assignments allowed us to draw a predicted sRNA-target interaction network. A similar sRNA-target reconstruction was recently performed by Modi et al. [10] using CLR in a non-module based setting. Our approach is intrinsically different from the single gene based approach applied by Modi et al. [10]. In a module-based approach one assumes that different genes in the same module all support the inferred interactions, which should contribute to the reliability of the assignments, but might come at the expense of missing interactions with genes that will not end up in a module which might be recovered by a single gene based approach [12]. Secondly, rather than presenting the interactions inferred through the expression data as a final sRNA-target interaction network as was done in Modi et al. [10] we combined the expression-based inference with a sequence-based inference. Because of these differences in the number of interactions, it is not trivial to compare the results of our analysis and the one of Modi et al. [10] directly. Applying also a sequence-based filtering to distinguish direct from indirect targets on the results of Modi et al. [10] indicated that for both methods the recovery of known interactions is still very limited. At this stage this does not say much about the quality of the novel predicted interactions, but rather indicates the limitation of the currently available expression compendia that are mainly compiled from expression arrays that were not yet designed to measure sRNAs.

## Conclusion

In this work we show the potential of combining network-based inference with sequence-based prediction techniques for identifying sRNA-target interactions and for studying the intricate relation between the transcriptional and the posttranscriptional network. Such integrative data-analysis techniques can be expected to become increasingly attractive as more specialized compendia measuring simultaneously protein coding genes and sRNAs will become available.

## Authors’ contribution

II performed the analyses and helped designing the experiments and drafting the text. YW performed the module detection, contributed to the benchmark analysis and the draft. SVP contributed extensively to the biological interpretation and helped drafting the text. KM supervised the work, designed the analyses and wrote the manuscript. JV contributed to helpful discussions and critically read the manuscript. All authors read and approved the final manuscript.

## Supplementary Material

Additional file 1: Table S1 Characteristics of module network as reconstructed by CLR and LeMoNe. Click here for file

Additional file 2: Table S2 Overview of the sRNAs in the different modules. Click here for file

Additional file 3: Table S3 Performance of inferring sRNA-target interactions. Click here for file

Additional file 4 Module overview. Click here for file

Additional file 5: Table S4 Description of the expression compendium. Click here for file

Additional file 6: Table S5 Probe information. Click here for file

Additional file 7 Performance tests to optimize sequence-based sRNA-target predictions. Click here for file

Additional file 8: Table S6 Predictions of sRNA targets based on intaRNA and TargetRNA. Click here for file

Additional file 9: Table S7 List of regulators. Click here for file

Additional file 10: Table S8 Description of our benchmark of known sRNA-target interactions. Click here for file
