# Supplementary material for: Inferring the relation between transcriptional and posttranscriptional regulation from expression compendia
Source: BMC Microbiol. 2014 Jan 27;14:14. doi: 10.1186/1471-2180-14-14 (PMC3948049; doi:10.1186/1471-2180-14-14)
Supplement: Additional file 1: Table S1 — Characteristics of module network as reconstructed by CLR and LeMoNe. [file 1471-2180-14-14-S1.pdf]

Additional file 1 - Supplementary Table 1 Characteristics of module network as reconstructed by CLR and LeMoNe

[a]Module ID	[b]Number of genes	[c]Number of conditions	[d]Functional overrepresentation (KEGG and GO)	[e]Assigned sRNAs	[f]sRNAs for which predicted/known targets are present in the modules	[g]Assigned TFs	[h]Enriched TFs
1	48	13	(ECOCYC)SALVADEHYPOX-PWY:salvage pathways of adenine, hypoxanthine, and their nucleosides(0.00054485)<br>(GO) 9308 :amine metabolic process 4,5572E-2<br>(GO) 6807 :nitrogen compound metabolic process 3,8009E-2<br>(GO) 55114 :oxidation reduction 3,8009E-2			**GadE**<br>**CueR**<br>YgiV(CLR)<br>MlrA(CLR)	AppY (0,0001472179)<br>**CueR** (0,000043962)<br>Fnr (0,0001909592)<br>Fis (0,0001153955)<br>**GadE** (0,0003415078)<br>GadW (0,000432771)<br>GadX (0,0001442004)<br>IscR (0,0001690305)<br>NarL (0,000213676)<br>NarP (0,000000814)
2	35	7	(ECOCYC)GALACTARDEG-PWY:D-galactarate degradation(6.8171e-006)<br>(ECOCYC)GLUCARDEG-PWY:D-glucarate degradation(4.4669e-008)<br>(GO) 43649 :dicarboxylic acid catabolic process 6,9170E-3<br>(GO) 46395 :carboxylic acid catabolic process			YbaQ (LeMoNe)<br>DcuR (LeMoNe)<br>GutM (LeMoNe)	MelR (0,0001911569)

			6,9170E-3				
			(GO) 16054 :organic acid catabolic process 7,3603E-3				
3	43	8	(ECOCYC)CYANCAT-PWY:cyanate degradation(8.0136e-005)	**ryhB**			CRP (0,0002162127)
			(ECOCYC)GALACTUROCAT-PWY:D-galacturonate degradation(3.2978e-006)				CynR (0,0005758219)
			(ECOCYC)GLUCUROCAT-PWY:β-D-glucuronide degradation(7.6878e-007)				ExuR (0,000050014)
							Fis (0,000245568)
							FruR (0,0006043947)
			(GO) 6810 :transport 9,2959E-3				Fur (0,0001901954)
			(GO) 51234 :establishment of localization 9,2959E-3				MtlR (0,0002897477)
			(GO) 15980 :energy derivation by oxidation of organic compounds 9,2959E-3				NanR (0,000050014)
							PdhR (0,0008015672)
4	45	5	(ECOCYC)PWY0-41:allantoin degradation I(4.6854e-008)			**LsrR**<br>HcaR (LeMoNe)<br>PuuR (LeMoNe)	AllR (0,0001212253)
			(ECOCYC)GLYCOLATEMET-PWY:glycolate degradation I(5.5756e-005)				CRP (0,0003568554)
							DnaA (0,0006309069)
			(GO) 255 :allantoin metabolic process 5,8499E-3				GntR (0,0002190156)
			(GO) 6144 :purine base metabolic process 5,8499E-3				
			(GO) 9112 :nucleobase				**LsrR**

			metabolic process<br>3,6561E-2				(0,000000012)
5	34	8	N/A			RacR (LeMoNe)<br>RelE (LeMoNe)	N/A
6	53	17	(ECOCYC)ENTBACSYN-PWY:enterobactin biosynthesis(0)<br>(GO) 15674 :di-, tri-valent inorganic cation transport<br>8,3706E-28<br>(GO) 41 :transition metal ion transport<br>4,5176E-25<br>(GO) 16226 :iron-sulfur cluster assembly<br>1,4182E-3	**ryhB**	**ryhB** (sufB^{u}shiA^{l})	**IscR**	Fur<br>(0,00007763)<br>**IscR**<br>(0,0002492163)<br>MntR<br>(0,0004414737)<br>NsrR<br>(0,000472961)<br>OxyR<br>(0,000151701)
7	1	22	N/A	c0343<br>(CLR)		ArsR(CLR)<br>DeoR(CLR)	Fur<br>(0,00007763)
8	78	8	N/A	oxyS<br>(LeMoNe)	oxyS (rimK^{u}inaA^{u}mltC^{u})	Fur (LeMoNe)<br>MarA (LeMoNe)	EnvR<br>(0,0003232437)<br>MarA$^{\#}$<br>(0,0006063839)<br>PhoP<br>(0,0003617701)<br>Rob<br>(0,0002503464)<br>SoxR<br>(0,0009583286)<br>SoxS<br>(0,0008587882)
9	19	3	(ECOCYC)ARGSYN-PWY:arginine biosynthesis I(0.00094)	N/A		N/A	ArgR<br>(0,0005131897)<br>DgsA<br>(0,0008110324)

10	56	6	(ECOCYC)GLYCEROLMETAB-PWY:glycerol degradation II(0)<br>(ECOCYC)GALACTUROCAT-PWY:D-galacturonate degradation(9.8147e-006)<br>(ECOCYC)GLUCUROCAT-PWY:β-D-glucuronide degradation(3.0155e-006)<br>(ECOCYC)ARABCAT-PWY:L-arabinose degradation(0.00018)<br>(GO) 8643 :catabolic process 4,4960E-2<br>(GO) 5975 :carbohydrate metabolic process 3,0053E-2<br>(GO) 8643 :carbohydrate transport 2,6590E-9	ryhB (LeMoNe)		GutM (LeMoNe)	AgaR (0,0003183022)<br>GutM[#] (8,68845664E-007)<br>AraC (0,0003183022)<br>CRP (0,0003193036)<br>ExuR (0,0001110681)<br>LacI (0,0004931432)<br>MelR (0,0004931432)<br>TorR (0,0004205008)<br>Uidr (0,0009780537)
11	27	6	(ECOCYC)PWY0-44:D-allose degradation(0)			**HcaR**<br>PuuR(CLR)<br>Glcc(CLR)	ArcA (0,000000253)<br>CRP (0,0001814015)<br>DnaA (0,0002249404)<br>Fur (0,0001696494)<br>GadE (0,0008764505)<br>PdhR (0,0001986533)<br>AlsR

							(0,0005580126)
12	28	5	(ECOCYC)NRI-PWY:Nitrogen Regulation Two-Component System(0.00040131)<br>(ECOCYC)ASPARAGINESYN-PWY:asparagine biosynthesis III(0.00079608)<br>(ECOCYC)GLNSYN-PWY:glutamine biosynthesis I(0)	tpke70 (LeMoNe)	tpke70 (ybeD^{u})	CspE (LeMoNe)<br>MalT (LeMoNe)<br>NadR (LeMoNe)	ArcA (0,000048091)<br>CysB (0,0002421683)<br>GadX (0,0005130548)<br>NtrC (0,0001617529)<br>RutR (0,000000033)
13	65	31	(ECOCYC)CHE-PWY:Chemotactic Signal Transduction System(0)<br>(GO) 51674 :localization of cell 2,2753E-47<br>(GO) 51179 :localization 6,5446E-8<br>(GO) 8151 :cellular process 4,5780E-3	micF (LeMoNe)<br>rydB (LeMoNe)	rydB (fliI^{u})	YjjM(CLR)<br>MalT(CLR)<br>NanR(CLR)<br>TdcA(CLR)	CpxR (0,0002037391)<br>FlhDC (0,0001702573)
14	60	16	(ECOCYC)GLYCEROLMETAB-PWY:glycerol degradation II(0)<br>(ECOCYC)FERMENTATION-PWY:mixed acid fermentation(4.3603e-005)<br>(ECOCYC)ANARESPACC-PWY:respiration (anaerobic)--electron acceptors reaction list(4.3603e-005)<br>(GO) 55114 :oxidation reduction 2,3717E-7<br>(GO) 22900 :electron transport chain 4,0491E-4			**AdiY**<br>**IscR**<br>MalI (LeMoNe)	FNR (0,0003842098)<br>FhlA (0,000000004)<br>IHF (0,0001778752)

			(GO) 6091 :generation of precursor metabolites and energy<br>1,0698E-2				ModE (0,0003903815)<br>NarL (0,0001427826)<br>NsrR (0,000000011)
15	45	17	N/A	sroD (LeMoNe)	sroD (yceO[u])	**YdeO**<br>YgeH(CLR)	EvgA (0,000013011)<br>LrhA (0,0003175647)
16	28	6	(ECOCYC)HCAMHPDEG-PWY:3-phenylpropionate and 3-(3-hydroxyphenyl)propionate degradation(0.000157)<br>(GO) 19439 :aromatic compound catabolic process<br>1,1716E-2<br>(GO) 44248 :cellular catabolic process<br>1,1716E-2<br>(GO) 9056 :catabolic process<br>1,1716E-2			XylR (LeMoNe)<br>Fis (LeMoNe)<br>SdiA (LeMoNe)	BolA (0,0004019914)<br>MhpR (0,0006005639)<br>Zur (0,0006005639)
17	31	11	(ECOCYC)LCYSDEG-PWY:L-cysteine degradation II(0.00097931)<br>(ECOCYC)TRYPDEG-PWY:tryptophan degradation II (via pyruvate)(0)<br>(ECOCYC)GLYCOCAT-PWY:glycogen degradation(0.0009362)<br>(ECOCYC)TREDEGLOW-PWY:trehalose degradation I (low osmolarity)(0.00054676)<br>(GO) 8643 :carbohydrate transport<br>2,3394E-2<br>(GO) 6812 :cation transport	isrB (LeMoNe)<br>micF (CLR)	micF (ompF[lu])	**MalT**<br>**YgiV**<br>**CueR**	CRP (0,000024247)<br>**MalT** (3,3731899E-009)<br>CpxR (0,0007081689)<br>MprA

			2,4656E-2<br>(GO) 6811 :ion transport<br>2,5828E-3				(0,0007374145)<br>OmpR<br>(0,0002139316)<br>PdhR<br>(0,0003018939)
18	23	21	(ECOCYC)ACETATEUTIL-PWY:acetate utilization(0.00053283)<br>(ECOCYC)FAO-PWY:fatty acid oxidation pathway I(4.5201e-005)<br>(GO) 6629 :lipid metabolic process<br>2,5771E-2<br>(GO) 15889 :cobalamin transport<br>7,7023E-3<br>(GO) 51180 :vitamin transport<br>9,1513E-3	ryeA (LeMoNe)	ryeA (btuE[u])	ArcA(CLR)	GlcC<br>(0,0005626667)
19	39	10	(ECOCYC)HOMOSER-METSYN-PWY:methionine biosynthesis I(3.1095e-005)<br>(GO) 9067 :aspartate family amino acid biosynthetic process<br>(GO) 9309 :amine biosynthetic process<br>1,6438E-2<br>(GO) 44271 :nitrogen compound biosynthetic process<br>1,6438E-2	**ryhB**		PuuR (LeMoNe)	MarR<br>(0,0002379145)<br>MetJ<br>(0,0002890061)<br>NanR<br>(0,0007017692)<br>NarL<br>(0,0004573207)<br>NarP<br>(0,0009047063)<br>NsrR<br>(0,000000031)<br>TorR<br>(0,000002689)

20	53	6	(ECOCYC)PWY0-301:L-ascorbate degradation(0.00024205)<br>(GO) 65007 :biological regulation 2,4214E-4<br>(GO) 6139 :nucleobase, nucleoside, nucleotide and nucleic acid metabolic process 2,2264E-3<br>(GO) 8151 :cellular process 4,3846E-2	ryfA (LeMoNe)	ryfA (znuC^{u})	SoxR (LeMoNe)<br>IclR (LeMoNe)	MalI (0,0001483283)<br>MarR (0,0004414737)<br>OxyR (0,000151701)<br>SoxR$^{\#}$ (0,0004414737)<br>UlaR (0,0008759862)
21	15	8	(GO) 10043 :response to zinc ion 3,5025E-2<br>(GO) 9409 :response to cold 4,2016E-2<br>(GO) 51869 :response to stimulus 2,8452E-2			**YefM**<br>CspA (LeMoNe)	CpxR (0,0009376863)<br>**YefM** (1,1302228578924 E-005)
22	26	22	(ECOCYC)PWY0-163:salvage pathways of pyrimidine ribonucleotides(0.00055008)<br>(ECOCYC)DRIBOPMET-PWY:(deoxy)ribose phosphate degradation(0.00055008)<br>(ECOCYC)GALACTMETAB-PWY:galactose degradation I(1.9613e-006)<br>(ECOCYC)GLYOXYLATE-BYPASS:glyoxylate cycle(0.00032001)<br>(GO) 6066 :alcohol metabolic process 9,3919E-3<br>(GO) 44262 :cellular carbohydrate metabolic	spf (CLR)<br>is128 (CLR)<br>isrB (CLR)<br>c0614 (CLR)	spf (mdH^{u}**;** gltA^{l})	**Fis**<br>YefM (LeMoNe)<br>Tpr(CLR)	BirA (0,0003459495)<br>CRP (0,000002671)<br>DeoR (0,0007211142)<br>GatR (0,000516999)<br>NagC (0,0002074892)<br>RbsR (0,000516999)

			process<br>2,6063E-2<br>(GO) 8643 :carbohydrate transport<br>1,0882E-4				
23	58	9	N/A	ryjA (CLR)		**BolA**	CsgD (0,0002594368)<br>HipB (0,0001779293)<br>RelEB (0,0005291632)<br>YoeB-YefM (0,0001779293)<br>MqsR-YgiT (0,0001779293)
24	14	13	(GO) 16265 :death<br>2,3066E-2<br>(GO) 8219 :cell death<br>2,3066E-2<br>(GO) 32502 :developmental process<br>2,5051E-2	**micF**<br>isrB (LeMoNe)			RstA (0,0004342837)
25	23	19	N/A			**LsrR**<br>IhfA (LeMoNe)<br>GadW (LeMoNe)	CRP (0,0002364086)<br>**LsrR** (0,0009583219)<br>NhaR (0,0005626667)
26	29	3	N/A	N/A		N/A	N/A
27	13	16	N/A	N/A		**YrbA**<br>MprA(CLR)	N/A
28	19	5	(ECOCYC)PWY0-21:BarA-UvrY Two-Component Signal Transduction System(0.00036013)	psrD (LeMoNe)		PaaX (LeMoNe)<br>IhfA (LeMoNe)	CspA (0,0008110324)
29	32	13	(GO) 42743 :hydrogen peroxide metabolic process<br>4,3280E-2<br>(GO) 7047 :cell wall			**YiaG**	CpxR (0,000801272)

			organization and biogenesis<br>3,7150E-2<br>(GO) 45229 :external encapsulating structure organization and biogenesis<br>4,3024E-2				
30	33	11	(ECOCYC)HOMOSER-METSYN-PWY:methionine biosynthesis I(1.9123e-007)<br>(ECOCYC)SAM-PWY:S-adenosylmethionine biosynthesis(3.5428e-005)<br>(GO) 19752 :carboxylic acid metabolic process<br>1,9876E-4<br>(GO) 6082 :organic acid metabolic process<br>2,2199E-4<br>(GO) 44249 :cellular biosynthetic process<br>1,6009E-2	ryfA (LeMoNe)<br>ryhB (LeMoNe)		Cbl (LeMoNe)<br>MarR (LeMoNe)<br>MetR (CLR)	CusR (0,0008362833)<br>CysB (0,0007415272)<br>MetJ (0,0001747189)<br>MetR$^{\#}$ (0,0008362833)<br>PhoP (0,0001261138)
31	28	11	(ECOCYC)PWY0-381:glycerol and glycerophosphodiester degradation(0.00068877)<br>(ECOCYC)PWY0-461:lysine degradation I(0.00079608)<br>(GO) 6059 :hexitol metabolic process<br>4,1919E-5<br>(GO) 19751 :polyol metabolic process<br>2,6429E-5<br>(GO) 6066 :alcohol metabolic process			GreA (LeMoNe)<br>**GatR**<br>LldR(CLR)	ArcA (0,000048091)<br>CadC (0,000121573)<br>GadE (0,000039734)<br>GadX (0,0005130548)<br>**GatR** (0,0006005639)<br>GcvA (0,0004019914)<br>GlpR (0,00002009)

			3,8811E-2				
32	12	9	(ECOCYC)ARABCAT-PWY:L-arabinose degradation(8.6363e-006)	**RybA**		BolA (LeMoNe)<br>PaaX (LeMoNe)	AraC (0,0003853274)
			(ECOCYC)LYXMET-PWY:L-lyxose degradation(0.00044227)				
			(ECOCYC)RHAMCAT-PWY:rhamnose degradation(0.00016563)				
			(GO) 6066 :alcohol metabolic process 7,1136E-3				
			(GO) 44262 :cellular carbohydrate metabolic process 7,1136E-3				RhaS (0,0001059059)
			(GO) 5975 :carbohydrate metabolic process 2,8910E-2				
33	31	18	(ECOCYC)HOMOSER-METSYN-PWY:methionine biosynthesis I(1.3715e-007)	ryhB (LeMoNe)<br>ryfA (LeMoNe)		Cbl (LeMoNe)<br>MetR (CLR)	CusR (0,0007374145)
			(ECOCYC)SAM-PWY:S-adenosylmethionine biosynthesis(2.9188e-005)				MetJ (0,0001445487)
			(ECOCYC)LCYSDEG-PWY:L-cysteine degradation II(0.00097931)				MetR# (0,0007374145)
			(GO) 6807 :carboxylic acid metabolic process 5,9299E-4				
			(GO) 6082 :organic acid metabolic process 6,5786E-4				
			(GO) 44249 :cellular biosynthetic process 3,2885E-2				
34	25	21	N/A	ryjA (CLR)		**BolA**<br>**YjdC**	DnaA (0,0001074673)

						MlrA(CLR)<br>YiaG(CLR)<br>YgiV(CLR)	FlhDC (0,0006814587)<br>ModE (0,00071051)<br>NarP (0,0009047063)<br>PurR (0,0001510348)
35	39	5	(ECOCYC)DENOVOPURINE2-PWY:purine nucleotides _de novo_ biosynthesis I(5.5511e-016)<br>(ECOCYC)PWY0-662:PRPP biosynthesis I(0)<br>(GO) 55086 :nucleobase, nucleoside and nucleotide metabolic process 1,4744E-10<br>(GO) 6139 :nucleobase, nucleoside, nucleotide and nucleic acid metabolic process 2,0346E-3	N/A		N/A	DnaA (0,0001074673)<br>FlhDC (0,0006814587)<br>ModE (0,00071051)<br>NarP (0,0009047063)<br>PurR (0,0001510348)
36	31	17	(ECOCYC)PWY0-381:glycerol and glycerophosphodiester degradation(1.6168e-011)<br>(ECOCYC)PWY0-461:lysine degradation I(0.00097931)<br>(GO) 6066 :alcohol metabolic process 1,6269E-15<br>(GO) 19751 :polyol metabolic process 2,3335E-14	N/A		Fis(LeMoNe)	ArcA (0,0007579047)<br>CRP (0,000000007)<br>CadC (0,0001494844)<br>DhaR (0,000297628)<br>DsdC (0,0001494844)<br>Fis[#] (0,0001151776)<br>GadX (0,0006959205)<br>GlpR

							(0,000000269)<br>NagC (0,000009788)<br>NanR (0,000018409)
37	27	19	N/A			LeuO (LeMoNe)<br>YjgJ (LeMoNe)<br>HcaR (LeMoNe)	H-NS (0,0007781998)
38	39	14	(GO) 9437 :carnitine metabolic process 3,7995E-2<br>(ECOCYC)PWY0-981:taurine degradation IV(0)			AdiY (LeMoNe)<br>LeuO (LeMoNe)<br>YdiP (LeMoNe)	SgrR (0,0004731066)
39	25	13	N/A			RacR (LeMoNe)<br>AlpA (LeMoNe)	ChbR (0,0004775261)<br>DicA (0,0003194867)
40	62	10	(ECOCYC)PWY0-381:glycerol and glycerophosphodiester degradation(1.6913e-005)<br>(ECOCYC)BGALACT-PWY:lactose degradation III(0)<br>(GO) 5988 :lactose metabolic process 2,6620E-2<br>(GO) 9401 :phosphoenolpyruvate-dependent sugar phosphotransferase system 1,9484E-2<br>(GO) 8643 :carbohydrate transport 1,9484E-2			**GutM**<br>TdcA (LeMoNe)<br>CytR (LeMoNe)	ArcA (0,0003636762)<br>CRP (0,000000002)<br>CadC (0,0006049746)<br>CytR[#] (0,0005683175)<br>DcuR (0,000095189)<br>DsdC (0,0006049746)<br>FNR (0,0001330186)<br>Fis (0,0002410244)<br>FlhDC (0,0001257713)

							GlpR (0,000000063)
							**GutM** (0,000095189)
							GutR (0,000095189)
							H-NS (0,000246218)
							LacI (0,0006049746)
							NagC (0,0001581966)
							NanR (0,000002602)
							NarL (0,0001806274)
							NarP (0,0006126946)
41	19	7	(ECOCYC)PHOR-PWY:PhoRB Two-Component Signal Transduction System(0)			PhoB (LeMoNe)<br>YgiV (LeMoNe)<br>YjjM (LeMoNe)	FNR (0,0009562891)
			(GO) 6810 :transport 8,3143E-3				FlhDC (0,0003448519)
							IHF (0,0002555689)
			(GO) 51234 :establishment of localization 8,3143E-3				PhoB# (0,000016244)
			(GO) 51179 :localization 2,6796E-4				
42	2	19	N/A	**rydC**		NadR(CLR)	N/A
43	34	11	(ECOCYC)SO4ASSIM-PWY:sulfate assimilation(0)			**Cbl**<br>RpiR (LeMoNe)<br>YjdC (LeMoNe)	BolA (0,0005949412)
			(GO) 103 :sulfate assimilation 4,6515E-11				CysB (0,0008103202)
			(GO) 6790 :sulfur metabolic				MhpR

			process<br>1,9173E-4<br>(GO) 6811 :ion transport<br>3,7788E-3				(0,0008879995)<br>Zur<br>(0,0008879995)
44	27	27	(ECOCYC)TRESYN-PWY:trehalose biosynthesis I(0.00073922)<br>(ECOCYC)PWY0-541:cyclopropane fatty acid (CFA) biosynthesis(0)<br>(ECOCYC)GLUTAMINDEG-PWY:glutamine degradation IV(0.00073922)			**YiaG**<br>**GadE**<br>**YjdC**<br>MlrA (CLR)<br>YgiV (CLR)<br>BolA (CLR)<br>CueR (CLR)	**GadE**<br>(0,0008764505)<br>GadX<br>(0,0004598427)<br>TorR<br>(0,0007781998)<br>YdeO<br>(0,0005580126)
45	28	27	(ECOCYC)TRESYN-PWY:trehalose biosynthesis I(0.00079608)<br>(ECOCYC)PUTDEG-PWY:putrescine degradation(0.00079608)			**YiaG**<br>**YjdC**<br>**IhfA**	N/A
46	22	22	N/A	c0299<br>(CLR)<br>**c0067**		**AlpA**<br>YjhU (LeMoNe)	ChbR<br>(0,00036838)<br>DicA<br>(0,0002463484)
47	45	25	(ECOCYC)PWY0-541:cyclopropane fatty acid (CFA) biosynthesis(0)<br>(GO) 6520 :amino acid metabolic process<br>4,7037E-2<br>(GO) 6519 :amino acid and derivative metabolic process<br>4,7037E-2<br>(GO) 9308 :amine metabolic process<br>4,7037E-2	gadY<br>(CLR)	gadY<br>(cueR∪poxB∪cbpA∪ygiW∪)	**GadE**<br>**YgiV**<br>CueR (CLR)<br>TdcA(CLR)<br>MlrA(CLR)<br>YjdC(CLR)	Ada<br>(0,0006309069)<br>AppY<br>(0,0001212253)<br>ArcA<br>(0,000184949)<br>EvgA<br>(0,000013011)<br>Fis<br>(0,0003529397)<br>**GadE**<br>(0,0002654715)

							GadW (0,0003571069)<br>GadX (0,00000014)<br>IscR (0,000131032)<br>MarA (0,0006529555)<br>NarL (0,000140525)
48	24	15	(GO) 19222 :regulation of metabolic process 2,4168E-2<br>(GO) 10468 :regulation of gene expression 2,4168E-2<br>(GO) 9058 :biosynthetic process 2,2950E-2	**is128**<br>**tp2**<br>ryfA (LeMoNe)		**PdhR**	CynR (0,000177041)<br>MhpR (0,0004395966)
49	52	17	(GO) 44237 :cellular metabolic process 1,2897E-9<br>(GO) 8152 :metabolic process 7,1852E-6<br>(GO) 8151 :cellular process 9,4849E-8	psrD (CLR)	psrD (rpsN[u])	**RplD**<br>RpsJ(CLR)<br>YbrA(CLR)<br>Fis(CLR)<br>NusA(CLR)	ArcA (0,0005191334)<br>FNR (0,000000085)
50	63	4	(ECOCYC)MANGDPMET-PWY:GDP-mannose metabolism(0.00098369)<br>(GO) 43284 :biopolymer biosynthetic process 3,3074E-5<br>(GO) 9059 :macromolecule biosynthetic process 4,8690E-4<br>(GO) 43283 :biopolymer metabolic process 7,5839E-3	c0343 (LeMoNe)	c0343 (rutR[u])	AllS (LeMoNe)<br>YjhU (LeMoNe)<br>MatA (LeMoNe)	LeuO (0,000595794)<br>RcsAB (0,0002978481)<br>TorR (0,000595794)

51	14	11	(GO) 10447 :response to acidity<br>4,6271E-2<br>(GO) 9268 :response to pH<br>4,6271E-2<br>(GO) 6944 :membrane fusion<br>4,6271E-2			AcrR (LeMoNe)<br>DnaA (LeMoNe)	NorR (0,0001458409)<br>RstA (0,0004342837)
52	4	20	N/A	sraA (CLR)		QseB (LeMoNe)	FhlA (0,0001051606)<br>GlcC (0,0002037983)<br>HyfR (0,0006345877)<br>PspF (0,0002037983)
53	14	25	(GO) 42773 :ATP synthesis coupled electron transport<br>3,4059E-2<br>(GO) 22904 :respiratory electron transport chain<br>4,6859E-2<br>(GO) 6119 :oxidative phosphorylation<br>4,6859E-2	**dicF**<br>tpke11 (CLR)<br>sroA (CLR)<br>rydC (CLR)<br>c0343 (CLR)		BolA (LeMoNe)<br>ArsR(LeMoNe)	FhlA (0,0001051606)<br>GlcC (0,0002037983)<br>HyfR (0,0006345877)<br>PspF (0,0002037983)
54	26	32	(GO) 32196 :transposition<br>3,0049E-4<br>(GO) 6310 :DNA recombination<br>7,1095E-4<br>(GO) 6259 :DNA metabolic process<br>1,7560E-2	sroD (CLR)<br>c0664 (CLR)<br>c0299 (CLR)<br>**c0067**		**AlpA**<br>YhcF (LeMoNe)	HcaR (0,000516999)<br>DicA (0,0003459495)
55	34	28	(GO) 6318 :transposition, DNA-mediated<br>1,9150E-2	sroD (CLR)<br>c0664 (CLR)<br>**c0299**<br>c0067	sroD (yihF[u])<br>c0067 (yhjB[u])	YjfJ (LeMoNe)<br>AlpA(CLR)	BglJ (0,0003587392)<br>HcaR (0,0008879995)<br>StpA (0,0003587392)

				(CLR)			
56	39	15	N/A	**ryhB**		BglJ (LeMoNe)<br>MarA(CLR)	IdnR (0,0004731066)
							MarA# (0,0003747925)
							MarR (0,0002379145)
							NanR (0,0007017692)
							NarL (0,0009047063)
							NarP (0,0008068999)
							NsrR (0,000044777)
							OxyR (0,0005086512)
							Rob (0,0005001796)
							SoxS (0,000002689)
							TorR (0,0003587392)
57	34	17	(ECOCYC)LEUSYN-PWY:leucine biosynthesis(1.7681e-005)			Cbl (LeMoNe)<br>UhpA (LeMoNe)<br>MetJ (LeMoNe)<br>MetR (CLR)	CusR (0,0008879995)
			(ECOCYC)HOMOSER-METSYN-PWY:methionine biosynthesis I(2.2401e-007)				MetJ# (0,0001912264)
			(ECOCYC)SAM-PWY:S-adenosylmethionine biosynthesis(3.8857e-005)				
			(GO) 6082 :organic acid metabolic process 1,7233E-6				
			(GO) 44249 :cellular biosynthetic process 1,1876E-4				MetR# (0,0008879995)
			(GO) 9058 :biosynthetic				PhoP

			process<br>3,9062E-2				(0,0001421073)
58	56	20	(ECOCYC)SALVADEHYPOX-PWY:salvage pathways of adenine, hypoxanthine, and their nucleosides(0.00099396)<br><br>(GO) 19752 :carboxylic acid metabolic process<br>4,7532E-2<br><br>(GO) 55114 :oxidation reduction<br>1,6319E-2<br><br>(GO) 6139 :nucleobase, nucleoside, nucleotide and nucleic acid metabolic process<br>4,2763E-2	gadY (CLR)	gadY (cueR^{u}poxB^{u}cbpA^{u}xdhC^u)	CueR (CLR)<br>**GadE**	Ada (0,0009780537)<br>ArcA (0,0008652516)<br>EvgA (0,000845925)<br>Fis (0,0004384941)<br>**GadE** (0,000000003)<br>GadW (0,000000565)<br>GadX (0,000000017)<br>IscR (0,0003087722)<br>NarL (0,0005661985)<br>NarP (0,0003806477)<br>YdeO (0,0004931432)<br>AppY (0,0002336417)
59	8	21	N/A	rdlD (CLR)<br>sroD (CLR)<br>sroA (CLR)	sroA (insH^u)	DicA (LeMoNe)	SgrR (0,0001077982)
60	33	17	(ECOCYC)TRYPDEG-PWY:tryptophan degradation II (via pyruvate)(0)<br>(ECOCYC)TRYPDEG-	isrB (LeMoNe)		CueR (LeMoNe)	CpxR (0,000000007)<br>MprA (0,0008362833)

			PWY:tryptophan degradation II (via pyruvate)(0)				OmpR (0,0002584037)
			(ECOCYC)TREDEGLOW-PWY:trehalose degradation I (low osmolarity)(0.00066052) (ECOCYC)TRYPDEG-PWY:tryptophan degradation II (via pyruvate)(0)				PhoP (0,0001261138)
							Rob (0,0003084687)
							TreR (0,000056834)
61	44	13	(ECOCYC)NARX-PWY:NarX Nitrate/Nitrite-Dependent Two-Component Regulatory System(8.5999e-005)	tpke70 (CLR)	tpke70 (napG^{u}napD^{u})	YahA (LeMoNe) AdiY (LeMoNe) CdaR(CLR)	CdaR$^{\#}$ (0,0001132822)
			(ECOCYC)GALACTARDEG-PWY:D-galactarate degradation(1.4944e-007)				FNR (0,000014875)
			(ECOCYC)GLUCARDEG-PWY:D-glucarate degradation(1.754e-005)				FlhDC (0,0001360082)
			(GO) 51179 :localization				IHF (0,0002952361)
							IscR (0,000000004)
							MurR (0,0001018277)
							NarL (0,0001212618)
							NarP (0,000119884)
							RstA (0,0001132822)
							TdcA (0,0006030543)
62	42	22	(GO) 51244 :regulation of cellular process 2,4877E-2			**YgeH**	LrhA (0,0002763141)
			(GO) 50791 :regulation of biological process 2,4877E-2				
			(GO) 65007 :biological				DicA

			regulation<br>3,3916E-2				(0,0009096927)
63	21	20	(GO) 16070 :RNA metabolic process<br>1,2480E-4<br>(GO) 10467 :gene expression<br>1,1199E-3<br>(GO) 6139 :nucleobase, nucleoside, nucleotide and nucleic acid metabolic process<br>2,1754E-2			**GreA**<br>HepA (LeMoNe)<br>Fis(CLR)<br>PuuR(CLR)	Zur<br>(0,0003350986)
64	26	13	(ECOCYC)BAS-PWY:BasSR Two-Component Signal Transduction System(0)<br>(GO) 44238 :biopolymer biosynthetic process<br>2,1688E-2<br>(GO) 43283 :biopolymer metabolic process<br>4,9910E-2<br>(GO) 44238 :primary metabolic process<br>3,8402E-2	rdlA<br>(CLR)<br>isrC<br>(CLR)		YdhM (LeMoNe)<br>NarP (LeMoNe)<br>XylR(CLR)	RhaS<br>(0,000516999)
65	73	9	(ECOCYC)ILEUSYN-PWY:isoleucine biosynthesis I(2.3834e-005)<br>(ECOCYC)ILEUSYN-PWY:isoleucine biosynthesis I(2.3834e-005)<br>(GO) 9058 :biosynthetic process<br>9,5921E-3<br>(GO) 44237 :cellular metabolic process<br>2,5367E-2	c0343<br>(LeMoNe)	c0343 (ilvG[u])	Cbl (LeMoNe)<br>YhcF(CLR)	CspA<br>(0,0002828786)<br>IlvY<br>(0,0002828786)<br>Lrp<br>(0,0002128918)<br>Nac<br>(0,0002828786)<br>SgrR<br>(0,0003637996)<br>TyrR<br>(0,0009185504)

			(GO) 8152 :metabolic process<br>3,3857E-2				
66	28	9	(GO) 51246 :regulation of protein metabolic process<br>4,8671E-2<br>(GO) 46677 :response to antibiotic<br>4,8671E-2<br>(GO) 6416 :translation<br>4,8671E-2			MntR (LeMoNe)	NorR (0,000121573)<br>UlaR (0,0008374118)
67	26	22	(ECOCYC)PHOR-PWY:PhoRB Two-Component Signal Transduction System(0)<br>(GO) 6810 :transport<br>2,6759E-6<br>(GO) 51234 :establishment of localization<br>2,6759E-6<br>(GO) 51179 :localization<br>3,8876E-6			StpA (LeMoNe)<br>PdhR (LeMoNe)	IHF (0,0002343247)<br>Lrp (0,0002467516)<br>PepA (0,0001045596)<br>PhoB (0,000060018)<br>PurR (0,0007828703)
68	30	16	(ECOCYC)BGALACT-PWY:lactose degradation III(0)<br>(GO) 8643 :carbohydrate transport<br>2,0357E-2<br>(GO) 7165 :signal transduction<br>2,0357E-2<br>(GO) 7154 :cell communication<br>2,5976E-2			**GutM**<br>CueR(CLR)<br>DcuR(CLR)<br>GadE(CLR)<br>TdcA(CLR)<br>YgiV(CLR)	ArgP (0,0009621947)<br>CadC (0,000139862)<br>**GutM** (0,0009621947)<br>GutR (0,0009621947)<br>H-NS (0,0004770907)<br>LacI (0,0002785127)<br>Lrp (0,0004973938)
69	34	28	(ECOCYC)GALACTARDEG-PWY:D-galactarate	ryhB (LeMoNe)	ryhB (dmsA^{u}frdA^{u})	**AdiY**<br>IscR(CLR)	ArcA (0,0001814898)

			degradation(6.0431e-006)				
			(ECOCYC)GLUCARDEG-PWY:D-glucarate degradation(6.0431e-006)				FNR (0,000000699)
			(ECOCYC)ANARESPACC-PWY:respiration (anaerobic)--electron acceptors reaction list(7.5085e-006)				GcvA (0,0005949412)
			(GO) 15675 :nickel ion transport 3,1391E-2				H-NS (0,0001309659)
							NarL (0,0002097502)
			(GO) 55114 :oxidation reduction 4,1037E-4				NikR (0,0008879995)
70	40	20	(ECOCYC)TRPSYN-PWY:tryptophan biosynthesis(1.1848e-005)	**ryhB**	**ryhB** (trpL^{u})	Cbl (LeMoNe) PurR (LeMoNe) YahA (CLR)	NadR (0,0003117852)
			(ECOCYC)THISYN-PWY:thiamin biosynthesis(1.0097e-005)				PurR$^{\#}$ (0,0001669755)
			(ECOCYC)DENOVOPURINE2-PWY:purine nucleotides _de novo_ biosynthesis I(1.8998e-009)				
			(GO) 44249 :cellular biosynthetic process 1,2891E-6				
			(GO) 9058 :biosynthetic process 6,6580E-4				
			(GO) 44237 :cellular metabolic process 2,8440E-2				TrpR (0,000153697)
71	26	26	N/A	**c0299**		**YbhD**	DicA (0,0003459495)
72	38	16	(ECOCYC)TRPSYN-PWY:tryptophan biosynthesis(9.5861e-006)	**ryhB**	**ryhB** (trpL^{u})	**YahA** MntR (LeMoNe) QseB (LeMoNe)	NadR (0,0002673342)
			(ECOCYC)THISYN-				

			PWY:thiamin biosynthesis(7.7567e-006)<br>(ECOCYC)DENOVOPURINE2-PWY:purine nucleotides _de novo_ biosynthesis I(5.3642e-007)<br>(GO) 9058 :organic acid metabolic process 1,7947E-2<br>(GO) 44249 :cellular biosynthetic process 1,6310E-4<br>(GO) 9058 :biosynthetic process 1,3250E-2				PurR (0,000000195)<br>TrpR (0,000131647)
73	45	13	(GO) 44237 :cellular metabolic process 3,4774E-5<br>(GO) 8151 :cellular process 2,0808E-3<br>(GO) 8152 :metabolic process 4,4318E-3			**RplD**<br>RpsJ(CLR)<br>YrbA(CLR)	ArcA (0,000184949)<br>FNR (0,0004877495)
74	29	20	(ECOCYC)GLUCUROCAT-PWY:β-D-glucuronide degradation(0.00044656)<br>(ECOCYC)ENTNER-DOUDOROFF-PWY:Entner-Doudoroff pathway I(9.3004e-005)	**isrB**<br>spf(CLR)<br>is128 (CLR)<br>sroH (CLR)	isrB (omrB[u])	**Fis**<br>YifE(CLR)<br>Tpr(CLR)	N/A
75	68	17	(ECOCYC)TRESYN-PWY:trehalose biosynthesis I(0)<br>(GO) 46351 :disaccharide biosynthetic process 2,1130E-2<br>(GO) 5992 :trehalose biosynthetic process 1,4179E-2			**YiaG**<br>**YjdC**<br>BolA(CLR)<br>GadE(CLR)<br>MlrA(CLR)<br>YgiV(CLR)	GadX (0,000035018)
76	8	18	N/A			RpiR (LeMoNe)	PaaX (0,0001970799)

77	18	20	(GO) 34470 :ncRNA processing<br>1,2671E-4<br>(GO) 6394 :RNA processing<br>1,7468E-4<br>(GO) 16070 :RNA metabolic process<br>1,5159E-3			**GreA**<br>YrbA (LeMoNe)<br>HepA (LeMoNe)<br>Fis(CLR)	N/A
78	43	23	N/A	c0299 (LeMoNe)<br>sroD (CLR)		ArsR (LeMoNe)	BglJ (0,0005758219)<br>LexA (0,0001910775)<br>StpA (0,0005758219)

Table Characteristics of the module network, as reconstructed with CLR and LeMoNe

[a]Module ID: ID of modules

[b]Number of genes: number of genes in module

[c]Number of conditions: number of conditions in module

[d]Functional overrepresentation (Ecocyc and GO): Functionally overrepresented Ecocyc and GO classes in the respective modules. Only categories with a p-value<0.05 are shown, p-values for enrichment are indicated.

[e]Assigned sRNAs (sRNAs that were assigned to each of the modules by either CLR or LeMoNe; in bold are indicated the ones that were assigned by both CLR and LeMoNe)

[f]sRNAs for which predicted/known targets are present in the modules: sRNAs for which at least one predicted target was found to be present in the respective modules. Indicated in bold are the sRNAs that were assigned by both CLR and LeMoNe to the respective modules. Targets of indicated sRNAs are indicated between brackets; [u]: indicates that the sRNA target was predicted based on the sequence-based sRNA-target analysis; [l] : indicates that the indicated sRNA target was described in literature (and present in the benchmark set); [lu] indicates that the given sRNA target was both described in literature and predicted based on our sequence-based sRNA-target analysis.

[g]Assigned TFs (TFs that were assigned to each of the modules by either CLR or LeMoNe; in bold are indicated the ones that are assigned by both CLR and LeMoNe).

[h]Enriched TFs: TFs for which targets (according to RegulonDB) were found be enriched in the respective modules. p-values for enrichment are indicated between brackets. Indicated in bold are the TFs that were assigned by both CLR and LeMoNe.Indicated with hash mark ([#])are the TFs that were assigned either by CLR or by LeMoNe.
