# Supplementary material for: Inferring the relation between transcriptional and posttranscriptional regulation from expression compendia
Source: BMC Microbiol. 2014 Jan 27;14:14. doi: 10.1186/1471-2180-14-14 (PMC3948049; doi:10.1186/1471-2180-14-14)
Supplement: Additional file 2: Table S2 — Overview of the sRNAs in the different modules. [file 1471-2180-14-14-S2.pdf]

Additional file 2 - Supplementary Table 2: overview of the sRNAs in the different modules

[a]module ID	[b]sRNA	[c]sRNAtarget
3	Spf	*nanT*
3	OmrA	*fecA*
3	OmrB	*fecA*
3	OmrB	*gntP*
6	OmrA	*fepA*
6	OmrB	*fepA*
6	RybB	*fiu*
6	RyhB	*shiA*
6	OmrA	*cirA*
6	OmrB	*cirA*
6	OmrA	*fecA*
6	OmrB	*fecA*
8	RyhB	*fur*
8	RybB	*fumC*
9	RyhA	*tpx*
10	RybB	*ydeN*
10	Spf	*xylF*
12	GcvB	*brnQ*
14	GcvB	*sstT*
15	Spf	*nanC*
17	RybB	*nmpC*
17	MicF	*ompF*
17	RybB	*ompF*
17	RybB	*lamB*
17	SraD	*lamB*
19	GcvB	*livK*
21	CyaR	*yqaE*
22	Spf	*gltA*
22	SgrS	*manX*
22	Rybb	*rbsB*
23	RybB	*rraB*
24	RybB	*asr*
28	SroB	*ybfM*
28	DsrA	*hns*
28	OxyS	*yobF*
29	CyaR	*yqaE*
29	GcvB	*dppA*
30	GcvB	*ybdH*
31	Spf	*glpF*
33	GcvB	*ybdH*
36	Spf	*nanT*
36	Spf	*glpF*
36	Spf	*nanC*
40	Spf	*srlA*
40	Spf	*nanT*
40	Spf	*nanC*

41	MicF	*phoE*
45	OxyS	*ybaY*
50	SraD	*fimB*
51	RybB	*asr*
56	OmrA	*fepA*
56	OmrB	*fepA*
57	MicF	*phoE*
57	GcvB	*ybdH*
57	GcvB	*livK*
60	RybB	*nmpC*
60	RybB	*fadL*
62	RybB	*ompW*
62	SraD	*ompW*
65	RybB	*ompA*
65	SraD	*ompA*
65	RyhB	*cysE*
65	GcvB	*ilvC*
67	MicF	*phoE*
67	GcvB	*livK*
67	GcvB	*livJ*
68	Spf	*srlA*
68	RybB	*fimA*
69	RybB	*ompW*
69	SraD	*ompW*
69	RyhB	*frdA*
75	OxyS	*ybaY*
75	DsrA	*rpoS*
75	OxyS	*rpoS*
75	RprA	*rpoS*
75	RyhA	*rpoS*

Supplementary file 2:
[a]moduleID: ID of ISA modules, which have at least one known sRNA target
[b]sRNA: small RNA for which the target was found in the module.
[c]sRNAtarget: the corresponding targets of each indicated sRNA in the respective modules.
Indicated in blue are the modules containing multiple targets of the same sRNA.
