# Supplementary material for: Inferring the relation between transcriptional and posttranscriptional regulation from expression compendia
Source: BMC Microbiol. 2014 Jan 27;14:14. doi: 10.1186/1471-2180-14-14 (PMC3948049; doi:10.1186/1471-2180-14-14)
Supplement: Additional file 3: Table S3 — Performance of inferring sRNA-target interactions. [file 1471-2180-14-14-S3.pdf]

Additional file 3 - Supplementary Table 3: Performance of inferring sRNA-target interactions

[a] small RNA	[b] # of targets in benchmark dataset	[c] # of targets predicted by CLR (Modi et al.)	[d] # of predicted targets identified by CLR and sequence (Modi er al.).	[e] # of known targets identified (Modi et al)	[f] average size of modules	[g] # of predicted sRNA targets in modules	[h] # of known targets identified in this study
GcvB[hfq]	22 (8)	126	2	2 (0)	0	0	0 (0)
MicF[hfq]	4 (1)	22	3	1 (1)	18 **(3)**	3	1 (1)
RyhB[hfq]	8 (7)	19	1	1 (1)	35 **(10)**	5	1(1)
ArcZ[hfq]	2 (1)	2	0	0 (0)	0	0	0 (0)
C0067	0/	-	-	-	52 **(3)**	1	-
C0299	0/	-	-	-	61 **(6)**	0	-
C0343	0/	-	-	-	47 **(4)**	2	-
C0614	0/	-	-	-	22 **(1)**	0	-
C0664	0/	-	-	-	55 **(2)**	0	-
C0719	0/	-	-	-	18 **(1)**	0	0 (0)
CyaR[hfq]	4 (4)	19	0	0 (0)	0	0	0 (0)
DicF[hfq]	1 (1)	38	0	0 (0)	53 **(1)**	0	0 (0)
DsrA[hfq]	2 (2)	20	0	0 (0)	0	0	0 (0)
GadY[hfq]	1 (1)	21	0	1 (1)	53 **(2)**	5	0 (0)
GlmY	1/	-		-	0	0	0 (0)
GlmZ[hfq]	1 (1)	27	0	0 (0)	0	0	0 (0)
IS128	0/	-	-	-	48 **(3)**	0	0 (0)
IsrA[hfq]	0 (0)	103	0	0 (0)	5 **(1)**	0	(-)
IsrB	0/	-	-	-	39 **(5)**	1	0 (0)
IsrC	0/	-	-	-	64 **(1)**	0	0 (0)
Istr2	2/	-	-	-	0	0	0 (0)
MicA[hfq]	9 (1)	4	0	0 (0)	0	0	0 (0)
MicC[hfq]	1 (1)	6	0	0 (0)	0	0	0 (0)
MicM[hfq]	2 (2)	13	0	0 (0)	0	0	0 (0)
OmrA[hfq]	6 (4)	7	0	0 (0)	0	0	0 (0)
OmrB[hfq]	6 (4)	2	0	0 (0)	0	0	0 (0)

OxyS[hfq]	4 (2)	8	0	0 (0)	8 **(1)**	3	0 (0)
PsrD	0/	-	-	-	43 **(5)**	1	0 (0)
RdlA	1/	-	-	-	64 **(1)**	0	0 (0)
RdlB	1/	-	-	-	0	0	0 (0)
RdlC[hfq]	1/	-	-	0 (0)	0	0	0 (0)
RdlD	1/	-	-	-	59 **(1)**	0	0 (0)
RprA[hfq]	3(1)	8	0	0 (0)	0	0	0 (0)
RseX[hfq]	0 (2)	25	-	0	-	-	(-)
RttR	0/	-	-	-	48 **(1)**	0	0 (0)
RybA[hfq]	0 (0)	10	0	0 (0)	32 **(1)**	0	(-)
RybB[hfq]	21 (2)	0	0	0 (0)	0	0	0 (0)
RydB	0/	-	-	-	13 **(1)**	1	0 (0)
RydC[hfq]	1 (0)	1	0	0 (0)	48 **(2)**	0	(-)
RyeA[hfq]	0/	-	-	-	18 **(1)**	1	0 (0)
RyeB[hfq]	0 (0)	0	0	0 (0)	0	0	0 (0)
RyfA	0/	-	-	-	33 **(4)**	1	0 (0)
RyjA	0/	-	-	-	29 **(2)**	0	0 (0)
SgrS[hfq]	3 (1)	7	0	0 (0)	0	0	0 (0)
SokB	2/	-	-	0	0	0	0 (0)
SokC	2/	-	-	-	0	0	0 (0)
Spf[hfq]	9 (1)	22	0	0 (0)	48 **(2)**	2	1(1)
SraA	0/	-	-	-	52 **(1)**	0	0 (0)
SroA	0/	-	-	-	56 **(2)**	1	0 (0)
SroD	0/	-	-	-	52 **(5)**	2	0 (0)
SroH	0/	-	-	-	74 **(1)**	0	0 (0)
SymR	1/	-	-	-	0	0	0 (0)
Tp2	0/	-	-	-	48 **(1)**	0	0 (0)
Tpke11	0/	-	-	-	53 **(1)**	0	0 (0)
Tpke70	0/	-	-	-	37 **(2)**	3	0 (0)

[a]small RNA:
sRNAs for which probes were available in the microarray dataset are shown. [hfq] marked indicates this sRNA was shown to bind to HFQ.

[b]Number of known targets of the indicated sRNA in our benchmark set. For comparison the number of known targets according to the benchmark set used in Modi et al. 2011 is indicated in brackets. '/' indicates that Modi et al. 2011 did not have a benchmark for this sRNA.

[c]The total number of predicted targets for this particular sRNA by Modi et al. 2011 (using CLR).

[d]Number of predicted targets for the indicated sRNA identified by Modi et al. 2011 by overlaying CLR predictions with sequence-based predictions obtained with TargetRNA. Sequence-based predictions were performed as defined by [1] (see Materials and Methods)

[e]Number of benchmark targets (using our updated benchmark) for the indicated sRNA identified by Modi et al. 2011: by either using CLR-based assignments. Between brackets the number of benchmark targets obtained by combining the CLR-based assignments with sequence based prediction. A'-' indicates that no assignments were made for this particular sRNA.

[f] the average size of the modules to which the indicated sRNA was assigned in this work (based on assignments with either CLR or LeMoNe). Between brackets is indicated to how many modules the indicated sRNAs were assigned.

[g]: number of predicted sRNA targets of the indicated sRNA in the modules to which the indicated sRNA was assigned (by either CLR or LeMoNe (union)). Targets were identified by overlaying the module-based prediction (also union) with the sequence based predictions (IntaRNA and TargetRNA (see Materials and Methods)).

[h]Number of benchmark targets (using our updated benchmark) for the indicated sRNA recovered by our analysis: without brackets: number of benchmark sRNA targets present in the modules to which the respective sRNA was assigned (by either CLR or LeMoNe (union). A'-' indicates that no assignments were made for this particular sRNA. Between brackets: Number of benchmark sRNA targets for the respective sRNAs recovered in our modules that also contained a sequence recognition site as predicted by the sequence based assignment.

Table reads as follows: For MicF 22 targets were predicted by Modi et al. 2011 using CLR one of which corresponded to a benchmark target. Of those 22, only 3 had a binding site of MicF using sequence-based predictions and one of these 3 corresponded to a benchmark target. According to our analysis we assigned MicF to 3 modules with an average size of 18 genes. These modules contained 1 previously known MicF target. All modules together were predicted to contain 1 target of MicF based on our sequence-based predictions. This also corresponded to the benchmark target.

References

1. Modi SR, Camacho DM, Kohanski MA, Walker GC, Collins JJ: **Functional characterization of bacterial sRNAs using a network biology approach**. *Proc Natl Acad Sci U S A* 2011, **108**(37):15522-15527.
