# Supplementary material for: Inferring the relation between transcriptional and posttranscriptional regulation from expression compendia
Source: BMC Microbiol. 2014 Jan 27;14:14. doi: 10.1186/1471-2180-14-14 (PMC3948049; doi:10.1186/1471-2180-14-14)
Supplement: Additional file 4 — Module overview. [file 1471-2180-14-14-S4.pdf]

Additional file 4 - Module overview
Only modules to which sRNAs were assigned are shown:

Module 17

Assignment of the regulatory program to module 17

[a]Module id	[b]Regulator id	[c]Algorithm	[d]CLR score	[e]LeMoNe score	[f]Regulator
17	MalT	clr	4,28	334,52	TF
17	IsrB	lemone	1,57	193,76	sRNA
17	YgiV	clrlemone	3,08	159,19	TF
17	CueR	clrlemone	3,52	153,84	TF
17	MicF	clr	3,02	1,28	sRNA

[a]Module id: id of cluster to which at least one regulator was assigned

[b]Regulator id: regulators assigned with CLR and LeMoNe

[c]Algorithm: indicates by which algorithm the regulator was assigned: “clr” if regulator was assigned by CLR, “lemone” if regulator was assigned by LeMoNe, “clrlemone” if regulator was assigned by both algorithms.

[d]CLR score: CLR score, the score assigned to the respective regulator by CLR. A CLR score is considered relevant if above 3 (see Materials and Methods)

[e]LeMoNe score: The score assigned to the respective regulator by LeMoNe. A LeMoNe score is considered relevant if above 100 (see Materials and Methods)

[f]Regulator: the type of the assigned regulator: “TF”: transcription factor, “sRNA”: sRNA

Module content:

(31 genes and 11 conditions)

nmpC	outer membrane porin protein; locus of qsr prophage (NmpC)
borD	bacteriophage lambda Bor protein homolog (BorD)
ompF	OmpF is a member of the General Bacterial Porin (GBP) family.
yccA	putative carrier/transport protein; substrate or modulator of FtsH-mediated proteolysis (YccA)
ycfS	YcfS is an L,D-transpeptidase responsible removal of the D-alanine residue of peptidoglycan tetrapeptide stems and attachment of the lysine residue of Braun

	lipoprotein to the meso-diaminopimelyl (DAP) residue of the resulting tripeptide.
chaA	ChaA sodium/proton transporter
ygaH	YgaH L-valine exporter
yghG	putative lipoprotein (YghG)
pppA	encodes a peptidase that shows activity toward exogenous pilin substrates
yghJ	YghJ is a hypothetical protein. Sequence similarity suggests that it is a member of the Autotransporter (AT) Family.
ygiC	Deletion of ybiBC exacerbates the growth defects observed when *E.coli* tolC mutants are grown in glucose minimal media.
alx	Alx is a predicted to be a membrane-bound redox modulator
malQ	Amylomaltase was first described as a maltose-inducible enzyme that catalyzes a reversible transglycosidase reaction
malP	The malP gene product, maltodextrin phosphorylase, is one of two distinct α-glucan phosphorylases in *E. coli*.
tnaC	tryptophanase leader peptide
tnaA	The tnaCAB operon codes for two key enzymes in tryptophan metabolism, including trytophanase.
rdoA	rdoA is a serine/threonine protein kinase that is able to autophosphorylate at serine and threonine, but not tyrosine residues.
malK	MalK is the ATP-binding component of the maltose ABC transporter.
lamB	LamB is a member of the Sugar Porin (SP) family. It specifically facilitates the diffusion of maltose and other maltodextrins (α1-4 linked polyglucosyls) across the outer membrane.
malM	malM is the last gene in the malK-lamB-malM operon and is thus part of the maltose regulon.
miaA	Dimethylallyl diphosphate:tRNA dimethylallyltransferase (DMAPP-tRNA transferase, MiaA)
hflX	The HflX protein is a GTPase that interacts with the 50S subunit of the ribosome in the presence of both GTP, GDP, ATP and ADP.
hflK	HflK is an inner membrane protein which forms part of the HflCK complex that interacts with and regulates, the ATP-dependent protease FtsH .
treC	*E. coli* can utilize trehalose as the sole source of carbon.
treB	Component of: trehalose PTS permease
fecE	ATP binding component of the iron dicitrate ABC transporter
fecC	Component of: iron dicitrate ABC transporter
fecB	Component of: iron dicitrate ABC transporter
yjiA	The protein folds into two distinct domains; the structure of the N-terminal domain suggests the presence of a conserved NTP binding site with specificity for guanine, which was experimentally verified. A GTP-dependent regulatory function is proposed for YjiA
yjiX	No information about this protein was found by a literature search conducted on January 15, 2012.
yjiY	stress response protein [Singh09]

Regulators assigned:

MicF regulator was assigned by CLR.

IsrB regulator was assigned by LeMoNe.

MalT, YgiV, CueR regulators were assigned by CLR and LeMoNe.

GO Enrichment:

- Ion transport. 37% of gene content can be described with this GO term. (chaA fecE lamB nmpC fecB fecC ompF)
- carbohydrate transport 21% of gene content can be described with this GO term. (treB malM malK lamB)
- cation transport 21,0% of gene content can be described with this GO term. (chaA fecE fecB fecC)

Ecocyc Pathway Enrichment:

- L-cysteine degradation II: tnaA is involved in this pathway and this pathway is overpresented with *p*-value 0.00097931
- tryptophan degradation II (via pyruvate):tnaA is involved in this pathway and this pathway is overpresented with *p*-value 0(according the database of Ecocyc, there is only one gene in this pathway also enriched with GO terms)
- glycogen degradation: malQ and malP are involved in this pathway and this pathway is overpresented with *p*-value 0.0009362
- trehalose degradation I (low osmolarity): treB and treC are involved in this pathway and this pathway is overpresented with *p*-value 0.00054676

Module Description:

Module 17 contains 31 genes most of which relate to membrane encoded transport systems. The module was predicted to be regulated by the TFs MalT of which also the targets were found to be enriched in this module, and CueR and YgiV, both without obvious targets in the module. For the small RNA MicF assigned to this module, one of its known targets, *ompF*, belongs to the module [1]. Although the module does not contain any other predicted targets of MicF, we found evidence of a MicF recognition site in the TF YgiV also assigned to the module (located in the region [-102, -52 bp] upstream of the start codon of YgiV, a region that comprises the short intergenic region between YgiW and YgiV, and the C terminal end of the coding region of YgiW. The region on MicF covered by YgiV does not overlap with regions covered by known MicF targets. YgiV is known as a repressor of McbR (also known as YncC), a regulator of biofilm formation [2]. Also interesting is the assignment of the sRNA IsrB with unknown function to the same module. IsrB has no documented targets yet, but its genomic location overlaps with the coding regions of *azu*-genes, a set of innermembrane encoding genes with unknown function. A link between IsrB and membrane encoded functions is plausible viewing the large subset of membrane related functionalities in this module. However, relying on our sequence-based sRNA target prediction, no direct target of IsrB was found to be present in this module so IsrB could be involved in the indirect regulation of this module (e.g by regulating other regulators that on their turn regulate the genes in the module).

Module Figure

Panel A

The regulatory program assigned to this module. TFs are indicated by squares, sRNAs by triangles. Panel B: The module to which the regulatory program was assigned; yellow indicates high expression levels and blue refers to low expression levels of genes in the module. The genes

correspond to the genes present in the original module discovered by ISA. Conditions present in the original ISA module are indicated by a horizontal bar. As both LeMoNe and CLR use all conditions when assigning their respective regulatory program, we indicated also the additional compendium conditions that were relevant for assigning the respective regulatory programs. Genes in the module correspond to likely targets of the assigned regulators. Targets indicated by a square correspond to known targets of the assigned TF(s). Targets indicated by empty triangle correspond to predicted targets of the assigned sRNA, targets indicated by filled triangle correspond to known targets of the assigned sRNA.

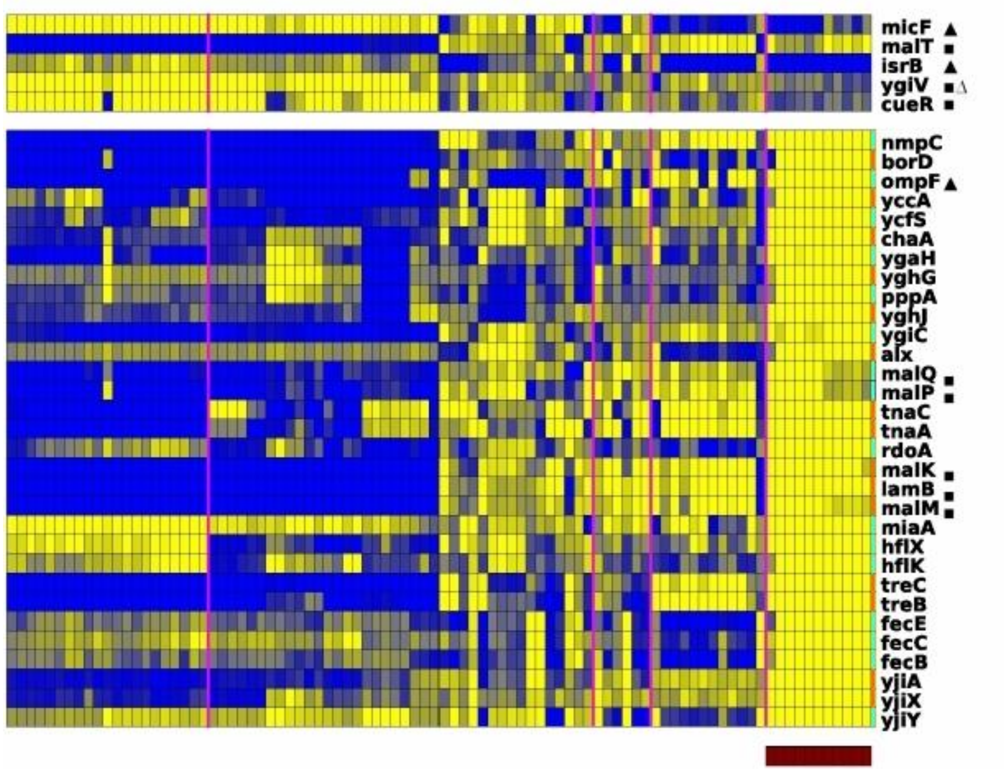

Panel B

sRNA-target interaction as predicted by the sequence-based analysis for both known and predicted targets of the sRNAs assigned to the module. Indicated sequence positions refer to the location of the recognition sequence relative to the translation start of the gene the sRNA is

predicted to interact with (if ATG is indicated in red). If the ATG is indicated in black it refers to the start codon of a neighbouring gene.

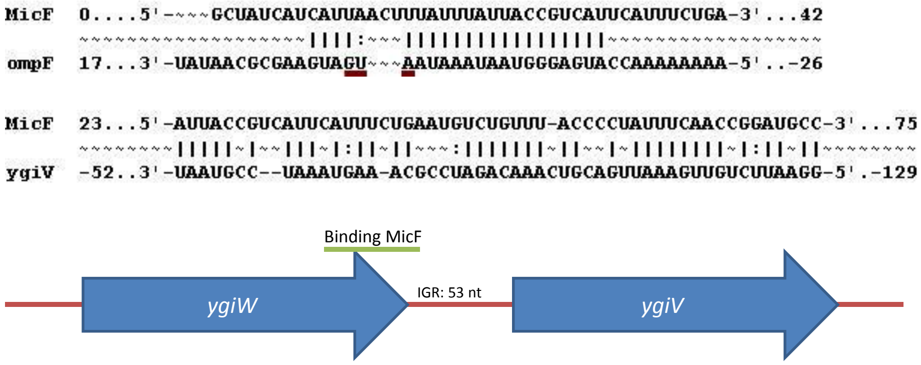

Panel C

Indication of the putative interaction region between MicF and its predicted target YgiV on the sequence of MicF. As a reference also the regions bound by known targets of MicF and information on the 2D conformation of the sRNA are indicated.

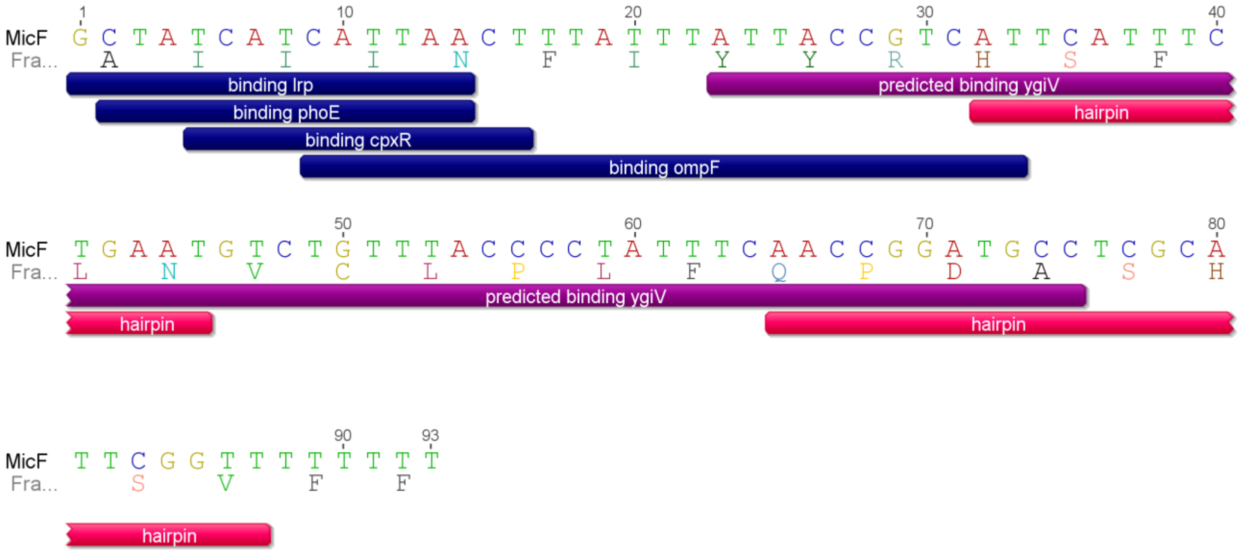

Module 58

Assignment of the regulatory program to module 58

[a]Module id	[b]Regulator id	[c]Algorithm	[d]CLR score	[e]LeMoNe score	[f]Regulator
58	CueR	clr	4,63	37,42	TF
58	GadE	clrlemone	4,86	828,24	TF
58	GadY	clr	3,08	1,45	sRNA

[a]Module id: id of cluster to which at least one regulator was assigned

[b]Regulator id: regulators assigned with CLR and LeMoNe

[c]Algorithm: indicates by which algorithm the regulator was assigned: "clr" if regulator was assigned by CLR, "lemone" if regulator was assigned by LeMoNe, "clrlemone" if regulator was assigned by both algorithms.

[d]CLR score: CLR score, the score assigned to the respective regulator by CLR. A CLR score is considered relevant if above 3 (see Materials and Methods)

[e]LeMoNe score: The score assigned to the respective regulator by LeMoNe. A LeMoNe score is considered relevant if above100 (see Materials and Methods)

[f]Regulator: the type of the assigned regulator: "TF": transcription factor, "sRNA": sRNA

Module content:

(56 genes and 20 conditions)

yaeR	No information about this protein was found by a literature search conducted on January 15, 2012.
ybaS	YbaS is a glutaminase that is highly selective for L-glutamine.
ybaT	YbaT is a putative amino acid/amine transporter [Riley06] of the APC superfamily.
cueR	See gene description in .doc module file.
poxB	See gene description in .doc module file.
hyaA	Component of: hydrogenase 1, small subunit
hyaB	hydrogenase 1, large subunit
hyaC	hydrogenase 1, b-type cytochrome subunit
hyaD	protein involved in processing of HyaA and HyaB proteins
hyaE	protein involved in quality control of HyaA (HyaE)

hyaF	The HyaF protein appeared to be required for the synthesis of active hydrogenase isoenzyme 1 (HYD1)
appC	cytochrome bd-II terminal oxidase subunit I (AppC)
appB	cytochrome bd-II terminal oxidase (AppCB)
appA	Acid phosphatase catalyzes the hydrolysis of the distal phosphoryl residues of GTP and of the regulatory nucleotide guanosine 5',3'-bisphosphate under very acidic conditions
cbpM	Purified CbpM protein specifically inhibits both the DNA binding and co-chaperone activity of CbpA.
cbpA	CbpA has similarity to DnaJ and functions as a co-chaperone with DnaK in vitro
yccJ	The sublethal pressure treatment down-regulated this gene significantly:
hlyE	Hemolysin E causes lysis of mammalian cells.
gadC	GadC, a glutamic acid:γ-aminobutyrate antiporter, is part of the glutamate-depenent acid resistance system 2 (AR2) which confers resistance to extreme acid conditions.
gadB	GadB, a glutamate decarboxylase enzyme, is part of the glutamate-dependent acid resistance system 2 (AR2) which confers resistance to extreme acid conditions.
sufS	SufS exhibits activity with respect to assembly of the ferredoxin iron-sulfur cluster in an in vitro assay.
osmE	OsmE is an osmotically inducible gene product in *Escherichia coli*. OsmE has a lipoprotein-type signal sequence at the amino terminus.
yegP	No information about this protein was found by a literature search conducted on January 15, 2012.
xdhA	An xdhA mutant exhibits a defect in an indirect assay of xanthine dehydrogenase activity and exhibits sensitivity to adenine, which is indicative of a defect in purine salvage. Component of: xanthine dehydrogenase.
xdhB	Component of: xanthine dehydrogenase.
xdhC	Component of: xanthine dehydrogenase
ygeW	YgeW was suggested to function as an oxamate transcarbamoylase in a predicted purine catabolic pathway
ygeX	The ygeX gene encodes 2,3-diaminopropionate ammonia-lyase. 2,3-diaminopropionate + H_2O <=> 2 ammonia + pyruvate + H^+
ygeY	No information about this protein was found by a literature search conducted on January 15, 2012.
hyuA	Subunit composition of phenylhydantoinase = [HyuA]4
yqeA	multidrug resistance (indirect information)
yqeB	No information about this protein was found by a literature search conducted on January 15, 2012.
ygfK	Component of: putative selenate reductase
ssnA	predicted chlorohydrolase/aminohydrolase (SsnA)
ygfM	Component of: putative selenate reductase
xdhD	Component of: putative selenate reductase
ygfO	XanQ xanthine NCS2 transporter (YgfO)
guaD	guanine deaminase. Guanine deaminase is an aminohydrolase that converts guanine to xanthine and ammonia. Guanine deaminase is an aminohydrolase that converts guanine to xanthine and ammonia. This reaction removes guanine from the pool of

	guanine-containing metabolites, which helps to regulate cellular GTP and the guanylate nucleotide pool. Ammeline is an intermediate in the metabolism of melamine. *E. coli* is able to metabolize ammeline to ammelide, but does not metabolize either melamine or ammelide. A guaD deletion mutant is deficient in ammeline deaminase activity.
ygfQ	YgfQ has been predicted to be a xanthine, uracil permease by bioinformatic analysis
ygfS	predicted oxidoreductase, 4Fe-4S ferredoxin-type subunit (YgfS)
ygfT	fused predicted oxidoreductase, Fe-S subunit and nucleotide-binding subunit (YgfT)
ygfU	YgfU is predicted to be a xanthine, uracil permease and to have a σ-54 promoter.
ygiV	based on nickel enrichment DNA microarrays and additional gel shift assays, it was identified as a transcriptional repressor of mcbR, which regulates biofilm formation and mucoidity by repressing expression of mcbA (ybiM).
yqjE	YqjE is an inner membrane protein with two predicted transmembrane domains.
yqjK	No information about this protein was found by a literature search conducted on January 15, 2012.
fic	filamentation induced by cAMP
yhiM	YhiM is an inner membrane protein with ten predicted membrane spanning domains; its C terminus is located in the cytoplasm
slp	Slp (starvation lipoprotein) is the product of the slp gene which forms an operon with the downstream gene dctR. Slp is believed to take part in acid resistance as expression increased when cells were grown at pH 5.5 and 4.5 under conditions known to induce glutamate-dependent acid resistance compared to pH 7.4 under the same conditions.
hdeD	acid-resistance membrane protein (HdeD)
mdtE	MdtE is a component of the MdtEF multidrug transporter.
mdtF	MdtF is a component of the MdtEF multidrug transporter.
gadW	The transcription factor GadW, for "Glutamic acid decarboxylase," is negatively autoregulated and controls the transcription of the genes involved in the principal acid resistance system, is glutamate dependent (GAD), and is also referred to as the GAD system
gadA	M
yjbR	The yjbR gene expression could be induced in a direct or indirect way by the OmpR protein.
aidB	isovaleryl-CoA dehydrogenase (AidB)

Regulators assigned:

CueR, GadY regulators were assigned by CLR.

GadE regulators were assigned by CLR and LeMoNe.

GO Enrichment:

- Carboxylic acid metabolic process. 25% of gene content can be described with this GO term. (yqeA sufS ygeW ygeX gadB ybaS gadA ldcC ygfT)
- Oxidation reduction. 33% of gene content can be described with this GO term. (appC poxB appB xdhA aidB xdhB ygfS hyaC hyaB xdhD hyaA ygfT)
- Nucleobase, nucleoside and nucleotide metabolic process. 16% of gene content can be described with this GO term. (ygeW xdhA xdhC xdhB xdhD hyuA)

Ecocyc Pathway Enrichment:

- Salvage pathways of adenine, hypoxanthine, and their nucleosides: xdhC,xdhB and xdhA are involved in this pathway and this pathway is overpresented with *p*-value 0.00099396.

Module Description:

Module 58 contains 56 genes and is mainly been expressed under stationary growth and contains pathways involved in acid response, amino acid starvation (purine salvage, amino acid uptake) and induction of microaerobiosis (represented by Ecocyc pathway enrichment analysis). 3 regulators were assigned to Module 58, GadE, CueR and the sRNA GadY, all of which are known targets of RpoS [3]. Module 58 was also found to be enriched in direct targets of GadE (see **Additional file 1)**, indicating that the assignment of GadE as a regulator to module 58 is true. GadE, the central activator of the acid response system controls genes involved in the maintenance of pH homeostasis through its direct targets involved in the glutamate-dependent acid resistance system (here represented by *gadA* and *gadBC* genes) and is involved in multidrug

efflux (*mdtE, mdtF*) through controlling the expression of the TFs, GadW and GadX both related to acid resistance (of which only GadW was found in the module) [4]).

The small RNA GadY which is highly expressed during entry into stationary phase and regulated by low pH [5] is related to the GadE dependent acid response through an intricate network of interactions with gadW (also in module 58) and GadX (according to Regulon DB) one of the TFs that is also a target of GadE, but that was not found in module 58. A last regulator assigned to module 58 was CueR "Cu efflux regulator", which was also predicted to be a target of GadY using our sequence-based predictions. CueR, regulates genes related to the primary copper homeostasis system in response to the presence of copper, silver, or gold ions [6]. None of the known CueR targets related to its function in Cu^{2+} homeostasis were found in module 58. However, CueR being a target of GadY and also being assigned as a regulator to module 58 points towards a connection between Cu and pH homeostasis, a link that has been suggested before. Yamamoto et al, for instance, showed that pH changes affect the genome-wide transcription pattern of copper-balance genes in the presence of $CuSO_4$ [7].

Besides CueR module 58 contained three additional predicted targets of GadY (assuming that genes belonging to a module with an assigned sRNA as regulator that also contain a recognition sequence of that sRNA in their upstream region are direct targets of the sRNA). A first one, CbpA has a functionality related to the one of DnaJ and functions as a co-chaperone with DnaK A second one, PoxB is pyruvate oxidase and the last one XdhA-XdhB-XdhC is a putative heterotrimericxanthine dehydrogenase [8]. How their functionalities link to the role of GadY is less clear. The fact that the regions to which GadY would bind in its predicted targets CueR, DnaJ and PoxB are located quite far upstream of their respective annotated TSSs could explain why such non-conventional targets have largely been overlooked by computational predictions.

.Module Figure: legend as above

Panel A

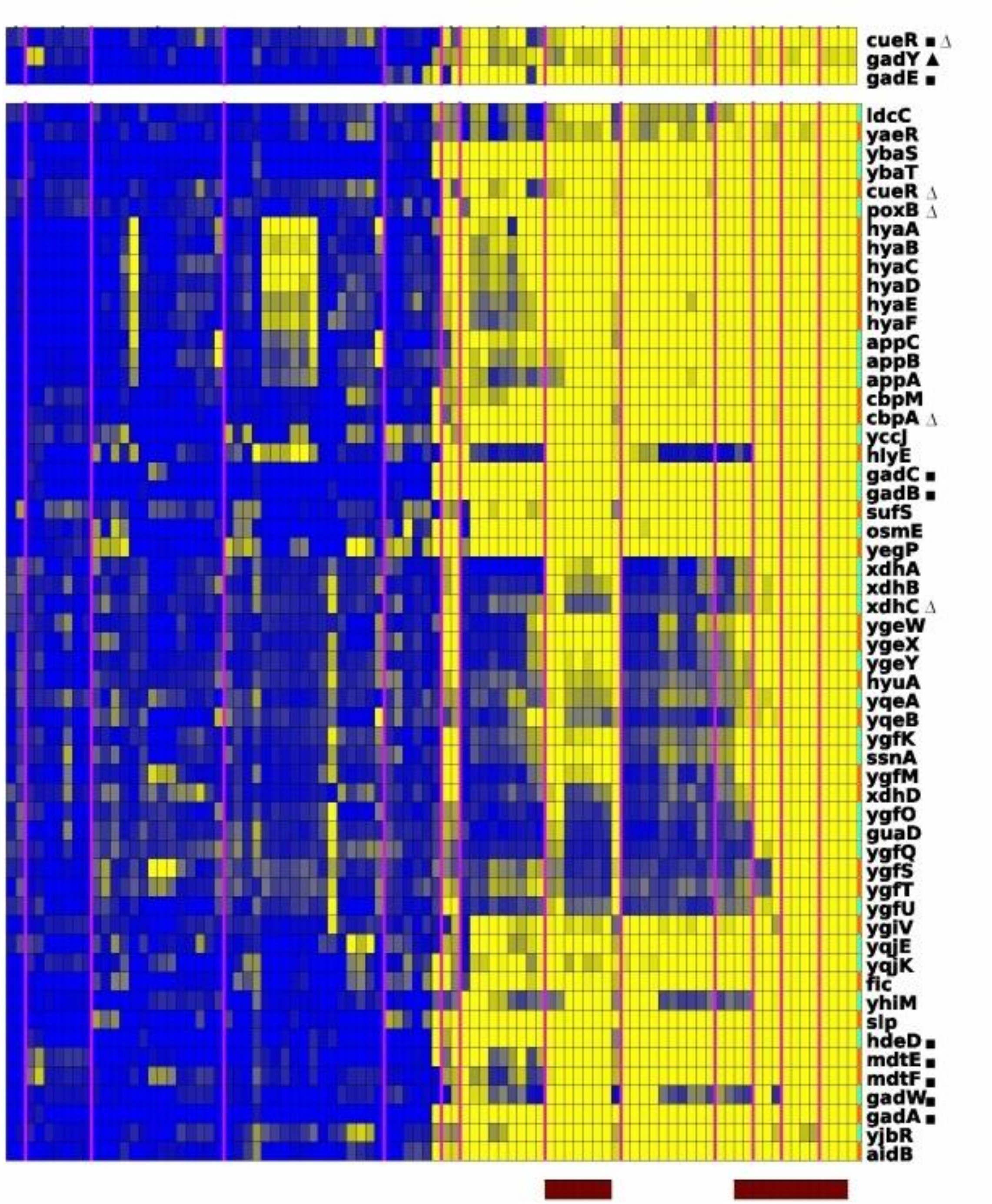

Panel B

```
GadY  18...5'-UCCCGUGCCAACAGGGAG~UGUUAUAACGGUUUAUUAGUCUGG-3'...42
              ~~~~~~~~~~~~~~~~~~~~~~||||||||~|||||||~||||||~~~~~~~~~~~~~~~~~~~~~~
cueR  -114.3'-UCAUGACGCGUGUCUCUCUACAAUAU~GCCAAAAUAACGCGAA-5'.-136
```

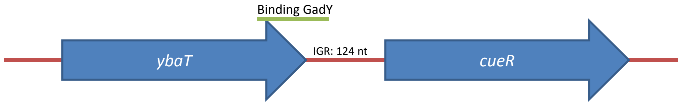


```
GadY  10...5'-ACAAAGUUUCCCGUGCCAACA-GGGA-GUGUUAUA-3'...42
              ~~~~~~~~~||||||:~|:|:~||||||||:~~||||:||||~~~~~~~~~
cbpA  -75..3'-AGUUUUAAAGUAUACGGUUGUGUCUUAUACAAUAA-5'.-109
```

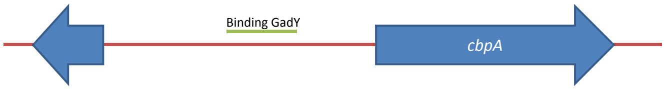


```
GadY  20...5'-CCGUGCCAACAGGGAGUGUUAUAACGGUUUAUUAGUCUGGAGACGGCAGACUAUCCUCUUCCCGGUCC-3'...87
              ~~~~~~~~~~~~~~~~~~|:||||~|:~~||||||||~|~|||:|:|:~||~:|~~||~||||~||~~~~~~~~~~~~~~~~~~
poxB  -65..3'-UACCCUCUCUUUCCUC~CGUGAUUGCCAAUUUAUCGGGCUACUUUC~~CUUAUAGUAGCCCGUAUUCC-5'.-129
```

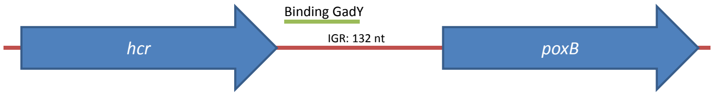


```
GadY  45...5'-GUGUUAUAACGGUUUAUUAGUCUGGAGACGGCAGACUAUCCUCUUCCC~GGUCC~CCUAUGCCGGGU-3'...90
              ~~~~~~~~~~~~~~~~~~||||~~~~~|||||||~||~||||||~|~|||||~~~|~|||||~||~~~~~~~~~~~~~~~~~~
xdhC  -47..3'-AGUGAAAAAACCAGUAGCAGACCUACUCUACGUCUGCUUUGAGAAAUGACCGGGCGGUACUUCUUGC-5'..-94
```

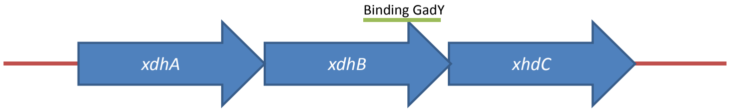

Panel C

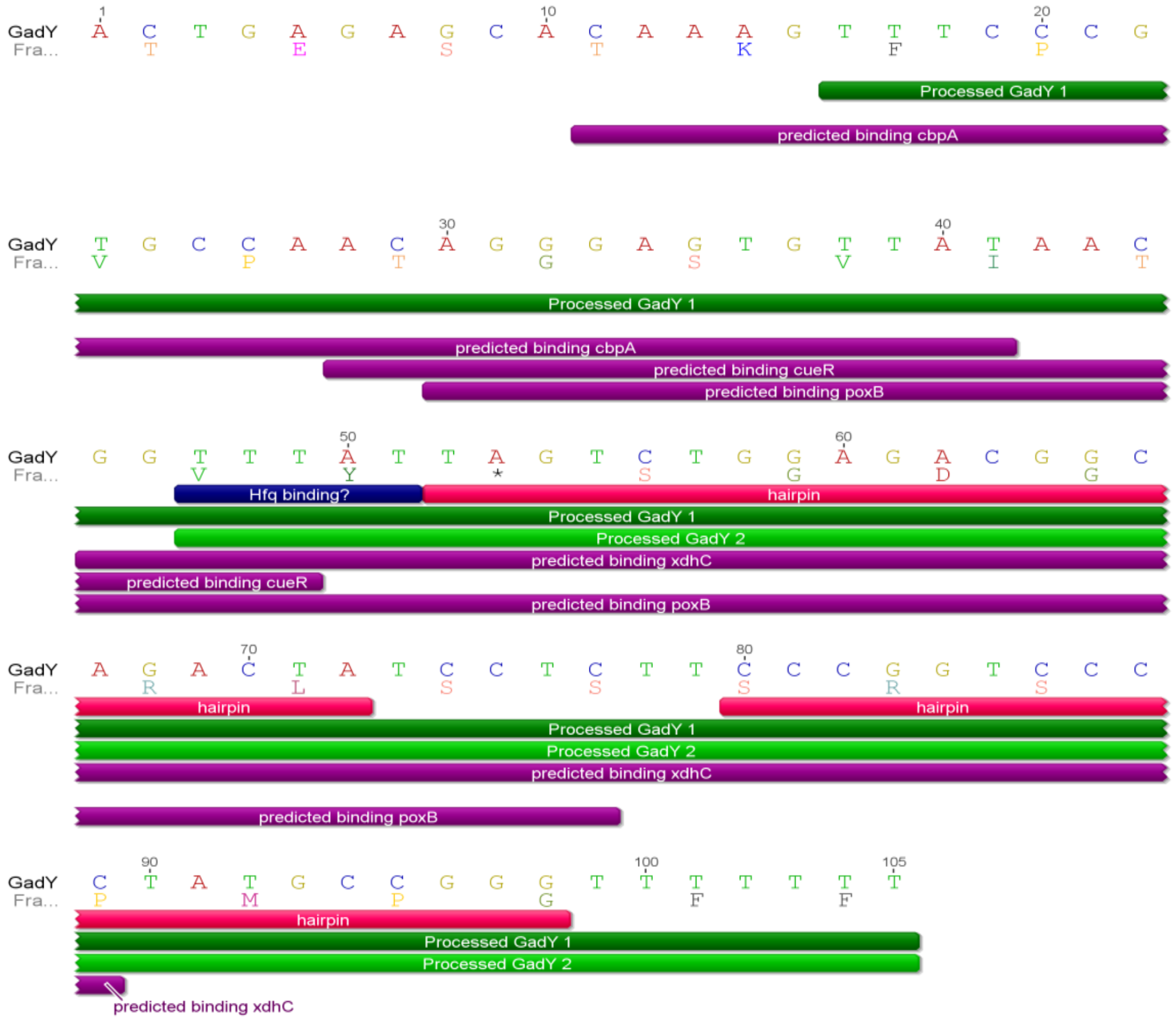

Legend: as above modules

Module 6

Assignment of the regulatory program to module 6

[a]Module id	[b]Regulator id	[c]Algorithm	[d]CLR score	[e]LeMoNe score	[f]Regulator
6	RyhB	clrlemone	3,32	434,5	sRNA
6	IscR	clrlemone	3,52	109,99	TF

[a]Module id: id of cluster to which at least one regulator was assigned

[b]Regulator id: regulators assigned with CLR and LeMoNe

[c]Algorithm: indicates by which algorithm the regulator was assigned: "clr" if regulator was assigned by CLR, "lemone" if regulator was assigned by LeMoNe, "clrlemone" if regulator was assigned by both algorithms.

[d]CLR score: CLR score, the score assigned to the respective regulator by CLR. A CLR score is considered relevant if above 3 (see Materials and Methods)

[e]LeMoNe score: The score assigned to the respective regulator by LeMoNe. A LeMoNe score is considered relevant if above 100 (see Materials and Methods)

[f]Regulator: the type of the assigned regulator: "TF": transcription factor, "sRNA": sRNA

Module content:
(53 genes and 17 conditions)

fhuA	outer membrane protein receptor for ferrichrome, colicin M, and phages T1, T5, and phi80
entD	phosphopantetheinyl transferase
fepA	outer membrane receptor for ferric enterobactin (enterochelin) and colicins B and D
fes	enterochelin esterase. Fes catalyzes hydrolysis of free enterobactin and ferric enterobactin
ybdZ	No information about this protein was found
entF	apo-serine activating enzyme, located in cytoplasm and inner membrane
fepC	Subunit of: ferric enterobactin ABC transporter
fepG	Subunit of: ferric enterobactin ABC transporter
fepD	Subunit of: ferric enterobactin ABC transporter
entS	isochorismate synthase 1, expression of entC is induced by low iron concentration both aerobically and anaerobically
fepB	Subunit of: ferric enterobactin ABC transporter

entC	isochorismate synthase 1
entE	EntE is an enzyme of the enterobactin biosynthesis pathway that catalyzes the ATP-dependent condensation of 2,3-dihydroxybenzoate (DHB) and holo-EntB to form the covalently arylated form of EntB.
entB	apo-EntB multimer
entA	2,3-Dihydro-2,3-dihydroxybenzoate dehydrogenase catalyzes the formation of 2,3-dihydroxybenzoate (DHB), an intermediate in the enterobactin biosynthesis pathway.
ybdB	thioesterase that is involved in the biosynthesis of enterobactin
ybiI	No information about this protein was found
ybiX	Expression of ybiX is induced in $Fe2^{+}$ chelator and fur mutant strains
fiu	putative outer membrane receptor for iron transport
efeU	ferrous iron permease component of the EfeUOB ferrous iron transporter
efeO	periplasmic protein component of the EfeUOB ferrous iron transporter.
efeB	heme-containing component of the cryptic EfeUOB ferrous iron transporter
fhuE	outer membrane receptor for ferric iron uptake
tonB	cytoplasmic membrane protein which transduces the proton motive force (pmf) of the cytoplasmic membrane to the outer membrane active transporters thus providing the energy source required for the import of iron-siderophore complexes and vitamin B12 across the outer membrane
yncE	hypothetical protein. YncE is de-repressed under iron restriction through the action of the global iron regulator Fur, suggesting a role in iron acquisition
pqqL	putative zinc peptidase
yddB	predicted porin protein, was found to be required for growth under optimum growth conditions (rich medium at 37℃), but not under cold conditions (15℃) or in minimal medium
yddA	YddA complex, to be required for growth in optimum (rich medium at 37 degrees C) growth conditions but not under cold conditions (15 degrees C) or in minimal medium.
sufE	sulfur acceptor that interacts with and increases the activity of the SufS cysteine desulfurase
sufS	selenocysteine lyase, exhibits activity with respect to assembly of the ferredoxin iron-sulfur cluster in an in vitro assay
sufD	component of the SufBC2D Fe-S cluster assembly scaffold complex
sufC	component of the SufBC2D Fe-S cluster assembly scaffold complex
sufB	component of the SufBC2D Fe-S cluster assembly scaffold complex
sufA	Fe-S transport protein in Fe-S cluster assembly
ydiE	conserved protein, belongs to the Fur regulon
btuD	ATP-binding component of the BtuCD, an ABC-type vitamin B12 uptake system
shiA	high affinity transport of shikimate, an intermediate in the aromatic amino acid biosynthetic pathway
cirA	Transporter: outer membrane receptor involved in uptake of ferric dihyroxybenzoylserine
mntH	member of the natural resistance-associated macrophage proteins (NRAMP) family of metal ion transporters. The protein has 11 putative membrane spanning alpha

	helices. This permease is involved in the uptake of Metal 2^+.
nrdH	electron donor for ribonucleotide reductase
nrdI	lavodoxin that mediates generation of the tyrosyl radical cofactor of NrdF, the β subunit of the class Ib ribonucleotide reductase. The nature of the metal ion cofactor for NrdF has been controversial, but recent results indicate that the MnIII2-Y ·form is the active form of NrdF and is generated from two HO2- anions supplied by NrdI
nrdE	ribonucleoside-diphosphate reductase 2, α subunit dimer
nrdF	ribonucleoside-diphosphate reductase 2, β subunit dimer
exbD	part of the TonB-dependent energy transduction system for the import of iron-siderophore complexes and vitamin B12 across the outer membrane.
exbB	part of the TonB-dependent energy transduction system for the import of iron-siderophore complexes and vitamin B12 across the outer membrane.
yqjH	putative tRNA synthetase,
bfd	bacterioferritin-associated ferredoxin, is thought to be involved in Bfr iron storage and iron release functions or in regulation of Bfr
fecA	outer membrane receptor; citrate-dependent iron transport, outer membrane receptor
fecR	regulator for fec operon, periplasmic
fecI	RNA polymerase sigma 19
bglJ	positive DNA-binding transcriptional regulator of transport and utilization of the aromatic β-glucosides arbutin and salicin
fhuF	acts in reduction of ferrioxamine B iron
yjjZ	No information about this protein was found

Regulators assigned:

RyhB regulator was assigned by CLR and LeMoNe.

IscR regulator was assigned by CLR and LeMoNe.

GO Enrichment:

- Iron-sulfur cluster assembly. 7% of gene content can be described with this GO term.(sufA sufB sufD)
- Di-, tri-valent inorganic cation transport. 50% (entE entF cirA entC fecI fhuE entD fiu fepB fecR fepA fepD fepC tonB entA fes entB fepG mntH fecA fhuA)
- Transition metal ion transport. 50% (entE entF cirA entC fecI fhuE entD fiu fepB fecR fepA fepD fepC tonB entA fes entB fepG mntH fecA fhuA)

Ecocyc Pathway Enrichment:

- Enterobactin biosynthesis: Module 6 fully covers this pathwya with genes entC,entA,entE,entF,entD and entB.(*p*-value : 0)

Module Description:

Module 6 is a rather large module being overrepresented for genes involved in iron transport and iron-sulfur cluster assembly. Two regulators, one TF (IscR) and one sRNA (RyhB) were assigned to this module with a high reliability (as their assignment was confirmed by both LeMoNe and CLR): IscR, a sulfur-cluster containing TF, belonging to a polycistronic mRNA *iscRSUA* is known to regulate the expression of operons that encode components of a pathway of iron-sulfur cluster assembly, iron-sulfur proteins, anaerobic respiration enzymes and biofilm formation [9, 10]. Module 6 contains two known targets being regulated by IscR (the operons *nrdHIEF* and *sufABCDES*, involved in iron-sulfur cluster assembly [11]).

Besides IscR, also the sRNA RyhB was assigned to this module. This assignment was confirmed by that fact that the module contained one known target of RyhB (ShiA) [12]. In addition to this known target we also have one predicted RyhB target in the module, SufB (Fig 5 panel B), which is a component of the $SufBC_2D$ Fe-S cluster assembly scaffold complex [13] that is responsible for the synthesis of Fe-S clusters [13-15].

Downregulation of proteins involved in assembly of Fe-S clusters by RyhB would make sense given the known function of RyhB during Fe homeostasis: RyhB is known to reduce iron consumption under low-iron conditions by downregulating expression of iron-containing proteins [16-18] and the Fur regulon, of which also several genes are present in the module and which is known to have a central role in iron metabolism). In addition the direct binding of RyhB to *sufB* seems likely as the interacting region identified in RyhB is located in an unstructured

region and overlaps with those of previously detected targets (see Supplementary_Table_1_Modulenetwork).

Interestingly, both the ISC assembly system (*iscRSUA* operon*)*, which is responsible for Fe-S cluster production under normal conditions and the SUF assembly system (*sufABCDSE* operon) responsible for Fe-S cluster production under oxidative stress conditions are encoded by polycistronic operons. The polycistronic *iscRSUA* mRNA to which also IscR the regulator assigned to the module belongs, is known to be processed by RyhB by inducing a cleavage of the operonic transcript between *iscR* and *iscSUA* [19]. Our predicted interaction between RyhB and *sufB* would suggest a similar regulation as the one observed for the RyhB – *iscRSUA* interaction. [19].

Module Figure: legend as above

Panel A

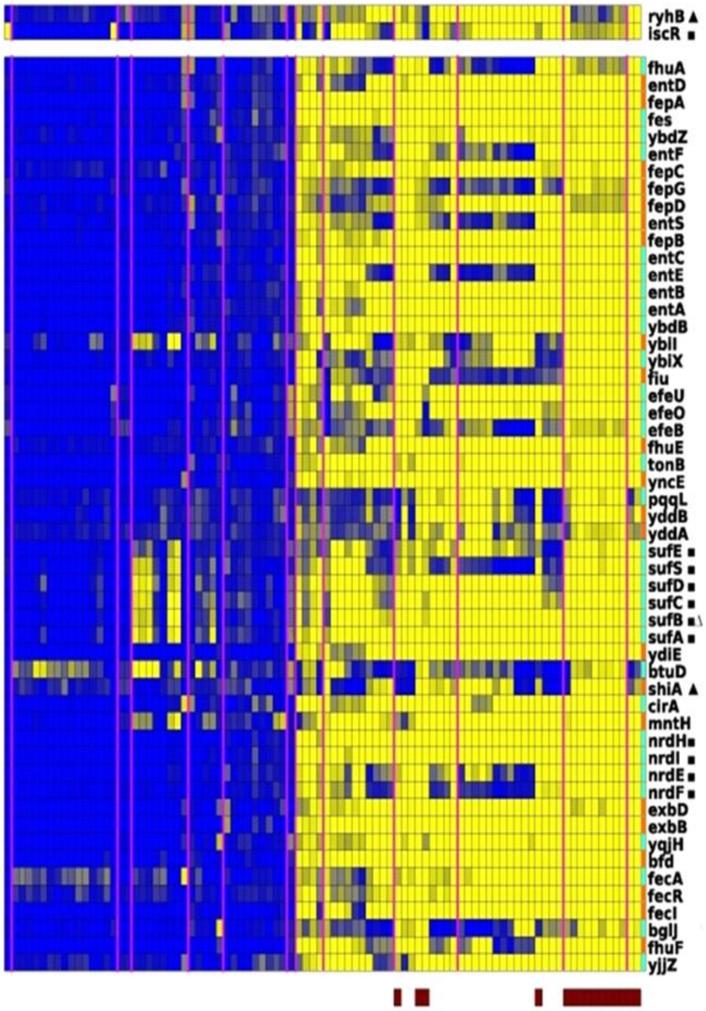

Panel B

```
RyhB   40...5'-AGCACGACAUUGCUCACAUUGCUUCCAGUAUUACUUAGCCAGCCGG-3'...65
              ~~~~~~~~~~~~~~~~~~|||||||~~~~~~~~~~~~~||:|||:~~~~~~~~~~~~~~~~~~~
iscR   604..3'-CAUUAAAGUAACGAGAUAUUUGAGGCAUGUAGUGAGUUACGUUCCU-5'..579
```

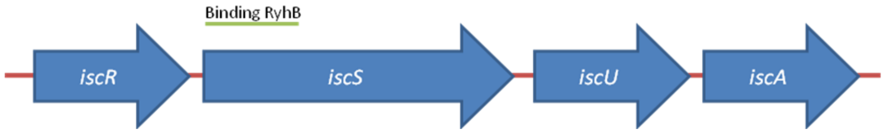


```
RyhB   35...5'-CUGAAAGCACGACAUUGCUCACAUUGCUUCCAGUAUUACUUAGCCAGCC-3'...64
              ~~~~~~~~~~~~~~~~~~|||||||~|||~~|||||||~||||||||
sufB   34...3'-AGGUCCAAAACUGUAGC~AGUC~AACGAAG~UCAUAAUGCUCUGUAAUC-5'....8
```

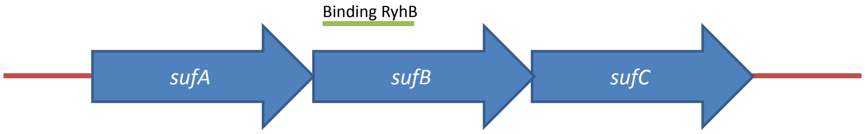

Panel C

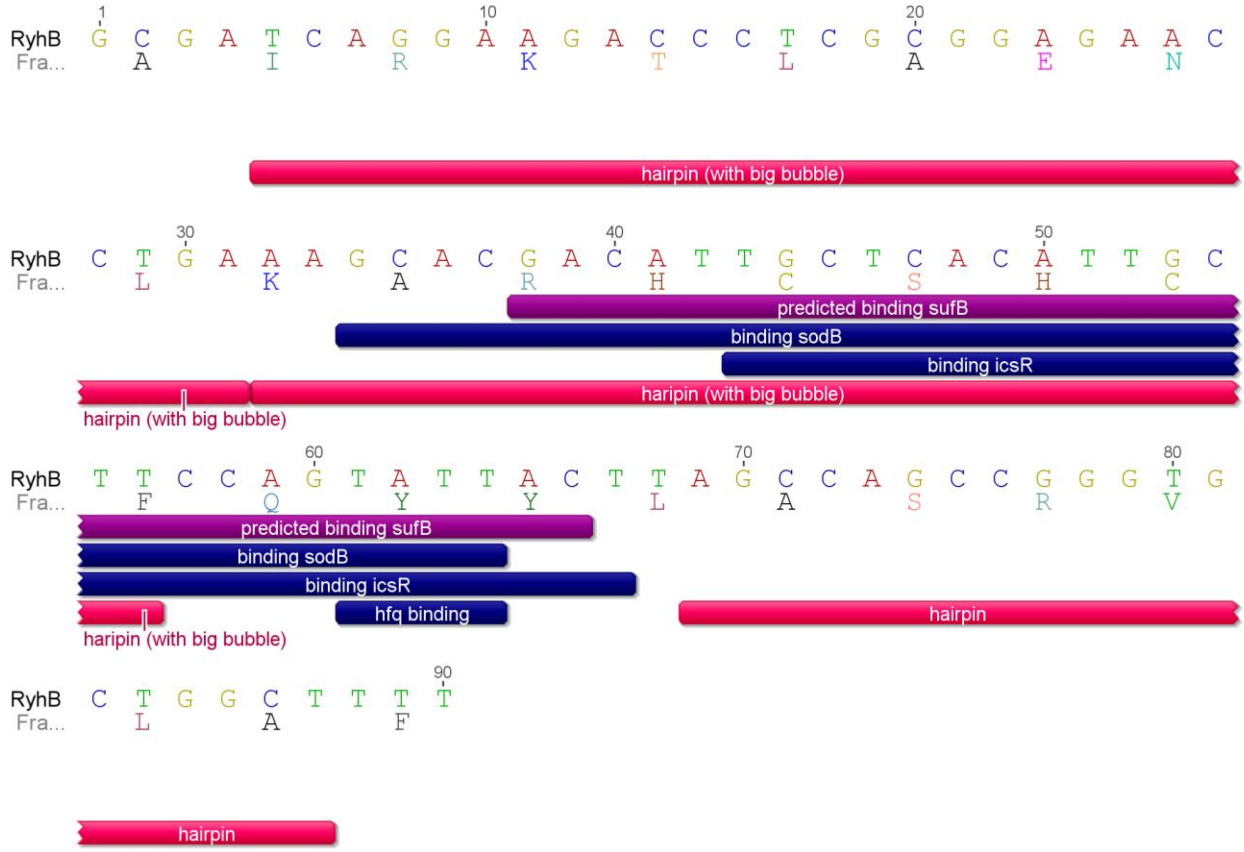

Legend: as above

Module 8

Assignment of the regulatory program to module 8

[a]Module id	[b]Regulator id	[c]Algorithm	[d]CLR score	[e]LeMoNe score	[f]Regulator
8	Fur	lemone	2,47	366,55	TF
8	MarA	lemone	2,57	200,99	TF
8	OxyS	lemone	1,64	87,3	sRNA

[a]Module id: id of cluster to which at least one regulator was assigned

[b]Regulator id: regulators assigned with CLR and LeMoNe

[c]Algorithm: indicates by which algorithm the regulator was assigned: "clr" if regulator was assigned by CLR, "lemone" if regulator was assigned by LeMoNe, "clrlemone" if regulator was assigned by both algorithms.

[d]CLR score: CLR score, the score assigned to the respective regulator by CLR. A CLR score is considered relevant if above 3 (see Materials and Methods)

[e]LeMoNe score: The score assigned to the respective regulator by LeMoNe. A LeMoNe score is considered relevant if above 100 (see Materials and Methods)

[f]Regulator: the type of the assigned regulator: "TF": transcription factor, "sRNA": sRNA

Module content:

(78 genes and 8 conditions)

yadM	predicted fimbrial-like adhesin protein
glnD	[protein-PII] uridylyltransferase
map	methionine aminopeptidase
hemB	porphobilinogen synthase (HemB)
ampH	penicillin-binding protein
panE	2-Dehydropantoate 2-reductase catalyzes the NADPH-dependent reduction of ketopantoate to pantoate.
ybaO	putative LRP-like transcriptional regulator
mdlA	predicted multidrug transporter subunit of ABC superfamily: ATP-binding component
mdlB	predicted multidrug transporter subunit of ABC superfamily: ATP-binding

	component
acrB	RND-type inner-membrane associate proton-substrate antiporter. It functions as a part of the AcrAB/TolC multidrug-efflux complex, linking electrochemical-gradient energy to the efflux of drugs from the cytoplasm
acrA	periplasmic lipoprotein component of the AcrAB-TolC multidrug efflux pump in *Escherichia coli* and can function in chimeric constructions with three other RND-family pumps, AcrD, AcrF and YhiV.
ybaL	uncharacterised member of the CPA-2 family of monovalent/proton antiporters.
nfsB	dihydropteridine reductase
fepD	ferric enterobactin ABC transporter
ybdO	predicted DNA-binding transcriptional regulator LYSR-type
ahpF	channels electrons from NAD(P)H via a series of disulfides to the AhpC component for reduction of the hydroperoxide substrate
fur	Component of: Fur-Fe+2 DNA-binding transcriptional dual regulator
fldA	Flavodoxin is an important member of the multi-enzyme complexes that are involved in the activation of anaerobic nucleoside reductase and pyruvate-formate lyase.
ybhT	small membrane protein involved in the cell envelope stress response
cmr	multidrug efflux protein belonging to the major facilitator superfamily (MFS)
grxA	reduced glutaredoxin 1, glutaredoxins are ubiquitous proteins that catalyze the reduction of disulfides via reduced glutathione (GSH)
ybjC	predicted inner membrane protein
nfsA	the major oxygen-insensitive nitroreductase present in *E. coli*
rimK	ribosomal protein S6 modification protein
ybjN	predicted oxidoreductase
pqiA	paraquat-inducible protein A
pqiB	paraquat-inducible protein B
ymbA	No information about this protein was found by a literature search conducted on January 15, 2012.
mdoC	No information about this protein was found by a literature search conducted on January 15, 2012.
mdtG	YceE drug MFS transporter
ychA	predicted transcriptional regulator,
acnA	No information about this protein was found by a literature search conducted on January 15, 2012.
ribA	GTP cyclohydrolase II catalyzes the opening of the imidazole ring of GTP and removal of pyrophosphate, the first committed step in riboflavin biosynthesis.
ompN	outer membrane pore protein N, non-specific
ydbK	predicted pyruvate:flavodoxin oxidoreductase
ydcQ	antitoxin of the HicA-HicB toxin-antitoxin system
yncD	probable TonB-dependent receptor
nhoA	N-hydroxyarylamine O-acetyltransferase
eamA	O-acetylserine/cysteine export protein
fumC	fumarase C, is induced by glycolaldehyde, the induction is oxygen and SoxRS dependent.

ydiK	inner membrane protein with eight predicted transmembrane domains.
ydjN	No information about this protein was found by a literature search conducted on January 15, 2012.
yobH	No information about this protein was found by a literature search conducted on January 15, 2012.
zwf	glucose 6-phosphate-1-dehydrogenase
nfo	endonuclease IV
yeiI	predicted kinase
inaA	pH-inducible protein involved in stress response
yfcD	predicted Nudix hydrolase
yfcE	phosphodiesterase
ligA	DNA ligase
sseB	Overexpression of sseA or sseB enhances the serine sensitivity caused by serine-mediated inhibition of homoserine dehydrogenase I activity during growth on some carbon sources, an sseB mutation causes increased fimbrial production in an *E. coli* strain that exhibits K88 fimbriae
pepB	aminopeptidase B
yfhD	membrane-bound lytic murein transglycosylase F
ygaY	ygaX and ygaY appear to be two segments of a gene encoding an uncharacterised permease of the major facilitator superfamily (MFS) of transporters
iap	alkaline phosphatase isozyme conversion protein
idi	isopentenyl diphosphate isomerase
fldB	flavodoxin 2
ygfZ	folate-binding protein, may be involved in the modification of stable RNAs
yggX	protein that protects iron-sulfur proteins against oxidative damage
mltC	membrane-bound lytic murein transglycosylase C
tolC	TolC outer membrane channel
yraL	16S rRNA 2'-O-ribose C1402 methyltransferase
lptB	LptB is the predicted ATP binding component of the LptABCFG lipopolysaccharide transporter.
yrbL	predicted protein, transcription of yrbL is regulated by $Mg2^+$ via the PhoP/PhoQ system
pabA	para-aminobenzoate synthase glutamine amidotransferase component II
envZ	sensory histidine kinase of the EnvZ/OmpR two-component system which regulates expression of the major outer membrane porin genes, ompF and ompC, in response to fluctuations in extracellular osmotic pressure.
treF	cytoplasmic trehalase, catalyze the hydrolysis of trehalose into two molecules of D-glucose
eptB	Ca2+-induced phosphoethanolamine transferase
rfaY	lipopolysaccharide core heptose (II) kinase
nepI	purine ribonucleoside exporter
frvR	member of the frvABXR operon which encodes a putative fructose-specific PEP-dependent sugar phosphotransferase system
frvX	member of the frvABXR operon which encodes a putative fructose-specific PEP-dependent sugar phosphotransferase system

kdgT	2-dehydro-3-deoxy-D-gluconate transporter
fpr	Component of: anaerobic nucleoside-triphosphate reductase activating system
soxS	transcriptional activator and participates in the removal of superoxide and nitric oxide and protection from organic solvents and antibiotics
yjjW	predicted pyruvate formate lyase activating enzyme
yjjI	in theoretical study, were found in a module enriched for factors involved in nucleotide transport and degradation of deoxyribonucleosides; under anaerobic conditions, yjji mutant produces small colonies on glucose and no growth on glycerol and nitrate
micC	small RNA that regulates expression of OmpC at the post-transcriptional level

Regulators assigned:

(LeMoNe): OxyS small RNA, and Fur, MarA transcription factors.

Module 8 is enriched by MarA targets 13 out of the 39 MarA targets in all modules) (YbaO AcrB AcrA NfsB PqiA PqiB FumC zwf nfo InaA TolC RfaY fpr)

Module 8 contains 2 targets of fur (amongst which fur itself and FepD)

Module 8 contains 3 predicted OxyS targets (sequence-based): imk InaA MltC

GO Enrichment:

None

Ecocyc Pathway Enrichment:

None

Module Description:

Module 8 contains pathways which relate to oxidative membrane stress (osmotic stress response, efflux pumps, membrane remodeling). Three regulators have been assigned to this module by LeMoNe: MarA, Fur and OxyS. MarA, is a "multiple antibiotic resistance" regulator of which indeed part of its known regulon was found in the module. MarA is an outer membrane porin involved in the efflux of several hydrophobic and amphipathic molecules and is known to be involved in resistance to antibiotics, oxidative stress [20]. The module indeed contains MarA

targets such as tolC, an outer membrane porin. Although the Fur regulon members are not well represented in this module, the autoregulated TF Fur has not only been assigned to the module, but also belongs to the module itself, further supporting its assignment. Besides its well documented role in iron homeostasis, Fur is also known to be involved in oxidative stress responses by downregulating iron uptake systems [21].

Next to these TFs also the sRNA, the sRNA OxyS known to play a regulatory role in the oxidative stress response [22] was assigned to this module. Three targets regulated by OxyS were predicted with our approach and were found in module 8, implying that OxyS regulates together with MarA the genes in module 8: RimK, a ribosomal protein S6 modification protein belonging to the ybjC-nfsA-rimK-ybjN operon, an operon which indeed is known to be regulated by (Rob/MarA/SoxS) and OxyR. So, the additional regulation of rimK (intraoperonic promotor site) by OxyS is plausible. inaA, a second predicted target of oxyS present in module 8 is pH-inducible protein involved in stress response [23, 24]. A third target of OxyS which we could predict is MltC is membrane-bound lytic murein transglycosylase C, known to be induced by oxidative stress via SoxS [25].

Module Figure (legend as above):

Panel A

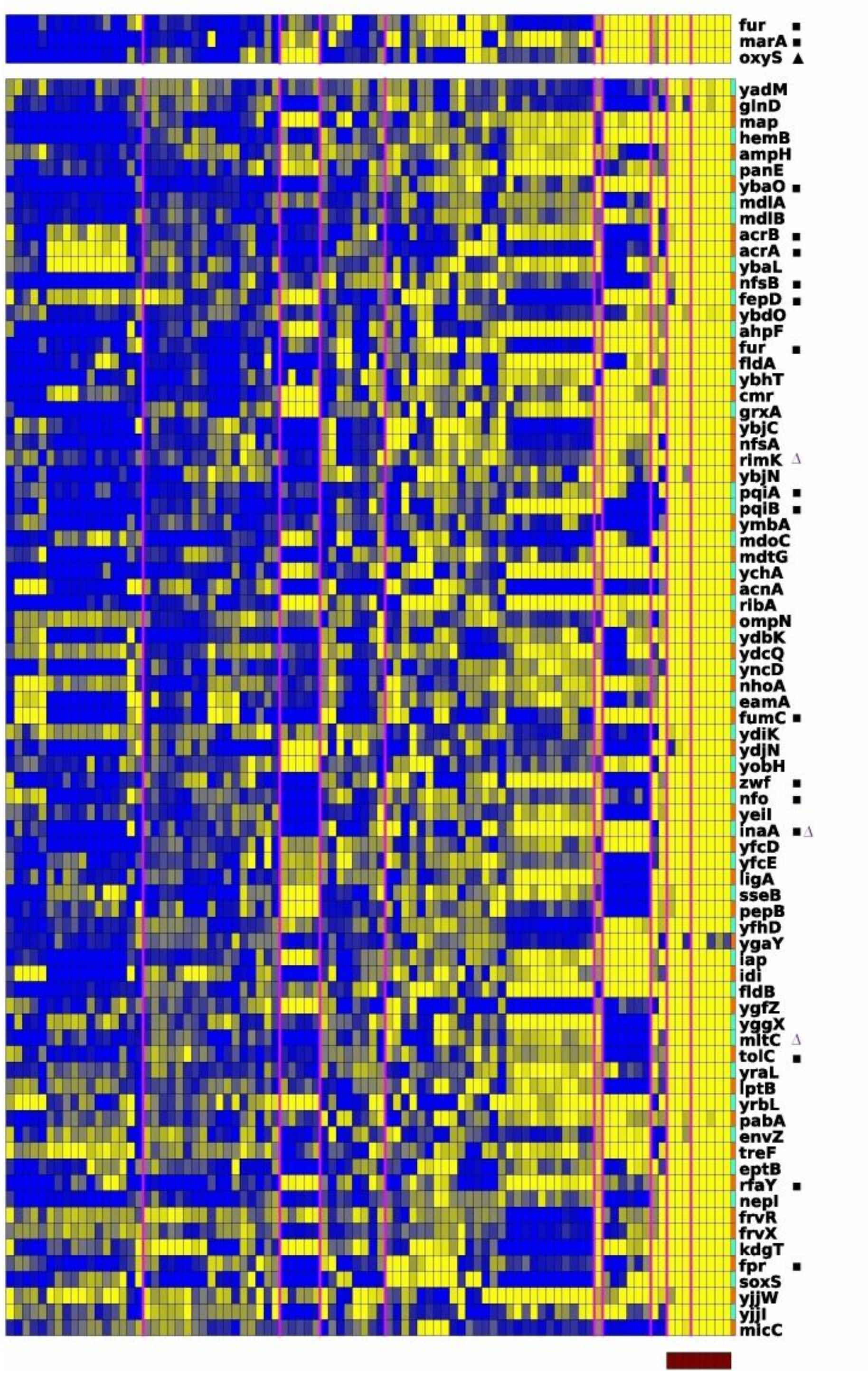

Panel B

```
OxyS   12...5'-GCACCUCUUUUA~ACCCUUGAAGUC-3'...36
       ~~~~~~~~||||||||||~~~|~~||||~~|||~~~~~~~~
rimK   -6...3'-CGUGGAGAGACAGUCUGGAC~~CAG-5'..-29


OxyS   11...5'-GGCACCUCUUUUAACCCUU-3'...30
       ~~~~~~~~||||||~|||||~||||||~~~~~~~~
inaA   -109.3'-CUGUGGCGAAAA~UGGGAA-5'.-127
```

```
OxyS  12...5'-GCACCUCUUUUAACCCUUGAA-3'...32
              ~~~~~~~~||||||||||~~|||||:|||~~~~~~~~
mltC  -97..3'-CGUGGAGAAA--UGGGAGCUU-5'.-115
```

Legend: as above

Module 20

Assignment of the regulatory program to module 20

[a]Module id	[b]Regulator id	[c]Algorithm	[d]CLR score	[e]LeMoNe score	[f]Regulator
20	soxR	lemone	2,13	226,23	TF
20	iclR	lemone	1,35	128,38	TF
20	ryfA	lemone	0,74	103,72	sRNA

[a]Module id: id of cluster to which at least one regulator was assigned

[b]Regulator id: regulators assigned with CLR and LeMoNe

[c]Algorithm: indicates by which algorithm the regulator was assigned: “clr” if regulator was assigned by CLR, “lemone” if regulator was assigned by LeMoNe, “clrlemone” if regulator was assigned by both algorithms.

[d]CLR score: CLR score, the score assigned to the respective regulator by CLR. A CLR score is considered relevant if above 3 (see Materials and Methods)

[e]LeMoNe score: The score assigned to the respective regulator by LeMoNe. A LeMoNe score is considered relevant if above 100 (see Materials and Methods)

[f]Regulator: the type of the assigned regulator: “TF”: transcription factor, “sRNA”: sRNA

Module content:

(53 genes and 6 conditions)

yaaA	protein that reduces intracellular iron levels under peroxide stress
yadE	predicted polysaccharide deacetylase lipoprotein
yafD	conserved protein, has similarity to *E. coli* YadD, which is encoded within the panBCD gene cluster
ybaO	putative LRP-like transcriptional regulator, may be a regulator with a role in Rob-mediated regulation
hemH	Ferrochelatase is the terminal enzyme in the heme biosynthesis pathway and catalyzes the insertion of Fe^{2+} into protoporphyrin IX
ybiJ	No information about this protein was found by a literature search conducted on January 15, 2012.
grxA	reduced glutaredoxin 1
cspH	DNA replication inhibitor

cspG	cold shock protein
ycfQ	predicted DNA-binding transcriptional regulator
marR	participates in controlling several genes involved in resistance to antibiotics, multidrug efflux, oxidative stress, organic solvents, and heavy metals
marA	participates in controlling several genes involved in resistance to antibiotics, oxidative stress, organic solvents, and heavy metals
marB	multiple antibiotic resistance protein
ydfH	predicted DNA-binding transcriptional regulator
malI	controls genes related to the maltose system
cho	endonuclease of nucleotide excision repair
yeaP	diguanylate cyclase
znuC	ATP-binding component of ABC transporter
ypfH	esterase
hda	regulator of DnaA that prevents premature reinitiation of DNA replicatio
trmJ	tRNA Um32 methyltransferase / tRNA Cm32 methyltransferase
trxC	reduced thioredoxin 2
yfiP	among the genes most highly expressed during adaptation to all acids
yfiR	predicted periplasmic protein involved in swarming motility (YfiR)
yqfA	predicted oxidoreductase, inner membrane subunit
yqgA	putative transport protein
yghB	mutants yghB showed reduced biofilm formation in urine by 18 to 43% compared with the wild type ($P < 0.05$).
ygiD	predicted dioxygenase, is involved in biofilm formation in urine
yqiJ	putative oxidoreductase
exuR	transcription factor that negatively regulates its own synthesis and represses transcription of the operons involved in transport and catabolism of galacturonate and glucuronate
yqjF	predicted quinol oxidase subunit
yhaK	belongs to a subclass of the bicupin family and may be involved in chloride binding or sensing of oxidative stress
yhcN	involved in the cellular response to hydrogen peroxide stress
aaeA	AaeAB Hydroxylated, Aromatic Carboxylic Acid Efflux Transport System Protein A
aaeX	gene with crp regulation
yhfZ	DNA double-strand breaks at multiple positions also induce an oriC proximal hotspot at 3510 kb on the chromosome, the yhfZ gene
glpE	minor thiosulfate sulfurtransferase in *E. coli*
gntR	transcription factor that negatively regulates the operon involved in the catabolism of d-gluconate via the Entner-Doudoroff pathway and also represses genes involved in two different systems related to d-gluconate uptake: gluconate I and gluconate II
yhhW	was identified as a Pirin homolog and was shown to have quercetin 2,3-dioxygenase activity, releasing carbon monoxide
yhjY	putative lipase
ibpB	small heat shock protein that binds to aggregated proteins

mdtL	a member of the major facilitator superfamily (MFS)
asnA	asparagine synthetase A
soxR	transcription factor, controls the transcription of the regulon involved in defense against redox-cycling drugs
ulaR	transcription factor, represses transcription of a divergent operon (ula) involved in transport and utilization of L-ascorbate catabolism
ulaG	L-ascorbate 6-phosphate lactonase
ulaB	utilization of L-ascorbate, component of: L-ascorbate PTS permease
ulaC	utilization of L-ascorbate, component of: L-ascorbate PTS permease
ulaD	utilization of L-ascorbate, 3-keto-L-gulonate 6-phosphate decarboxylase
ulaE	utilization of L-ascorbate, L-xylulose 5-phosphate 3-epimerase
ulaF	L-ribulose 5-phosphate 4-epimerase is an enzyme in the pathway of anaerobic L-ascorbate degradation
ytfH	No information about this protein was found by a literature search conducted on January 15, 2012.
insB	IS1 is the smallest insertion sequence in *E. coli*. It codes for three proteins, InsA, InsB and InsAB

Regulators assigned:

3 regulators were assigned to that cluster, all recognized with LeMoNe: RyfA (small RNA); SoxR and IclR (transcription factors). SoxR is autoregulated and is part of the module itself as well. No other SoxR targets were found in the module.

number of all the SoxR targets in modules is 3.

Module 20 contains one predicted targets (sequence-based) of the sRNA RyfA: ZnuC

GO Enrichment:

- Biological regulation. 54% of gene content can be described with this GO term. (ibpB soxR cspH gntR ybaO marA ytfH cspG ycfQ grxA exuR hda malI ulaR ulaC trxC ulaB marR ydfH)
- Nucleobase, nucleoside, nucleotide and nucleic acid metabolic process. 54% of gene content can be described with this GO term. (soxR cspH gntR ybaO marA cspG ycfQ ytfH grxA exuR hda malI ulaR ulaD asnA cho trmJ marR ydfH)

Ecocyc Pathway Enrichment:

- L-ascorbate degradation: ulaD,ulaF are ulaE invovled in this pathway and this pathway is overpresented with *p*-value 0.00024205

Module Description:

Module 20 contains genes belonging to pathways involved in transport, oxidative stress response (Mar and Sox operons) and gluconate, ascorbate utilization. SoxR which was reliably assigned to the module is also part of the module. The regulator IclR also assigned to the module is known to regulate the glyoxylate bypass operon [26, 27].

According to our predictions, the sRNA assigned to this module RyfA, which has no assigned function yet would have one predicted target in the module, i.e. ZnuC, the ATP-binding component of an ABC transporter involved in high-affinity zinc uptake (ZnuABC). *znuC* transcripts were shown to disappear or markedly decreased at 5 min after zinc addition [22]. Such quick induction or repression of the zinc-responsive genes upon increasing environmental zinc levels suggests a regulation mechanism mediated by sRNAs. Some of the enzymes being expressed in the module are indeed known to depend upon a Zn containing active site (e.gUlaE [28]). In literature we found an indirect relation between SoxS and RyfA through the regulation of the predicted target ZnuC. SoxS is known to increase the expression of the zinc uptake system ZnuACB in *Escherichia coli* although no direct binding of SoxS to the promoter of *znuACB* has been observed [29].

Module Figure:

Panel A:

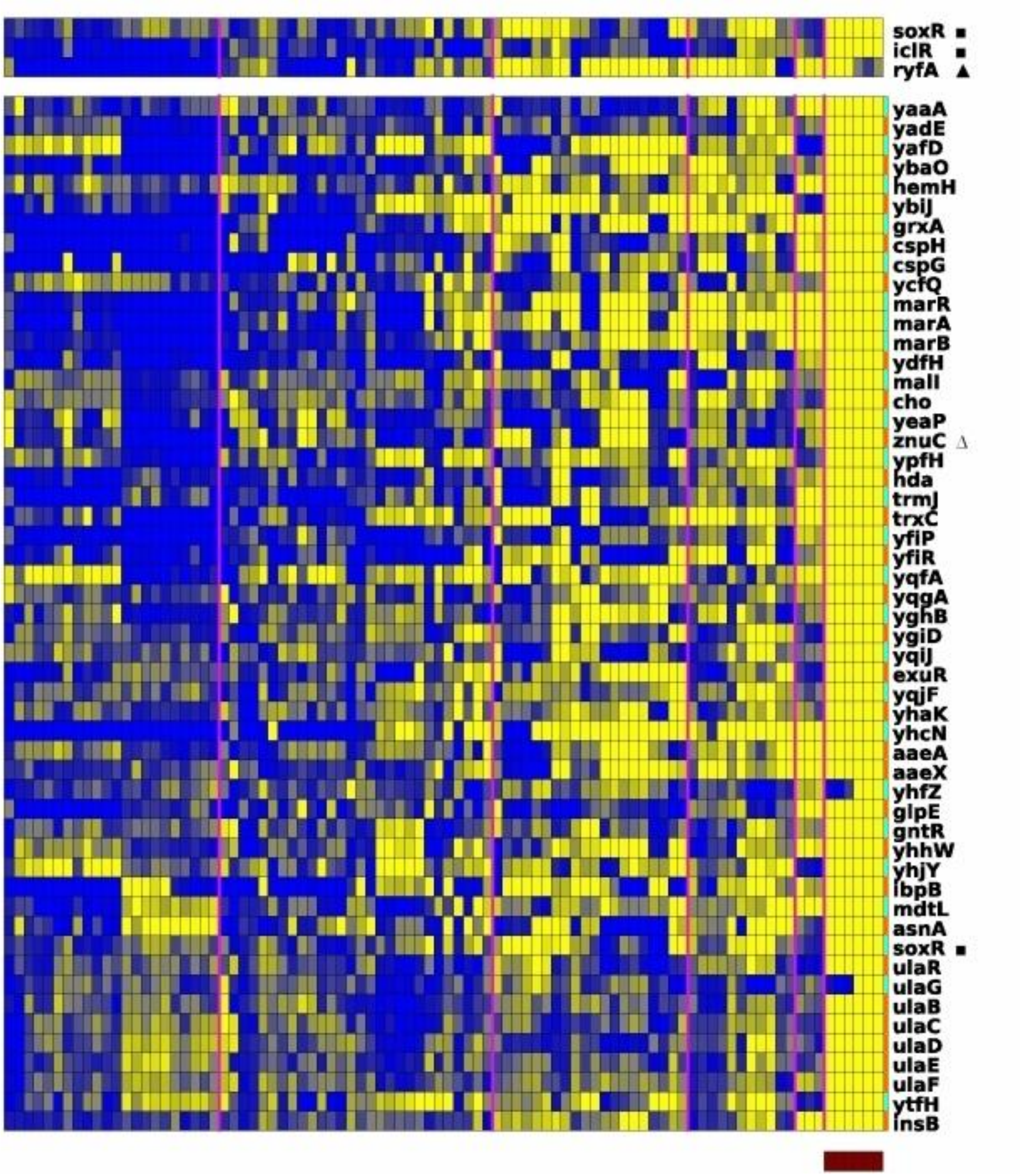

Panel B

```
RyfA   259..5'-GCCGCCCUUUGGG-3'..272
               ~~~~~~~~|||||~|||||||~~~~~~~~
znuC   31...3'-CGGCGAGAGACCC-5'...18
```

Legend: as above

Module 61

Assignment of the regulatory program to module 61

[a]Module id	[b]Regulator id	[c]Algorithm	[d]CLR score	[e]LeMoNe score	[f]Regulator
61	YahA	lemone	0,27	182,14	TF
61	AdiY	lemone	0,48	157,89	TF
61	tpke70	clr	3,16	47,44	sRNA
61	CdaR	clr	4.01	1.85	TF

[a]Module id: id of cluster to which at least one regulator was assigned

[b]Regulator id: regulators assigned with CLR and LeMoNe

[c]Algorithm: indicates by which algorithm the regulator was assigned: “clr” if regulator was assigned by CLR, “lemone” if regulator was assigned by LeMoNe, “clrlemone” if regulator was assigned by both algorithms.

[d]CLR score: CLR score, the score assigned to the respective regulator by CLR. A CLR score is considered relevant if above 3 (see Materials and Methods)

[e]LeMoNe score: The score assigned to the respective regulator by LeMoNe. A LeMoNe score is considered relevant if above 100(see Materials and Methods)

[f]Regulator: the type of the assigned regulator: “TF”: transcription factor, “sRNA”: sRNA

Module content:

(44 genes and 13 conditions)

cdaR	regulates genes involved in the uptake and metabolism of galactarate and glucarate
ykgE	predicted oxidoreductase
ykgF	predicted amino acid dehydrogenase with NAD(P)-binding domain and ferridoxin-like domain
ykgG	predicted transporter
dcuC	dicarboxylate transporter
ybiA	A ybiA mutant has a defect in swarming, but not swimming motility
narL	nitrate/nitrite response regulator
narX	NarX and NarQ proteins are paralogous sensor kinases that, together with the response regulators NarP and NarL, form a complex signal transduction system which controls anaerobic respiratory gene expression in response to nitrate and

	nitrite.
narK	MFS nitrate/nitrite antiporter
narG	nitrate reductase A, α subunit
narH	nitrate reductase A, β subunit
narJ	molybdenum cofactor assembly chaperone subunit (δ subunit) of nitrate reductase 1
yecJ	No information about this protein was found by a literature search conducted on January 15, 2012
napB	small subunit of periplasmic nitrate reductase, cytochrome c550 protein
napH	NapH together with NapG is required for electron transfer from ubiquinol, but not menaquinol, via NapC to the NapAB complex
napG	is required for electron transfer from ubiquinol, but not menaquinol, via NapC to the NapAB complex
napA	large subunit of periplasmic nitrate reductase, molybdoprotein
napD	signal peptide-binding chaperone for NapA
napF	ferredoxin-type protein
lpxP	palmitoleoyl acyltransferase
murQ	N-acetylmuramic acid 6-phosphate etherase
yfeW	Enzyme: penicillin binding protein 4B
ygcO	predicted 4Fe-4S cluster-containing protein, no information about this protein was found by a literature search conducted on January 15, 2012
gudX	glucarate dehydratase-related protein
gudP	YgcZ MFS transporter
glcG	a protein of unknown function encoded by a gene within the glycolate utilization operon
glcE	glycolate oxidase, predicted FAD-binding subunit
glcD	glycolate oxidase, predicted FAD-linked subunit
yhaM	transport and metabolism of threonine and serine?
yhaO	STP transporter, may function as an amino acid/proton symporter
tdcA	participates in controlling genes (tdc operon) involved in transport and metabolism of threonine and serine
garK	glycerate kinase I
garR	tartronate semialdehyde reductase, catalyzes the reduction of tatronate semialdehyde to yield glycerate
garL	α-dehydro-β-deoxy-D-glucarate aldolase
garP	MFS transporter, may function as a proton-driven glucarate uptake system
garD	galactarate dehydratase
nikC	Component of: nickel ABC transporter
nikD	Component of: nickel ABC transporter
yhjX	MFS transporter, may function as an exchange system for carboxylates, possibly for formate and oxalate
malS	α-amylase, a periplasmic enzyme that degrades linear dextrins of at least three glucose residues
yidF	distantly related to the anaerobic sulfatase maturation enzyme family
yihL	putative transcriptional regulator belongs to the GntR family

yihM	Expression of yihM is upregulated by hexane and is increased in an organic solvent-tolerant strain. Overexpression of yihM does not increase tolerance to hexane or cyclohexane
tpke70	small RNA

Regulators assigned:

Four regulators were assigned to that cluster: small RNA tpke70 (CLR), the transcription factors CdaR(CLR),YahA (LeMoNe), AdiY (LeMoNe).

tpke70 sRNA predicted targets: napG napD

GO Enrichment:

None

Ecocyc Pathway Enrichment:

- NarX Nitrate/Nitrite-Dependent Two-Component Regulatory System: narL and narX are involved in this pathway and this pathway is overpresented with *p*-value 8.5999e-005
- D-galactarate degradation: garR,garK,garD and garL are involved in this pathway and this pathway is overpresented with *p*-value 1.4944e-007
- D-glucarate degradation: garR,garK and garL are involved in this pathway and this pathway is overpresented with *p*-value 1.754e-005

Module Description

Module 61 (44 genes) contains genes related to oxidation-reduction, electron transport and energy generation. Three TFs, AdiY and YahA were assigned to this module by LeMoNe and one CdaR by CLR. According to Ecocyc and a recent literature overview the cellular role of YahA, a c-di-GMP-specific phosphodiesterase is yet unknown (YahA contains an EAL domain close to an N-terminal putative DNA-binding domain). AdiY was previously shown to be strongly upregulated after a rapid decrease in external pH.Its known target, the arginine decarboxylase system (*adi*) is known to be induced in rich medium, under anaerobic conditions, and at low pH, conditions under which genes present in the module are also known to be

expressed. CdaR, regulates genes involved in the uptake and metabolism of galactarate and glucarate and is also found to be one of the regulators for which the targets are enriched in the module. BBesides these TFs also Tpke70 a small RNA of approximately 40 nt in length with yet unknown function was assigned to this module.

Module 61 also contains two predicted targets of Tpke70 that is NapG and NapD (predicted using sequence-based methods) both parts of the periplasmic nitrate reductase system in *E. coli*.

Module Figure (legend as above):

Panel A

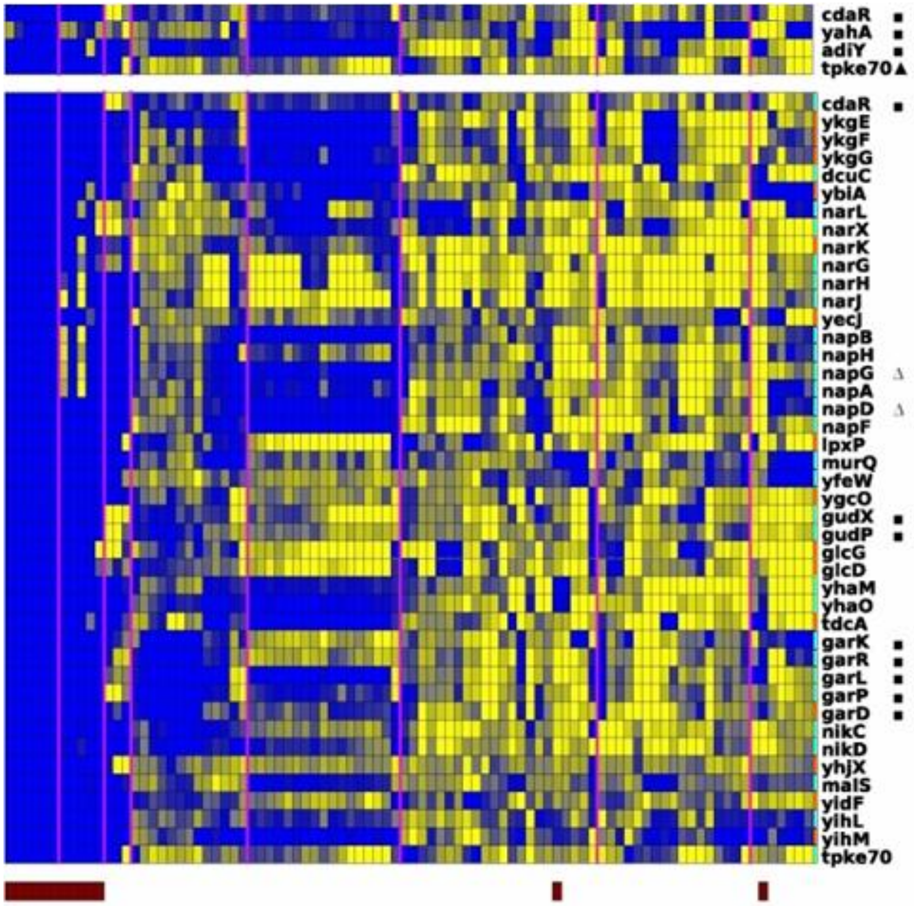

Panel B

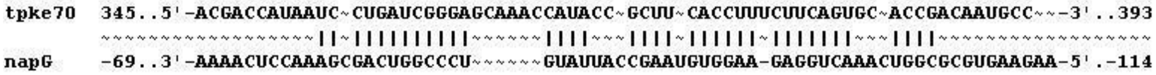

```
tpke70   289..5'-UGGCUGCGCCAGAGAGACGGGAGAGAACAUAGGUGCCAUUGGUUGUGGCGACAU-3'..342
         ~~~~~~~~~~~~~~~~~~|||~|~|||||~||~||~|||:~|||||||||:|~~~~~~~~~~~~~~~~~
napD     -90..3'-AUCUAGGGCCUCUCGCAGCCCGCU~UCUAUUAACGGUAACCGAGUGUCGAUAG~-5'.-122
```

Legend: as above

Module 12

Assignment of the regulatory program to module 12

[a]Module id	[b]Regulator id	[c]Algorithm	[d]CLR score	[e]LeMoNe score	[f]Regulator
12	MalT	lemone	1,08	122,58	TF
12	NadR	lemone	-0,54	112,12	TF
12	CspE	lemone	1.15	128.2	TF
12	tpke70	lemone	0,31	108,74	sRNA

[a]Module id: id of cluster to which at least one regulator was assigned

[b]Regulator id: regulators assigned with CLR and LeMoNe

[c]Algorithm: indicates by which algorithm the regulator was assigned: "clr" if regulator was assigned by CLR, "lemone" if regulator was assigned by LeMoNe, "clrlemone" if regulator was assigned by both algorithms.

[d]CLR score: CLR score, the score assigned to the respective regulator by CLR. A CLR score is considered relevant if above 3 (see Materials and Methods)

[e]LeMoNe score: The score assigned to the respective regulator by LeMoNe. A LeMoNe score is considered relevant if above 100 (see Materials and Methods)

[f]Regulator: the type of the assigned regulator: "TF": transcription factor, "sRNA": sRNA

Module content:

(28 genes and 5 conditions)

brnQ	branched chain amino acid LIVCS transporter
glnK	Subunit composition of nitrogen regulatory protein GlnK = [GlnK]3
amtB	a member of the Amt family of ammonium/ammonia transporters
ybeD	conserved protein of unknown function
asnB	Subunit composition of asparagine synthetase B = [AsnB]2, catalyzing the glutamine-dependent and ammonia-dependent conversion of aspartate to asparagine.
potF	Component of: putrescine ABC transporter
ycaD	uncharacterised member of the major facilitator superfamily (MFS) of transporters,

	may function as a proton-driven metabolite uptake system.
rutG	uncharacterized member of the NCS2 family of nucleobase transporters, may function as a proton-driven uracil uptake system
rutF	flavin reductase whose activity is required for the first catalytic step, the flavin hydroperoxide-catalyzed ring opening by the RutA pyrimidine oxygenase
rutE	*E. coli* contains a previously undescribed pathway for pyrimidine degradation, RutE may function as a malonic semialdehyde reductase
rutD	*E. coli* K-12 contains a previously undescribed pathway for pyrimidine degradation, RutD may thus be required to remove a toxic intermediate or byproduct of the pathway
rutC	*E. coli* K-12 contains a previously undescribed pathway for pyrimidine degradation, RutC may thus be required to remove a toxic intermediate or byproduct of the pathway
rutB	peroxyureidoacrylate / ureidoacrylate amido hydrolase (RutB)
rutA	pyrimidine oxygenase
chaC	predicted cation transport protein
abgR	predicted DNA-binding transcriptional regulator, LYSR-type
yddM	predicted DNA-binding transcriptional regulator
ddpX	D-Ala-D-Ala dipeptidase
ynfM	an uncharacterised member of the major facilitator superfamily (MFS) of transporters, it may function as a proton-driven drug efflux system
yedL	predicted acyltransferase
cbl	regulator involved in the expression of genes required for aliphatic sulfonate utilization and homeostatic response to sulfate starvation
nac	The genes regulated by Nac are transcribed by RNA polymerase σ70. These genes are coupled to the nitrogen regulatory (Ntr) system, which is σ54 dependent, through Nac, whose transcription is activated by NtrC.
rumA	23S rRNA m5U1939 methyltransferase
yqfA	predicted oxidoreductase, inner membrane subunit
deaD	DEAD-box RNA helicase
nlpI	lipoprotein involved in cell division
glnA	glutamine synthetase
yjcB	predicted inner membrane protein

Regulators assigned:

The small RNA tpke70 (LeMoNe), the transcription factors CspE (LeMoNe), MalT (LeMoNe), NadR (LeMoNe).

No known targets of MalT and NadR were found in the module.

The module contains one tpke70 sRNA predicted target (sequence-based methods): ybed

GO Enrichment:

None

Ecocyc Pathway Enrichment:

- Nitrogen Regulation Two-Component System: glnA and glnK are invovled in this pathway and this pathway is overpresented with *p*-value 0.00040131.
- Asparagine biosynthesis III: asnB is invovled in this pathway and this pathway is overpresented with *p*-value 0.00079608.
- Glutamine biosynthesis I: glnA is invovled in this pathway and this pathway is overpresented with *p*-value 0. (according the database of Ecocyc, there is only one gene in this pathway also enriched with GO terms)

Module Description:

Module 12 contains genes which are related to NAD regulation, and amino acid metabolism (such as pyrimidine degradation) and organic cation transport genes (unknown targets of MalT in module). The module contains one predicted target of Tpke 70 i.e. YbeD, a conserved protein of unknown function [30].

Module Figure: **legend as above**

Panel A:

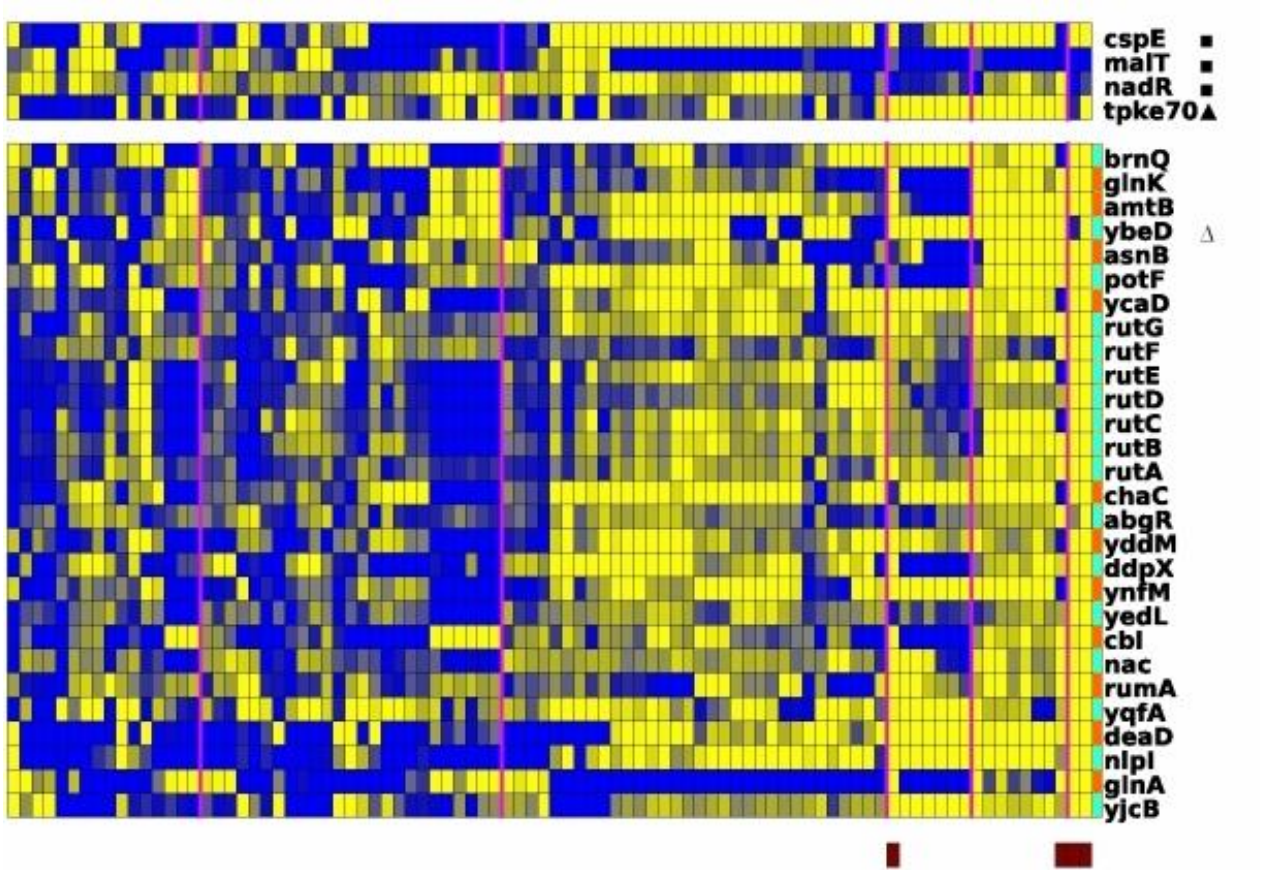

Panel B:

```
tpke70   39...5'-UGGCCGAUUUGAGGAGGGAAAGAGUAAGAGCAGUUUGUUAAAUGUACAACG-3'...70
         ~~~~~~~~~~~~~~~~~~||||||~~~~~||||~~||||||||||||||~~~~~~~~~~~~~~~~~~
ybeD     -142.3'-~~CAUUCAUUUUCCUCAUCC-CUUAAGUUCGUCAAGCAAUUCAAACCAAAA-5'.-112
```

Legend: as above

Module 42

Assignment of the regulatory program to module 42

[a]Module id	[b]Regulator id	[c]Algorithm	[d]CLR score	[e]LeMoNe score	[f]Regulator
42	rydC	clrlemone	3,06	108,85	sRNA
42	nadR	clr	3,01	85,76	TF

[a]Module id: id of cluster to which at least one regulator was assigned

[b]Regulator id: regulators assigned with CLR and LeMoNe

[c]Algorithm: indicates by which algorithm the regulator was assigned: "clr" if regulator was assigned by CLR, "lemone" if regulator was assigned by LeMoNe, "clrlemone" if regulator was assigned by both algorithms.

[d]CLR score: CLR score, the score assigned to the respective regulator by CLR. A CLR score is considered relevant if above 3 (see Materials and Methods)

[e]LeMoNe score: The score assigned to the respective regulator by LeMoNe. A LeMoNe score is considered relevant if above 100 (see Materials and Methods)

[f]Regulator: the type of the assigned regulator: "TF": transcription factor, "sRNA": sRNA

Module content:

(2 genes and 19 conditions)

yheU	No information about this protein was found
rtcB	RtcB is an ATP-independent 3'-5' RNA ligase

Regulators assigned:

2 regulators were assigned to that cluster: RydC small RNA, and NadR transcription factor.

RydC regulator was recognized with CLR and LeMoNe.NadR regulator was assigned by CLR.

For NadR 4 targets are known according to RegulonDB none of which were found in the module.

Module Description:

GO Enrichment:

None

Ecocyc Pathway Enrichment:

None

Module Description:

Despite the fact that this module only contains two genes, the regulator NadR and the sRNA RydC were reliably assigned to the module (assignment by both LeMoNe and CLR). Based on our predictions the module does not contain direct targets of RydC implying a potential direct or indirect regulatory interaction between NadR and RydC. We could indeed find an interaction region between RydC and *nadR* which is, however, located quite far downstream in the *nadR* TU (200NT before the stop codon). We could not find further literature back up to support this interaction.

Module Figure (legend as above):

PanelA

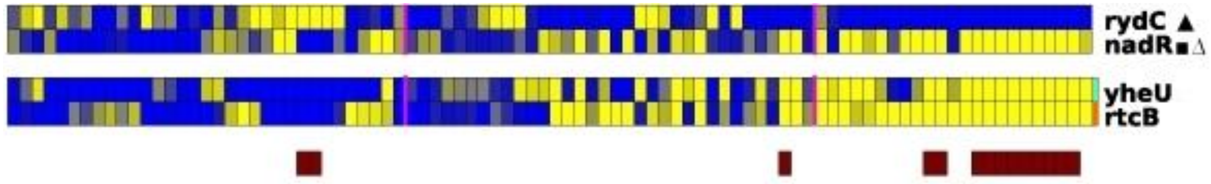

Panel B

```
RydC  1....5'-CUUCCGAUGUAGACCCGUAU-3'...20
      ~~~~~~~~|||||~~|~~~|||||||||~~~~~~~~
nadR  1176.3'-GAAGGAGAAG~UUGGGCGUG-5'.1157
```

Legend: as above

References

1. Storz G, Vogel J, Wassarman KM: **Regulation by small RNAs in bacteria: expanding frontiers**. *Mol Cell* 2011, **43**(6):880-891.
2. Zhang XS, Garcia-Contreras R, Wood TK: ***Escherichia coli* transcription factor YncC (McbR) regulates colanic acid and biofilm formation by repressing expression of periplasmic protein YbiM (McbA)**. *ISME J* 2008, **2**(6):615-631.
3. Opdyke JA, Kang JG, Storz G: **GadY, a Small-RNA Regulator of Acid Response Genes in *Escherichia coli***. *J Bacteriol* 2004, **186**(20):6698-6705.
4. Tramonti A, De Canio M, De Biase D: **GadX/GadW-dependent regulation of the *Escherichia coli* acid fitness island: transcriptional control at the gadY-gadW divergent promoters and identification of four novel 42 bp GadX/GadW-specific binding sites**. *Mol Microbiol* 2008, **70**(4):965-982.
5. Hayes ET, Wilks JC, Sanfilippo P, Yohannes E, Tate DP, Jones BD, Radmacher MD, BonDurant SS, Slonczewski JL: **Oxygen limitation modulates pH regulation of catabolism and hydrogenases, multidrug transporters, and envelope composition in *Escherichia coli* K-12**. *BMC Microbiol* 2006, **6**:89.
6. Hobman JL, Wilkie J, Brown NL: **A design for life: prokaryotic metal-binding MerR family regulators**. *Biometals : an international journal on the role of metal ions in biology, biochemistry, and medicine* 2005, **18**(4):429-436.
7. Yamamoto K, Ishihama A: **Transcriptional response of *Escherichia coli* to external copper**. *Mol Microbiol* 2005, **56**(1):215-227.
8. Xi H, Schneider BL, Reitzer L: **Purine catabolism in *Escherichia coli* and function of xanthine dehydrogenase in purine salvage**. *J Bacteriol* 2000, **182**(19):5332-5341.
9. Tokumoto U, Takahashi Y: **Genetic analysis of the isc operon in *Escherichia coli* involved in the biogenesis of cellular iron-sulfur proteins**. *J Biochem* 2001, **130**(1):63-71.
10. Wu Y, Outten FW: **IscR controls iron-dependent biofilm formation in *Escherichia coli* by regulating type I fimbria expression**. *J Bacteriol* 2009, **191**(4):1248-1257.
11. Takahashi Y, Tokumoto U: **A third bacterial system for the assembly of iron-sulfur clusters with homologs in archaea and plastids**. *J Biol Chem* 2002, **277**(32):28380-28383.
12. Whipp MJ, Camakaris H, Pittard AJ: **Cloning and analysis of the shiA gene, which encodes the shikimate transport system of *escherichia coli* K-12**. *Gene* 1998, **209**(1-2):185-192.
13. Layer G, Gaddam SA, Ayala-Castro CN, Ollagnier-de Choudens S, Lascoux D, Fontecave M, Outten FW: **SufE transfers sulfur from SufS to SufB for iron-sulfur cluster assembly**. *J Biol Chem* 2007, **282**(18):13342-13350.
14. Outten FW, Wood MJ, Munoz FM, Storz G: **The SufE protein and the SufBCD complex enhance SufS cysteine desulfurase activity as part of a sulfur transfer pathway for Fe-S cluster assembly in *Escherichia coli***. *J Biol Chem* 2003, **278**(46):45713-45719.
15. Bolstad HM, Wood MJ: **An in vivo method for characterization of protein interactions within sulfur trafficking systems of *E. coli***. *J Proteome Res* 2010, **9**(12):6740-6751.
16. Masse E, Gottesman S: **A small RNA regulates the expression of genes involved in iron metabolism in *Escherichia coli***. *Proc Natl Acad Sci U S A* 2002, **99**(7):4620-4625.
17. Masse E, Vanderpool CK, Gottesman S: **Effect of RyhB small RNA on global iron use in *Escherichia coli***. *J Bacteriol* 2005, **187**(20):6962-6971.
18. Semsey S, Andersson AM, Krishna S, Jensen MH, Masse E, Sneppen K: **Genetic regulation of fluxes: iron homeostasis of *Escherichia coli***. *Nucleic Acids Res* 2006, **34**(17):4960-4967.
19. Desnoyers G, Morissette A, Prevost K, Masse E: **Small RNA-induced differential degradation of the polycistronic mRNA iscRSUA**. *EMBO J* 2009, **28**(11):1551-1561.
20. Cohen SP, Hachler H, Levy SB: **Genetic and functional analysis of the multiple antibiotic resistance (mar) locus in *Escherichia coli***. *J Bacteriol* 1993, **175**(5):1484-1492.
21. Imlay JA: **Cellular defenses against superoxide and hydrogen peroxide**. *Annu Rev Biochem* 2008, **77**:755-776.

22. Altuvia S, Weinstein-Fischer D, Zhang A, Postow L, Storz G: **A small, stable RNA induced by oxidative stress: role as a pleiotropic regulator and antimutator**. *Cell* 1997, **90**(1):43-53.
23. Slonczewski JL, Gonzalez TN, Bartholomew FM, Holt NJ: **Mu d-directed lacZ fusions regulated by low pH in *Escherichia coli***. *J Bacteriol* 1987, **169**(7):3001-3006.
24. White S, Tuttle FE, Blankenhorn D, Dosch DC, Slonczewski JL: **pH dependence and gene structure of inaA in *Escherichia coli***. *J Bacteriol* 1992, **174**(5):1537-1543.
25. Pomposiello PJ, Koutsolioutsou A, Carrasco D, Demple B: **SoxRS-regulated expression and genetic analysis of the yggX gene of *Escherichia coli***. *J Bacteriol* 2003, **185**(22):6624-6632.
26. Gui L, Sunnarborg A, Pan B, LaPorte DC: **Autoregulation of iclR, the gene encoding the repressor of the glyoxylate bypass operon**. *J Bacteriol* 1996, **178**(1):321-324.
27. Peskov K, Goryanin I, Prank K, Tobin F, Demin O: **Kinetic modeling of ace operon genetic regulation in *Escherichia coli***. *J Bioinform Comput Biol* 2008, **6**(5):933-959.
28. Shi R, Pineda M, Ajamian E, Cui Q, Matte A, Cygler M: **Structure of L-xylulose-5-Phosphate 3-epimerase (UlaE) from the anaerobic L-ascorbate utilization pathway of *Escherichia coli*: identification of a novel phosphate binding motif within a TIM barrel fold**. *J Bacteriol* 2008, **190**(24):8137-8144.
29. Warner DM, Levy SB: **SoxS increases the expression of the zinc uptake system ZnuACB in an *Escherichia coli* murine pyelonephritis model**. *J Bacteriol* 2012, **194**(5):1177-1185.
30. Kozlov G, Elias D, Semesi A, Yee A, Cygler M, Gehring K: **Structural similarity of YbeD protein from *Escherichia coli* to allosteric regulatory domains**. *J Bacteriol* 2004, **186**(23):8083-8088.
