# Supplementary material for: Inferring the relation between transcriptional and posttranscriptional regulation from expression compendia
Source: BMC Microbiol. 2014 Jan 27;14:14. doi: 10.1186/1471-2180-14-14 (PMC3948049; doi:10.1186/1471-2180-14-14)
Supplement: Additional file 5: Table S4 — Description of the expression compendium. [file 1471-2180-14-14-S5.pdf]

Additional file 5 - Table 4: Description of the expression compendium

[a]Experiment id	[b]Platform	[c]Accession	[d]Description	[e]Database	[f]Number of contrast
1	Ecoli_ASv2	GSE4562	*E. coli* K-12 mutants yceP, trpE, and tnaA biofilm cell 24 hr for indole paper	GEO	6
2	Ecoli_ASv2	GSE6923	*E. coli* K-12 wild type with R1drd19 biofilm vs wild type without R1drd19 biofilm	GEO	9
3	E_coli_2	GSE6195	EHEC hydroxyindole project	GEO	3
4	Ecoli_ASv2	GSE4778	Acid-Shifted Time Course of Gene Expression Profiles of Aerobically Grown Escherichia coli K-12	GEO	19
5	E_coli_2, Ecoli_ASv2	GSE8706	*E. coli* BW25113 yncC vs wt biofilm cells in LB 15h 37C and MG1655 yncC vs wt colony cells in LB plates 15h 37C	GEO	3
6	E_coli_2	GSE4724	Transcriptome analysis of the arginine regulon in *E.coli*	GEO	8
7	Ecoli_ASv2	GSE9814	RpoS regulation of gene expression during exponential growth of *E. coli* K-12	GEO	5
8	E_coli_2	GSE6992	Expression data from a paraquat time course experiment in wild type and SoxR deficient strains	GEO	17
9	E_coli_2	GSE5552	*E coli* O157:H7 w/t LB-Glu 7 hr biofilm cells with various chemicals	GEO	3
10	Ecoli_ASv2	GSE1121	changes of global gene expression in *E. coli* during an oxygen shift	GEO	42
11	E_coli_2	GSE7243	Expression data from overnight minimal medium culture	GEO	5
12	Ecoli_ASv2	GSE9755	Tannin resistance strategies of *Escherichia coli* in anaerobic conditions	GEO	3
13	E_coli_2	GSE3665	Global transcriptional machinery engineering in *E. coli* in the presence and absence of ethanol	GEO	19
14	Ecoli_ASv2	GSE6925	BW25113 ymgB and W/T in LBglu 24h biofilm cells	GEO	1
15	Ecoli_ASv2	GSE6426	*E. coli* expression data from gnotobiotic ceca 14d post-inoculation	GEO	9
16	Ecoli_ASv2	GSE7398	Expression data from *E. coli* covering four phases of growth in Fis KO and WT (Fis+) strains	GEO	23
17	Ecoli_ASv2	GSE10974	Expression profiles of *E. coli* groESL(-) cells expressing human Hsp60(wt)/Hsp10 or Hsp60-(p.Val98Ile)/Hsp10 operons	GEO	1
18	Ecoli_ASv2	GSE9582	luxS-Dependent Gene Regulation in *Escherichia coli* K-12 Revealed by Genomic Expression Profiling	GEO	3
19	Ecoli_ASv2	GSE6836	Large-Scale Mapping and Validation of E. coli Transcriptional Regulation from a Compendium of Expression Profiles.	GEO	262
20	Ecoli_ASv2	GSE4511	pH Regulates Genes for Flagellar Motility, Catabolism, and Oxidative Stress in *Escherichia coli* K-12	GEO	14
21	E_coli_2	GSE7439	*Escherichia coli* strain 8624 and	GEO	4

			Escherichia coli strain VS94 with signaling molecules		
22	Ecoli_ASv2	GSE6425	Expression data from time courses of *E. coli* MG1655 and UTI89 in vitro	GEO	43
23	Ecoli_ASv2	GSE4556	pH and Anaerobiosis Coregulate Metabolism, Multidrug Transporters, and Envelope Composition in *Escherichia coli* K-12	GEO	14
24	E_coli_2	GSE9388	VS94 SAPI AI-2 Temporal study	GEO	7
25	E_coli_2	E-MEXP-732	Transcription profiling of *E. coli* cells containing shuffle network constructs | SerranoLab_Coli_ShuffleNetworks	ArrayExpress	14
26	E_coli_2	E-MEXP-584	Transcription profiling of *E. coli* 83972 grown in minimal lab media, in urine and in 3 individual patients | Roos V. *E. coli* ABU 83972	ArrayExpress	14
27	Ecoli_ASv2	E-MEXP-1001	Transcription profiling of *E. coli* F- phenocopies compared with K-12 young biofilm | F- phenocopies global gene expression compared with *E.coli* K-12 young biofilm	ArrayExpress	7
28	Ecoli_ASv2	E-MEXP-1145	Transcription profiling of pNCF (non-conjugative factor of F plasmid) carrying *E.coli* biofilm | The global gene expression of pNCF (non-conjugative factor of F plasmid) carrying *E.coli* biofilm	ArrayExpress	2
29	Ecoli_ASv2	E-TABM-103	Transcription profiling of *E. coli* in two replicate series of 8 timepoints of exponential to stationary phase planktonic cultures growing in rich media | Timecourse experiment of coli cells	ArrayExpress	15
30	E_coli_2	E-MEXP-926	Transcription profiling of two *E. coli* ABU strains during biofilm growth in human urine | Hancock V. E. coli Biofilm ABUs	ArrayExpress	17
31	Ecoli_ASv2	E-MEXP-953	Transcription profiling of an *E. coli* biofilm to assess the global gene impact of a natural F conjugative plasmid-carrying *E.coli* biofilm | The global gene impact of a natural F conjugative plasmid-ca	ArrayExpress	18
32	Ecoli_ASv2	E-MEXP-910	Transcription profiling of *E. coli* strains MG Kan N15(+), MG Kan-Tos N15(-) and MG Kan-Tos N15(+) which has a linear genome made by MG Kan N15(+) and MG Kan-Tos N15(-) | NIBB *E.coli* linear genome	ArrayExpress	2

Table Experiments for sRNAs *Escherichia coli*

[a]Experiment id: number of experiment

[b]Platform: id of platform (E_coli_2 or Ecoli_ASv2)

[c]Accession: id of experiment in database

[d]Description: description of the experiment

[e]Database: database, where experiment description placed. Can be GEO or ArrayExpress.

[f]Number of contrasts: number of contrasts in experiment
