# Supplementary material for: Inferring the relation between transcriptional and posttranscriptional regulation from expression compendia
Source: BMC Microbiol. 2014 Jan 27;14:14. doi: 10.1186/1471-2180-14-14 (PMC3948049; doi:10.1186/1471-2180-14-14)
Supplement: Additional file 6: Table S5 — Probe information. [file 1471-2180-14-14-S6.pdf]

Additional file 6 - Table 5: Probe information

[a]sRNA id	[b] platform id	[c] number of probes of sRNAs
C0067	Ecoli_ASv2	4
C0067	Ecoli_Sv2	4
C0293	Ecoli_ASv2	4
C0293	Ecoli_Sv2	5
C0299	Ecoli_ASv2	5
C0299	Ecoli_Sv2	7
C0343	Ecoli_ASv2	2
C0362	Ecoli_ASv2	6
C0362	Ecoli_Sv2	11
c0465	Ecoli_ASv2	7
C0614	Ecoli_ASv2	1
C0614	Ecoli_Sv2	4
C0664	Ecoli_ASv2	15
c0719	Ecoli_ASv2	16
c0719	Ecoli_Sv2	17
c2248_ryeB	Ecoli_ASv2	5
c2248_ryeB	Ecoli_Sv2	11
cyaR	Ecoli_ASv2	10
cyaR	Ecoli_Sv2	11
dicF	Ecoli_ASv2	14
dicF	Ecoli_Sv2	13
dsrA	Ecoli_ASv2	15
dsrA	Ecoli_Sv2	11
gadY	Ecoli_ASv2	7
gadY	Ecoli_Sv2	11
gcvB	Ecoli_ASv2	16
gcvB	Ecoli_Sv2	11
glmY	Ecoli_ASv	10
glmY	Ecoli_Sv2	11
glmZ	Ecoli_ASv2	7
glmZ	Ecoli_Sv2	11
IS128	Ecoli_ASv2	3
IS128	Ecoli_Sv2	6
isrA	Ecoli_ASv2	15
isrA	Ecoli_Sv2	11
isrB	Ecoli_ASv2	10
isrB	Ecoli_Sv2	22
isrC	Ecoli_Sv2	11
istr2	Ecoli_ASv2	5
istr2	Ecoli_Sv2	2
micC	Ecoli_ASv2	8
micC	Ecoli_Sv2	11
micF	Ecoli_ASv2	2
micF	Ecoli_Sv2	10
ohsC	Ecoli_ASv2	1
omrA	Ecoli_ASv2	3
omrA	Ecoli_Sv2	11
omrB	Ecoli_ASv2	4
omrB	Ecoli_Sv2	22

oxyS	Ecoli_ASv2	13
oxyS	Ecoli_Sv2	11
psrD	Ecoli_ASv2	22
psrD	Ecoli_Sv2	11
psrN	Ecoli_ASv2	19
psrN	Ecoli_Sv2	11
psrO	Ecoli_ASv2	23
psrO	Ecoli_Sv2	11
rdlA	Ecoli_Sv2	11
rdlB	Ecoli_Sv2	6
rdlC	Ecoli_Sv2	16
rdlD	Ecoli_ASv2	2
rdlD	Ecoli_Sv2	11
rprA	Ecoli_ASv2	5
rprA	Ecoli_Sv2	11
rpsB_tff	Ecoli_ASv2	10
rpsB_tff	Ecoli_Sv2	11
rttR	Ecoli_ASv2	21
rttR	Ecoli_Sv2	11
rttR_tpr	Ecoli_ASv2	2
rttR_tpr	Ecoli_Sv2	20
rybA	Ecoli_ASv2	5
rybA	Ecoli_Sv2	11
rybB	Ecoli_ASv2	14
rybB	Ecoli_Sv2	11
rydB	Ecoli_ASv2	2
rydB	Ecoli_Sv2	11
rydC	Ecoli_ASv2	5
rydC	Ecoli_Sv2	4
ryeA	Ecoli_ASv2	6
ryeA	Ecoli_Sv2	11
ryfA	Ecoli_ASv2	15
ryfA	Ecoli_Sv2	22
ryfB	Ecoli_ASv2	18
ryfB	Ecoli_Sv2	20
ryfD	Ecoli_ASv2	28
ryhA	Ecoli_ASv2	14
ryhA	Ecoli_Sv2	11
ryhB	Ecoli_ASv2	7
ryhB	Ecoli_Sv2	11
ryjA	Ecoli_ASv2	3
ryjA	Ecoli_Sv2	11
ryjB	Ecoli_ASv2	17
sgrS	Ecoli_ASv2	24
sgrS	Ecoli_Sv2	20
sibA	Ecoli_ASv2	6
sibA	Ecoli_Sv2	11
sibB	Ecoli_ASv2	4
sibB	Ecoli_Sv2	11
sibC	Ecoli_ASv2	8
sibC	Ecoli_Sv2	11
sibD	Ecoli_ASv2	6
sibD	Ecoli_Sv2	11

sokB	Ecoli_ASv2	1
sokB	Ecoli_Sv2	11
sokC	Ecoli_Sv2	11
spf	Ecoli_ASv2	14
spf	Ecoli_Sv2	11
sraA	Ecoli_ASv2	3
sraD	Ecoli_ASv2	8
sraD	Ecoli_Sv2	6
sroA	Ecoli_ASv2	17
sroB	Ecoli_ASv2	9
sroC	Ecoli_ASv2	30
sroD	Ecoli_ASv2	23
sroE	Ecoli_ASv2	30
sroG	Ecoli_ASv2	10
sroG	Ecoli_Sv2	8
sroH	Ecoli_ASv2	12
symR	Ecoli_ASv2	1
symR	Ecoli_Sv2	3
tp2	Ecoli_ASv2	6
tp2	Ecoli_Sv2	1
tpke11	Ecoli_ASv2	30
tpke70	Ecoli_ASv2	14
tpke70	Ecoli_Sv2	6

Note that not for all sRNAs probes are available on all platforms.

The number of probes per platform also differs.

[a]sRNA id: names of small RNAs for 87 sRNAs for which probes are available.

[b] platform id: platform

[c] number of probes of sRNAs: the number of probes on the array that can be used to estimate the expression of the sRNA (the higher the number of probes the more reliable the measurement of that sRNA). In total we had probes for 72 sRNAs.
