# Supplementary material for: Inferring the relation between transcriptional and posttranscriptional regulation from expression compendia
Source: BMC Microbiol. 2014 Jan 27;14:14. doi: 10.1186/1471-2180-14-14 (PMC3948049; doi:10.1186/1471-2180-14-14)
Supplement: Additional file 7 — Performance tests to optimize sequence-based sRNA-target predictions. [file 1471-2180-14-14-S7.pdf]

Additional file 7 - Performance tests to optimize sequence-based sRNA-target predictions

Benchmark dataset

To test the performance of the sequence-based sRNA-target predictions we compiled a subset of the initial benchmark of sRNA-target interactions for which the actual binding site had been verified or for which at least the physical binding between the sRNA and the known target had described (see Table below). This benchmark consists of 54 sRNA-target interactions.

Table: Benchmark of sRNA and corresponding targets known to physically interact

[a]sRNA ID	[b]target ID	[c]known binding site position in target	[d]Literature references
dicF	*ftsZ*	(-28,+2)	tetart_1992!abstr!
dsrA	*Hns*	(+7,+19)	lease_1998
dsrA	*rpoS*	(-126,-97)	Majdalani_1998
gcvB	*dppA*	(-43,-11)	Urbanowski_2000
gcvB	*oppA*	(-43,-11)	urbanowski_2000
micC	*ompC*	(-62,-15)	chen_2004
micF	*ompF*	(-16,-10)	andersen_1989
oxyS	*fhlA*	(34; 41)	altuvia_1998, argaman_2000
oxyS	*ybaY*	unknown	tjaden_2006
oxyS	*yobF*	unknown	tjaden_2006
oxyS	*wrbA*	unknown	tjaden_2006
oxyS	*rpoS*	unknown	liu_2005
rprA	*rpoS*	(-117,-94)	majdalani_2002
ryhB	*SdhD*	(-33,-13)	Masse_2002
ryhB	*sodB*	(-6;5)	geissmann_2004
ryhB	*shiA*	unknown	prevost_2007
ryhB	*sdhC*	unknown	Wilderman_2004
ryhB	*fur*	(-98,-53)	Vecerek_2007
ryhB	*fumA*	unknown	masse_2002
ryhB	*ftnA*	unknown	masse_2002
ryhB	*bfr*	unknown	masse_2002
ryhB	*acnA*	unknown	masse_2002
Psf (==spot42)	*galK*	(-18;14)	moller_2002
SgrS (==RyaA)	*ptsG*	(-28,-9)	vanderpool_2004
RydC	*yejABEF*	unknown	Antal_2004

RybB	*ompW*	unknown	Wassarman_2001, papenfort_2006
RybB	*ompC*	unknown	johansen_2006
RybB	*rpoE*	unknown	thompson_2007
gadY	*gadX*	unknown	opdyke_2004
sraD==MicA	*ompA*	unknown	udekwu_2005
glmY	*glmS*	unknown	urban1_2007
glmZ	*glmS*	unknown	kalamorz_2007
IstR-1	*tisAB*	unknown	vogel_2004
rseX	*ompA*	unknown	douchin_2006
rseX	*ompC*	unknown	douchin_2006
omrA and omrB(==RygA and RygB)	*cirA*	unknown	guillier_2006
omrA and omrB(==RygA and RygB)	*fecA*	unknown	guillier_2006
omrA and omrB(==RygA and RygB)	*fepA*	unknown	guillier_2006
omrA and omrB(==RygA and RygB)	*ompT*	(-12,+2)	guillier_2006
omrA and omrB(==RygA and RygB)	*gntP*	unknown	tjaden_2006
IpeX	*ompF*	unknown	castillo-keller_2005
IpeX	*ompC*	unknown	castillo-keller_2005
SymR	*SymE (==MazE)*	unknown	kawano_2007
SokB	*mok-hok*	unknown	faridani_2006
RdlD	*LdrD*	unknown	kawano_2002

[a]sRNA: ID of small RNA

[b]Target: ID of the benchmark target

[c]Known binding site position in target: this column indicates, if known the position of the interaction site between the sRNA and target, relative to the ATG (start codon) of the following gene. Otherwise is indicated unknown.

[d]Literature references: literature reference from which this binding interaction was derived

Defining thresholds on sequence-based predictions

From the benchmark defined above, it appeared that for the 15 sRNA/target interactions with known binding sites, 7 of them were located close to the ATG in a region around [-30; +20 relative to the ATG], whereas another 8 were being positioned in an interval [-150;+50 relative to the ATG]. To increase our sensitivity, we therefore choose to predict sRNA binding sites in the larger region (corresponding to the default parameters of intaRNA, and recommended by (Busch, Richter et al. 2008).

Final predictions on sRNA-target interactions were obtained by combining the predictions from either TargetRNA (Tjaden, Goodwin et al. 2006) and IntaRNA (Busch, Richter et al. 2008). Target predictions were considered selected stringently if they were in the top x list of both methods (defined as the intersection of both predictions lists). If they were only predicted in the top x list of one of the two methods, they were considered selected less stringently (defined as the union of both predictions lists). Below are summarized the sensitivity and PPV of both selection criteria on the benchmark indicated above. Using the stringent selection criteria for e.g. the top 25 list approximately 12% of the known targets for sRNAs could be retrieved with an PPV of 19% whereas with the non-stringent criteria these numbers are respectively 18% for the sensitivity and 1% for the PPV.

Table Sequence-based sRNA-target predictions of the benchmark

To decide on a threshold on the number of predictions per sRNA we performed a sensitivity analysis on the benchmark defined above (see table below). We defined for each of the known sRNA-target interactions whether they could be recovered in either the top5, top 10 or to 25 predictionsby respectively TargetRNA or IntaRNA using the parameters defined above.

Table: Sequence-based sRNA-target predictions of the benchmark

Performance measures	[a]TargetRNA OR IntaRNA [-150;50] seed 8 nt Top25	[b]TargetRNA AND IntaRNA [-150;50] seed 8 nt Top25	[c]TargetRNA OR IntaRNA [-150;50] seed 8 nt Top10	[d]TargetRNA AND IntaRNA [-150;50] seed 8 nt Top10	[e]TargetRNA OR IntaRNA [-150;50] seed 8 nt Top5	[f]TargetRNA AND IntaRN[illegible] [-150;50] seed 8 nt Top5
**Sensitivity**	**18%**	**12%**	**17%**	**10%**	**13%**	**8%**
**PPV**	**1%**	**19%**	**3%**	**23%**	**5%**	**33%**

Sensitivity: indicates the number of known interactions retrieved by combining the results of respectively TargetRNA and IntaRNA on the total number of interactions in the benchmark (benchmark displayed in Table above).

PPV: the PPV is approximated by taking the ratio of the number of predicted interactions present in the benchmark on the total number of predictions retrieved by combining the results of TargetRNA and IntaRNA.

a) Predictions are defined as the union of the top 25 predictions obtained by respectively TargetRNA and IntaRNA (using the indicated parameter settings for both algorithms)

b) Predictions are defined as the intersection of the top 25 predictions obtained by respectively TargetRNA and IntaRNA (using the indicated parameter settings for both algorithms)

c) Predictions are defined as the union of the top 10 predictions obtained by respectively TargetRNA and IntaRNA (using the indicated parameter settings for both algorithms)

d) Predictions are defined as the intersection of the top 10 predictions obtained by respectively TargetRNA and IntaRNA (using the indicated parameter settings for both algorithms)

e) Predictions are defined as the union of the top 5 predictions obtained by respectively TargetRNA and IntaRNA (using the indicated parameter settings for both algorithms)

f) Predictions are defined as the intersection of the top 5 predictions obtained by respectively TargetRNA and IntaRNA (using the indicated parameter settings for both algorithms)

Final sRNA-target predictions

The final sequence-based predictions on sRNA-target interactions made by taking the union of the top 25 predictions made by either TargetRNA or IntaRNA using a seed of 8 NT and searching for recognition sites in a the region located at -150 till +50 relative to the ATG in the target) are listed below (copy here the updated table from Supplementary file: Predictions of sRNA targets based on intaRNA (Busch, Richter et al. 2008) and targetRNA (Tjaden, Goodwin et al. 2006).

References

Busch, A., A. S. Richter and R. Backofen (2008). "IntaRNA: efficient prediction of bacterial sRNA targets incorporating target site accessibility and seed regions." Bioinformatics **24**(24): 2849-2856.

Tjaden, B., S. S. Goodwin, J. A. Opdyke, M. Guillier, D. X. Fu, S. Gottesman and G. Storz (2006). "Target prediction for small, noncoding RNAs in bacteria." Nucleic Acids Res **34**(9): 2791-2802.
