# Supplementary material for: Inferring the relation between transcriptional and posttranscriptional regulation from expression compendia
Source: BMC Microbiol. 2014 Jan 27;14:14. doi: 10.1186/1471-2180-14-14 (PMC3948049; doi:10.1186/1471-2180-14-14)
Supplement: Additional file 8: Table S6 — Predictions of sRNA targets based on intaRNA and TargetRNA. [file 1471-2180-14-14-S8.pdf]

Additional file 8 - Table 6: Predictions of sRNA targets based on intaRNA and targetRNA

		Predictions made by targetRNA				Predictions made by intaRNA		
[a]sRNA ID	[b]predictions rank	[c]target name (targetRNA)	[d]target b-number (targetRNA)	[e]score (targetRNA)	[f]p-value (targetRNA)	[g]target name (intaRNA)	[h]target b-number (intaRNA)	[i]score (intaRNA)
Spf	1	*goaG*	b1302	-103	4.49665e-05	*wrbA*	b1004	-14,76
Spf	2	*galK*	b0757	-92	0.000250851	*amyA*	b1927	-13,36
Spf	3	*recE*	b1350	-84	0.000875473	*prmC*	b1212	-12,88
Spf	4	*sucD*	b0729	-80	0.00163515	*fucP*	b2801	-12,37
Spf	5	*cirA*	b2155	-80	0.00163515	*yiaO*	b3579	-12,23
Spf	6	*nmpC*	b0553	-79	0.00191147	*bacA*	b3057	-12,13
Spf	7	*ymfL*	b1147	-76	0.00305303	*yheM*	b3344	-11,8
Spf	8	*ygjM*	b3082	-76	0.00305303	*nanT*	b3224	-11,73
Spf	9	*ydjH*	b1772	-75	0.00356853	*glpF*	b3927	-11,72
Spf	10	*ycdC*	b1013	-74	0.00417088	*ybfQ*	b4514	-11,7
Spf	11	*ygbF*	b2754	-73	0.00487466	*puuE*	b1302	-11,68
Spf	12	*rfaY*	b3625	-73	0.00487466	*tsx*	b0411	-11,51
Spf	13	*ppc*	b3956	-73	0.00487466	*aat*	b0885	-11,49
Spf	14	*hemK*	b1212	-72	0.00569686	*ybiX*	b0804	-11,45
Spf	15	*yfgI*	b2506	-71	0.00665727	*hyfD*	b2484	-11,39
Spf	16	*tsx*	b0411	-70	0.00777896	*paaK*	b1398	-11,37
Spf	17	*accC*	b3256	-70	0.00777896	*galK*	b0757	-11,32
Spf	18	*fkpA*	b3347	-69	0.00908877	*ybbW*	b0511	-11,28
Spf	19	*miaA*	b4171	-69	0.00908877	*yfeK*	b2419	-11,22
Spf	20	*hlyE*	b1182	-68	0.0106179	*rpmC*	b3312	-11,16
Spf	21	*ycjN*	b1310	-68	0.0106179	*rdlA*	b4420	-11,15
Spf	22	*yggX*	b2962	-68	0.0106179	*glyA*	b2551	-10,89
Spf	23	*caiA*	b0039	-67	0.0124028	*aspA*	b4139	-10,72
Spf	24	*rluC*	b1086	-67	0.0124028	*cysC*	b2750	-10,68
Spf	25	*yehI*	b2118	-67	0.0124028	*mdh*	b3236	-10,62
MicF	1	*metN*	b0199	-96	0.00010013	*ycbR*	b0939	-18,14
MicF	2	*yciF*	b1258	-87	0.000420023	*hsdS*	b4348	-16,66
MicF	3	*sfmC*	b0531	-84	0.000677343	*yffQ*	b2448	-15,93
MicF	4	*oppA*	b1243	-81	0.00109222	*yciY*	b4595	-14,31
MicF	5	*hyi*	b0508	-80	0.00128076	*ybiH*	b0796	-13,92
MicF	6	*ompF*	b0929	-80	0.00128076	*pyrI*	b4244	-13,32
MicF	7	*yfcN*	b2331	-79	0.00150181	*sbcB*	b2011	-13,29
MicF	8	*zur*	b4046	-79	0.00150181	*yaeP*	b4406	-13,05
MicF	9	*yggU*	b2953	-78	0.00176099	*hypB*	b2727	-12,97
MicF	10	*pyrI*	b4244	-78	0.00176099	*lrp*	b0889	-12,88
MicF	11	*mcrA*	b1159	-77	0.00206485	*metN*	b0199	-12,78
MicF	12	*tdcR*	b3119	-77	0.00206485	*baeR*	b2079	-12,56

MicF	13	*yhbS*	b3156	-75	0.00283867	*yfcN*	b2331	-12,22
MicF	14	*sfmA*	b0530	-74	0.00332817	*metL*	b3940	-12,05
MicF	15	*spy*	b1743	-74	0.00332817	*insH*	b0259	-11,69
MicF	16	*relE*	b1563	-73	0.00390191	*rng*	b3247	-11,66
MicF	17	*rfbC*	b2038	-73	0.00390191	*yadH*	b0128	-11,44
MicF	18	*wza*	b2062	-73	0.00390191	*dgoR*	b4479	-11,43
MicF	19	*yaeP*	b4406	-73	0.00390191	*omrB*	b4445	-11,4
MicF	20	*sbcB*	b2011	-72	0.00457434	*yhcF*	b3219	-11,33
MicF	21	*yeeY*	b2015	-72	0.00457434	*argP*	b2916	-11,27
MicF	22	*yegX*	b2102	-72	0.00457434	*fliY*	b1920	-11,13
MicF	23	*gabT*	b2662	-71	0.00536234	*yigA*	b3810	-11,06
MicF	24	*yqhD*	b3011	-71	0.00536234	*proV*	b2677	-10,96
MicF	25	*ygiW*	b3024	-71	0.00536234	*atpB*	b3738	-10,93
DicF	1	*ygcQ*	b2769	-103	2.3506e-05	*nrfF*	b4075	-15,19
DicF	2	*sieB*	b1353	-81	0.000840077	*mtr*	b3161	-14,59
DicF	3	*rpmJ*	b3299	-81	0.000840077	*yrhC*	b4552	-13,88
DicF	4	*cusC*	b0572	-77	0.00160906	*puuC*	b1300	-13,27
DicF	5	*yhjD*	b3522	-75	0.00222664	*cyoC*	b0430	-13
DicF	6	*nuoL*	b2278	-74	0.0026192	*yciY*	b4595	-12,81
DicF	7	*ubiG*	b2232	-72	0.00362377	*nuoL*	b2278	-12,34
DicF	8	*ybcX*	b0561	-71	0.00426214	*yjcZ*	b4110	-12,32
DicF	9	*stfQ*	b1547	-71	0.00426214	*ylbA*	b0515	-12,31
DicF	10	*yfjT*	b2637	-71	0.00426214	*ycfD*	b1128	-12,17
DicF	11	*yjeQ*	b4161	-70	0.00501267	*ampE*	b0111	-12,12
DicF	12	*sms*	b4389	-70	0.00501267	*ycjT*	b1316	-11,82
DicF	13	*rna*	b0611	-69	0.00589498	*nrfC*	b4072	-11,81
DicF	14	*rnb*	b1286	-69	0.00589498	*pykA*	b1854	-11,73
DicF	15	*xisR*	b1346	-69	0.00589498	*umuD*	b1183	-11,66
DicF	16	*ykgG*	b0308	-68	0.00693204	*rlmN*	b2517	-11,66
DicF	17	*rnk*	b0610	-68	0.00693204	*tauB*	b0366	-11,59
DicF	18	*ynfD*	b1586	-68	0.00693204	*ybiT*	b0820	-11,56
DicF	19	*ddg*	b2378	-68	0.00693204	*ygbI*	b2735	-11,55
DicF	20	*ppk*	b2501	-68	0.00693204	*yjgB*	b4269	-11,51
DicF	21	*recA*	b2699	-68	0.00693204	*ydcN*	b1434	-11,46
DicF	22	*ybfN*	b0682	-67	0.00815079	*phoP*	b1130	-11,4
DicF	23	*yadF*	b0126	-66	0.00958279	*ubiF*	b0662	-11,37
DicF	24	*mtr*	b3161	-66	0.00958279	*ygcQ*	b2769	-11,3
DicF	25	*carB*	b0033	-65	0.0112649	*gsiB*	b0830	-11,26
RpsB_tff	1	*ydfG*	b1539	-90	0.000743311	*metK*	b2942	-13,31
RpsB_tff	2	*infA*	b0884	-83	0.00208846	*rtcB*	b3421	-12,95
RpsB_tff	3	*ylbA*	b0515	-82	0.00242042	*iscR*	b2531	-12,49
RpsB_tff	4	*hokD*	b1562	-77	0.00505817	*yrbL*	b3207	-12,02
RpsB_tff	5	*perR*	b0254	-76	0.00586076	*yfjG*	b2619	-11,93

RpsB_tff	6	*pepT*	b1127	-76	0.00586076	*insH*	b0259	-11,82
RpsB_tff	7	*abrB*	b0715	-75	0.00679026	*nadK*	b2615	-11,73
RpsB_tff	8	*ispF*	b2746	-75	0.00679026	*arnD*	b2256	-11,58
RpsB_tff	9	*yfjK*	b2627	-74	0.0078666	*pta*	b2297	-11,53
RpsB_tff	10	*ygaQ*	b2654	-74	0.0078666	*yegR*	b2085	-11,53
RpsB_tff	11	*nrfG*	b4076	-74	0.0078666	*puuE*	b1302	-11,46
RpsB_tff	12	*argI*	b4254	-74	0.0078666	*yfjK*	b2627	-11,23
RpsB_tff	13	*potI*	b0857	-73	0.00911276	*torS*	b0993	-11,2
RpsB_tff	14	*rpsB*	b0169	-72	0.0105553	*dacB*	b3182	-11,2
RpsB_tff	15	*yfaX*	b2248	-72	0.0105553	*yciB*	b1254	-11,14
RpsB_tff	16	*ynaF*	b1376	-71	0.0122247	*ypeC*	b2390	-10,98
RpsB_tff	17	*hyfD*	b2484	-71	0.0122247	*aidB*	b4187	-10,94
RpsB_tff	18	*ybdQ*	b0607	-70	0.0141563	*sodB*	b1656	-10,71
RpsB_tff	19	*yraM*	b3147	-70	0.0141563	*yhhS*	b3473	-10,68
RpsB_tff	20	*phnG*	b4101	-70	0.0141563	*uvrC*	b1913	-10,64
RpsB_tff	21	*gatZ*	b2095	-69	0.0163906	*fliA*	b1922	-10,53
RpsB_tff	22	*ygiY*	b3026	-69	0.0163906	*apaH*	b0049	-10,43
RpsB_tff	23	*yaaY*	b0024	-68	0.0189741	*ygjK*	b3080	-10,43
RpsB_tff	24	*rfbX*	b2037	-68	0.0189741	*pheT*	b1713	-10,33
RpsB_tff	25	*yfaE*	b2236	-68	0.0189741	*glgX*	b3431	-10,24
RprA	1	*sieB*	b1353	-106	2.87671e-05	*hemH*	b0475	-19,16
RprA	2	*pin*	b1158	-92	0.000255717	*csgD*	b1040	-17,47
RprA	3	*hemH*	b0475	-86	0.000652156	*pal*	b0741	-17,38
RprA	4	*creA*	b4397	-83	0.00104137	*insH*	b0259	-16,4
RprA	5	*ydfI*	b1542	-82	0.00121716	*ymgC*	b1167	-16,38
RprA	6	*yddL*	b1472	-80	0.00166268	*purH*	b4006	-16,28
RprA	7	*glnS*	b0680	-78	0.0022711	*rph*	b3643	-16,1
RprA	8	*csgA*	b1042	-77	0.00265419	*purT*	b1849	-15,63
RprA	9	*yhcR*	b3242	-76	0.0031018	*glnS*	b0680	-15,53
RprA	10	*yadE*	b0130	-75	0.00362476	*yihN*	b3874	-15,12
RprA	11	*xylA*	b3565	-75	0.00362476	*ytfN*	b4221	-14,76
RprA	12	*yabF*	b0046	-74	0.00423571	*lplT*	b2835	-14,32
RprA	13	*yeaU*	b1800	-74	0.00423571	*ddpA*	b1487	-14,16
RprA	14	*ygdI*	b2809	-74	0.00423571	*ddpX*	b1488	-13,91
RprA	15	*hdeD*	b3511	-73	0.00494937	*ybaZ*	b0454	-13,79
RprA	16	*mtlR*	b3601	-72	0.00578292	*flgL*	b1083	-13,67
RprA	17	*miaA*	b4171	-72	0.00578292	*yadL*	b0137	-13,54
RprA	18	*ycbQ*	b0938	-71	0.00675639	*aroE*	b3281	-13,35
RprA	19	*ddpX*	b1488	-71	0.00675639	*fecR*	b4292	-13,2
RprA	20	*flgM*	b1071	-70	0.00789306	*rplQ*	b3294	-13,17
RprA	21	*yddS*	b1487	-70	0.00789306	*galP*	b2943	-13,16
RprA	22	*yiaW*	b3587	-70	0.00789306	*fliS*	b1925	-13,13
RprA	23	*argF*	b0273	-69	0.00922009	*livK*	b3458	-13,12
RprA	24	*csgD*	b1040	-69	0.00922009	*galF*	b2042	-12,76

RprA	25	*ymgC*	b1167	-69	0.00922009	*pinE*	b1158	-12,72
RttR_tpr	1	*ybdF*	b0579	-55	0.0163546	*cysI*	b2763	-19,06
RttR_tpr	2	*ycfS*	b1113	-54	0.0196514	*uidB*	b1616	-18,75
RttR_tpr	3	*yjeT*	b4176	-54	0.0196514	*yqhA*	b3002	-17,97
RttR_tpr	4	*yliH*	b0836	-53	0.0236046	*yeiW*	b4502	-16,94
RttR_tpr	5	*pfkB*	b1723	-53	0.0236046	*psiE*	b4030	-16,88
RttR_tpr	6	*yqhA*	b3002	-53	0.0236046	*hemG*	b3850	-16,63
RttR_tpr	7	*ppk*	b2501	-52	0.0283416	*yibQ*	b3614	-16,38
RttR_tpr	8	*yibQ*	b3614	-52	0.0283416	*yghB*	b3009	-16,36
RttR_tpr	9	*yhiI*	b3487	-51	0.0340124	*ycjP*	b1312	-16,33
RttR_tpr	10	*gloB*	b0212	-50	0.040794	*cysP*	b2425	-16,28
RttR_tpr	11	*ybfM*	b0681	-50	0.040794	*eutM*	b2457	-16,28
RttR_tpr	12	*ybgE*	b0735	-50	0.040794	*ytcA*	b4622	-15,92
RttR_tpr	13	*moaE*	b0785	-50	0.040794	*ydiZ*	b1724	-15,8
RttR_tpr	14	*ybhN*	b0788	-50	0.040794	*ychF*	b1203	-15,76
RttR_tpr	15	*gst*	b1635	-50	0.040794	*aas*	b2836	-15,69
RttR_tpr	16	*fliQ*	b1949	-50	0.040794	*hemB*	b0369	-15,58
RttR_tpr	17	*napF*	b2208	-50	0.040794	*priA*	b3935	-15,57
RttR_tpr	18	*ubiH*	b2907	-50	0.040794	*yheS*	b3352	-15,53
RttR_tpr	19	*arsC*	b3503	-50	0.040794	*yfgH*	b2505	-15,5
RttR_tpr	20	*yidH*	b3676	-50	0.040794	*udk*	b2066	-15,48
RttR_tpr	21	*yiiL*	b3901	-50	0.040794	*leuL*	b0075	-15,35
RttR_tpr	22	*leuL*	b0075	-49	0.0488931	*yjeT*	b4176	-15,28
RttR_tpr	23	*fhuB*	b0153	-49	0.0488931	*arnA*	b2255	-15,26
RttR_tpr	24	*yliD*	b0832	-49	0.0488931	*flgH*	b1079	-15,19
RttR_tpr	25	*ycbC*	b0920	-49	0.0488931	*gltI*	b0655	-15,18
DsrA	1	*rpoS*	b2741	-87	0.000372436	*ybcM*	b0546	-17,01
DsrA	2	*ycjX*	b1321	-85	0.00051359	*dcuS*	b4125	-15,19
DsrA	3	*argR*	b3237	-85	0.00051359	*ycjN*	b1310	-14,81
DsrA	4	*ybeB*	b0637	-83	0.000708222	*cbl*	b1987	-14,46
DsrA	5	*cybB*	b1418	-83	0.000708222	*gspB*	b3322	-13,74
DsrA	6	*yfiE*	b2577	-82	0.000831649	*ylbH*	b0499	-13,38
DsrA	7	*yhcD*	b3216	-81	0.000976577	*ilvH*	b0078	-13,16
DsrA	8	*greB*	b3406	-80	0.00114675	*argR*	b3237	-12,91
DsrA	9	*uxuR*	b4324	-80	0.00114675	*ybeB*	b0637	-12,79
DsrA	10	*eaeH*	b0297	-77	0.00185655	*nhoA*	b1463	-12,73
DsrA	11	*ileS*	b0026	-76	0.00217988	*purN*	b2500	-12,65
DsrA	12	*ygeH*	b2852	-76	0.00217988	*entB*	b0595	-12,56
DsrA	13	*yieK*	b3718	-75	0.00255946	*ydjI*	b1773	-12,41
DsrA	14	*rbsA*	b3749	-75	0.00255946	*mrp*	b2113	-12,4
DsrA	15	*tra8_3*	b4284	-74	0.00300503	*nudG*	b1759	-12,37
DsrA	16	*ybhI*	b0770	-72	0.00414187	*mobA*	b3857	-12,27
DsrA	17	*lysA*	b2838	-72	0.00414187	*ybjI*	b0844	-12,26

DsrA	18	*ilvI*	b0077	-70	0.00570756	*citT*	b0612	-12,25
DsrA	19	*csgF*	b1038	-70	0.00570756	*qseB*	b3025	-12,09
DsrA	20	*hns*	b1237	-70	0.00570756	*rfbD*	b2040	-11,93
DsrA	21	*yeaA*	b1778	-70	0.00570756	*ddpC*	b1485	-11,87
DsrA	22	*yi22_1*	b0361	-69	0.00669934	*metY*	b3171	-11,83
DsrA	23	*yi22_2*	b1402	-69	0.00669934	*valW*	b1666	-11,8
DsrA	24	*yi22_3*	b1996	-69	0.00669934	*yigA*	b3810	-11,74
DsrA	25	*yi22_4*	b2860	-69	0.00669934	*ybcO*	b0549	-11,73
OxyS	1	*oxyR*	b3961	-275	2.22045e-16	*oxyR*	b3961	-64,59
OxyS	2	*yobF*	b1824	-94	0.000231877	*ydbJ*	b4529	-18,41
OxyS	3	*wbbJ*	b2033	-94	0.000231877	*frwD*	b3953	-15,07
OxyS	4	*yfdH*	b2351	-86	0.000793329	*wcaD*	b2056	-14,53
OxyS	5	*mltB*	b2701	-80	0.00199494	*yfbO*	b2274	-14,41
OxyS	6	*rpoC*	b3988	-78	0.00271239	*spr*	b2175	-14,32
OxyS	7	*trpE*	b1264	-76	0.0036874	*wzc*	b2060	-14,21
OxyS	8	*nagC*	b0676	-74	0.00501199	*csgC*	b1043	-13,89
OxyS	9	*mltC*	b2963	-74	0.00501199	*cadC*	b4133	-13,67
OxyS	10	b3000	b3000	-71	0.00793853	*tyrA*	b2600	-13,41
OxyS	11	*frwD*	b3953	-71	0.00793853	*mltB*	b2701	-13,3
OxyS	12	*ycfR*	b1112	-70	0.00925214	*clpA*	b0882	-13,27
OxyS	13	*fabG*	b1093	-69	0.0107819	*yfiQ*	b2584	-13,2
OxyS	14	*csiD*	b2659	-68	0.0125631	*emrY*	b2367	-13,17
OxyS	15	*xisR*	b1346	-67	0.0146363	*ydhF*	b1647	-13,12
OxyS	16	*napG*	b2205	-66	0.0170486	*yggD*	b2929	-12,86
OxyS	17	*sgcQ*	b4303	-65	0.0198545	*secE*	b3981	-12,74
OxyS	18	*yahJ*	b0324	-64	0.0231168	*inaA*	b2237	-12,72
OxyS	19	*yajO*	b0419	-64	0.0231168	*rimK*	b0852	-12,37
OxyS	20	*ymgD*	b1171	-64	0.0231168	*fabG*	b1093	-12,31
OxyS	21	*hypD*	b2729	-64	0.0231168	*ynjE*	b1757	-11,91
OxyS	22	*yheU*	b3354	-64	0.0231168	*yeeZ*	b2016	-11,85
OxyS	23	*yjeJ*	b4145	-64	0.0231168	*tfaX*	b0563	-11,85
OxyS	24	*ldrB*	b4421	-64	0.0231168	*hisM*	b2307	-11,75
OxyS	25	*ldrC*	b4423	-64	0.0231168	*ttdA*	b3061	-11,6
C3046_RyfA	1	*yjeS*	b4166	-90	0.000349381	*kefC*	b0047	-15,78
C3046_RyfA	2	*nrdH*	b2673	-88	0.000477343	*gcvT*	b2905	-14,87
C3046_RyfA	3	*tmk*	b1098	-84	0.000890961	*cstA*	b0598	-14,77
C3046_RyfA	4	*ybbA*	b0495	-78	0.0022711	*dsbC*	b2893	-14,74
C3046_RyfA	5	*purC*	b2476	-76	0.0031018	*flhC*	b1891	-13,92
C3046_RyfA	6	*fic*	b3361	-73	0.00494937	*ygiW*	b3024	-13,55
C3046_RyfA	7	*yiiE*	b3889	-73	0.00494937	*dcuC*	b0621	-13,37
C3046_RyfA	8	*ybgA*	b0707	-72	0.00578292	*lit*	b1139	-13,14
C3046_RyfA	9	*hyaC*	b0974	-72	0.00578292	*ybbA*	b0495	-12,78
C3046_RyfA	10	*djlA*	b0055	-71	0.00675639	*yrdE*	b4646	-12,78

C3046_RyfA	11	*yliF*	b0834	-71	0.00675639	*yqiA*	b3031	-12,73
C3046_RyfA	12	*ccmH*	b2194	-71	0.00675639	*yjeS*	b4166	-12,67
C3046_RyfA	13	*ptrB*	b1845	-70	0.00789306	*ychJ*	b1233	-12,61
C3046_RyfA	14	*araG*	b1900	-70	0.00789306	*tus*	b1610	-12,52
C3046_RyfA	15	*hyfF*	b2486	-70	0.00789306	*citT*	b0612	-12,5
C3046_RyfA	16	*kgtP*	b2587	-70	0.00789306	*eutP*	b2461	-12,42
C3046_RyfA	17	*cmtA*	b2933	-70	0.00789306	*yfcQ*	b2334	-12,38
C3046_RyfA	18	*acpT*	b3475	-70	0.00789306	*glnP*	b0810	-12,27
C3046_RyfA	19	*yhiO*	b3494	-70	0.00789306	*insC*	b0360	-12,17
C3046_RyfA	20	*coaA*	b3974	-70	0.00789306	*guaA*	b2507	-12,15
C3046_RyfA	21	*gltA*	b0720	-69	0.00922009	*ccmH*	b2194	-12,14
C3046_RyfA	22	*torT*	b0994	-68	0.010769	*yacF*	b0102	-12,08
C3046_RyfA	23	*ymfS*	b1155	-68	0.010769	*fecC*	b4289	-11,99
C3046_RyfA	24	*ydaU*	b1359	-68	0.010769	*purL*	b2557	-11,87
C3046_RyfA	25	*yefM*	b2017	-68	0.010769	*ybgO*	b0716	-11,69
CyaR	1	*ydeR*	b1503	-78	0.00154537	*nrfE*	b4074	-18,51
CyaR	2	*ecpD*	b0140	-68	0.00770797	*hsdR*	b4350	-18,01
CyaR	3	*nrfE*	b4074	-68	0.00770797	*metQ*	b0197	-17,18
CyaR	4	*dps*	b0812	-67	0.00904841	*ydcX*	b1445	-16,11
CyaR	5	*rfbA*	b2039	-66	0.0106207	*bolA*	b0435	-15,88
CyaR	6	*yfcY*	b2342	-64	0.014626	*ydeR*	b1503	-15,84
CyaR	7	*guaB*	b2508	-64	0.014626	*cbl*	b1987	-15,77
CyaR	8	*ykgM*	b0296	-63	0.017159	*lolB*	b1209	-15,73
CyaR	9	*yffR*	b2449	-63	0.017159	*yciY*	b4595	-15,22
CyaR	10	*hypD*	b2729	-63	0.017159	*ompX*	b0814	-15,15
CyaR	11	*ycbZ*	b0955	-62	0.0201262	*ddpC*	b1485	-15,12
CyaR	12	*ykfF*	b0249	-61	0.0236004	*yohK*	b2142	-15,08
CyaR	13	*intR*	b1345	-61	0.0236004	*dppB*	b3543	-14,8
CyaR	14	*intQ*	b1579	-61	0.0236004	*cobS*	b1992	-14,58
CyaR	15	*hisQ*	b2308	-61	0.0236004	*hisQ*	b2308	-14,58
CyaR	16	b2859	b2859	-61	0.0236004	*motA*	b1890	-14,57
CyaR	17	*yqiG*	b3046	-61	0.0236004	*mobB*	b3856	-14,44
CyaR	18	*ispB*	b3187	-61	0.0236004	*yacC*	b0122	-14,4
CyaR	19	*eutE*	b2455	-60	0.0276658	*mdtI*	b1599	-14,16
CyaR	20	*kdpA*	b0698	-59	0.0324197	*wcaD*	b2056	-14,11
CyaR	21	*ymfI*	b1143	-59	0.0324197	*idnK*	b4268	-14,08
CyaR	22	*yjgW*	b4274	-59	0.0324197	*birA*	b3973	-13,94
CyaR	23	*yncH*	b1455	-58	0.0379744	*trkG*	b1363	-13,72
CyaR	24	*glf*	b2036	-58	0.0379744	*yfbP*	b2275	-13,7
CyaR	25	*yehE*	b2112	-58	0.0379744	*ymgC*	b1167	-13,64
GadY	1	*yhbV*	b3159	-89	0.000415923	*cueR*	b0487	-16,22
GadY	2	*thrS*	b1719	-87	0.000568016	*malE*	b4034	-15,97
GadY	3	*cbpA*	b1000	-83	0.00105929	*bssR*	b0836	-14,93

GadY	4	*rfaB*	b3628	-83	0.00105929	*mscS*	b2924	-14,87
GadY	5	*poxB*	b0871	-74	0.00430052	*xdhC*	b2868	-14,17
GadY	6	*ssuC*	b0934	-72	0.00586893	*ypdJ*	b4545	-14,02
GadY	7	*kdsB*	b0918	-70	0.00800705	*yieP*	b3755	-13,81
GadY	8	*ycdO*	b1018	-70	0.00800705	*cpxP*	b4484	-13,8
GadY	9	b2651	b2651	-70	0.00800705	*chbA*	b1736	-13,51
GadY	10	*dkgA*	b3012	-69	0.00935123	*gfcD*	b0984	-13,43
GadY	11	*yhjR*	b3535	-69	0.00935123	*proL*	b2189	-13,35
GadY	12	*yhdX*	b3269	-67	0.0127498	*visC*	b2906	-13,2
GadY	13	*sodB*	b1656	-66	0.0148842	*cbpA*	b1000	-13,18
GadY	14	*rnhB*	b0183	-65	0.0173727	*flgL*	b1083	-13,16
GadY	15	*sdhC*	b0721	-64	0.020273	*ycbW*	b0946	-12,85
GadY	16	*ycfC*	b1132	-64	0.020273	*yiaN*	b3578	-12,72
GadY	17	*yfdF*	b2345	-64	0.020273	*fliE*	b1937	-12,71
GadY	18	*talA*	b2464	-64	0.020273	*yzgL*	b3427	-12,65
GadY	19	*exuR*	b3094	-64	0.020273	*glnP*	b0810	-12,47
GadY	20	*yiiD*	b3888	-64	0.020273	*ydiA*	b1703	-12,45
GadY	21	*ecpD*	b0140	-63	0.0236516	*wcaC*	b2057	-12,42
GadY	22	*narJ*	b1226	-63	0.0236516	*ygiW*	b3024	-12,29
GadY	23	*cheW*	b1887	-63	0.0236516	*narV*	b1465	-12,28
GadY	24	*yfeK*	b2419	-63	0.0236516	*fadR*	b1187	-12,18
GadY	25	*dapB*	b0031	-62	0.0275854	*mpaA*	b1326	-12,13
GlmZ	1	*yfhA*	b2554	-90	0.000945831	*grpE*	b2614	-16
GlmZ	2	*nanK*	b3222	-90	0.000945831	*pgk*	b2926	-15,71
GlmZ	3	*yabB*	b0081	-89	0.00109334	*phoE*	b0241	-15,33
GlmZ	4	*yegX*	b2102	-86	0.00168866	*scpC*	b2920	-14,86
GlmZ	5	*yfgA*	b2516	-85	0.0019519	*ftnB*	b1902	-14,5
GlmZ	6	*ycjD*	b1289	-84	0.00225612	*infC*	b1718	-14,28
GlmZ	7	*hslU*	b3931	-84	0.00225612	*yafS*	b0213	-13,67
GlmZ	8	*yccX*	b0968	-81	0.00348348	*elaA*	b2267	-13,63
GlmZ	9	*ygjP*	b3085	-80	0.00402594	*ytjB*	b4387	-13,6
GlmZ	10	*ynhG*	b1678	-79	0.00465268	*pdxK*	b2418	-13,41
GlmZ	11	*ypdF*	b2385	-79	0.00465268	*yhjQ*	b3534	-13,4
GlmZ	12	*yhiO*	b3494	-79	0.00465268	*yggD*	b2929	-13,26
GlmZ	13	*argT*	b2310	-78	0.00537672	*ugpQ*	b3449	-13,09
GlmZ	14	*xerD*	b2894	-77	0.00621308	*insA*	b0022	-12,9
GlmZ	15	*ycgJ*	b1177	-76	0.00717908	*ydaN*	b1342	-12,78
GlmZ	16	*oppB*	b1244	-76	0.00717908	*pyrI*	b4244	-12,77
GlmZ	17	*ydhV*	b1673	-76	0.00717908	*insA*	b0022	-12,67
GlmZ	18	*ygeW*	b2870	-76	0.00717908	*aroE*	b3281	-12,64
GlmZ	19	*ruvC*	b1863	-75	0.00829463	*mmuM*	b0261	-12,62
GlmZ	20	*yeeA*	b2008	-75	0.00829463	*ysaC*	b4648	-12,61
GlmZ	21	*yraR*	b3152	-75	0.00829463	*hcaR*	b2537	-12,59
GlmZ	22	*yigF*	b3817	-75	0.00829463	*ycgB*	b1188	-12,56

GlmZ	23	*nfi*	b3998	-75	0.00829463	*renD*	b0542	-12,55
GlmZ	24	*yjgX*	b4275	-75	0.00829463	*glgA*	b3429	-12,53
GlmZ	25	*era*	b2566	-74	0.0095827	*rraB*	b4255	-12,36
C2248_RyeB	1	*potE*	b0692	-94	0.000183523	*ilvL*	b3766	-12,7
C2248_RyeB	2	*acrE*	b3265	-90	0.000342876	*ilvB*	b3671	-10,64
C2248_RyeB	3	*yeeT*	b2003	-84	0.000875473	*ynfH*	b1590	-10,29
C2248_RyeB	4	*ybaL*	b0478	-81	0.00139875	*yajR*	b0427	-10,04
C2248_RyeB	5	*yeeD*	b2012	-81	0.00139875	*hybG*	b2990	-9,88
C2248_RyeB	6	*ygdH*	b2795	-81	0.00139875	*yhcF*	b3219	-9,86
C2248_RyeB	7	*ypdA*	b2380	-79	0.00191147	*ybjI*	b0844	-9,71
C2248_RyeB	8	*ydhP*	b1657	-77	0.0026119	*ompG*	b1319	-9,59
C2248_RyeB	9	*yciS*	b1279	-76	0.00305303	*rpsC*	b3314	-9,5
C2248_RyeB	10	*csgD*	b1040	-75	0.00356853	*yncI*	b1459	-9,47
C2248_RyeB	11	*bglG*	b3723	-75	0.00356853	*mutL*	b4170	-9,45
C2248_RyeB	12	*yhcF*	b3219	-74	0.00417088	*chpS*	b4224	-9,31
C2248_RyeB	13	*pheS*	b1714	-73	0.00487466	*ycbB*	b0925	-9,14
C2248_RyeB	14	*perM*	b2493	-73	0.00487466	*mdtA*	b2074	-9,04
C2248_RyeB	15	*yrbC*	b3192	-72	0.00569686	*yrhC*	b4552	-9,02
C2248_RyeB	16	*purA*	b4177	-72	0.00569686	*yeeD*	b2012	-8,99
C2248_RyeB	17	*rpsD*	b3296	-71	0.00665727	*ascG*	b2714	-8,97
C2248_RyeB	18	*yeeI*	b1976	-70	0.00777896	*paaF*	b1393	-8,94
C2248_RyeB	19	*rpmD*	b3302	-70	0.00777896	*ynhF*	b4602	-8,9
C2248_RyeB	20	*yjfK*	b4183	-70	0.00777896	*rutR*	b1013	-8,76
C2248_RyeB	21	*ybgR*	b0752	-69	0.00908877	*yicJ*	b3657	-8,75
C2248_RyeB	22	*hyaD*	b0975	-69	0.00908877	*ftsB*	b2748	-8,71
C2248_RyeB	23	*nrfA*	b4070	-69	0.00908877	*yhfW*	b3380	-8,62
C2248_RyeB	24	*dcuR*	b4124	-69	0.00908877	*mdtO*	b4081	-8,61
C2248_RyeB	25	*yecH*	b1906	-68	0.0106179	*ydeP*	b1501	-8,47
PsrO	1	*pnp*	b3164	-690	0	*pnp*	b3164	-104,12
PsrO	2	*ydhI*	b1643	-96	0.000315998	*yciI*	b1251	-17,84
PsrO	3	*intQ*	b1579	-95	0.00036616	*yggF*	b2930	-17,62
PsrO	4	b2859	b2859	-95	0.00036616	*yegW*	b2101	-17,17
PsrO	5	*yqiG*	b3046	-95	0.00036616	*intQ*	b1579	-16,9
PsrO	6	*yadG*	b0127	-92	0.000569665	*ygeO*	b2859	-16,21
PsrO	7	*ybjH*	b0843	-92	0.000569665	*sirA*	b3470	-15,71
PsrO	8	*thiF*	b3992	-91	0.000660081	*ymgI*	b4593	-15,68
PsrO	9	*yjdP*	b4487	-90	0.000764843	*yfaT*	b2229	-15,47
PsrO	10	*yedM*	b1935	-87	0.0011898	*tyrA*	b2600	-15,47
PsrO	11	*yegW*	b2101	-83	0.00214416	*truA*	b2318	-15,02
PsrO	12	*pdxJ*	b2564	-83	0.00214416	*yhjQ*	b3534	-14,96
PsrO	13	*yojL*	b2214	-82	0.00248416	*flgB*	b1073	-14,82
PsrO	14	*yghO*	b2981	-82	0.00248416	*yhaM*	b4470	-13,77
PsrO	15	*rbsR*	b3753	-82	0.00248416	*cysC*	b2750	-13,21

PsrO	16	*yphC*	b2545	-81	0.00287801	*wbbK*	b2032	-13,16
PsrO	17	*yciI*	b1251	-80	0.00333419	*nfsB*	b0578	-13,09
PsrO	18	*yfaP*	b2225	-80	0.00333419	*yjdL*	b4130	-13,08
PsrO	19	*yhbZ*	b3183	-80	0.00333419	*cheW*	b1887	-12,75
PsrO	20	*phoH*	b1020	-79	0.00386254	*nsrR*	b4178	-12,74
PsrO	21	*mviN*	b1069	-79	0.00386254	*yggN*	b2958	-12,65
PsrO	22	*yjhC*	b4280	-79	0.00386254	*rpmC*	b3312	-12,52
PsrO	23	*gntT*	b3415	-78	0.00447443	*hcaD*	b2542	-12,5
PsrO	24	*corA*	b3816	-78	0.00447443	*eutQ*	b2460	-12,43
PsrO	25	*norM*	b1663	-77	0.00518299	*minC*	b1176	-12,42
GcvB	1	*gltI*	b0655	-110	2.09496e-05	*argT*	b2310	-23,73
GcvB	2	*gcvA*	b2808	-110	2.09496e-05	*yhjQ*	b3534	-21,61
GcvB	3	*argT*	b2310	-108	2.84647e-05	*ebgR*	b3075	-21,53
GcvB	4	*gst*	b1635	-97	0.000153644	*cycA*	b4208	-20,93
GcvB	5	*livK*	b3458	-97	0.000153644	*livK*	b3458	-19,82
GcvB	6	*fabI*	b1288	-96	0.000179092	*gltI*	b0655	-19,81
GcvB	7	*glpR*	b3423	-96	0.000179092	*asnB*	b0674	-19,15
GcvB	8	*dppA*	b3544	-91	0.000385359	*abgT*	b1336	-19,1
GcvB	9	*leuB*	b0073	-89	0.000523564	*yafX*	b0248	-17,27
GcvB	10	*aroC*	b2329	-88	0.000610264	*dppA*	b3544	-16,52
GcvB	11	*yfdT*	b2363	-86	0.000829094	*fes*	b0585	-16,33
GcvB	12	*glgB*	b3432	-86	0.000829094	*moeA*	b0827	-16,32
GcvB	13	*aroP*	b0112	-84	0.00112635	*creC*	b4399	-16,28
GcvB	14	*ymfL*	b1147	-84	0.00112635	*cheY*	b1882	-16,23
GcvB	15	*yfcJ*	b2322	-84	0.00112635	*glpR*	b3423	-16,19
GcvB	16	*livJ*	b3460	-84	0.00112635	*menC*	b2261	-16,17
GcvB	17	*mltD*	b0211	-82	0.0015301	*gcvB*	b4443	-16
GcvB	18	*ybjF*	b0859	-82	0.0015301	*yahI*	b0323	-15,75
GcvB	19	*ugd*	b2028	-82	0.0015301	*yfcJ*	b2322	-15,7
GcvB	20	*yhfS*	b3376	-81	0.00178332	*rffD*	b3787	-15,68
GcvB	21	b3776	b3776	-81	0.00178332	*lrp*	b0889	-15,42
GcvB	22	*ycbS*	b0940	-80	0.00207842	*yqeB*	b2875	-15,39
GcvB	23	*ygcE*	b2776	-80	0.00207842	*kefF*	b0046	-15,36
GcvB	24	*intS*	b2349	-79	0.00242228	*yfdZ*	b2379	-15,26
GcvB	25	*uxuR*	b4324	-79	0.00242228	*metF*	b3941	-15,23
RyeA	1	*pphA*	b1838	-285	0	*pphA*	b1838	-63,92
RyeA	2	*glnL*	b3869	-88	0.000503557	*yiaA*	b3562	-16,44
RyeA	3	*pitB*	b2987	-84	0.000937598	*yhjH*	b3525	-15,13
RyeA	4	*yfbO*	b2274	-81	0.00149432	*gspM*	b3334	-14,8
RyeA	5	*ynfJ*	b1592	-80	0.00174543	*fiu*	b0805	-14,3
RyeA	6	*fimE*	b4313	-79	0.00203871	*dnaX*	b0470	-14,27
RyeA	7	*manA*	b1613	-77	0.00278115	*rfaG*	b3631	-14,25
RyeA	8	*fhuF*	b4367	-76	0.00324817	*yecT*	b1877	-13,3

RyeA	9	*srlB*	b2704	-75	0.00379346	*rph*	b3643	-13,28
RyeA	10	*livG*	b3455	-75	0.00379346	*yfiM*	b2586	-13,25
RyeA	11	*ftsQ*	b0093	-72	0.00604078	*ynaI*	b1330	-12,95
RyeA	12	*btuE*	b1710	-71	0.00705322	*yjiK*	b4333	-12,91
RyeA	13	*yggC*	b2928	-71	0.00705322	*dacD*	b2010	-12,82
RyeA	14	*sfcA*	b1479	-70	0.00823464	*gspD*	b3325	-12,62
RyeA	15	*yeaD*	b1780	-70	0.00823464	*chpS*	b4224	-12,52
RyeA	16	*yeeO*	b1985	-70	0.00823464	*ycfS*	b1113	-12,42
RyeA	17	*ybeU*	b0648	-69	0.009613	*ynjI*	b1762	-12,1
RyeA	18	*ypdF*	b2385	-69	0.009613	*yheS*	b3352	-12,06
RyeA	19	*yidG*	b3675	-68	0.0112208	*scpA*	b2917	-11,93
RyeA	20	*yijP*	b3955	-68	0.0112208	*yifL*	b4558	-11,93
RyeA	21	*abgB*	b1337	-67	0.0130956	*hisJ*	b2309	-11,87
RyeA	22	*lpp*	b1677	-66	0.0152814	*perR*	b0254	-11,84
RyeA	23	*yjdC*	b4135	-66	0.0152814	*yhcH*	b3221	-11,78
RyeA	24	*bcr*	b2182	-65	0.0178286	*ydcF*	b1414	-11,78
RyeA	25	*yfaO*	b2251	-65	0.0178286	*gltL*	b0652	-11,75
IsrA	1	*amiB*	b4169	-104	7.06393e-05	*csgD*	b1040	-17,33
IsrA	2	*yfjU*	b2638	-99	0.00014986	*hycD*	b2722	-16,25
IsrA	3	*cdsA*	b0175	-85	0.00123053	*dcuD*	b3227	-16,05
IsrA	4	b0362	b0362	-85	0.00123053	*fxsA*	b4140	-15,76
IsrA	5	*ycgN*	b1181	-85	0.00123053	*yagT*	b0286	-15,29
IsrA	6	*csgD*	b1040	-80	0.00260885	*yhfS*	b3376	-15,14
IsrA	7	*gyrA*	b2231	-79	0.00303172	*amiB*	b4169	-15,08
IsrA	8	*napC*	b2202	-78	0.003523	*mhpD*	b0350	-14,96
IsrA	9	*yceG*	b1097	-77	0.00409374	*rtn*	b2176	-14,69
IsrA	10	*yfdV*	b2372	-77	0.00409374	*gadY*	b4452	-14,62
IsrA	11	*yfiP*	b2583	-77	0.00409374	*suhB*	b2533	-14,56
IsrA	12	*mhpD*	b0350	-76	0.00475672	*ivbL*	b3672	-14,54
IsrA	13	*ycjU*	b1317	-76	0.00475672	*yhdL*	b4550	-14,31
IsrA	14	*ybdJ*	b0580	-75	0.00552676	*ampC*	b4150	-14,09
IsrA	15	*rtn*	b2176	-75	0.00552676	*ybhC*	b0772	-13,96
IsrA	16	*kefA*	b0465	-74	0.00642106	*yjbH*	b4029	-13,84
IsrA	17	*rseB*	b2571	-74	0.00642106	*ygeQ*	b2863	-13,65
IsrA	18	*xylF*	b3566	-74	0.00642106	*mdtE*	b3513	-13,52
IsrA	19	*cusR*	b0571	-73	0.00745953	*bglF*	b3722	-13,47
IsrA	20	*ynfC*	b1585	-73	0.00745953	*ptrB*	b1845	-13,41
IsrA	21	*yfiQ*	b2584	-73	0.00745953	*ypjF*	b2646	-13,37
IsrA	22	*apaH*	b0049	-72	0.00866522	*nuoH*	b2282	-13,37
IsrA	23	*yciA*	b1253	-72	0.00866522	*bioH*	b3412	-13,3
IsrA	24	*ygiV*	b3023	-72	0.00866522	*ydjX*	b1750	-13,29
IsrA	25	*slp*	b3506	-72	0.00866522	*nikE*	b3480	-13,27
RttR	1	*ylbH*	b0499	-78	0.000820339	*yhjQ*	b3534	-22,69

RttR	2	*gcvT*	b2905	-70	0.00317036	*hyaE*	b0976	-19,65
RttR	3	*yjeJ*	b4145	-69	0.00375349	*yciY*	b4595	-17,85
RttR	4	*yccX*	b0968	-68	0.00444363	*tktB*	b2465	-17,49
RttR	5	*celB*	b1737	-67	0.00526034	*bamC*	b2477	-16,65
RttR	6	*arcA*	b4401	-63	0.010321	*sdhA*	b0723	-16,44
RttR	7	*yffL*	b2443	-60	0.0170842	*yccX*	b0968	-16,2
RttR	8	*ybaD*	b0413	-59	0.0202005	*aspA*	b4139	-15,96
RttR	9	*btuB*	b3966	-59	0.0202005	*ybjM*	b0848	-15,78
RttR	10	*prpB*	b0331	-58	0.0238782	*creC*	b4399	-15,73
RttR	11	*yegW*	b2101	-58	0.0238782	*infC*	b1718	-15,65
RttR	12	*metL*	b3940	-58	0.0238782	*yfjS*	b2636	-15,62
RttR	13	*ydfI*	b1542	-57	0.0282157	*yedQ*	b1956	-15,51
RttR	14	*yehB*	b2109	-57	0.0282157	*ygbJ*	b2736	-15,48
RttR	15	*yeiR*	b2173	-57	0.0282157	*lrp*	b0889	-15,3
RttR	16	*perM*	b2493	-57	0.0282157	*yfaZ*	b2250	-15,29
RttR	17	*yphG*	b2549	-57	0.0282157	*codB*	b0336	-15,07
RttR	18	*yicE*	b3654	-57	0.0282157	*yahE*	b0319	-14,85
RttR	19	*wzxE*	b3792	-57	0.0282157	*tmk*	b1098	-14,66
RttR	20	*pfkA*	b3916	-57	0.0282157	*valZ*	b0746	-14,61
RttR	21	*sdhA*	b0723	-55	0.0393469	*glcA*	b2975	-14,59
RttR	22	*yliH*	b0836	-55	0.0393469	*idnK*	b4268	-14,52
RttR	23	*ymfN*	b1149	-55	0.0393469	*aceB*	b4014	-14,45
RttR	24	*ispF*	b2746	-55	0.0393469	*gabP*	b2663	-14,43
RttR	25	*rpsH*	b3306	-55	0.0393469	*ycaM*	b0899	-14,35
RdlD	1	*ldrD*	b4453	-165	2.3231e-10	*ldrD*	b4453	-45,88
RdlD	2	*ldrB*	b4421	-110	2.87445e-06	*ldrC*	b4423	-25,66
RdlD	3	*ldrC*	b4423	-110	2.87445e-06	*ldrB*	b4421	-25,64
RdlD	4	*ldrA*	b4419	-103	9.53708e-06	*ldrA*	b4419	-23,82
RdlD	5	*fimE*	b4313	-64	0.00758075	*ybcK*	b0544	-9,97
RdlD	6	*yraL*	b3146	-63	0.0089911	*eutN*	b2456	-9,46
RdlD	7	*yibO*	b3612	-62	0.0106624	*rdlA*	b4420	-9,37
RdlD	8	*yaaH*	b0010	-61	0.0126424	*yeiT*	b2146	-9,32
RdlD	9	*ytfP*	b4222	-61	0.0126424	*hipA*	b1507	-8,34
RdlD	10	*wecG*	b3794	-60	0.0149873	*yeaM*	b1790	-8,3
RdlD	11	*zntA*	b3469	-58	0.0210478	*yghB*	b3009	-7,67
RdlD	12	*ydfA*	b1571	-55	0.0349416	*dacA*	b0632	-7,57
RdlD	13	*yfgC*	b2494	-55	0.0349416	*hisC*	b2021	-7,44
RdlD	14	*dhaK*	b1200	-54	0.041335	*ygjN*	b3083	-7,44
RdlD	15	*ynfI*	b1591	-54	0.041335	*ldcC*	b0186	-7,36
RdlD	16	*yeeE*	b2013	-54	0.041335	*fmt*	b3288	-7,34
RdlD	17	*yfjZ*	b2645	-54	0.041335	*treA*	b1197	-7,26
RdlD	18	*ycfM*	b1105	-53	0.0488683	*fadH*	b3081	-7,21
RdlD	19	*yfdU*	b2373	-52	0.0577326	*ycbG*	b0956	-7,19
RdlD	20	*prmA*	b3259	-52	0.0577326	*yedZ*	b1972	-6,96

RdlD	21	*atpI*	b3739	-52	0.0577326	*hisI*	b2026	-6,84
RdlD	22	*sugE*	b4148	-52	0.0577326	*yfiB*	b2605	-6,8
RdlD	23	*imp*	b0054	-51	0.0681464	*rdlB*	b4422	-6,8
RdlD	24	*tolB*	b0740	-51	0.0681464	*rdlD*	b4454	-6,76
RdlD	25	*brnQ*	b0401	-50	0.0803571	*ruvA*	b1861	-6,52
SibA	1	*gcvP*	b2903	-100	0.000142905	*xylF*	b3566	-19,69
SibA	2	*plsX*	b1090	-89	0.000739005	*hofO*	b3393	-17,98
SibA	3	*yihR*	b3879	-83	0.00181018	*yidR*	b3689	-17,29
SibA	4	*dedD*	b2314	-82	0.00210157	*ypfN*	b4547	-17,15
SibA	5	*pdxB*	b2320	-78	0.00381687	*fliK*	b1943	-16,92
SibA	6	*cmtB*	b2934	-78	0.00381687	*secA*	b0098	-16,64
SibA	7	*mog*	b0009	-77	0.00443056	*fimG*	b4319	-16,6
SibA	8	*melR*	b4118	-77	0.00443056	*puuP*	b1296	-16,27
SibA	9	*osmY*	b4376	-77	0.00443056	*uxuB*	b4323	-16,06
SibA	10	*ykgK*	b0294	-76	0.00514266	*yoaA*	b1808	-15,96
SibA	11	*pdxY*	b1636	-76	0.00514266	*purN*	b2500	-15,9
SibA	12	*gatY*	b2096	-76	0.00514266	*ybdO*	b0603	-15,61
SibA	13	*yhaC*	b3121	-76	0.00514266	*ycgF*	b1163	-15,51
SibA	14	*nanR*	b3226	-76	0.00514266	*yidK*	b3679	-15,47
SibA	15	*mrcB*	b0149	-75	0.00596887	*yfjM*	b2629	-15,39
SibA	16	*ycfP*	b1108	-75	0.00596887	*cadC*	b4133	-15,33
SibA	17	*ygdR*	b2833	-74	0.00692735	*yaeR*	b0187	-15,27
SibA	18	*fepB*	b0592	-72	0.00932849	*yffM*	b2444	-15,25
SibA	19	*gltK*	b0653	-72	0.00932849	*pyrG*	b2780	-15,09
SibA	20	*ygcJ*	b2758	-71	0.0108235	*mog*	b0009	-14,91
SibA	21	*yibA*	b3594	-71	0.0108235	*nhaB*	b1186	-14,9
SibA	22	*malK*	b4035	-71	0.0108235	*yceI*	b1056	-14,9
SibA	23	*yciS*	b1279	-70	0.0125566	*mdtN*	b4082	-14,86
SibA	24	*yidJ*	b3678	-70	0.0125566	*dgoR*	b4479	-14,75
SibA	25	*zipA*	b2412	-69	0.0145652	*psaA*	b4645	-14,64
RyhA	1	*yhbL*	b3209	-210	7.48057e-12	*elbB*	b3209	-34,72
RyhA	2	*yciF*	b1258	-94	0.000301949	*metR*	b3828	-17,07
RyhA	3	*yfaU*	b2245	-86	0.00101003	*agaV*	b3133	-16,22
RyhA	4	*ycaR*	b0917	-85	0.00117454	*fliF*	b1938	-15,06
RyhA	5	*cysC*	b2750	-81	0.00214751	*ttdR*	b3060	-14,37
RyhA	6	*ygcO*	b2767	-81	0.00214751	*aspA*	b4139	-13,81
RyhA	7	*yadR*	b0156	-80	0.00249706	*rssA*	b1234	-13,71
RyhA	8	*trpB*	b1261	-80	0.00249706	*rbsA*	b3749	-13,45
RyhA	9	*ylcE*	b0563	-79	0.00290342	*fruR*	b0080	-13,42
RyhA	10	*deaD*	b3162	-79	0.00290342	*yebO*	b1825	-13,36
RyhA	11	*btuD*	b1709	-77	0.00392488	*hsdS*	b4348	-13,3
RyhA	12	*cspF*	b1558	-76	0.00456307	*serT*	b0971	-13,29
RyhA	13	*ybhR*	b0792	-75	0.00530475	*bioC*	b0777	-13,07

RyhA	14	*ynfH*	b1590	-74	0.0061666	*ytfR*	b4485	-13,07
RyhA	15	*nuoC*	b2286	-74	0.0061666	*hycD*	b2722	-13,01
RyhA	16	*metR*	b3828	-74	0.0061666	*yedQ*	b1956	-12,99
RyhA	17	*tatE*	b0627	-73	0.00716798	*yggF*	b2930	-12,98
RyhA	18	*ydjN*	b1729	-73	0.00716798	*flgA*	b1072	-12,82
RyhA	19	*fliF*	b1938	-73	0.00716798	*hybA*	b2996	-12,8
RyhA	20	*greA*	b3181	-73	0.00716798	*aes*	b0476	-12,77
RyhA	21	*nanR*	b3226	-73	0.00716798	*btuD*	b1709	-12,73
RyhA	22	*yeaU*	b1800	-72	0.00833129	*yraK*	b3145	-12,63
RyhA	23	*hycH*	b2718	-72	0.00833129	*yegU*	b2099	-12,63
RyhA	24	*yebC*	b1864	-71	0.00968247	*yhjQ*	b3534	-12,41
RyhA	25	b2857	b2857	-71	0.00968247	*yhiS*	b3504	-12,34
RdlB	1	*ldrB*	b4421	-270	0	*ldrB*	b4421	-61,49
RdlB	2	*ldrC*	b4423	-259	2.22045e-16	*ldrC*	b4423	-56,28
RdlB	3	*ldrA*	b4419	-244	2.77556e-15	*ldrA*	b4419	-54,62
RdlB	4	*ldrD*	b4453	-171	3.92937e-10	*ldrD*	b4453	-29,82
RdlB	5	*fimI*	b4315	-81	0.00086263	*marB*	b1532	-11,96
RdlB	6	*hslJ*	b1379	-75	0.00228189	*glxR*	b0509	-11,39
RdlB	7	*frdB*	b4153	-73	0.00315523	*yfgC*	b2494	-10,59
RdlB	8	*ymfL*	b1147	-72	0.00371	*ycgH*	b4491	-10,52
RdlB	9	*marA*	b1531	-71	0.00436209	*chbG*	b1733	-10,08
RdlB	10	*ydiI*	b1686	-71	0.00436209	*yfbL*	b2271	-9,89
RdlB	11	*yhiJ*	b3488	-71	0.00436209	*deaD*	b3162	-9,64
RdlB	12	*ego*	b1513	-70	0.00512851	*ydaL*	b1340	-9,57
RdlB	13	*yfiA*	b2597	-70	0.00512851	*tyrS*	b1637	-9,33
RdlB	14	*oppA*	b1243	-69	0.00602917	*ybaT*	b0486	-9,15
RdlB	15	*dcp*	b1538	-66	0.00979096	*ynhF*	b4602	-9,08
RdlB	16	*yegU*	b2099	-66	0.00979096	*rplF*	b3305	-9,08
RdlB	17	*ycdJ*	b1009	-65	0.0115057	*ydiY*	b1722	-9,02
RdlB	18	*bglG*	b3723	-65	0.0115057	*gsk*	b0477	-8,96
RdlB	19	*yohD*	b2136	-64	0.0135187	*rfbC*	b2038	-8,84
RdlB	20	*bglF*	b3722	-64	0.0135187	*yfbO*	b2274	-8,5
RdlB	21	*ubiA*	b4040	-64	0.0135187	*quuQ*	b1559	-8,47
RdlB	22	*hlpA*	b0178	-63	0.015881	*otsA*	b1896	-8,44
RdlB	23	*bioF*	b0776	-63	0.015881	*grpE*	b2614	-8,37
RdlB	24	*dhaK*	b1200	-63	0.015881	*gadY*	b4452	-8,16
RdlB	25	*ydjH*	b1772	-63	0.015881	*galK*	b0757	-8,03
RdlA	1	*ldrA*	b4419	-300	0	*ldrA*	b4419	-70,21
RdlA	2	*ldrB*	b4421	-277	0	*ldrB*	b4421	-59,37
RdlA	3	*ldrC*	b4423	-266	1.11022e-16	*ldrC*	b4423	-53,54
RdlA	4	*ldrD*	b4453	-197	8.27927e-12	*ldrD*	b4453	-32,78
RdlA	5	*fimI*	b4315	-76	0.002228	*yhjH*	b3525	-13,09
RdlA	6	*ymfL*	b1147	-73	0.00360275	*yhjR*	b3535	-12,35

RdlA	7	*ybiO*	b0808	-70	0.00582329	*yqgE*	b2948	-12,28
RdlA	8	*yheU*	b3354	-70	0.00582329	*chbF*	b1734	-12,09
RdlA	9	*malP*	b3417	-70	0.00582329	*uspG*	b0607	-11,23
RdlA	10	*oppA*	b1243	-69	0.00683314	*galR*	b2837	-11,17
RdlA	11	*pgm*	b0688	-66	0.0110337	*yfaT*	b2229	-11,17
RdlA	12	*ego*	b1513	-66	0.0110337	*ftsK*	b0890	-11,14
RdlA	13	*ndk*	b2518	-66	0.0110337	*deoC*	b4381	-10,66
RdlA	14	*phnJ*	b4098	-65	0.0129412	*ribF*	b0025	-10,64
RdlA	15	*hslJ*	b1379	-64	0.015176	*yiaT*	b3584	-10,62
RdlA	16	*manX*	b1817	-64	0.015176	*serS*	b0893	-10,44
RdlA	17	*ygiD*	b3039	-64	0.015176	*ymfI*	b1143	-10,3
RdlA	18	*dhaK*	b1200	-63	0.0177931	*yghE*	b2969	-10,14
RdlA	19	*trpB*	b1261	-63	0.0177931	*ydiT*	b1700	-10,06
RdlA	20	*dcp*	b1538	-63	0.0177931	*mukF*	b0922	-10,01
RdlA	21	*yohD*	b2136	-63	0.0177931	*entS*	b0591	-9,99
RdlA	22	*yccJ*	b1003	-62	0.0208569	*yciT*	b1284	-9,67
RdlA	23	*yffM*	b2444	-62	0.0208569	*ygcR*	b2770	-9,66
RdlA	24	*gcvT*	b2905	-62	0.0208569	*rplS*	b2606	-9,62
RdlA	25	*ubiA*	b4040	-62	0.0208569	*intR*	b1345	-9,61
RybA	1	*csiE*	b2535	-93	0.000134386	*atoD*	b2221	-17,12
RybA	2	*ubiX*	b2311	-82	0.000792172	*yegS*	b2086	-16,89
RybA	3	*yjiQ*	b4339	-73	0.00337874	*yaeJ*	b0191	-16,8
RybA	4	*yeaT*	b1799	-71	0.00466215	*ykgP*	b4630	-15,75
RybA	5	*yfhM*	b2520	-71	0.00466215	*proB*	b0242	-15,65
RybA	6	*tyrB*	b4054	-70	0.00547601	*ybdG*	b0577	-15,15
RybA	7	*yhfK*	b3358	-68	0.00755303	*nikB*	b3477	-15,12
RybA	8	*ymfP*	b1152	-67	0.00886929	*galT*	b0758	-14,8
RybA	9	*ygaH*	b2683	-67	0.00886929	*tyrB*	b4054	-14,23
RybA	10	*rrmB*	b3289	-66	0.0104137	*arpB*	b4494	-14,16
RybA	11	*rffT*	b4481	-66	0.0104137	*yhfK*	b3358	-13,85
RybA	12	*codB*	b0336	-64	0.01435	*yjbB*	b4020	-13,79
RybA	13	*ybaQ*	b0483	-64	0.01435	*malT*	b3418	-13,42
RybA	14	*galT*	b0758	-64	0.01435	*hisL*	b2018	-13,29
RybA	15	*ycbJ*	b0919	-64	0.01435	*rsxG*	b1631	-13,08
RybA	16	*wecG*	b3794	-64	0.01435	*rsmB*	b3289	-13,07
RybA	17	*yfeH*	b2410	-63	0.0168407	*marR*	b1530	-12,97
RybA	18	*exbB*	b3006	-63	0.0168407	*pqiB*	b0951	-12,93
RybA	19	*ligT*	b0147	-62	0.0197593	*exbB*	b3006	-12,89
RybA	20	*flgC*	b1074	-62	0.0197593	*ydiY*	b1722	-12,77
RybA	21	*ydjY*	b1751	-62	0.0197593	*yciW*	b1287	-12,73
RybA	22	*yi91a*	b0255	-61	0.0231778	*emrK*	b2368	-12,57
RybA	23	*ybcL*	b0545	-61	0.0231778	*yjbE*	b4026	-12,48
RybA	24	*marR*	b1530	-61	0.0231778	*pepE*	b4021	-12,35
RybA	25	*yejE*	b2179	-61	0.0231778	*potB*	b1125	-12,32

OmrA	1	*ispD*	b2747	-82	0.000851486	*yjcH*	b4068	-15,43
OmrA	2	*gidB*	b3740	-81	0.000999581	*yedN*	b4495	-15,13
OmrA	3	*ygeR*	b2865	-76	0.002228	*yncI*	b1459	-14,65
OmrA	4	*celB*	b1737	-75	0.00261519	*dgoD*	b4478	-14,22
OmrA	5	*lit*	b1139	-74	0.00306957	*yhjQ*	b3534	-14,04
OmrA	6	*ycjS*	b1315	-72	0.00422834	*yeiH*	b2158	-13,67
OmrA	7	*flk*	b2321	-72	0.00422834	*yncJ*	b1436	-12,64
OmrA	8	*ssnA*	b2879	-72	0.00422834	*yahE*	b0319	-12,17
OmrA	9	*yjhB*	b4279	-71	0.0049623	*yahB*	b0316	-12,13
OmrA	10	*yadL*	b0137	-70	0.00582329	*ymiA*	b4522	-12,07
OmrA	11	*eutQ*	b2460	-70	0.00582329	*leuU*	b3174	-12,04
OmrA	12	*yiaA*	b3562	-70	0.00582329	*yccA*	b0970	-11,92
OmrA	13	*deoR*	b0840	-69	0.00683314	*fabA*	b0954	-11,92
OmrA	14	*gmhB*	b0200	-67	0.00940597	*yciX*	b4523	-11,9
OmrA	15	*glcD*	b2979	-67	0.00940597	*dxs*	b0420	-11,86
OmrA	16	*yccS*	b0960	-65	0.0129412	*deoD*	b4384	-11,7
OmrA	17	*appC*	b0978	-65	0.0129412	*ygeR*	b2865	-11,68
OmrA	18	*ychK*	b1234	-65	0.0129412	*psrO*	b4449	-11,65
OmrA	19	*yfcK*	b2324	-65	0.0129412	*btuR*	b1270	-11,62
OmrA	20	*glmS*	b3729	-64	0.015176	*pldB*	b3825	-11,61
OmrA	21	*yccK*	b0969	-63	0.0177931	*ydcQ*	b1438	-11,6
OmrA	22	*rpmC*	b3312	-63	0.0177931	*ompT*	b0565	-11,59
OmrA	23	*yadD*	b0132	-62	0.0208569	*yjiJ*	b4332	-11,52
OmrA	24	*yafJ*	b0223	-62	0.0208569	*fhuC*	b0151	-11,48
OmrA	25	*yefM*	b2017	-62	0.0208569	*yhcC*	b3211	-11,47
PsrD	1	*yceF*	b1087	-420	0	*yceF*	b1087	-127,69
PsrD	2	*ydeO*	b1499	-88	0.000529971	*yhdJ*	b3262	-15,6
PsrD	3	*dps*	b0812	-87	0.000618722	*yfdP*	b2359	-14,21
PsrD	4	*yncA*	b1448	-85	0.000843283	*yiaT*	b3584	-11,15
PsrD	5	*narU*	b1469	-85	0.000843283	*ilvE*	b3770	-11,03
PsrD	6	*mglC*	b2148	-85	0.000843283	*glpR*	b3423	-10,52
PsrD	7	*trs5_2*	b0552	-83	0.0011493	*ybdF*	b0579	-10,49
PsrD	8	*mqo*	b2210	-83	0.0011493	*fdnI*	b1476	-10,48
PsrD	9	*ynjI*	b1762	-81	0.00156628	*rfaF*	b3620	-10,4
PsrD	10	*ybaV*	b0442	-80	0.00182842	*yohJ*	b2141	-9,96
PsrD	11	*pspA*	b1304	-80	0.00182842	*acnA*	b1276	-9,95
PsrD	12	*ebgA*	b3076	-80	0.00182842	*micC*	b4427	-9,85
PsrD	13	*pyrH*	b0171	-79	0.00213438	*yfgM*	b2513	-9,82
PsrD	14	*acrB*	b0462	-79	0.00213438	*tfaS*	b2353	-9,77
PsrD	15	*ygdP*	b2830	-79	0.00213438	*insH*	b0259	-9,76
PsrD	16	*ynbC*	b1410	-78	0.00249148	*frsA*	b0239	-9,55
PsrD	17	*ascG*	b2714	-78	0.00249148	*ybcI*	b0527	-9,54
PsrD	18	*yqeB*	b2875	-78	0.00249148	*rpsN*	b3307	-9,47

PsrD	19	*yhdJ*	b3262	-78	0.00249148	*ompF*	b0929	-9,44
PsrD	20	*yhdU*	b3263	-78	0.00249148	*cusS*	b0570	-9,42
PsrD	21	*yjcC*	b4061	-78	0.00249148	*cobU*	b1993	-9,36
PsrD	22	*ycjU*	b1317	-77	0.00290824	*malT*	b3418	-9,2
PsrD	23	*yoaF*	b1793	-76	0.00339459	*hisM*	b2307	-9,19
PsrD	24	*ascF*	b2715	-76	0.00339459	*cysA*	b2422	-9,12
PsrD	25	*pgi*	b4025	-76	0.00339459	*yjgF*	b4243	-9,08
RyfA	1	*tmk*	b1098	-120	2.76503e-05	*bolA*	b0435	-16,49
RyfA	2	*yaaH*	b0010	-112	8.35237e-05	*yodC*	b1957	-14,88
RyfA	3	*ydfT*	b1559	-107	0.000166674	*omrB*	b4445	-14,57
RyfA	4	*purC*	b2476	-101	0.000381867	*yraH*	b3142	-14,56
RyfA	5	*yfhG*	b2555	-98	0.000577983	*yagS*	b0285	-14,42
RyfA	6	*folK*	b0142	-95	0.000874775	*ydcV*	b1443	-13,8
RyfA	7	*yfjY*	b2644	-94	0.00100435	*argY*	b2693	-13,62
RyfA	8	*yjeS*	b4166	-94	0.00100435	*argQ*	b2691	-13,62
RyfA	9	*fliL*	b1944	-92	0.00132387	*valT*	b0744	-13,54
RyfA	10	*mepA*	b2328	-92	0.00132387	*lysQ*	b0749	-13,53
RyfA	11	*motA*	b1890	-90	0.00174495	*yaaH*	b0010	-13,43
RyfA	12	*ynfJ*	b1592	-89	0.00200328	*ylbF*	b0520	-13,41
RyfA	13	*nrdH*	b2673	-89	0.00200328	*nhaA*	b0019	-13,4
RyfA	14	*yhiO*	b3494	-89	0.00200328	*yfiQ*	b2584	-13,32
RyfA	15	*paaE*	b1392	-88	0.00229982	*gcvB*	b4443	-13,29
RyfA	16	*yjcP*	b4080	-88	0.00229982	*prlC*	b3498	-13,26
RyfA	17	*yjiX*	b4353	-88	0.00229982	*znuC*	b1858	-12,89
RyfA	18	*ampE*	b0111	-87	0.00264018	*accA*	b0185	-12,7
RyfA	19	*yfeD*	b2399	-87	0.00264018	*yjbR*	b4057	-12,64
RyfA	20	*lplA*	b4386	-87	0.00264018	*astB*	b1745	-12,56
RyfA	21	*yeaZ*	b1807	-85	0.00347922	*ytjC*	b4395	-12,24
RyfA	22	*csgG*	b1037	-84	0.00399379	*ynjD*	b1756	-12,22
RyfA	23	*tap*	b1885	-84	0.00399379	*nagK*	b1119	-12,15
RyfA	24	*gudP*	b2789	-84	0.00399379	*yhbQ*	b3155	-12,11
RyfA	25	*ynfH*	b1590	-83	0.00458429	*fdnG*	b1474	-11,99
SibC	1	*ykgI*	b0303	-79	0.00280717	*yhjC*	b3521	-14
SibC	2	*hyfE*	b2485	-76	0.00441756	*fliZ*	b1921	-12,96
SibC	3	b2681	b2681	-76	0.00441756	*yifE*	b3764	-12,55
SibC	4	*yghK*	b2975	-74	0.00597521	*dnaA*	b3702	-12,48
SibC	5	*ydhQ*	b1664	-72	0.00807986	*yoeB*	b4539	-12,06
SibC	6	*yfjB*	b2615	-72	0.00807986	*yfgM*	b2513	-12,05
SibC	7	*clpB*	b2592	-71	0.00939445	*yjiE*	b4327	-12,01
SibC	8	*yijC*	b3963	-70	0.0109217	*yodC*	b1957	-11,86
SibC	9	*mazG*	b2781	-69	0.0126957	*mraW*	b0082	-11,79
SibC	10	*pspA*	b1304	-68	0.0147557	*trmJ*	b2532	-11,67
SibC	11	b1998	b1998	-68	0.0147557	*ftsL*	b0083	-11,66

SibC	12	*gcvT*	b2905	-68	0.0147557	*ydhZ*	b1675	-11,6
SibC	13	*ybcH*	b0567	-67	0.0171471	*uspG*	b0607	-11,53
SibC	14	*yliF*	b0834	-67	0.0171471	*hycE*	b2721	-11,34
SibC	15	*ydaS*	b1357	-67	0.0171471	*yfaU*	b2245	-11,11
SibC	16	*ygaA*	b2709	-67	0.0171471	*yhfA*	b3356	-11,11
SibC	17	*yhdG*	b3260	-67	0.0171471	*yoaI*	b1788	-11,1
SibC	18	*cdh*	b3918	-67	0.0171471	*mltD*	b0211	-11,07
SibC	19	*glpX*	b3925	-67	0.0171471	*napH*	b2204	-10,99
SibC	20	*ybhE*	b0767	-66	0.019922	*ykgM*	b0296	-10,85
SibC	21	*ybiT*	b0820	-66	0.019922	*nupX*	b2161	-10,78
SibC	22	*ybjI*	b0844	-66	0.019922	*ybiB*	b0800	-10,78
SibC	23	*yobB*	b1843	-65	0.0231407	*ydiB*	b1692	-10,68
SibC	24	*hcaC*	b2540	-65	0.0231407	*fimF*	b4318	-10,66
SibC	25	*yqiC*	b3042	-65	0.0231407	*pheU*	b4134	-10,66
IsrC	1	*yhhN*	b3468	-78	0.00172489	*sseB*	b2522	-14,99
IsrC	2	*trs5_8*	b2192	-77	0.00202307	*ydeP*	b1501	-14,08
IsrC	3	*sseB*	b2522	-73	0.00382709	*ynjB*	b1754	-13,46
IsrC	4	*oppD*	b1246	-70	0.00617015	*yejO*	b2190	-13,19
IsrC	5	*dbpA*	b1343	-70	0.00617015	*ndk*	b2518	-12,5
IsrC	6	*yfgH*	b2505	-70	0.00617015	*paaK*	b1398	-12,22
IsrC	7	*allA*	b0505	-69	0.00723396	*leuT*	b3798	-12,17
IsrC	8	*fepD*	b0590	-65	0.0136528	*fkpB*	b0028	-12,13
IsrC	9	*ylaC*	b0458	-64	0.0159963	*ccmB*	b2200	-12,01
IsrC	10	*thrS*	b1719	-64	0.0159963	*ygaP*	b2668	-11,97
IsrC	11	*yfiR*	b2603	-64	0.0159963	*ydcY*	b1446	-11,73
IsrC	12	*ydcY*	b1446	-63	0.0187382	*oppD*	b1246	-11,68
IsrC	13	*hsdM*	b4349	-63	0.0187382	*fabR*	b3963	-11,67
IsrC	14	*yijP*	b3955	-62	0.0219448	*aqpZ*	b0875	-11,59
IsrC	15	*potB*	b1125	-61	0.0256929	*yphD*	b2546	-11,58
IsrC	16	*ugd*	b2028	-61	0.0256929	*insH*	b0259	-11,49
IsrC	17	*kdgT*	b3909	-61	0.0256929	*ivbL*	b3672	-11,49
IsrC	18	*mrr*	b4351	-61	0.0256929	*ygaY*	b2681	-11,26
IsrC	19	*rnk*	b0610	-60	0.0300714	*isrC*	b4435	-11,26
IsrC	20	*aqpZ*	b0875	-60	0.0300714	*ybgT*	b4515	-11,25
IsrC	21	*yraO*	b3149	-60	0.0300714	*aslB*	b3800	-11,23
IsrC	22	*atpF*	b3736	-60	0.0300714	*yahC*	b0317	-11,1
IsrC	23	*aidB*	b4187	-60	0.0300714	*rimN*	b3282	-11,06
IsrC	24	*ytfG*	b4211	-59	0.0351824	*nikC*	b3478	-11,05
IsrC	25	*nadD*	b0639	-58	0.0411435	*ypfN*	b4547	-11,03
SokB	1	*mokB*	b1420	-265	1.11022e-16	*hokB*	b4428	-67,49
SokB	2	*hokB*	b4428	-265	1.11022e-16	*mokB*	b1420	-61,3
SokB	3	*mokC*	b0018	-104	1.99791e-05	*relB*	b1564	-14,06
SokB	4	*hokC*	b4412	-104	1.99791e-05	*ftnA*	b1905	-13,84

SokB	5	*hokE*	b4415	-97	6.23452e-05	*ygbT*	b2755	-12,82
SokB	6	*phnD*	b4105	-84	0.000515909	*metF*	b3941	-12,8
SokB	7	*yhcO*	b3239	-77	0.00160906	*malP*	b3417	-12,25
SokB	8	*yqiI*	b3048	-76	0.00189285	*tisB*	b4618	-12,18
SokB	9	*srlB*	b2704	-74	0.0026192	*htpX*	b1829	-11,89
SokB	10	*ydhT*	b1669	-70	0.00501267	*yeeE*	b2013	-11,56
SokB	11	*hcr*	b0872	-69	0.00589498	*ung*	b2580	-11,51
SokB	12	*ymgC*	b1167	-69	0.00589498	*flgC*	b1074	-11,28
SokB	13	*yffN*	b2445	-65	0.0112649	*ysaC*	b4648	-11,2
SokB	14	*ydfA*	b1571	-63	0.0155595	*glpC*	b2243	-11,18
SokB	15	*ydfB*	b1572	-63	0.0155595	*erpA*	b0156	-11
SokB	16	*ydjC*	b1733	-63	0.0155595	*pgaD*	b1021	-10,97
SokB	17	*glpC*	b2243	-63	0.0155595	*yeaN*	b1791	-10,85
SokB	18	*bipA*	b3871	-63	0.0155595	*fliK*	b1943	-10,77
SokB	19	*hpt*	b0125	-62	0.0182811	*ybeR*	b0645	-10,6
SokB	20	*ycbC*	b0920	-62	0.0182811	*yjeT*	b4176	-10,52
SokB	21	*phnP*	b4092	-62	0.0182811	*rsmB*	b3289	-10,48
SokB	22	*ymjA*	b1295	-61	0.0214735	*envY*	b0566	-10,44
SokB	23	*yehT*	b2125	-60	0.0252162	*entF*	b0586	-10,39
SokB	24	*acrF*	b3266	-60	0.0252162	*dcd*	b2065	-10,39
SokB	25	*dsbB*	b1185	-59	0.0296012	*cheA*	b1888	-10,37
RyhB	1	*yhhY*	b3441	-145	4.71877e-08	*yhhY*	b3441	-30,89
RyhB	2	*sdhD*	b0722	-87	0.000458786	*rraB*	b4255	-18,67
RyhB	3	*kdpA*	b0698	-83	0.000864069	*sufB*	b1683	-18,21
RyhB	4	*yciT*	b1284	-83	0.000864069	*ykgE*	b0306	-16,99
RyhB	5	*dmsA*	b0894	-82	0.00101221	*sdhD*	b0722	-16,47
RyhB	6	*ssuC*	b0934	-79	0.00162708	*metH*	b4019	-16,39
RyhB	7	*xerC*	b3811	-77	0.00223249	*lpxA*	b0181	-15,91
RyhB	8	*yiaM*	b3577	-75	0.00306283	*yjeT*	b4176	-15,33
RyhB	9	*recD*	b2819	-73	0.00420134	*citX*	b0614	-14,97
RyhB	10	*pgk*	b2926	-71	0.00576183	*cysE*	b3607	-14,59
RyhB	11	*frdA*	b4154	-71	0.00576183	*erpA*	b0156	-14,46
RyhB	12	*pinH*	b2648	-69	0.00789964	*citG*	b0613	-14,15
RyhB	13	*radC*	b3638	-69	0.00789964	*mdtQ*	b2139	-14,06
RyhB	14	*yjcE*	b4065	-69	0.00789964	*dmsA*	b0894	-13,97
RyhB	15	*folD*	b0529	-68	0.00924844	*yodC*	b1957	-13,88
RyhB	16	*napF*	b2208	-68	0.00924844	*hemN*	b3867	-13,72
RyhB	17	*yhhM*	b3467	-68	0.00924844	*acpS*	b2563	-13,64
RyhB	18	*fadE*	b0221	-67	0.0108263	*ypdE*	b2384	-13,55
RyhB	19	*yagJ*	b0276	-67	0.0108263	*phoE*	b0241	-13,51
RyhB	20	*trpE*	b1264	-67	0.0108263	*ileY*	b2652	-13,5
RyhB	21	*trpL*	b1265	-67	0.0108263	*yifN*	b3777	-13,4
RyhB	22	*yebB*	b1862	-67	0.0108263	*astA*	b1747	-13,37
RyhB	23	*sodA*	b3908	-66	0.0126716	*yadH*	b0128	-13,35

RyhB	24	*motA*	b1890	-65	0.0148291	*sugE*	b4148	-13,28
RyhB	25	*cysE*	b3607	-64	0.0173507	*tag*	b3549	-13,24
RyjA	1	*yaeL*	b0176	-92	0.000340828	*fadI*	b2342	-17,06
RyjA	2	*galR*	b2837	-81	0.00183174	*minC*	b1176	-15,5
RyjA	3	*melB*	b4120	-78	0.00289669	*yciW*	b1287	-15,38
RyjA	4	*ygeZ*	b2873	-77	0.00337459	*insH*	b0259	-15,25
RyjA	5	*nikC*	b3478	-76	0.00393118	*yehH*	b4499	-15,05
RyjA	6	*yiaB*	b3563	-76	0.00393118	*ychJ*	b1233	-14,88
RyjA	7	*nfsA*	b0851	-75	0.00457936	*cysQ*	b4214	-14,75
RyjA	8	*ychJ*	b1233	-74	0.00533413	*phoP*	b1130	-14,58
RyjA	9	*mgsA*	b0963	-73	0.0062129	*ygeQ*	b2863	-14,52
RyjA	10	*narU*	b1469	-72	0.00723593	*yibL*	b3602	-14,38
RyjA	11	*secB*	b3609	-72	0.00723593	*nikC*	b3478	-14,33
RyjA	12	*ybbK*	b0489	-71	0.00842669	*xapA*	b2407	-13,82
RyjA	13	*epd*	b2927	-71	0.00842669	*deaD*	b3162	-13,79
RyjA	14	*phoP*	b1130	-70	0.00981244	*ddpA*	b1487	-13,73
RyjA	15	*yeeS*	b2002	-70	0.00981244	*yejO*	b2190	-13,65
RyjA	16	*ychP*	b1220	-69	0.0114248	*sspA*	b3229	-13,52
RyjA	17	*ydaY*	b1366	-69	0.0114248	*yfgF*	b2503	-13,02
RyjA	18	*ybhC*	b0772	-68	0.0133002	*ybeZ*	b0660	-12,98
RyjA	19	*trs5_8*	b2192	-68	0.0133002	*rtn*	b2176	-12,83
RyjA	20	*rfaC*	b3621	-68	0.0133002	*ulaF*	b4198	-12,78
RyjA	21	*yjiR*	b4340	-68	0.0133002	*yfdK*	b2354	-12,77
RyjA	22	*yfdH*	b2351	-67	0.0154811	*qmcA*	b0489	-12,71
RyjA	23	*yhfK*	b3358	-66	0.0180164	*matC*	b0292	-12,69
RyjA	24	*yhhJ*	b3485	-66	0.0180164	*ycgH*	b4491	-12,56
RyjA	25	*yfcC*	b2298	-65	0.0209623	*secB*	b3609	-12,51
OmrB	1	*ynaK*	b1365	-81	0.000907982	*nadA*	b0750	-19,12
OmrB	2	*rhoL*	b3782	-80	0.00106716	*yibD*	b3615	-17,75
OmrB	3	*yadL*	b0137	-77	0.00173241	*mutM*	b3635	-16,65
OmrB	4	*rhlB*	b3780	-74	0.00281178	*yeiH*	b2158	-15,69
OmrB	5	*yddR*	b1486	-73	0.00330417	*rpsB*	b0169	-14,97
OmrB	6	*yiaA*	b3562	-73	0.00330417	*vacJ*	b2346	-14,9
OmrB	7	b0816	b0816	-72	0.00388262	*purB*	b1131	-14,82
OmrB	8	*slp*	b3506	-72	0.00388262	*frvA*	b3900	-14,69
OmrB	9	*ybaE*	b0445	-69	0.00629744	*acrB*	b0462	-14,69
OmrB	10	*yqaD*	b2658	-67	0.00868996	*yjcH*	b4068	-14,3
OmrB	11	*celB*	b1737	-65	0.011986	*fimH*	b4320	-14,3
OmrB	12	*ycfV*	b1117	-64	0.0140735	*speG*	b1584	-14,09
OmrB	13	*yfcK*	b2324	-62	0.0193914	*csgD*	b1040	-14,06
OmrB	14	*yjhI*	b4299	-62	0.0193914	*arsR*	b3501	-13,59
OmrB	15	*gntP*	b4321	-62	0.0193914	*yedN*	b4495	-13,57
OmrB	16	*rnhA*	b0214	-61	0.0227538	*argE*	b3957	-13,56

OmrB	17	*ybcY*	b0562	-61	0.0227538	*yjhI*	b4299	-13,36
OmrB	18	*srlB*	b2704	-61	0.0227538	*fhuC*	b0151	-13,36
OmrB	19	*pldA*	b3821	-61	0.0227538	*relA*	b2784	-13,3
OmrB	20	*nanR*	b3226	-60	0.0266912	*emrY*	b2367	-13,24
OmrB	21	*melA*	b4119	-60	0.0266912	*yliL*	b0816	-13,22
OmrB	22	*ymfS*	b1155	-59	0.0312991	*trpD*	b1263	-13,16
OmrB	23	*sdaA*	b1814	-59	0.0312991	*malQ*	b3416	-13,14
OmrB	24	*yqiC*	b3042	-59	0.0312991	*paaK*	b1398	-13,03
OmrB	25	*setA*	b0070	-58	0.0366873	*yebF*	b1847	-12,98
RybB	1	*trpR*	b4393	-105	2.93212e-05	*yicJ*	b3657	-20,54
RybB	2	*yfjO*	b2631	-90	0.00031068	*gpt*	b0238	-18,67
RybB	3	*ygfA*	b2912	-78	0.00205158	*trpR*	b4393	-18,26
RybB	4	*pheP*	b0576	-77	0.00240081	*intB*	b4271	-17,58
RybB	5	*yfjM*	b2629	-76	0.00280941	*rfbD*	b2040	-17,11
RybB	6	*pitA*	b3493	-76	0.00280941	*ybhD*	b0768	-16,5
RybB	7	*ompN*	b1377	-75	0.00328744	*ydiT*	b1700	-16,27
RybB	8	*yegW*	b2101	-73	0.00450075	*potD*	b1123	-16,2
RybB	9	*uxaA*	b3091	-73	0.00450075	*glmM*	b3176	-16,07
RybB	10	*yhjS*	b3536	-71	0.00616049	*aspU*	b0206	-16,01
RybB	11	*sdhB*	b0724	-70	0.00720665	*aspT*	b3760	-16,01
RybB	12	*fruR*	b0080	-69	0.0084297	*trpT*	b3761	-15,91
RybB	13	*pqiB*	b0951	-69	0.0084297	*nupX*	b2161	-15,85
RybB	14	*yebU*	b1835	-69	0.0084297	*ynfF*	b1588	-15,27
RybB	15	*pinH*	b2648	-68	0.00985928	*ptsI*	b2416	-15,14
RybB	16	*nfnB*	b0578	-67	0.0115299	*ribD*	b0414	-14,74
RybB	17	*pflA*	b0902	-67	0.0115299	*miaB*	b0661	-14,42
RybB	18	*xylH*	b3568	-66	0.0134817	*dedD*	b2314	-14,42
RybB	19	*ydiB*	b1692	-65	0.0157612	*yhhZ*	b3442	-14,18
RybB	20	*yieE*	b3712	-65	0.0157612	*ybfA*	b0699	-14,08
RybB	21	*glpF*	b3927	-65	0.0157612	*xylH*	b3568	-13,99
RybB	22	*pinR*	b1374	-64	0.0184225	*wbbJ*	b2033	-13,84
RybB	23	*pinQ*	b1545	-64	0.0184225	*mokC*	b0018	-13,73
RybB	24	*dcm*	b1961	-64	0.0184225	*yfdT*	b2363	-13,69
RybB	25	*wbbJ*	b2033	-64	0.0184225	*gcd*	b0124	-13,66
PsrN	1	*ygjT*	b3088	-104	0.000117668	*yecJ*	b4537	-12,76
PsrN	2	*ycgJ*	b1177	-95	0.000435908	*tolC*	b3035	-12,46
PsrN	3	*ydjY*	b1751	-93	0.000583127	*setB*	b2170	-12,17
PsrN	4	*yhdP*	b4472	-87	0.00139569	*accD*	b2316	-12,11
PsrN	5	*bfr*	b3336	-83	0.00249662	*trg*	b1421	-12,11
PsrN	6	*ylbH*	b0499	-80	0.00386061	*ygcF*	b2777	-11,85
PsrN	7	*yiaF*	b3554	-79	0.00446401	*yjcO*	b4078	-11,66
PsrN	8	*yjcP*	b4080	-79	0.00446401	*insL*	b0016	-11,62
PsrN	9	*hdhA*	b1619	-78	0.00516147	*ydgD*	b1598	-11,5

PsrN	10	*ycdO*	b1018	-76	0.00689914	*hyi*	b0508	-11,38
PsrN	11	*cysZ*	b2413	-76	0.00689914	*yeeF*	b2014	-11,35
PsrN	12	*rpsP*	b2609	-76	0.00689914	*mfd*	b1114	-11,22
PsrN	13	*thiI*	b0423	-75	0.00797554	*ypjJ*	b4548	-11,2
PsrN	14	*shiA*	b1981	-75	0.00797554	*psaA*	b4645	-11,17
PsrN	15	*glyA*	b2551	-75	0.00797554	*qseB*	b3025	-11,08
PsrN	16	*acrE*	b3265	-75	0.00797554	*quuQ*	b1559	-10,89
PsrN	17	*yjiK*	b4333	-75	0.00797554	*ybfB*	b0702	-10,86
PsrN	18	*ytfM*	b4220	-74	0.00921911	*dnaT*	b4362	-10,76
PsrN	19	*potI*	b0857	-73	0.0106555	*ydaV*	b1360	-10,72
PsrN	20	*recE*	b1350	-73	0.0106555	*yfbL*	b2271	-10,72
PsrN	21	*otsA*	b1896	-73	0.0106555	*yafZ*	b0252	-10,6
PsrN	22	*ada*	b2213	-73	0.0106555	*gudD*	b2787	-10,47
PsrN	23	*yfbQ*	b2290	-73	0.0106555	*ygfA*	b2912	-10,47
PsrN	24	*thiH*	b3990	-73	0.0106555	*rcnR*	b2105	-10,45
PsrN	25	*putP*	b1015	-72	0.0123144	*yfdF*	b2345	-10,42
RdlC	1	*ldrB*	b4421	-284	0	*ldrC*	b4423	-67,8
RdlC	2	*ldrC*	b4423	-290	0	*ldrB*	b4421	-67,02
RdlC	3	*ldrA*	b4419	-251	1.22125e-15	*ldrA*	b4419	-58,86
RdlC	4	*ldrD*	b4453	-183	7.03434e-11	*ldrD*	b4453	-33,38
RdlC	5	*marB*	b1532	-101	3.81136e-05	*glxR*	b0509	-12,7
RdlC	6	*yfcJ*	b2322	-84	0.000588421	*dnaK*	b0014	-12,64
RdlC	7	*menD*	b2264	-79	0.0013157	*garD*	b3128	-12,04
RdlC	8	*cheZ*	b1881	-72	0.00405542	*marB*	b1532	-11,93
RdlC	9	b2596	b2596	-72	0.00405542	*metC*	b3008	-11,46
RdlC	10	*frdB*	b4153	-72	0.00405542	*acrA*	b0463	-11,2
RdlC	11	*dcp*	b1538	-71	0.0047622	*yciT*	b1284	-10,65
RdlC	12	*yfiA*	b2597	-71	0.0047622	*cbpM*	b0999	-10,28
RdlC	13	*dnaK*	b0014	-69	0.00656545	*ycgH*	b4491	-10,19
RdlC	14	*hslJ*	b1379	-69	0.00656545	*yecH*	b1906	-10,13
RdlC	15	*ego*	b1513	-68	0.00770797	*yfgC*	b2494	-10,07
RdlC	16	*tam*	b1519	-68	0.00770797	*yrbE*	b3194	-10,06
RdlC	17	*manX*	b1817	-68	0.00770797	*gadY*	b4452	-9,99
RdlC	18	*bglF*	b3722	-68	0.00770797	*ydhC*	b1660	-9,98
RdlC	19	*yggP*	b4465	-68	0.00770797	*uspG*	b0607	-9,96
RdlC	20	*ybiF*	b0813	-67	0.00904841	*mobA*	b3857	-9,93
RdlC	21	*grpE*	b2614	-67	0.00904841	*tatC*	b3839	-9,81
RdlC	22	*glgS*	b3049	-67	0.00904841	*chbG*	b1733	-9,79
RdlC	23	*ymfL*	b1147	-66	0.0106207	*yffP*	b2447	-9,67
RdlC	24	*ycdJ*	b1009	-65	0.0124645	*ydaM*	b1341	-9,66
RdlC	25	*marA*	b1531	-65	0.0124645	*deaD*	b3162	-9,6
SgrS	1	*yabN*	b0069	-415	0	*sgrR*	b0069	-184,58
SgrS	2	*yhcP*	b3240	-114	3.94982e-05	*idi*	b2889	-15,36

SgrS	3	*ybiN*	b0807	-100	0.000289693	*yhdH*	b3253	-15,01
SgrS	4	b1364	b1364	-100	0.000289693	*ydeE*	b1534	-14,56
SgrS	5	*yjfL*	b4184	-100	0.000289693	*otsA*	b1896	-14,1
SgrS	6	*yahF*	b0320	-99	0.000333997	*dedD*	b2314	-13,58
SgrS	7	*yhcD*	b3216	-92	0.000904329	*hisD*	b2020	-13,42
SgrS	8	*ydeO*	b1499	-91	0.00104259	*lacZ*	b0344	-13,39
SgrS	9	*ptsO*	b3206	-91	0.00104259	*hycE*	b2721	-13,3
SgrS	10	*yagZ*	b0293	-90	0.00120197	*mioC*	b3742	-13,13
SgrS	11	*yecM*	b1875	-90	0.00120197	*ygeK*	b2856	-13,13
SgrS	12	*yhbO*	b3153	-90	0.00120197	*flgL*	b1083	-13,12
SgrS	13	*ptsI*	b2416	-89	0.00138569	*queF*	b2794	-13,01
SgrS	14	*ynbB*	b1409	-88	0.00159749	*mreB*	b3251	-12,94
SgrS	15	*murI*	b3967	-88	0.00159749	*fbp*	b4232	-12,87
SgrS	16	*paaJ*	b1397	-86	0.00212302	*yjdI*	b4126	-12,68
SgrS	17	*yiaW*	b3587	-85	0.00244736	*rfaP*	b3630	-12,6
SgrS	18	*ydaU*	b1359	-84	0.00282119	*alsE*	b4085	-12,56
SgrS	19	*yneG*	b1523	-84	0.00282119	*yghQ*	b2983	-12,52
SgrS	20	*yaeJ*	b0191	-83	0.00325202	*acnB*	b0118	-12,47
SgrS	21	*argC*	b3958	-83	0.00325202	*pflD*	b3951	-12,43
SgrS	22	*wcaD*	b2056	-81	0.00432067	*cynS*	b0340	-12,32
SgrS	23	*dedD*	b2314	-81	0.00432067	*cmtB*	b2934	-12,31
SgrS	24	*yfcZ*	b2343	-81	0.00432067	*yegD*	b2069	-12,22
SgrS	25	*gabP*	b2663	-81	0.00432067	*yfcC*	b2298	-12,21
RydB	1	*ymgD*	b1171	-92	0.000140544	*ymgD*	b1171	-25,59
RydB	2	*yagP*	b0282	-80	0.000988308	*insA*	b0022	-19,21
RydB	3	*yiaI*	b3573	-75	0.00222664	*fadI*	b2342	-18,14
RydB	4	*parC*	b3019	-74	0.0026192	*yncD*	b1451	-18,13
RydB	5	*yi21_3*	b1997	-70	0.00501267	*insA*	b0022	-18
RydB	6	*ygfX*	b2896	-69	0.00589498	*ddpX*	b1488	-17,91
RydB	7	*sgcA*	b4302	-69	0.00589498	*ulaF*	b4198	-17,53
RydB	8	*ddpX*	b1488	-68	0.00693204	*yjfC*	b4186	-17,36
RydB	9	*wbbJ*	b2033	-67	0.00815079	*ascF*	b2715	-16,76
RydB	10	*groS*	b4142	-65	0.0112649	*phoE*	b0241	-16,68
RydB	11	*efp*	b4147	-65	0.0112649	*vacJ*	b2346	-16,66
RydB	12	*ppiA*	b3363	-62	0.0182811	*yihG*	b3862	-16,43
RydB	13	*ygfZ*	b2898	-61	0.0214735	*hokA*	b4455	-16,39
RydB	14	*citT*	b0612	-60	0.0252162	*yjbR*	b4057	-16,37
RydB	15	*yebY*	b1839	-60	0.0252162	*lplA*	b4386	-16,3
RydB	16	*yjgJ*	b4251	-60	0.0252162	*insA*	b0022	-15,63
RydB	17	*sgcE*	b4301	-60	0.0252162	*mokA*	b4647	-15,55
RydB	18	*pdhR*	b0113	-58	0.0347352	*rsxB*	b1628	-15,42
RydB	19	*fhiA*	b0229	-58	0.0347352	*ddpD*	b1484	-14,99
RydB	20	*cyoD*	b0429	-58	0.0347352	*phnN*	b4094	-14,91
RydB	21	*ybaL*	b0478	-58	0.0347352	*ygfX*	b2896	-14,8

RydB	22	*speF*	b0693	-58	0.0347352	*amtB*	b0451	-14,69
RydB	23	*fliI*	b1941	-58	0.0347352	*ybjS*	b0868	-14,66
RydB	24	*xdhA*	b2866	-58	0.0347352	*yoaE*	b1816	-14,63
RydB	25	*intD*	b0537	-57	0.0407407	*mmuM*	b0261	-14,56
SokC	1	*mokC*	b0018	-255	3.33067e-16	*hokC*	b4412	-60,23
SokC	2	*hokC*	b4412	-255	3.33067e-16	*mokC*	b0018	-56,3
SokC	3	*mokB*	b1420	-104	1.86197e-05	*hokB*	b4428	-16,12
SokC	4	*hokB*	b4428	-104	1.86197e-05	*suhB*	b2533	-14,27
SokC	5	*hokE*	b4415	-101	3.03857e-05	*ygdI*	b2809	-13,6
SokC	6	*atoC*	b2220	-81	0.000795234	*mokB*	b1420	-13,56
SokC	7	*hdeA*	b3510	-76	0.00179794	*yahG*	b0321	-13,19
SokC	8	*ribB*	b3041	-69	0.00562649	*ruvB*	b1860	-12,76
SokC	9	*ycdJ*	b1009	-68	0.00662095	*glnQ*	b0809	-12,14
SokC	10	*mtr*	b3161	-67	0.00779048	*mfd*	b1114	-12,11
SokC	11	*fhlA*	b2731	-66	0.00916565	*ybgQ*	b0718	-11,81
SokC	12	*yddB*	b1495	-65	0.0107822	*sapF*	b1290	-11,73
SokC	13	*cusB*	b0574	-64	0.0126821	*tap*	b1885	-11,72
SokC	14	*yiiU*	b3928	-64	0.0126821	*glnA*	b3870	-11,58
SokC	15	*ydcV*	b1443	-63	0.0149142	*ribC*	b1662	-11,58
SokC	16	*btuD*	b1709	-63	0.0149142	*yafO*	b0233	-11,58
SokC	17	*ygjD*	b3064	-63	0.0149142	*yibD*	b3615	-11,54
SokC	18	*yfdG*	b2350	-62	0.0175357	*dnaG*	b3066	-11,44
SokC	19	*fdrA*	b0518	-61	0.0206131	*kbl*	b3617	-11,32
SokC	20	*ftsX*	b3462	-61	0.0206131	*fruK*	b2168	-11,32
SokC	21	*yeiI*	b2160	-60	0.0242239	*bcsC*	b3530	-11,11
SokC	22	*yahO*	b0329	-59	0.028458	*pdhR*	b0113	-11,05
SokC	23	b2191	b2191	-59	0.028458	*yjgH*	b4248	-11
SokC	24	*yfbP*	b2275	-59	0.028458	*eutH*	b2452	-10,99
SokC	25	*yphD*	b2546	-59	0.028458	*dinG*	b0799	-10,81
SibD	1	*ydcH*	b1426	-84	0.00147847	*thiK*	b1106	-14,8
SibD	2	*nrdG*	b4237	-82	0.00199535	*mdtJ*	b1600	-12,98
SibD	3	*pdxY*	b1636	-79	0.00312789	*ybfK*	b4590	-12,62
SibD	4	*yliI*	b0837	-78	0.0036333	*setB*	b2170	-12,5
SibD	5	*yrbA*	b3190	-78	0.0036333	*yjbQ*	b4056	-12,29
SibD	6	*artJ*	b0860	-75	0.00569286	*rplV*	b3315	-11,93
SibD	7	*dmsB*	b0895	-75	0.00569286	*tyrR*	b1323	-11,92
SibD	8	*setB*	b2170	-75	0.00569286	*yfjH*	b2623	-11,85
SibD	9	*hyfE*	b2485	-74	0.00661134	*yeaP*	b1794	-11,64
SibD	10	*yfhL*	b2562	-74	0.00661134	*prpE*	b0335	-11,41
SibD	11	*ychP*	b1220	-72	0.00891467	*kgtP*	b2587	-11,22
SibD	12	*rrmB*	b3289	-72	0.00891467	*xthA*	b1749	-11,14
SibD	13	*rarD*	b3819	-72	0.00891467	*yhbQ*	b3155	-11,11
SibD	14	*yfbQ*	b2290	-71	0.0103502	*rpsI*	b3230	-11,06

SibD	15	*ygaD*	b2700	-71	0.0103502	*dctA*	b3528	-11,06
SibD	16	*fxsA*	b4140	-71	0.0103502	*ycjP*	b1312	-10,9
SibD	17	*yiaB*	b3563	-70	0.0120156	*narL*	b1221	-10,84
SibD	18	*yiaO*	b3579	-70	0.0120156	*zapA*	b2910	-10,7
SibD	19	*ilvL*	b3766	-70	0.0120156	*yfgM*	b2513	-10,61
SibD	20	*ytfF*	b4210	-70	0.0120156	*tesA*	b0494	-10,6
SibD	21	*holC*	b4259	-70	0.0120156	*yqjC*	b3097	-10,6
SibD	22	*hokE*	b4415	-70	0.0120156	*artJ*	b0860	-10,52
SibD	23	*yaeL*	b0176	-69	0.013947	*poxB*	b0871	-10,48
SibD	24	*yeaC*	b1777	-69	0.013947	*yeaC*	b1777	-10,43
SibD	25	*mazG*	b2781	-69	0.013947	*yebY*	b1839	-10,33
SibB	1	*ycjP*	b1312	-90	0.000538377	*mhpD*	b0350	-13,66
SibB	2	*moaA*	b0781	-88	0.000728501	*yjjY*	b4402	-13,48
SibB	3	*hyfB*	b2482	-86	0.000985733	*xylF*	b3566	-13,27
SibB	4	*htpG*	b0473	-81	0.00209884	*fimG*	b4319	-12,9
SibB	5	*gcvP*	b2903	-81	0.00209884	*hyfB*	b2482	-12,48
SibB	6	*gidB*	b3740	-77	0.00384035	*kdsB*	b0918	-12,32
SibB	7	*ybfL*	b0705	-76	0.00446609	*yigM*	b3827	-12,3
SibB	8	*ydgI*	b1605	-76	0.00446609	*yeeE*	b2013	-11,71
SibB	9	*ybjM*	b0848	-73	0.00702178	*ppx*	b2502	-11,68
SibB	10	*yfeX*	b2431	-72	0.00816376	*mltB*	b2701	-11,41
SibB	11	*yhfZ*	b3383	-72	0.00816376	*yneH*	b1524	-11,1
SibB	12	*ygdR*	b2833	-71	0.00949058	*tonB*	b1252	-11,04
SibB	13	b4285	b4285	-71	0.00949058	*fliA*	b1922	-11,04
SibB	14	*yacA*	b0097	-70	0.0110318	*spy*	b1743	-10,96
SibB	15	*nikE*	b3480	-70	0.0110318	*melA*	b4119	-10,95
SibB	16	*ygiN*	b3029	-69	0.0128218	*ybgC*	b0736	-10,93
SibB	17	*pdxB*	b2320	-68	0.0148999	*ybgF*	b0742	-10,76
SibB	18	*yjjK*	b4391	-68	0.0148999	*ssuE*	b0937	-10,72
SibB	19	*glnS*	b0680	-66	0.0201104	*yohJ*	b2141	-10,69
SibB	20	*yfcY*	b2342	-66	0.0201104	*frlR*	b3375	-10,68
SibB	21	*yjjY*	b4402	-66	0.0201104	*ahpF*	b0606	-10,56
SibB	22	*fepB*	b0592	-65	0.0233558	*rutR*	b1013	-10,53
SibB	23	*sapC*	b1292	-65	0.0233558	*lsrB*	b1516	-10,52
SibB	24	*pfs*	b0159	-64	0.0271177	*mtn*	b0159	-10,51
SibB	25	*ybcT*	b0556	-64	0.0271177	*insE*	b0298	-10,51
MicC	1	*ompC*	b2215	-101	6.54169e-05	*ydaQ*	b1346	-18,2
MicC	2	*ydeJ*	b1537	-100	7.64346e-05	*rsmF*	b1835	-17,77
MicC	3	*yniA*	b1725	-97	0.000121923	*rdgC*	b0393	-15,78
MicC	4	*bacA*	b3057	-97	0.000121923	*rraB*	b4255	-15,12
MicC	5	*wbbJ*	b2033	-85	0.000789167	*pntB*	b1602	-14,66
MicC	6	*yahM*	b0327	-80	0.00171782	*rfaL*	b3622	-14,23
MicC	7	*yhfN*	b3371	-80	0.00171782	*thrA*	b0002	-14,15

MicC	8	*xylE*	b4031	-80	0.00171782	*fiu*	b0805	-13,84
MicC	9	*ymgB*	b1166	-79	0.00200686	*dgoD*	b4478	-13,71
MicC	10	*cybB*	b1418	-78	0.00234448	*yfdK*	b2354	-13,6
MicC	11	*ybfH*	b0691	-76	0.00319937	*dedD*	b2314	-13,54
MicC	12	*allD*	b0517	-75	0.00373723	*can*	b0126	-13,47
MicC	13	*yehV*	b2127	-75	0.00373723	*yjiL*	b4334	-13,35
MicC	14	*yhbX*	b3173	-75	0.00373723	*dkgA*	b3012	-13,35
MicC	15	*yaiD*	b0393	-74	0.00436531	*skp*	b0178	-13,33
MicC	16	*pepN*	b0932	-74	0.00436531	*yrbC*	b3192	-13,3
MicC	17	*ycgY*	b1196	-74	0.00436531	*yhjJ*	b3527	-13,13
MicC	18	*ydeH*	b1535	-74	0.00436531	*cbl*	b1987	-13,11
MicC	19	*yebU*	b1835	-73	0.00509868	*abgR*	b1339	-13,04
MicC	20	*holE*	b1842	-73	0.00509868	*coaE*	b0103	-12,99
MicC	21	*dkgA*	b3012	-73	0.00509868	*cyoD*	b0429	-12,92
MicC	22	*rplK*	b3983	-73	0.00509868	*ygeN*	b2858	-12,92
MicC	23	*zur*	b4046	-73	0.00509868	*ompW*	b1256	-12,91
MicC	24	*fruR*	b0080	-72	0.00595488	*ycgL*	b1179	-12,79
MicC	25	*yceL*	b1065	-72	0.00595488	*tyrS*	b1637	-12,72
GlmY	1	*uvrB*	b0779	-80	0.00358406	*yoaB*	b1809	-11,95
GlmY	2	*yccE*	b1001	-79	0.00414817	*acrF*	b3266	-11,78
GlmY	3	*lpdA*	b0116	-76	0.00642944	*setC*	b3659	-11,53
GlmY	4	*fbaA*	b2925	-76	0.00642944	*srmB*	b2576	-11,49
GlmY	5	*mltE*	b1193	-75	0.00743973	*yiaF*	b3554	-11,34
GlmY	6	*ydcC*	b1460	-75	0.00743973	*yjjQ*	b4365	-11,32
GlmY	7	*ftn*	b1905	-75	0.00743973	*mdtB*	b2075	-11,21
GlmY	8	*sdaC*	b2796	-75	0.00743973	*ftsA*	b0094	-11,17
GlmY	9	*ftsA*	b0094	-74	0.00860809	*yjiK*	b4333	-11,11
GlmY	10	*ykfJ*	b0235	-74	0.00860809	*proL*	b2189	-10,95
GlmY	11	*yajO*	b0419	-74	0.00860809	*galS*	b2151	-10,94
GlmY	12	*yeaU*	b1800	-74	0.00860809	*mgrB*	b1826	-10,92
GlmY	13	*ygfB*	b2909	-74	0.00860809	*cho*	b1741	-10,87
GlmY	14	*yehX*	b2129	-72	0.0115207	*gph*	b3385	-10,52
GlmY	15	*hscA*	b2526	-72	0.0115207	*yccJ*	b1003	-10,52
GlmY	16	*uppS*	b0174	-71	0.0133256	*citE*	b0616	-10,48
GlmY	17	*ybeR*	b0645	-71	0.0133256	*ydgJ*	b1624	-10,33
GlmY	18	b0725	b0725	-71	0.0133256	*narL*	b1221	-10,31
GlmY	19	*tolB*	b0740	-71	0.0133256	*nagB*	b0678	-10,29
GlmY	20	*dsbB*	b1185	-71	0.0133256	*kduI*	b2843	-10,29
GlmY	21	*argC*	b3958	-71	0.0133256	*amtB*	b0451	-10,25
GlmY	22	*phnO*	b4093	-71	0.0133256	*fldA*	b0684	-10,21
GlmY	23	*amtB*	b0451	-70	0.0154111	*yjaA*	b4011	-10,2
GlmY	24	*uvrD*	b3813	-70	0.0154111	*yghD*	b2968	-10,12
GlmY	25	*murD*	b0088	-69	0.01782	*ycfH*	b1100	-10,02

SraD	1	*ygaG*	b2687	-255	5.55112e-16	*luxS*	b2687	-80,52
SraD	2	*fhuF*	b4367	-103	2.67807e-05	*map*	b0168	-15,22
SraD	3	*yegX*	b2102	-82	0.000792172	*yaiT*	b4580	-15,04
SraD	4	*emrK*	b2368	-82	0.000792172	*citE*	b0616	-14,68
SraD	5	*yebO*	b1825	-80	0.00109362	*glyA*	b2551	-14,52
SraD	6	*ygbQ*	b2748	-80	0.00109362	*truD*	b2745	-14,52
SraD	7	*ymgD*	b1171	-77	0.00177371	*paaH*	b1395	-14,39
SraD	8	*ydfE*	b1577	-76	0.00208387	*ompA*	b0957	-14,24
SraD	9	b2856	b2856	-75	0.0024482	*rhlE*	b0797	-13,56
SraD	10	*ybgI*	b0710	-74	0.00287613	*nrdA*	b2234	-13,51
SraD	11	*yegH*	b2063	-74	0.00287613	*cusB*	b0574	-13,34
SraD	12	*araC*	b0064	-73	0.00337874	*ivy*	b0220	-13,2
SraD	13	*ycjD*	b1289	-73	0.00337874	*dgkA*	b4042	-13,14
SraD	14	*entD*	b0583	-71	0.00466215	*pagP*	b0622	-13,1
SraD	15	*phoQ*	b1129	-70	0.00547601	*ydiH*	b1685	-12,96
SraD	16	*phoP*	b1130	-70	0.00547601	*phoB*	b0399	-12,9
SraD	17	*ynhG*	b1678	-70	0.00547601	*amiC*	b2817	-12,75
SraD	18	*ygbO*	b2745	-69	0.00643148	*yphH*	b2550	-12,71
SraD	19	*alsA*	b4087	-69	0.00643148	*ycjD*	b1289	-12,69
SraD	20	*yadL*	b0137	-68	0.00755303	*ompR*	b3405	-12,66
SraD	21	*ybeU*	b0648	-68	0.00755303	*pgk*	b2926	-12,64
SraD	22	*yceF*	b1087	-67	0.00886929	*secF*	b0409	-12,59
SraD	23	*ydjE*	b1769	-67	0.00886929	*yjcH*	b4068	-12,57
SraD	24	*yicJ*	b3657	-67	0.00886929	*entC*	b0593	-12,39
SraD	25	*fimD*	b4317	-67	0.00886929	*wzc*	b2060	-12,38

C0465	1	*tar*	b1886	-230	2.73115e-14	*tar*	b1886	-72,62
C0465	2	*yebQ*	b1828	-87	0.000325958	*vsr*	b1960	-21,01
C0465	3	*ycdO*	b1018	-73	0.00315523	*ushA*	b0480	-19,1
C0465	4	*rpmH*	b3703	-72	0.00371	*rdgB*	b2954	-18,23
C0465	5	*purT*	b1849	-70	0.00512851	*pmrD*	b2259	-17,72
C0465	6	b2337	b2337	-69	0.00602917	*elaA*	b2267	-17,58
C0465	7	*ybcC*	b0539	-68	0.00708745	*tag*	b3549	-17,52
C0465	8	*fixA*	b0041	-67	0.0083307	*yahC*	b0317	-17,44
C0465	9	*allR*	b0506	-67	0.0083307	*rfaD*	b3619	-17,37
C0465	10	*ydcY*	b1446	-67	0.0083307	*tatA*	b3836	-17,32
C0465	11	*ydiF*	b1694	-67	0.0083307	*hcaD*	b2542	-17,28
C0465	12	*yeiB*	b2152	-67	0.0083307	*feaR*	b1384	-17,21
C0465	13	*ycdU*	b1029	-66	0.00979096	*baeS*	b2078	-17,09
C0465	14	*ycaD*	b0898	-65	0.0115057	*yigZ*	b3848	-16,94
C0465	15	*recC*	b2822	-65	0.0115057	*potD*	b1123	-16,72
C0465	16	*acpD*	b1412	-64	0.0135187	*agaR*	b3131	-16,72
C0465	17	*uidA*	b1617	-64	0.0135187	*ves*	b1742	-16,64
C0465	18	*yjdE*	b4115	-64	0.0135187	*glnB*	b2553	-16,44
C0465	19	*yffH*	b2467	-63	0.015881	*rplF*	b3305	-16,32

C0465	20	*ybbY*	b0513	-62	0.0186522	*yfiQ*	b2584	-16,31
C0465	21	*rluC*	b1086	-62	0.0186522	*yhiR*	b3499	-16,26
C0465	22	*ydbC*	b1406	-62	0.0186522	*ydhL*	b1648	-16,23
C0465	23	*baeS*	b2078	-62	0.0186522	*sufS*	b1680	-16,19
C0465	24	*pmrD*	b2259	-62	0.0186522	*ybhC*	b0772	-16,06
C0465	25	*yjeI*	b4144	-62	0.0186522	*yeiA*	b2147	-16,03
C0719	1	*yghK*	b2975	-360	0	*glcA*	b2975	-89,97
C0719	2	*yhcM*	b3232	-96	0.000619344	*aceA*	b4015	-18,93
C0719	3	*yidP*	b3684	-96	0.000619344	*ygbN*	b2740	-16,26
C0719	4	*pgk*	b2926	-95	0.000712631	*yehS*	b2124	-16,21
C0719	5	*yneB*	b1517	-91	0.00124899	*fliL*	b1944	-16,2
C0719	6	*zipA*	b2412	-90	0.00143705	*feoC*	b3410	-16,07
C0719	7	*rpsM*	b3298	-89	0.00165339	*leuO*	b0076	-15,9
C0719	8	*ydaS*	b1357	-88	0.00190228	*dcd*	b2065	-15,03
C0719	9	*yhcR*	b3242	-88	0.00190228	*glmS*	b3729	-14,8
C0719	10	*cysE*	b3607	-87	0.0021886	*hycD*	b2722	-14,7
C0719	11	*idnR*	b4264	-87	0.0021886	*yihF*	b3861	-14,5
C0719	12	*yehS*	b2124	-85	0.00289679	*glnS*	b0680	-14,27
C0719	13	*yhhZ*	b3442	-85	0.00289679	*mglC*	b2148	-14,22
C0719	14	*yjeJ*	b4145	-85	0.00289679	*rutE*	b1008	-14,18
C0719	15	*ybdQ*	b0607	-84	0.00333254	*yfcQ*	b2334	-13,9
C0719	16	*citG*	b0613	-84	0.00333254	*yfdS*	b2362	-13,75
C0719	17	*mglC*	b2148	-84	0.00333254	*ilvG*	b4488	-13,71
C0719	18	*yiaV*	b3586	-84	0.00333254	*yfiQ*	b2584	-13,66
C0719	19	*ycbF*	b0944	-82	0.00441007	*yfdV*	b2372	-13,61
C0719	20	*yecE*	b1868	-82	0.00441007	*ubiB*	b3835	-13,48
C0719	21	*lysA*	b2838	-82	0.00441007	*manX*	b1817	-13,33
C0719	22	*yfcP*	b2333	-81	0.00507287	*dgoT*	b3691	-13,27
C0719	23	*yghG*	b2971	-81	0.00507287	*bioH*	b3412	-13,12
C0719	24	*ygiP*	b3060	-81	0.00507287	*yoeB*	b4539	-13,04
C0719	25	*nhaA*	b0019	-80	0.005835	*ydhY*	b1674	-12,81
IS128	1	*yjfI*	b4181	-116	3.42357e-05	*nrdG*	b4237	-18,07
IS128	2	*wbbL*	b2031	-108	0.000105863	*yajL*	b0424	-16,34
IS128	3	*caiF*	b0034	-106	0.00014038	*yebQ*	b1828	-16,29
IS128	4	*yhcN*	b3238	-95	0.000662712	*ivy*	b0220	-14,6
IS128	5	*ybgP*	b0717	-94	0.000763111	*ynbD*	b1411	-14,26
IS128	6	*ydbD*	b1407	-94	0.000763111	*yjbI*	b4038	-14,03
IS128	7	*yhbC*	b3170	-93	0.000878714	*yneJ*	b1526	-13,83
IS128	8	*yhiL*	b3490	-93	0.000878714	*yeaX*	b1803	-13,74
IS128	9	*gspA*	b3323	-91	0.00116508	*argE*	b3957	-13,48
IS128	10	*aqpZ*	b0875	-90	0.00134153	*rfbB*	b2041	-13,38
IS128	11	*yneJ*	b1526	-90	0.00134153	*prmC*	b1212	-13,29
IS128	12	*yfjG*	b2619	-90	0.00134153	*caiA*	b0039	-13,26

IS128	13	*osmC*	b1482	-89	0.00154469	*caiF*	b0034	-13,21
IS128	14	*yafC*	b0208	-88	0.00177859	*iscX*	b2524	-12,97
IS128	15	*cyoA*	b0432	-87	0.00204787	*feaB*	b1385	-12,8
IS128	16	*ymcC*	b0986	-87	0.00204787	*ybdM*	b0601	-12,73
IS128	17	*ycdF*	b1005	-86	0.00235787	*yffQ*	b2448	-12,56
IS128	18	*sppA*	b1766	-86	0.00235787	*ythA*	b4655	-12,5
IS128	19	*ygeR*	b2865	-86	0.00235787	*yadH*	b0128	-12,49
IS128	20	*ybeQ*	b0644	-85	0.00271473	*kup*	b3747	-12,47
IS128	21	*yfcV*	b2339	-85	0.00271473	*rfbD*	b2040	-12,44
IS128	22	*yhhZ*	b3442	-85	0.00271473	*yqiB*	b3033	-12,43
IS128	23	*ybiT*	b0820	-84	0.00312552	*ymfA*	b1122	-12,28
IS128	24	*wcaE*	b2055	-84	0.00312552	*ydgH*	b1604	-12,25
IS128	25	*yehU*	b2126	-84	0.00312552	*narI*	b1227	-12,23
[redacted]								
SroB	2	*ydhP*	b1657	-92	0.000184807	*chbC*	b1737	-15,71
SroB	3	*ybhD*	b0768	-89	0.000298285	*rimL*	b1427	-14,61
SroB	4	*ascG*	b2714	-82	0.000911356	*lplT*	b2835	-12,7
SroB	5	b0501	b0501	-81	0.00106897	*hisG*	b2019	-12,65
SroB	6	*yiiG*	b3896	-81	0.00106897	*agaV*	b3133	-12,51
SroB	7	*gdhA*	b1761	-80	0.00125383	*cysS*	b0526	-12,38
SroB	8	*putA*	b1014	-79	0.00147063	*uxuA*	b4322	-11,67
SroB	9	*ycbW*	b0946	-77	0.00202307	*dpiB*	b0619	-11,59
SroB	10	*aroH*	b1704	-77	0.00202307	*srlD*	b2705	-11,3
SroB	11	*ydeN*	b1498	-75	0.00278272	*hscB*	b2527	-11,11
SroB	12	*spr*	b2175	-74	0.00326346	*hflC*	b4175	-11,03
SroB	13	*ppiD*	b0441	-73	0.00382709	*eutM*	b2457	-10,98
SroB	14	*celB*	b1737	-73	0.00382709	*yfgM*	b2513	-10,94
SroB	15	*tdcA*	b3118	-73	0.00382709	*hupB*	b0440	-10,92
SroB	16	*yiaT*	b3584	-73	0.00382709	*hlyE*	b1182	-10,89
SroB	17	*ycbQ*	b0938	-72	0.00448784	*fabG*	b1093	-10,87
SroB	18	*yciI*	b1251	-72	0.00448784	*zapA*	b2910	-10,87
SroB	19	*exbB*	b3006	-72	0.00448784	*dusB*	b3260	-10,84
SroB	20	*ybbC*	b0498	-70	0.00617015	*ybcO*	b0549	-10,75
SroB	21	*yciT*	b1284	-70	0.00617015	*ygdQ*	b2832	-10,74
SroB	22	*ymcC*	b0986	-69	0.00723396	*ybjK*	b0846	-10,5
SroB	23	*cbl*	b1987	-69	0.00723396	*yodC*	b1957	-10,49
SroB	24	b2856	b2856	-69	0.00723396	*yphC*	b2545	-10,44
SroB	25	*yihF*	b3861	-69	0.00723396	*psrN*	b4448	-10,43
SroC	1	*gltJ*	b0654	-85	0.00159728	*exuR*	b3094	-14,92
SroC	2	*ynaJ*	b1332	-82	0.00248416	*yieE*	b3712	-14,21
SroC	3	*ycbW*	b0946	-81	0.00287801	*htpG*	b0473	-13,23
SroC	4	b3004	b3004	-81	0.00287801	*mtfA*	b1976	-12,83
SroC	5	*gutM*	b2706	-77	0.00518299	*ycfM*	b1105	-12,54

SroC	6	*yggL*	b2959	-77	0.00518299	*sfsB*	b3188	-12,43
SroC	7	*yahI*	b0323	-76	0.00600343	*hisH*	b2023	-12,32
SroC	8	*ybaK*	b0481	-76	0.00600343	*ybdF*	b0579	-12,23
SroC	9	*ydjM*	b1728	-75	0.00695328	*yphD*	b2546	-11,92
SroC	10	*yecM*	b1875	-74	0.00805281	*wcaE*	b2055	-11,83
SroC	11	*ygdL*	b2812	-74	0.00805281	*hisQ*	b2308	-11,73
SroC	12	*fxsA*	b4140	-74	0.00805281	*ydhW*	b1672	-11,52
SroC	13	*wcaE*	b2055	-73	0.00932538	*yfiM*	b2586	-11,49
SroC	14	*nlp*	b3188	-73	0.00932538	*asr*	b1597	-11,28
SroC	15	*ybeD*	b0631	-72	0.010798	*uof*	b4637	-11,28
SroC	16	*mscL*	b3291	-72	0.010798	*mcrC*	b4345	-11,2
SroC	17	*ompR*	b3405	-72	0.010798	*yeeD*	b2012	-11,15
SroC	18	*fabF*	b1095	-71	0.0125016	*yejE*	b2179	-11,06
SroC	19	*fdx*	b2525	-71	0.0125016	*thiG*	b3991	-10,94
SroC	20	*yjgY*	b4276	-71	0.0125016	*yfiC*	b2575	-10,93
SroC	21	*ybaJ*	b0461	-70	0.0144721	*ryjA*	b4459	-10,88
SroC	22	*wbbH*	b2035	-70	0.0144721	*ygfI*	b2921	-10,71
SroC	23	*ykfC*	b0258	-69	0.0167505	*ybaN*	b0468	-10,58
SroC	24	*kdpA*	b0698	-69	0.0167505	*pyrE*	b3642	-10,57
SroC	25	*ycfM*	b1105	-69	0.0167505	*rplU*	b3186	-10,39
SroD	1	*sapB*	b1293	-90	0.00028535	*thiQ*	b0066	-15,24
SroD	2	*fdnI*	b1476	-83	0.000864069	*sapB*	b1293	-14,88
SroD	3	*kdsB*	b0918	-76	0.00261495	*ulaB*	b4194	-14,53
SroD	4	*rrmA*	b1822	-76	0.00261495	*yqfE*	b2915	-14,05
SroD	5	b2681	b2681	-76	0.00261495	*ddpA*	b1487	-13,67
SroD	6	*sgaB*	b4194	-74	0.00358728	*yhhX*	b3440	-13,46
SroD	7	*dld*	b2133	-72	0.00492026	*yafK*	b0224	-12,89
SroD	8	*dkgB*	b0207	-70	0.00674687	*lon*	b0439	-12,73
SroD	9	*yahL*	b0326	-70	0.00674687	*yafY*	b0251	-12,72
SroD	10	*yceO*	b1058	-70	0.00674687	*fdnI*	b1476	-12,61
SroD	11	*wcaB*	b2058	-70	0.00674687	*sspB*	b3228	-12,58
SroD	12	*pitA*	b3493	-70	0.00674687	*lipB*	b0630	-12,23
SroD	13	*aslB*	b3800	-70	0.00674687	*fliL*	b1944	-12,09
SroD	14	*ybjH*	b0843	-69	0.00789964	*ygeW*	b2870	-11,76
SroD	15	*tyrP*	b1907	-69	0.00789964	*yggS*	b2951	-11,75
SroD	16	*zwf*	b1852	-68	0.00924844	*nuoI*	b2281	-11,74
SroD	17	*yheU*	b3354	-68	0.00924844	*eptB*	b3546	-11,72
SroD	18	*nlpA*	b3661	-68	0.00924844	*mdtE*	b3513	-11,54
SroD	19	*fhiA*	b0229	-67	0.0108263	*yihF*	b3861	-11,49
SroD	20	*ydeK*	b1510	-67	0.0108263	*ltaE*	b0870	-11,44
SroD	21	*yhaL*	b3107	-67	0.0108263	*rutA*	b1012	-11,38
SroD	22	*yrbK*	b3199	-66	0.0126716	*pitB*	b2987	-11,31
SroD	23	*fdoH*	b3893	-66	0.0126716	*chiA*	b3338	-11,24
SroD	24	*rne*	b1084	-65	0.0148291	*mhpB*	b0348	-11,22

SroD	25	*udk*	b2066	-65	0.0148291	*fepB*	b0592	-11,15
SroE	1	*leuA*	b0074	-96	0.000196542	*fixA*	b0041	-11,43
SroE	2	*tolQ*	b0737	-86	0.000901083	*metE*	b3829	-10,86
SroE	3	*rpmJ*	b3299	-77	0.0035441	*mnmG*	b3741	-10,03
SroE	4	*yhfN*	b3371	-76	0.00412596	*artJ*	b0860	-9,01
SroE	5	*yiiF*	b3890	-75	0.00480312	*pldB*	b3825	-8,69
SroE	6	*yjeM*	b4156	-74	0.00559111	*sppA*	b1766	-8,62
SroE	7	*ycjX*	b1321	-69	0.0119355	*yehW*	b2128	-8,57
SroE	8	*lepA*	b2569	-69	0.0119355	*gspK*	b3332	-8,55
SroE	9	*radC*	b3638	-69	0.0119355	*tolQ*	b0737	-8,45
SroE	10	*trs5_6*	b1994	-68	0.0138854	*ogrK*	b2082	-8,33
SroE	11	*yafJ*	b0223	-67	0.0161513	*kduI*	b2843	-8,2
SroE	12	*ymfK*	b1145	-67	0.0161513	*yddE*	b1464	-7,96
SroE	13	*ybeV*	b0649	-66	0.0187833	*metB*	b3939	-7,74
SroE	14	*iclR*	b4018	-66	0.0187833	*hemL*	b0154	-7,73
SroE	15	*prpR*	b0330	-65	0.0218396	*yhhT*	b3474	-7,68
SroE	16	*yaaW*	b0011	-64	0.0253866	*mdtA*	b2074	-7,54
SroE	17	*yraM*	b3147	-64	0.0253866	*yhcG*	b3220	-7,5
SroE	18	*yrhB*	b3446	-64	0.0253866	*paaB*	b1389	-7,49
SroE	19	*kdgT*	b3909	-64	0.0253866	*yajG*	b0434	-7,46
SroE	20	*basS*	b4112	-64	0.0253866	*chiA*	b3338	-7,43
SroE	21	*yi22_1*	b0361	-63	0.029501	*ycgE*	b1162	-7,14
SroE	22	*ycgI*	b1173	-63	0.029501	*creA*	b4397	-7,14
SroE	23	*yi22_2*	b1402	-63	0.029501	*fadJ*	b2341	-6,84
SroE	24	*yi22_3*	b1996	-63	0.029501	*tesA*	b0494	-6,84
SroE	25	*yi22_4*	b2860	-63	0.029501	*dxr*	b0173	-6,73
SroH	1	*yafM*	b0228	-110	4.34186e-05	*yfdE*	b2371	-14,02
SroH	2	*yaeL*	b0176	-97	0.000292136	*ymgG*	b1172	-13,8
SroH	3	*caiE*	b0035	-87	0.00126552	*fliR*	b1950	-13,66
SroH	4	*intD*	b0537	-86	0.00146526	*yjgR*	b4263	-13,49
SroH	5	*fliR*	b1950	-85	0.0016965	*pnuC*	b0751	-13,05
SroH	6	*modC*	b0765	-79	0.00408481	*atoS*	b2219	-12,95
SroH	7	*ymfO*	b1151	-79	0.00408481	*nrdG*	b4237	-12,91
SroH	8	*yfaX*	b2248	-79	0.00408481	*rplM*	b3231	-12,88
SroH	9	*appC*	b0978	-78	0.00472847	*afuB*	b0263	-12,59
SroH	10	*ribF*	b0025	-77	0.00547328	*yafD*	b0209	-12,45
SroH	11	*glgS*	b3049	-77	0.00547328	*paaG*	b1394	-12,39
SroH	12	*yhfK*	b3358	-77	0.00547328	*yjdO*	b4559	-12,36
SroH	13	*metI*	b0198	-75	0.00733197	*bioA*	b0774	-12,23
SroH	14	*glxR*	b0509	-75	0.00733197	*ygcS*	b2771	-12
SroH	15	*yijF*	b3944	-75	0.00733197	*cynS*	b0340	-11,83
SroH	16	*adk*	b0474	-74	0.00848513	*ydjX*	b1750	-11,8
SroH	17	*flgJ*	b1081	-73	0.00981875	*mutS*	b2733	-11,8

SroH	18	*yciU*	b1248	-73	0.00981875	*dmsD*	b1591	-11,8
SroH	19	*ydcE*	b1461	-73	0.00981875	*hpf*	b3203	-11,76
SroH	20	*ygcO*	b2767	-73	0.00981875	*panF*	b3258	-11,75
SroH	21	*yaaA*	b0006	-72	0.0113608	*rtcR*	b3422	-11,74
SroH	22	*zwf*	b1852	-72	0.0113608	*csgE*	b1039	-11,71
SroH	23	*yeeU*	b2004	-72	0.0113608	*ynjD*	b1756	-11,67
SroH	24	*ygcI*	b2757	-72	0.0113608	*ynbD*	b1411	-11,65
SroH	25	*speA*	b2938	-72	0.0113608	*rseP*	b0176	-11,64
Istr2	1	*tisA*	b4404	-122	5.58406e-06	*tisB*	b4618	-21,41
Istr2	2	*tisB*	b4405	-122	5.58406e-06	*ynhF*	b4602	-15,84
Istr2	3	*ybhA*	b0766	-103	9.4782e-05	*csdA*	b2810	-15,75
Istr2	4	*cobB*	b1120	-92	0.000488233	*ylbF*	b0520	-14,71
Istr2	5	*yabP*	b0056	-91	0.000566678	*cobB*	b1120	-14,3
Istr2	6	*def*	b3287	-89	0.000763389	*yehS*	b2124	-13,43
Istr2	7	*rnfD*	b1630	-86	0.00119352	*thyA*	b2827	-12,14
Istr2	8	*ppx*	b2502	-86	0.00119352	*mhpC*	b0349	-11,98
Istr2	9	*rplW*	b3318	-86	0.00119352	*cusS*	b0570	-11,97
Istr2	10	*ydiP*	b1696	-85	0.00138521	*dacA*	b0632	-11,87
Istr2	11	*zraS*	b4003	-84	0.00160765	*thiH*	b3990	-11,72
Istr2	12	*ykgB*	b0301	-83	0.00186579	*sdhA*	b0723	-11,53
Istr2	13	*ylbF*	b0520	-80	0.00291616	*motA*	b1890	-11,37
Istr2	14	*yieN*	b3746	-78	0.00392685	*sfmA*	b0530	-11,32
Istr2	15	*ampG*	b0433	-77	0.00455652	*yedA*	b1959	-11,27
Istr2	16	*ygjU*	b3089	-77	0.00455652	*ampG*	b0433	-11,2
Istr2	17	*sohB*	b1272	-76	0.00528688	*yffR*	b2449	-11,11
Istr2	18	*ispF*	b2746	-76	0.00528688	*typA*	b3871	-11,03
Istr2	19	*rpsI*	b3230	-75	0.00613396	*fucK*	b2803	-11,03
Istr2	20	*mdlB*	b0449	-73	0.00825524	*mdtK*	b1663	-10,97
Istr2	21	*ycfK*	b1154	-73	0.00825524	*mdtE*	b3513	-10,93
Istr2	22	*folX*	b2303	-73	0.00825524	*insX*	b4505	-10,88
Istr2	23	*cysU*	b2424	-73	0.00825524	*argW*	b2348	-10,68
Istr2	24	*rfaP*	b3630	-73	0.00825524	*rsmD*	b3465	-10,67
Istr2	25	*murG*	b0090	-72	0.00957562	*ygeX*	b2871	-10,65
Tp2	1	*infB*	b3168	-99	0.00026529	*eutD*	b2458	-19,64
Tp2	2	*glnQ*	b0809	-98	0.000306575	*glnQ*	b0809	-18,42
Tp2	3	*yfeH*	b2410	-98	0.000306575	*hisM*	b2307	-16,79
Tp2	4	*uxaA*	b3091	-95	0.000473128	*prmC*	b1212	-16
Tp2	5	*acnB*	b0118	-94	0.000546749	*acnB*	b0118	-15,74
Tp2	6	*ybfP*	b0689	-90	0.000974986	*kdgR*	b1827	-15,54
Tp2	7	*yccW*	b0967	-90	0.000974986	*yedI*	b1958	-15,5
Tp2	8	*rpmC*	b3312	-90	0.000974986	*ftsP*	b3017	-15,47
Tp2	9	*fdnI*	b1476	-89	0.00112666	*yccE*	b1001	-15,27
Tp2	10	*emrA*	b2685	-89	0.00112666	*rfaE*	b3052	-15,04

Tp2	11	*glgP*	b3428	-89	0.00112666	*yajL*	b0424	-14,77
Tp2	12	*rnd*	b1804	-88	0.0013019	*sfsB*	b3188	-14,63
Tp2	13	*trs5_6*	b1994	-87	0.00150439	*psaA*	b4645	-14,58
Tp2	14	*fecR*	b4292	-87	0.00150439	*infB*	b3168	-14,48
Tp2	15	*rpmF*	b1089	-86	0.00173834	*yciK*	b1271	-14,43
Tp2	16	*guaA*	b2507	-85	0.00200864	*lrp*	b0889	-14,42
Tp2	17	*appA*	b0980	-83	0.00268168	*ybaM*	b0466	-14,27
Tp2	18	*eutI*	b2458	-83	0.00268168	*afuB*	b0263	-14,13
Tp2	19	*nrfF*	b4075	-82	0.00309844	*rpsU*	b3065	-13,94
Tp2	20	*prpE*	b0335	-81	0.00357984	*metJ*	b3938	-13,8
Tp2	21	*yhdE*	b3248	-81	0.00357984	*hybF*	b2991	-13,63
Tp2	22	*nupC*	b2393	-79	0.00477809	*yeiI*	b2160	-13,56
Tp2	23	*yqjK*	b3100	-79	0.00477809	*yccU*	b0965	-13,54
Tp2	24	*rpoZ*	b3649	-79	0.00477809	*syd*	b2793	-13,53
Tp2	25	*rpsC*	b3314	-78	0.00551974	*ydfG*	b1539	-13,53
Tpke11	1	*dnaJ*	b0015	-120	4.98696e-06	*recB*	b2820	-12,49
Tpke11	2	*ydcX*	b1445	-88	0.00065546	*miaA*	b4171	-12,06
Tpke11	3	*ybdR*	b0608	-85	0.0010354	*yliI*	b0837	-11,36
Tpke11	4	*dnaT*	b4362	-82	0.00163538	*dxs*	b0420	-10,55
Tpke11	5	*yfeS*	b2420	-74	0.00552693	*mltB*	b2701	-10,28
Tpke11	6	*proA*	b0243	-71	0.00871824	*rcsC*	b2218	-10,1
Tpke11	7	*yegD*	b2069	-71	0.00871824	*nuoM*	b2277	-9,83
Tpke11	8	*oraA*	b2698	-71	0.00871824	*yajL*	b0424	-9,8
Tpke11	9	*treR*	b4241	-71	0.00871824	*cpdB*	b4213	-9,68
Tpke11	10	*yjhE*	b4282	-70	0.0101468	*ymfJ*	b1144	-9,68
Tpke11	11	*guaA*	b2507	-69	0.0118081	*rffC*	b3790	-9,57
Tpke11	12	*ybbK*	b0489	-67	0.0159842	*yacC*	b0122	-9,08
Tpke11	13	*aldA*	b1415	-67	0.0159842	*mtfA*	b1976	-8,97
Tpke11	14	*ycdR*	b1023	-66	0.0185922	*yhiN*	b3492	-8,77
Tpke11	15	*cls*	b1249	-66	0.0185922	*yebN*	b1821	-8,74
Tpke11	16	*ycjU*	b1317	-66	0.0185922	*grpE*	b2614	-8,74
Tpke11	17	*yhbY*	b3180	-66	0.0185922	*proA*	b0243	-8,73
Tpke11	18	*ymdC*	b1046	-65	0.021621	*yebW*	b1837	-8,69
Tpke11	19	*xylE*	b4031	-65	0.021621	*betI*	b0313	-8,65
Tpke11	20	*pntA*	b1603	-64	0.0251369	*pldB*	b3825	-8,64
Tpke11	21	*ydhL*	b1648	-64	0.0251369	*ldcA*	b1192	-8,61
Tpke11	22	*pheA*	b2599	-64	0.0251369	*ydgK*	b1626	-8,54
Tpke11	23	*deoD*	b4384	-64	0.0251369	*ydfG*	b1539	-8,53
Tpke11	24	*yneE*	b1520	-63	0.0292159	*pflD*	b3951	-8,49
Tpke11	25	*pphA*	b1838	-63	0.0292159	*fliF*	b1938	-8,47
Tpke70	1	*osmE*	b1739	-105	0.000411193	*arcB*	b3210	-20,81
Tpke70	2	*ybgI*	b0710	-100	0.000796298	*pntB*	b1602	-19,99
Tpke70	3	*yraR*	b3152	-100	0.000796298	*yebA*	b1856	-19,47

Tpke70	4	*tatE*	b0627	-99	0.000908812	*nlpE*	b0192	-18,42
Tpke70	5	*pbpC*	b2519	-99	0.000908812	*rng*	b3247	-18,11
Tpke70	6	*pntB*	b1602	-98	0.00103722	*asmA*	b2064	-17,92
Tpke70	7	*exoX*	b1844	-96	0.00135097	*sdhA*	b0723	-17,63
Tpke70	8	*ygjE*	b3063	-96	0.00135097	*napG*	b2205	-17,56
Tpke70	9	*arcB*	b3210	-96	0.00135097	*asd*	b3433	-17,55
Tpke70	10	*yfiM*	b2586	-95	0.0015418	*tas*	b2834	-17,44
Tpke70	11	*yjiA*	b4352	-94	0.00175956	*ydiH*	b1685	-17,25
Tpke70	12	*torT*	b0994	-92	0.00229157	*yidB*	b3698	-17,18
Tpke70	13	*ppiD*	b0441	-91	0.00261509	*metV*	b2816	-17,08
Tpke70	14	*wcaE*	b2055	-91	0.00261509	*yphD*	b2546	-17,01
Tpke70	15	*araH*	b4460	-91	0.00261509	*gcl*	b0507	-16,84
Tpke70	16	*evgS*	b2370	-89	0.00340533	*ybeD*	b0631	-16,69
Tpke70	17	*yfeK*	b2419	-89	0.00340533	*rplQ*	b3294	-16,59
Tpke70	18	*ycjP*	b1312	-88	0.00388577	*agaD*	b3140	-16,22
Tpke70	19	*yfiL*	b2602	-88	0.00388577	*fabF*	b1095	-16,17
Tpke70	20	*metI*	b0198	-87	0.00443385	*ddlA*	b0381	-16,15
Tpke70	21	*cfa*	b1661	-87	0.00443385	*yjcF*	b4066	-16,11
Tpke70	22	*ydaT*	b1358	-86	0.00505903	*mcrB*	b4346	-16,07
Tpke70	23	*cobS*	b1992	-86	0.00505903	*leuP*	b4369	-16,04
Tpke70	24	*napD*	b2207	-86	0.00505903	*hsdS*	b4348	-15,97
Tpke70	25	*srmB*	b2576	-86	0.00505903	*ycbZ*	b0955	-15,91
SymR	1	*yjiW*	b4347	-320	0	*symE*	b4347	-73,37
SymR	2	*yraQ*	b3151	-108	2.39385e-05	*hycC*	b2723	-13,83
SymR	3	*ynaA*	b1368	-83	0.0011493	*yoaH*	b1811	-9,24
SymR	4	*ydeU*	b1509	-82	0.0013417	*yfbT*	b2293	-8,15
SymR	5	*rpmB*	b3637	-81	0.00156628	*moaC*	b0783	-7,98
SymR	6	*hycC*	b2723	-79	0.00213438	*ygdG*	b2798	-7,93
SymR	7	*emrD*	b3673	-79	0.00213438	*speG*	b1584	-7,86
SymR	8	*yjiL*	b4334	-78	0.00249148	*ydjN*	b1729	-7,81
SymR	9	*yfgC*	b2494	-77	0.00290824	*cysG*	b3368	-7,71
SymR	10	*phnN*	b4094	-76	0.00339459	*yehL*	b2119	-7,63
SymR	11	b1228	b1228	-75	0.00396212	*ybdK*	b0581	-7,61
SymR	12	*yddV*	b1490	-75	0.00396212	*btuC*	b1711	-7,6
SymR	13	*ygjU*	b3089	-74	0.0046243	*yafJ*	b0223	-7,52
SymR	14	*kbl*	b3617	-74	0.0046243	*acrR*	b0464	-7,51
SymR	15	*rnt*	b1652	-73	0.00539686	*phnC*	b4106	-7,5
SymR	16	*ybaQ*	b0483	-72	0.00629807	*cusR*	b0571	-7,48
SymR	17	*ydjO*	b1730	-72	0.00629807	*nfsA*	b0851	-7,47
SymR	18	*ldrD*	b4453	-72	0.00629807	*torZ*	b1872	-7,45
SymR	19	*potG*	b0855	-71	0.00734922	*ybeM*	b4581	-7,43
SymR	20	*yhjV*	b3539	-71	0.00734922	*tonB*	b1252	-7,4
SymR	21	*rpsB*	b0169	-70	0.00857505	*insH*	b0259	-7,4
SymR	22	*nuoB*	b2287	-70	0.00857505	*nadC*	b0109	-7,39

SymR	23	*gltJ*	b0654	-69	0.0100043	*eutJ*	b2454	-7,38
SymR	24	*narZ*	b1468	-69	0.0100043	*ybbJ*	b0488	-7,36
SymR	25	*nupC*	b2393	-69	0.0100043	*yafW*	b0246	-7,35
SroG	1	*yqiC*	b3042	-445	0	*yqiC*	b3042	-35,24
SroG	2	*cdsA*	b0175	-114	2.71785e-05	*citG*	b0613	-21,01
SroG	3	*ykgL*	b0295	-109	5.62881e-05	*nrdI*	b2674	-20,86
SroG	4	*ycjQ*	b1313	-85	0.00185257	*cysJ*	b2764	-20,17
SroG	5	*rnfG*	b1631	-85	0.00185257	*ykgL*	b0295	-18,57
SroG	6	*yiiT*	b3923	-83	0.00247809	*pgl*	b0767	-17,48
SroG	7	*appC*	b0978	-80	0.00383301	*phnH*	b4100	-17,36
SroG	8	*wecB*	b3786	-80	0.00383301	*gfcD*	b0984	-16,52
SroG	9	*yegO*	b2076	-76	0.00685235	*fepB*	b0592	-16,48
SroG	10	*yfjY*	b2644	-76	0.00685235	*yfiP*	b2583	-16,02
SroG	11	*wcaE*	b2055	-74	0.00915829	*rsxG*	b1631	-16
SroG	12	*fecB*	b4290	-74	0.00915829	*rutE*	b1008	-15,92
SroG	13	*ygbO*	b2745	-73	0.0105862	*yihR*	b3879	-15,8
SroG	14	*yhcH*	b3221	-72	0.0122354	*caiA*	b0039	-15,58
SroG	15	*aas*	b2836	-71	0.0141397	*cutA*	b4137	-15,51
SroG	16	*hybB*	b2995	-71	0.0141397	*caiB*	b0038	-15,26
SroG	17	*yjfQ*	b4191	-71	0.0141397	*trg*	b1421	-15,26
SroG	18	*yjiO*	b4337	-71	0.0141397	*phnE*	b4104	-15,16
SroG	19	*metQ*	b0197	-70	0.0163379	*agaD*	b3140	-15,04
SroG	20	*cbpA*	b1000	-70	0.0163379	*tyrB*	b4054	-14,98
SroG	21	*yeiP*	b2171	-70	0.0163379	*livF*	b3454	-14,8
SroG	22	*hscB*	b2527	-70	0.0163379	*yjiK*	b4333	-14,75
SroG	23	*yrfC*	b3394	-70	0.0163379	*ybaZ*	b0454	-14,64
SroG	24	*yjcQ*	b4081	-70	0.0163379	*purN*	b2500	-14,58
SroG	25	*moeA*	b0827	-69	0.0188746	*sugE*	b4148	-14,55
SroA	1	*yhcP*	b3240	-108	3.21357e-05	*yjbI*	b4038	-15,92
SroA	2	*atpA*	b3734	-85	0.00106312	*insH*	b0259	-15,82
SroA	3	*yaaA*	b0006	-84	0.00123772	*yeaR*	b1797	-15,08
SroA	4	*ycdP*	b1021	-82	0.00167759	*ypjK*	b2635	-15,06
SroA	5	*tbpA*	b0068	-80	0.0022736	*pinQ*	b1545	-14,19
SroA	6	*yecM*	b1875	-77	0.00358645	*aaeB*	b3240	-14,18
SroA	7	*yfbT*	b2293	-77	0.00358645	*astD*	b1746	-13,8
SroA	8	*astD*	b1746	-74	0.00565524	*dsrB*	b1952	-13,41
SroA	9	*fhuD*	b0152	-73	0.00658155	*pinR*	b1374	-13,17
SroA	10	*gatD*	b2091	-73	0.00658155	*dicF*	b1574	-13,05
SroA	11	*flgA*	b1072	-72	0.00765899	*fecD*	b4288	-13,02
SroA	12	*lgt*	b2828	-71	0.00891203	*yfbT*	b2293	-12,6
SroA	13	*trs5_5*	b1370	-70	0.010369	*pspA*	b1304	-12,47
SroA	14	*ydfO*	b1549	-70	0.010369	*puuR*	b1299	-12,43
SroA	15	*yjcQ*	b4081	-70	0.010369	*csiE*	b2535	-12,3

SroA	16	*cheW*	b1887	-68	0.0140311	*maeA*	b1479	-11,9
SroA	17	*thiM*	b2104	-68	0.0140311	*codB*	b0336	-11,86
SroA	18	*yacL*	b0119	-67	0.016318	*paaX*	b1399	-11,47
SroA	19	*tolQ*	b0737	-67	0.016318	*recD*	b2819	-11,39
SroA	20	*osmC*	b1482	-67	0.016318	*yjdP*	b4487	-11,35
SroA	21	*yeaR*	b1797	-67	0.016318	*ycbV*	b0943	-11,25
SroA	22	*crp*	b3357	-67	0.016318	*glk*	b2388	-11,07
SroA	23	*ilvB*	b3671	-67	0.016318	*cyaA*	b3806	-10,97
SroA	24	*kch*	b1250	-66	0.0189741	*rybB*	b4417	-10,96
SroA	25	*ymfH*	b1142	-65	0.0220576	*emrD*	b3673	-10,88
SraA	1	*lon*	b0439	-150	3.9043e-09	*lon*	b0439	-53,04
SraA	2	*yedR*	b1963	-75	0.00130994	*yciA*	b1253	-18,21
SraA	3	*kefA*	b0465	-68	0.00428908	*artJ*	b0860	-16,81
SraA	4	*hofC*	b0106	-65	0.007125	*yaaU*	b0045	-15,4
SraA	5	*ruvC*	b1863	-64	0.0084368	*ruvC*	b1863	-15,12
SraA	6	*yecH*	b1906	-64	0.0084368	*insA*	b0022	-15,04
SraA	7	*nikC*	b3478	-63	0.00998891	*yhaO*	b3110	-14,54
SraA	8	*ecnB*	b4411	-63	0.00998891	*asnT*	b1977	-14,36
SraA	9	*fsr*	b0479	-62	0.0118249	*yneN*	b1500	-14,35
SraA	10	*smf*	b4473	-62	0.0118249	*ybjJ*	b0845	-14,33
SraA	11	*setA*	b0070	-61	0.0139958	*ycjP*	b1312	-14,22
SraA	12	*allP*	b0511	-60	0.0165621	*yfeN*	b2408	-14,2
SraA	13	*chaA*	b1216	-59	0.0195941	*ybiO*	b0808	-14,11
SraA	14	b1500	b1500	-59	0.0195941	*dfp*	b3639	-14,07
SraA	15	*yjiH*	b4330	-59	0.0195941	*fadI*	b2342	-13,86
SraA	16	*yaaU*	b0045	-58	0.0231747	*rffT*	b4481	-13,78
SraA	17	*kdpD*	b0695	-58	0.0231747	*dmsD*	b1591	-13,76
SraA	18	*yjiN*	b4336	-57	0.0274004	*ynjC*	b1755	-13,71
SraA	19	*hslJ*	b1379	-55	0.0382554	*yiaY*	b3589	-13,62
SraA	20	*argT*	b2310	-55	0.0382554	*yhaM*	b4470	-13,59
SraA	21	*ygiB*	b3037	-55	0.0382554	*ydbA*	b4492	-13,53
SraA	22	*ycjP*	b1312	-54	0.0451667	*fabB*	b2323	-13,49
SraA	23	*cvpA*	b2313	-54	0.0451667	*yraI*	b3143	-13,41
SraA	24	*wzzE*	b3785	-54	0.0451667	*motA*	b1890	-13,39
SraA	25	*ybcH*	b0567	-53	0.0532915	*ygbM*	b2739	-13,36
RyjB	1	*sgcA*	b4302	-450	0	*sgcA*	b4302	-124,61
RyjB	2	*hflK*	b4174	-105	4.82854e-05	*fryA*	b2383	-19,91
RyjB	3	*ydhX*	b1671	-91	0.000408976	*macA*	b0878	-17,69
RyjB	4	*yiiR*	b3921	-87	0.000752931	*dcyD*	b1919	-17,54
RyjB	5	*yrbB*	b3191	-84	0.0011899	*rutC*	b1010	-16,71
RyjB	6	*yafZ*	b0252	-83	0.00138595	*poxB*	b0871	-16,46
RyjB	7	*lacY*	b0343	-83	0.00138595	*fdnI*	b1476	-16
RyjB	8	*hybG*	b2990	-83	0.00138595	*yedQ*	b1956	-15,09

RyjB	9	*yheM*	b3344	-83	0.00138595	*glpC*	b2243	-14,99
RyjB	10	*tdcR*	b3119	-82	0.00161429	*tyrV*	b1230	-14,63
RyjB	11	*cdsA*	b0175	-80	0.00218989	*chaB*	b1217	-14,35
RyjB	12	*yehM*	b2120	-80	0.00218989	*fadA*	b3845	-14,29
RyjB	13	*ypdD*	b2383	-80	0.00218989	*yhaV*	b3130	-14,27
RyjB	14	*ordL*	b1301	-79	0.00255051	*nuoL*	b2278	-14,22
RyjB	15	*ycgI*	b1173	-78	0.00297043	*pqiB*	b0951	-14,04
RyjB	16	*ugpC*	b3450	-78	0.00297043	*chbR*	b1735	-13,92
RyjB	17	*fucO*	b2799	-76	0.0040286	*yfiQ*	b2584	-13,88
RyjB	18	*cysI*	b2763	-75	0.0046913	*ybiC*	b0801	-13,86
RyjB	19	*chaB*	b1217	-74	0.0054627	*guaA*	b2507	-13,8
RyjB	20	*fliE*	b1937	-73	0.00636055	*motA*	b1890	-13,65
RyjB	21	*aceF*	b0115	-72	0.00740541	*melA*	b4119	-13,61
RyjB	22	*rpsA*	b0911	-72	0.00740541	*pntB*	b1602	-13,5
RyjB	23	*ycjV*	b1318	-72	0.00740541	*yfaE*	b2236	-13,38
RyjB	24	*caiA*	b0039	-71	0.00862117	*leuO*	b0076	-13,36
RyjB	25	*btuE*	b1710	-71	0.00862117	*ybaS*	b0485	-13,25
RyfD	1	*clpB*	b2592	-131	2.12639e-06	*gntP*	b4321	-14,54
RyfD	2	*idi*	b2889	-103	0.000127367	*yeiP*	b2171	-14,16
RyfD	3	*gltS*	b3653	-101	0.000170611	*gudD*	b2787	-12,36
RyfD	4	*ygiT*	b3021	-100	0.000197461	*ydiI*	b1686	-11,87
RyfD	5	*tesB*	b0452	-96	0.000354297	*uvrY*	b1914	-11,84
RyfD	6	*napF*	b2208	-93	0.000549245	*nudL*	b1813	-11,72
RyfD	7	*yjfR*	b4192	-92	0.000635663	*yjbR*	b4057	-11,54
RyfD	8	*ydfO*	b1549	-89	0.000985352	*atpD*	b3732	-11,47
RyfD	9	*yrfD*	b3395	-88	0.00114035	*ybhQ*	b0791	-11,46
RyfD	10	*yfhM*	b2520	-87	0.00131971	*trpA*	b1260	-11,43
RyfD	11	*narX*	b1222	-85	0.00176743	*gspH*	b3329	-11,34
RyfD	12	*yjgL*	b4253	-85	0.00176743	*recB*	b2820	-11,27
RyfD	13	*sbcD*	b0398	-83	0.00236685	*yhfL*	b3369	-11,25
RyfD	14	*yfbS*	b2292	-82	0.00273886	*yjdI*	b4126	-11,14
RyfD	15	*yqjF*	b3101	-82	0.00273886	*lpp*	b1677	-11,08
RyfD	16	*yfdU*	b2373	-81	0.00316925	*nuoL*	b2278	-11,02
RyfD	17	*yijD*	b3964	-81	0.00316925	*yggP*	b4465	-10,91
RyfD	18	*gntP*	b4321	-81	0.00316925	*yjjP*	b4364	-10,89
RyfD	19	*caiD*	b0036	-80	0.00366715	*ygbF*	b2754	-10,75
RyfD	20	*yabN*	b0069	-80	0.00366715	*hisA*	b2024	-10,67
RyfD	21	*csgC*	b1043	-80	0.00366715	*eutE*	b2455	-10,64
RyfD	22	*yejA*	b2177	-80	0.00366715	*syd*	b2793	-10,47
RyfD	23	*ygcG*	b2778	-80	0.00366715	*ycgH*	b4491	-10,44
RyfD	24	b0359	b0359	-79	0.0042431	*yfaD*	b2244	-10,27
RyfD	25	*ydiI*	b1686	-79	0.0042431	*ybcV*	b0558	-10,25
RyfB	1	*dcuC*	b0621	-112	0.000107551	*yagX*	b0291	-20,63

RyfB	2	*ymfQ*	b1153	-105	0.000278497	*pagP*	b0622	-19,61
RyfB	3	*araF*	b1901	-104	0.000319041	*ycjG*	b1325	-19,02
RyfB	4	*nirC*	b3367	-102	0.000418692	*hisL*	b2018	-18,12
RyfB	5	*kdgT*	b3909	-102	0.000418692	*yfbR*	b2291	-17,64
RyfB	6	*crcA*	b0622	-99	0.000629438	*grxA*	b0849	-17,59
RyfB	7	*fucU*	b2804	-99	0.000629438	*gspC*	b3324	-17,41
RyfB	8	*ppdB*	b2825	-98	0.000721055	*kdpD*	b0695	-17,27
RyfB	9	*ycdU*	b1029	-97	0.000826	*hybC*	b2994	-16,96
RyfB	10	*guaD*	b2883	-96	0.000946213	*atoD*	b2221	-16,93
RyfB	11	*ydhB*	b1659	-95	0.00108391	*ypeC*	b2390	-16,92
RyfB	12	*ycbB*	b0925	-94	0.00124164	*valV*	b1665	-16,54
RyfB	13	*mutH*	b2831	-91	0.00186622	*nac*	b1988	-16,51
RyfB	14	*hybC*	b2994	-90	0.00213766	*rsxD*	b1630	-16,48
RyfB	15	*msyB*	b1051	-88	0.00280455	*yidR*	b3689	-16,44
RyfB	16	*yfbS*	b2292	-88	0.00280455	*ydhP*	b1657	-16,22
RyfB	17	*hydN*	b2713	-88	0.00280455	*ydeE*	b1534	-16,18
RyfB	18	*yzgL*	b3427	-88	0.00280455	*aat*	b0885	-16,16
RyfB	19	*yccJ*	b1003	-87	0.00321225	*yqfB*	b2900	-16,16
RyfB	20	*yeeJ*	b1978	-87	0.00321225	*mdtE*	b3513	-16,13
RyfB	21	*phnA*	b4108	-87	0.00321225	*ptrB*	b1845	-15,87
RyfB	22	*ykgK*	b0294	-86	0.0036791	*yeaJ*	b1786	-15,81
RyfB	23	*yhfQ*	b3374	-86	0.0036791	*ubiC*	b4039	-15,81
RyfB	24	*rbsB*	b3751	-86	0.0036791	*ygfF*	b2902	-15,72
RyfB	25	*yahL*	b0326	-84	0.00482571	*ybhB*	b0773	-15,71
RydC	1	*ydiT*	b1700	-100	6.29372e-05	*yhdJ*	b3262	-16,53
RydC	2	*dsbA*	b3860	-80	0.0014707	*rpmC*	b3312	-16,2
RydC	3	*yahE*	b0319	-76	0.00276075	*pgpA*	b0418	-16,1
RydC	4	*yeeW*	b2006	-75	0.00323125	*ydiR*	b1698	-15,85
RydC	5	*yiiE*	b3889	-75	0.00323125	*ytfN*	b4221	-15,82
RydC	6	*narQ*	b2469	-72	0.00517946	*gabT*	b2662	-15,35
RydC	7	*malF*	b4033	-71	0.00606092	*moaB*	b0782	-15,05
RydC	8	*yjeT*	b4176	-71	0.00606092	*hycD*	b2722	-15,05
RydC	9	*yjiM*	b4335	-71	0.00606092	*flhA*	b1879	-14,98
RydC	10	*eutA*	b2451	-70	0.00709185	*ulaA*	b4193	-14,97
RydC	11	*pheP*	b0576	-69	0.0082974	*rpmH*	b3703	-14,92
RydC	12	*yejO*	b2190	-69	0.0082974	*yghW*	b2998	-14,92
RydC	13	*cmr*	b0842	-68	0.00970689	*grpE*	b2614	-14,81
RydC	14	*ydcU*	b1442	-68	0.00970689	*yahE*	b0319	-14,69
RydC	15	*nuoG*	b2283	-68	0.00970689	*rpoC*	b3988	-14,63
RydC	16	*yaaA*	b0006	-67	0.0113544	*yihR*	b3879	-14,35
RydC	17	*dacB*	b3182	-67	0.0113544	*yfdM*	b2356	-14,3
RydC	18	*artI*	b0863	-66	0.0132797	*grxC*	b3610	-14,13
RydC	19	*ycjC*	b1299	-66	0.0132797	*gsiB*	b0830	-14,1
RydC	20	*ytfN*	b4221	-65	0.0155289	*hisM*	b2307	-13,98

RydC	21	*recG*	b3652	-64	0.0181555	*yecE*	b1868	-13,9
RydC	22	*rpoC*	b3988	-64	0.0181555	*rplO*	b3301	-13,83
RydC	23	*panC*	b0133	-63	0.0212216	*ispA*	b0421	-13,82
RydC	24	*pepP*	b2908	-63	0.0212216	*yciW*	b1287	-13,79
RydC	25	*mreB*	b3251	-63	0.0212216	*mhpD*	b0350	-13,59
OhsC	1	*yjeI*	b4144	-89	0.000453947	*rspB*	b1580	-15,89
OhsC	2	*ybfM*	b0681	-83	0.0011493	*hsdM*	b4349	-15,84
OhsC	3	*cusB*	b0574	-80	0.00182842	*fliN*	b1946	-15,63
OhsC	4	*yafA*	b0239	-77	0.00290824	*mioC*	b3742	-15,15
OhsC	5	*yjiT*	b4342	-77	0.00290824	*phnG*	b4101	-14,65
OhsC	6	*argC*	b3958	-74	0.0046243	*hchA*	b1967	-14,23
OhsC	7	*yghJ*	b4466	-74	0.0046243	*yajO*	b0419	-14,18
OhsC	8	*glpF*	b3927	-73	0.00539686	*ydfO*	b1549	-13,91
OhsC	9	*yahE*	b0319	-72	0.00629807	*ygiW*	b3024	-13,55
OhsC	10	*fliN*	b1946	-72	0.00629807	*ycjW*	b1320	-13,32
OhsC	11	*frdD*	b4151	-72	0.00629807	*sdaC*	b2796	-12,78
OhsC	12	*ybaP*	b0482	-71	0.00734922	*sbcC*	b0397	-12,73
OhsC	13	*yiiP*	b3915	-71	0.00734922	*yedZ*	b1972	-12,18
OhsC	14	*arcA*	b4401	-71	0.00734922	*yraM*	b3147	-11,89
OhsC	15	*ybaO*	b0447	-70	0.00857505	*nhoA*	b1463	-11,89
OhsC	16	*hlyE*	b1182	-69	0.0100043	*gfcA*	b0987	-11,73
OhsC	17	*ydiI*	b1686	-69	0.0100043	*ydcV*	b1443	-11,52
OhsC	18	*yqgD*	b2941	-69	0.0100043	*hycC*	b2723	-11,48
OhsC	19	*yrbG*	b3196	-69	0.0100043	*aslB*	b3800	-11,32
OhsC	20	*serB*	b4388	-69	0.0100043	*cheR*	b1884	-11,31
OhsC	21	*appY*	b0564	-68	0.0116704	*xseB*	b0422	-11,29
OhsC	22	*pspC*	b1306	-68	0.0116704	*fumC*	b1611	-11,18
OhsC	23	*ligA*	b2411	-68	0.0116704	*yehS*	b2124	-11,16
OhsC	24	*gspG*	b3328	-68	0.0116704	*yhjV*	b3539	-10,97
OhsC	25	*csgA*	b1042	-67	0.013612	*yifL*	b4558	-10,97
IsrB	1	*ndk*	b2518	-87	0.00115741	*mukE*	b0923	-15,7
IsrB	2	*yrdA*	b3279	-86	0.00134147	*yihF*	b3861	-15,49
IsrB	3	*aphA*	b4055	-85	0.00155479	*cdaR*	b0162	-15,14
IsrB	4	*sfmA*	b0530	-84	0.00180199	*hisQ*	b2308	-14,91
IsrB	5	b1364	b1364	-84	0.00180199	*ydhI*	b1643	-14,3
IsrB	6	*nadD*	b0639	-80	0.00325073	*tmk*	b1098	-14,2
IsrB	7	*tehB*	b1430	-78	0.00436525	*yhgA*	b3411	-14,08
IsrB	8	*gnsB*	b1550	-78	0.00436525	*yahE*	b0319	-14,06
IsrB	9	*ygaC*	b2671	-78	0.00436525	*efeO*	b1018	-13,92
IsrB	10	*alsE*	b4085	-78	0.00436525	*kefB*	b3350	-13,9
IsrB	11	*ycdO*	b1018	-77	0.00505817	*ynbD*	b1411	-13,84
IsrB	12	*mukE*	b0923	-76	0.00586076	*puuD*	b1298	-13,5
IsrB	13	*ydaV*	b1360	-75	0.00679026	*yghB*	b3009	-13,44

IsrB	14	*yqfA*	b2899	-75	0.00679026	*yfaA*	b2230	-13,3
IsrB	15	*yggL*	b2959	-75	0.00679026	*hybA*	b2996	-13,3
IsrB	16	*yhiP*	b3496	-75	0.00679026	*sufA*	b1684	-13,3
IsrB	17	*ycdW*	b1033	-74	0.0078666	*yafK*	b0224	-13,22
IsrB	18	*tonB*	b1252	-74	0.0078666	*ndk*	b2518	-13,2
IsrB	19	*yobD*	b1820	-74	0.0078666	*psiE*	b4030	-13,18
IsrB	20	*metH*	b4019	-74	0.0078666	*torC*	b0996	-12,96
IsrB	21	*yjfM*	b4185	-74	0.0078666	*ybjE*	b0874	-12,91
IsrB	22	*yjhC*	b4280	-74	0.0078666	*nikR*	b3481	-12,86
IsrB	23	*yhhA*	b3448	-73	0.00911276	*metH*	b4019	-12,83
IsrB	24	*ygfM*	b2880	-72	0.0105553	*omrB*	b4445	-12,79
IsrB	25	*wzxE*	b3792	-72	0.0105553	*yeaX*	b1803	-12,76
C0664	1	*phnL*	b4096	-394	0	*phnL*	b4096	-48,28
C0664	2	*nrdG*	b4237	-294	0	*nrdG*	b4237	-42,43
C0664	3	*nrdB*	b2235	-279	3.33067e-16	*yahI*	b0323	-39,34
C0664	4	*malF*	b4033	-273	8.88178e-16	*nrdB*	b2235	-38,85
C0664	5	*holC*	b4259	-273	8.88178e-16	*holC*	b4259	-35,77
C0664	6	*wcaK*	b2045	-267	2.10942e-15	*yjcH*	b4068	-34,45
C0664	7	b0725	b0725	-240	1.21347e-13	*malF*	b4033	-33,17
C0664	8	*yahI*	b0323	-237	1.89959e-13	*wcaK*	b2045	-31,13
C0664	9	*prpC*	b0333	-221	2.06934e-12	*pstB*	b3725	-29,11
C0664	10	*fkpB*	b0028	-203	3.03948e-11	*prpC*	b0333	-27,79
C0664	11	*sseB*	b2522	-199	5.52236e-11	*sseB*	b2522	-26,78
C0664	12	*yjcH*	b4068	-196	8.64208e-11	*yqjI*	b3071	-26,75
C0664	13	*tldD*	b3244	-189	2.45718e-10	*phnE*	b4104	-26,5
C0664	14	*mhpT*	b0353	-184	5.18314e-10	*rhaD*	b3902	-23,11
C0664	15	*ygdL*	b2812	-184	5.18314e-10	*sanA*	b2144	-21,59
C0664	16	b3004	b3004	-184	5.18314e-10	*entD*	b0583	-19,24
C0664	17	*yqjI*	b3071	-180	9.4171e-10	*lolA*	b0891	-16,35
C0664	18	*rhaD*	b3902	-180	9.4171e-10	*lacA*	b0342	-16,26
C0664	19	*yjfH*	b4180	-178	1.26935e-09	*yjbT*	b4620	-16,22
C0664	20	*phnE*	b4104	-176	1.71097e-09	*mhpT*	b0353	-15,15
C0664	21	*entD*	b0583	-174	2.30624e-09	*ygbT*	b2755	-14,62
C0664	22	*eutH*	b2452	-173	2.67754e-09	*rlmB*	b4180	-14,09
C0664	23	*pstB*	b3725	-170	4.19015e-09	*yjdK*	b4128	-12,17
C0664	24	*araD*	b0061	-169	4.86475e-09	*gsiA*	b0829	-12,15
C0664	25	*aceK*	b4016	-168	5.64796e-09	*fsaB*	b3946	-11,85
C0614	1	*ascG*	b2714	-99	0.000118742	*ryfA*	b4440	-62,43
C0614	2	*yjjV*	b4378	-81	0.00185597	*sdaB*	b2797	-17,29
C0614	3	*farR*	b0730	-80	0.00216201	*ulaD*	b4196	-14,57
C0614	4	*tdcR*	b3119	-80	0.00216201	*qmcA*	b0489	-14,21
C0614	5	*yraQ*	b3151	-79	0.00251845	*ybaS*	b0485	-13,97
C0614	6	*nadB*	b2574	-77	0.00341698	*melA*	b4119	-13,78

C0614	7	*proV*	b2677	-77	0.00341698	*yfbO*	b2274	-13,62
C0614	8	*ecpD*	b0140	-76	0.0039799	*insE*	b0298	-13,62
C0614	9	*cspF*	b1558	-76	0.0039799	*yjcZ*	b4110	-13,61
C0614	10	*amiA*	b2435	-76	0.0039799	*rhaD*	b3902	-13,5
C0614	11	*ygbL*	b2738	-76	0.0039799	*carB*	b0033	-13,24
C0614	12	*rstB*	b1609	-75	0.00463534	*scpB*	b2919	-13,19
C0614	13	*glpE*	b3425	-75	0.00463534	*prfB*	b2891	-13,13
C0614	14	*aroL*	b0388	-74	0.00539843	*glpR*	b3423	-13,06
C0614	15	*glcG*	b2977	-74	0.00539843	*grxD*	b1654	-13,05
C0614	16	*uxaC*	b3092	-74	0.00539843	*rsmC*	b4371	-13,03
C0614	17	*yjgN*	b4257	-74	0.00539843	*yjjV*	b4378	-12,98
C0614	18	*ykfG*	b0247	-73	0.00628676	*ycbG*	b0956	-12,93
C0614	19	*ddlA*	b0381	-73	0.00628676	*fldA*	b0684	-12,81
C0614	20	*ydcC*	b1460	-73	0.00628676	*gatC*	b2092	-12,77
C0614	21	*mcrB*	b4346	-73	0.00628676	*pgm*	b0688	-12,61
C0614	22	*yfhM*	b2520	-72	0.00732071	*insE*	b0298	-12,51
C0614	23	*ygiL*	b3043	-72	0.00732071	*ydhJ*	b1644	-12,44
C0614	24	*yhaC*	b3121	-72	0.00732071	*emrY*	b2367	-12,28
C0614	25	*fdnG*	b1474	-71	0.00852399	*ravA*	b3746	-12,24
C0362	1	*ybfP*	b0689	-390	0	*yiiX*	b3937	-56,57
C0362	2	*ybiO*	b0808	-348	0	*ybfP*	b0689	-52,26
C0362	3	*yiiX*	b3937	-386	0	*yceK*	b1050	-45,19
C0362	4	*yceK*	b1050	-323	1.11022e-16	*yggN*	b2958	-34,68
C0362	5	*flgG*	b1078	-266	1.59983e-13	*flgG*	b1078	-21,94
C0362	6	*yggN*	b2958	-240	5.16731e-12	*yidB*	b3698	-21,33
C0362	7	*malM*	b4037	-210	2.84806e-10	*wcaA*	b2059	-19,52
C0362	8	*fkpB*	b0028	-206	4.86094e-10	*ybdD*	b4512	-19,44
C0362	9	*tldD*	b3244	-189	4.71462e-09	*mtlD*	b3600	-17,61
C0362	10	*phnB*	b4107	-185	8.04671e-09	*deoA*	b4382	-17,14
C0362	11	*mtlD*	b3600	-177	2.34402e-08	*yjdN*	b4107	-16,83
C0362	12	*rbfA*	b3167	-176	2.67919e-08	*gutM*	b2706	-16,22
C0362	13	*deoA*	b4382	-176	2.67919e-08	*ytfR*	b4485	-15,57
C0362	14	*araD*	b0061	-174	3.50017e-08	*aceK*	b4016	-15,42
C0362	15	*yafJ*	b0223	-172	4.57272e-08	*ibpA*	b3687	-14,3
C0362	16	*pppA*	b2972	-172	4.57272e-08	*livH*	b3457	-14,23
C0362	17	*yidB*	b3698	-171	5.22657e-08	*amiC*	b2817	-14,07
C0362	18	*eutH*	b2452	-169	6.82814e-08	*rnt*	b1652	-13,78
C0362	19	*wcaA*	b2059	-164	1.33204e-07	*cyoD*	b0429	-13,49
C0362	20	*aceK*	b4016	-163	1.52251e-07	*panF*	b3258	-13,27
C0362	21	*caiB*	b0038	-162	1.74021e-07	*rzoD*	b4510	-13,2
C0362	22	*yrbA*	b3190	-158	2.97011e-07	*yraM*	b3147	-13,18
C0362	23	*uxuR*	b4324	-157	3.39481e-07	*yffP*	b2447	-13,16
C0362	24	*ytfR*	b4485	-150	8.65197e-07	*argT*	b2310	-13,05
C0362	25	*yjfH*	b4180	-149	9.88913e-07	*rzoR*	b4528	-12,9

C0343	1	*fepB*	b0592	-80	0.00180073	*hybA*	b2996	-33,69
C0343	2	b1371	b1371	-76	0.00334578	*hsdS*	b4348	-25,96
C0343	3	*yaaU*	b0045	-74	0.00455959	*yeeZ*	b2016	-23,75
C0343	4	*dgoA*	b4477	-74	0.00455959	*hisF*	b2025	-23,67
C0343	5	*fliD*	b1924	-71	0.00725065	*yhdP*	b4472	-22,97
C0343	6	*yihT*	b3881	-70	0.00846171	*yjhC*	b4280	-21,5
C0343	7	*yhbZ*	b3183	-69	0.00987405	*arcB*	b3210	-21,41
C0343	8	*ilvB*	b3671	-69	0.00987405	*yciY*	b4595	-21,02
C0343	9	*yfcY*	b2342	-68	0.0115207	*ilvG*	b4488	-20,96
C0343	10	*yhhL*	b3466	-67	0.0134402	*atpF*	b3736	-20,09
C0343	11	*rnc*	b2567	-66	0.0156769	*rutR*	b1013	-19,5
C0343	12	*wecC*	b3787	-65	0.0182824	*ydaN*	b1342	-19,4
C0343	13	*ilvH*	b0078	-64	0.0213161	*ilvB*	b3671	-19,33
C0343	14	*apbA*	b0425	-64	0.0213161	*ulaF*	b4198	-19,26
C0343	15	*yfeW*	b2430	-63	0.0248469	*ycjV*	b4524	-19,16
C0343	16	*yiaK*	b3575	-63	0.0248469	*cbl*	b1987	-19,12
C0343	17	*dgoT*	b3691	-63	0.0248469	*nrfF*	b4075	-18,91
C0343	18	*ispH*	b0029	-62	0.0289539	*sdhA*	b0723	-18,91
C0343	19	*yabK*	b0067	-62	0.0289539	*glcB*	b2976	-18,77
C0343	20	*ylaC*	b0458	-62	0.0289539	*ycbW*	b0946	-18,73
C0343	21	*yfcA*	b2327	-62	0.0289539	*ldrC*	b4423	-18,58
C0343	22	*uxaA*	b3091	-62	0.0289539	*ubiH*	b2907	-18,24
C0343	23	*pflB*	b0903	-61	0.0337278	*srlD*	b2705	-18,2
C0343	24	*exoX*	b1844	-61	0.0337278	*yhhL*	b3466	-18,13
C0343	25	*yfjD*	b4461	-61	0.0337278	*rfbD*	b2040	-18,02
C0299	1	*torT*	b0994	-85	0.000884122	*ycgH*	b4491	-18,32
C0299	2	*mgsA*	b0963	-80	0.0019116	*xseA*	b2509	-17,99
C0299	3	*yggN*	b2958	-78	0.00260189	*suhB*	b2533	-17,79
C0299	4	*yhhS*	b3473	-78	0.00260189	*appA*	b0980	-17,73
C0299	5	*ybiJ*	b0802	-76	0.00354101	*insA*	b0022	-17,63
C0299	6	*yliF*	b0834	-75	0.00413067	*modA*	b0763	-17,24
C0299	7	*ogt*	b1335	-75	0.00413067	*ybiJ*	b0802	-16,67
C0299	8	*ynaF*	b1376	-75	0.00413067	*ychH*	b1205	-16,19
C0299	9	*gnsB*	b1550	-75	0.00413067	*trpD*	b1263	-16,06
C0299	10	*htrL*	b3618	-74	0.00481828	*rnpA*	b3704	-15,72
C0299	11	*ccmC*	b2199	-73	0.00562003	*mdtK*	b1663	-15,4
C0299	12	*yahA*	b0315	-72	0.00655475	*ypfG*	b2466	-15,33
C0299	13	*menD*	b2264	-72	0.00655475	*ymgI*	b4593	-14,78
C0299	14	*nuoC*	b2286	-72	0.00655475	*kefC*	b0047	-14,76
C0299	15	*xseA*	b2509	-72	0.00655475	*ybhP*	b0790	-14,73
C0299	16	*mtgA*	b3208	-72	0.00655475	*flgI*	b1080	-14,72
C0299	17	*yhhX*	b3440	-71	0.00764434	*ddpB*	b1486	-14,59
C0299	18	*thiI*	b0423	-70	0.00891423	*aas*	b2836	-14,56

C0299	19	*ybjS*	b0868	-70	0.00891423	*ppiA*	b3363	-14,46
C0299	20	*yecG*	b1895	-70	0.00891423	*ybdG*	b0577	-14,42
C0299	21	*yfcC*	b2298	-70	0.00891423	*galR*	b2837	-14,41
C0299	22	*yfeA*	b2395	-69	0.010394	*yecM*	b1875	-14,31
C0299	23	*ppiA*	b3363	-69	0.010394	*prmB*	b2330	-14,13
C0299	24	*cysD*	b2752	-68	0.0121178	*hupB*	b0440	-13,99
C0299	25	*slp*	b3506	-68	0.0121178	*yfeY*	b2432	-13,89
C0293	1	*yecE*	b1868	-77	0.00278115	*yhdY*	b3270	-17,71
C0293	2	*thiI*	b0423	-76	0.00324817	*yhhS*	b3473	-17,08
C0293	3	*phnD*	b4105	-76	0.00324817	*ptrB*	b1845	-16,39
C0293	4	*yceJ*	b1057	-75	0.00379346	*fecI*	b4293	-16,15
C0293	5	*nrfG*	b4076	-73	0.00517328	*yqfE*	b2915	-16,02
C0293	6	*ymfI*	b1143	-71	0.00705322	*yjbB*	b4020	-15,98
C0293	7	*yjbB*	b4020	-70	0.00823464	*ccmF*	b2196	-15,78
C0293	8	*mhpR*	b0346	-69	0.009613	*eda*	b1850	-15,59
C0293	9	*ccmG*	b2195	-69	0.009613	*frdB*	b4153	-15,4
C0293	10	*ygfU*	b2888	-69	0.009613	*ilvE*	b3770	-15,39
C0293	11	*ybaV*	b0442	-68	0.0112208	*nrdE*	b2675	-15,37
C0293	12	*ubiG*	b2232	-67	0.0130956	*ydfA*	b1571	-15,23
C0293	13	*yhfY*	b3382	-67	0.0130956	*mfd*	b1114	-15,11
C0293	14	*yehT*	b2125	-66	0.0152814	*yceJ*	b1057	-14,89
C0293	15	*yibQ*	b3614	-66	0.0152814	*ydcV*	b1443	-14,71
C0293	16	b1027	b1027	-65	0.0178286	*rpmH*	b3703	-14,7
C0293	17	*yebA*	b1856	-65	0.0178286	*stfE*	b1157	-14,62
C0293	18	*pth*	b1204	-64	0.0207959	*nrfG*	b4076	-14,48
C0293	19	*ydiT*	b1700	-64	0.0207959	*ydiT*	b1700	-14,46
C0293	20	*glcF*	b4467	-64	0.0207959	*hcaE*	b2538	-14,25
C0293	21	*folA*	b0048	-63	0.0242509	*exuR*	b3094	-14,17
C0293	22	*pin*	b1158	-63	0.0242509	*uidC*	b1615	-14,03
C0293	23	*ynfA*	b1582	-63	0.0242509	*rpsJ*	b3321	-13,95
C0293	24	*thrS*	b1719	-63	0.0242509	*ansA*	b1767	-13,92
C0293	25	*yegS*	b2086	-63	0.0242509	*kefB*	b3350	-13,92
C0067	1	*ynfE*	b1587	-110	3.16446e-05	*astB*	b1745	-13,77
C0067	2	*ybeU*	b0648	-104	7.76104e-05	*dsbC*	b2893	-12,96
C0067	3	*yafQ*	b0225	-100	0.000141143	*yciB*	b1254	-12,84
C0067	4	*emrD*	b3673	-99	0.000163905	*ycgH*	b4491	-12,01
C0067	5	*ygiL*	b3043	-97	0.000221033	*yiaM*	b3577	-11,96
C0067	6	*yjjP*	b4364	-96	0.000256678	*def*	b3287	-11,72
C0067	7	*norV*	b2710	-94	0.000346135	*wbbK*	b2032	-11,49
C0067	8	*yeaU*	b1800	-92	0.000466763	*yfbO*	b2274	-11,4
C0067	9	*ydeH*	b1535	-91	0.000542024	*yehA*	b2108	-11,37
C0067	10	*ygaZ*	b2682	-91	0.000542024	*ascF*	b2715	-11,36
C0067	11	*yfbO*	b2274	-90	0.000629416	*wcaK*	b2045	-11,23

C0067	12	*yraH*	b3142	-89	0.000730893	*ypfM*	b4606	-11,2
C0067	13	*ompT*	b0565	-88	0.000848725	*ygfI*	b2921	-11,15
C0067	14	*ftn*	b1905	-87	0.000985543	*ygbJ*	b2736	-11,11
C0067	15	*yhgN*	b3434	-87	0.000985543	*udp*	b3831	-11,03
C0067	16	*glcG*	b2977	-86	0.0011444	*thiK*	b1106	-10,98
C0067	17	*ydiE*	b1705	-85	0.00132886	*rpiB*	b4090	-10,95
C0067	18	*yhjB*	b3520	-85	0.00132886	*isrC*	b4435	-10,93
C0067	19	*yhjA*	b3518	-84	0.00154301	*sufB*	b1683	-10,91
C0067	20	*yafL*	b0227	-83	0.00179165	*coaE*	b0103	-10,78
C0067	21	*ykgD*	b0305	-83	0.00179165	*yeeE*	b2013	-10,62
C0067	22	*yfbL*	b2271	-83	0.00179165	*yebN*	b1821	-10,61
C0067	23	*yhfZ*	b3383	-83	0.00179165	*rplB*	b3317	-10,4
C0067	24	*yiaT*	b3584	-83	0.00179165	*ykgM*	b0296	-10,38
C0067	25	*yciV*	b1266	-82	0.00208032	*ydhX*	b1671	-10,31

[a]sRNA id: at this column was indicated id of sRNA.

[b] predictions rank : predictions rank from 1 to 25, by either targetRNA or intaRNA

Predictions made by targetRNA:

[c]target name (targetRNA): gene name of the target, predicted by targetRNA.

[d]target b-number (targetRNA): b-number of the target, predicted by targetRNA.

[e]score (targetRNA): score of the predicted sRNA-target interaction, assigned by targetRNA.

[f]p-value: p-value for the indicated sRNA-target interaction, assigned by targetRNA.

Predictions made by intaRNA:

[g]target name (intaRNA): gene name of the target, predicted by intaRNA.

[h]target b-number (intaRNA): b-number of the target, predicted by intaRNA.

[i]score (intaRNA): score of the predicted sRNA-target interaction, assigned by intaRNA.
