# Supplementary material for: Inferring the relation between transcriptional and posttranscriptional regulation from expression compendia
Source: BMC Microbiol. 2014 Jan 27;14:14. doi: 10.1186/1471-2180-14-14 (PMC3948049; doi:10.1186/1471-2180-14-14)
Supplement: Additional file 9: Table S7 — List of regulators. [file 1471-2180-14-14-S9.pdf]

Additional file 9 - Table 7: List of regulators

[a]Regulator ID	[b]Regulator type	[c]Source
AcrR	TF	RegulonDB
Ada	TF	RegulonDB
AdiY	TF	RegulonDB
AgaR	TF	RegulonDB
AlaS	TF	RegulonDB
AllR	TF	RegulonDB
AllS	TF	RegulonDB
AlpA	TF	RegulonDB
AlsR	TF	RegulonDB
AppY	TF	RegulonDB
AraC	TF	RegulonDB
ArcA	TF	RegulonDB
ArgP	TF	RegulonDB
ArgR	TF	RegulonDB
ArsR	TF	RegulonDB
AscG	TF	RegulonDB
AsnC	TF	RegulonDB
AtoC	TF	RegulonDB
BaeR	TF	RegulonDB
BetI	TF	RegulonDB
BglJ	TF	RegulonDB
BirA	TF	RegulonDB
BolA	TF	RegulonDB
CRP	TF	RegulonDB
CadC	TF	RegulonDB
CaiF	TF	RegulonDB
Cbl	TF	RegulonDB
CdaR	TF	RegulonDB
ChbR	TF	RegulonDB
CpxR	TF	RegulonDB
CreB	TF	RegulonDB
CsgD	TF	RegulonDB
CsiR	TF	RegulonDB
CspA	TF	RegulonDB
CueR	TF	RegulonDB
CusR	TF	RegulonDB
CynR	TF	RegulonDB
CysB	TF	RegulonDB
CytR	TF	RegulonDB
DcuR	TF	RegulonDB
DeoR	TF	RegulonDB
DgsA	TF	RegulonDB
DhaR	TF	RegulonDB
DicA	TF	RegulonDB
DnaA	TF	RegulonDB
DsdC	TF	RegulonDB
EbgR	TF	RegulonDB
EnvR	TF	RegulonDB
EnvY	TF	RegulonDB

EvgA	TF	RegulonDB
ExuR	TF	RegulonDB
FNR	TF	RegulonDB
FabR	TF	RegulonDB
FadR	TF	RegulonDB
FeaR	TF	RegulonDB
FhlA	TF	RegulonDB
Fis	TF	RegulonDB
FlhDC	TF	RegulonDB
FruR	TF	RegulonDB
FucR	TF	RegulonDB
Fur	TF	RegulonDB
GadE	TF	RegulonDB
GadW	TF	RegulonDB
GadX	TF	RegulonDB
GalR	TF	RegulonDB
GalS	TF	RegulonDB
GatR	TF	RegulonDB
GcvA	TF	RegulonDB
GlcC	TF	RegulonDB
GlpR	TF	RegulonDB
GlrR	TF	RegulonDB
GntR	TF	RegulonDB
GutM	TF	RegulonDB
GutR	TF	RegulonDB
H-NS	TF	RegulonDB
HU	TF	RegulonDB
HcaR	TF	RegulonDB
HdfR	TF	RegulonDB
HipB	TF	RegulonDB
HyfR	TF	RegulonDB
IHF	TF	RegulonDB
IclR	TF	RegulonDB
IdnR	TF	RegulonDB
IlvY	TF	RegulonDB
IscR	TF	RegulonDB
KdgR	TF	RegulonDB
KdpE	TF	RegulonDB
LacI	TF	RegulonDB
LeuO	TF	RegulonDB
LexA	TF	RegulonDB
LldR	TF	RegulonDB
LrhA	TF	RegulonDB
Lrp	TF	RegulonDB
LsrR	TF	RegulonDB
LysR	TF	RegulonDB
MalI	TF	RegulonDB
MalT	TF	RegulonDB
MarA	TF	RegulonDB
MarR	TF	RegulonDB
MelR	TF	RegulonDB
MetJ	TF	RegulonDB
MetR	TF	RegulonDB

MhpR	TF	RegulonDB
MngR	TF	RegulonDB
MntR	TF	RegulonDB
ModE	TF	RegulonDB
MprA	TF	RegulonDB
MqsR-YgiT	TF	RegulonDB
MtlR	TF	RegulonDB
MurR	TF	RegulonDB
Nac	TF	RegulonDB
NadR	TF	RegulonDB
NagC	TF	RegulonDB
NanR	TF	RegulonDB
NarL	TF	RegulonDB
NarP	TF	RegulonDB
NemR	TF	RegulonDB
NhaR	TF	RegulonDB
NikR	TF	RegulonDB
NorR	TF	RegulonDB
NrdR	TF	RegulonDB
NsrR	TF	RegulonDB
NtrC	TF	RegulonDB
OmpR	TF	RegulonDB
OxyR	TF	RegulonDB
PaaX	TF	RegulonDB
PdhR	TF	RegulonDB
PepA	TF	RegulonDB
PhoB	TF	RegulonDB
PhoP	TF	RegulonDB
PrpR	TF	RegulonDB
PspF	TF	RegulonDB
PurR	TF	RegulonDB
PutA	TF	RegulonDB
QseB	TF	RegulonDB
RbsR	TF	RegulonDB
RcnR	TF	RegulonDB
RcsAB	TF	RegulonDB
RelEB	TF	RegulonDB
RhaR	TF	RegulonDB
RhaS	TF	RegulonDB
Rob	TF	RegulonDB
RstA	TF	RegulonDB
RtcR	TF	RegulonDB
RutR	TF	RegulonDB
SdiA	TF	RegulonDB
SgrR	TF	RegulonDB
SlyA	TF	RegulonDB
SoxR	TF	RegulonDB
SoxS	TF	RegulonDB
StpA	TF	RegulonDB
TdcA	TF	RegulonDB
TdcR	TF	RegulonDB
TorR	TF	RegulonDB
TreR	TF	RegulonDB

TrpR	TF	RegulonDB
TyrR	TF	RegulonDB
UhpA	TF	RegulonDB
UidR	TF	RegulonDB
UlaR	TF	RegulonDB
UxuR	TF	RegulonDB
XapR	TF	RegulonDB
XylR	TF	RegulonDB
YdeO	TF	RegulonDB
YeiL	TF	RegulonDB
YiaJ	TF	RegulonDB
YoeB-YefM	TF	RegulonDB
ZntR	TF	RegulonDB
ZraR	TF	RegulonDB
Zur	TF	RegulonDB
AbrB	TF	Ecocyc (predicted)
CspC	TF	Ecocyc (predicted)
CspE	TF	Ecocyc (predicted)
FeoC	TF	Ecocyc (predicted)
GreA	TF	Ecocyc (predicted)
GreB	TF	Ecocyc (predicted)
HepA	TF	Ecocyc (predicted)
HupA	TF	Ecocyc (predicted)
HupB	TF	Ecocyc (predicted)
MatA	TF	Ecocyc (predicted)
McbR	TF	Ecocyc (predicted)
MlrA	TF	Ecocyc (predicted)
NusA	TF	Ecocyc (predicted)
PuuR	TF	Ecocyc (predicted)
RacR	TF	Ecocyc (predicted)
Rof	TF	Ecocyc (predicted)
RplD	TF	Ecocyc (predicted)
RpsJ	TF	Ecocyc (predicted)
SspA	TF	Ecocyc (predicted)
SsrS	TF	Ecocyc (predicted)
TorI	TF	Ecocyc (predicted)
YagP	TF	Ecocyc (predicted)
YahA	TF	Ecocyc (predicted)
YahD	TF	Ecocyc (predicted)
YaiV	TF	Ecocyc (predicted)
YaiW	TF	Ecocyc (predicted)
YbaQ	TF	Ecocyc (predicted)
YbdO	TF	Ecocyc (predicted)
YbhD	TF	Ecocyc (predicted)
YbhN	TF	Ecocyc (predicted)
YbiH	TF	Ecocyc (predicted)
YcfQ	TF	Ecocyc (predicted)
YchA	TF	Ecocyc (predicted)
YchQ	TF	Ecocyc (predicted)
YdcN	TF	Ecocyc (predicted)
YdcR	TF	Ecocyc (predicted)
YddM	TF	Ecocyc (predicted)
YdeM	TF	Ecocyc (predicted)

YdhB	TF	Ecocyc (predicted)
YdiP	TF	Ecocyc (predicted)
YdjF	TF	Ecocyc (predicted)
YeaM	TF	Ecocyc (predicted)
YeaT	TF	Ecocyc (predicted)
YebK	TF	Ecocyc (predicted)
YehT	TF	Ecocyc (predicted)
YfaX	TF	Ecocyc (predicted)
YfeD	TF	Ecocyc (predicted)
YfhH	TF	Ecocyc (predicted)
YgaV	TF	Ecocyc (predicted)
YgeH	TF	Ecocyc (predicted)
YghO	TF	Ecocyc (predicted)
YgiV	TF	Ecocyc (predicted)
YgjM	TF	Ecocyc (predicted)
YhcF	TF	Ecocyc (predicted)
YiaG	TF	Ecocyc (predicted)
YidF	TF	Ecocyc (predicted)
YifE	TF	Ecocyc (predicted)
YiiE	TF	Ecocyc (predicted)
YjdC	TF	Ecocyc (predicted)
YjfJ	TF	Ecocyc (predicted)
YjgJ	TF	Ecocyc (predicted)
YjhU	TF	Ecocyc (predicted)
YjjM	TF	Ecocyc (predicted)
YnfL	TF	Ecocyc (predicted)
YphH	TF	Ecocyc (predicted)
YqjI	TF	Ecocyc (predicted)
YrbA	TF	Ecocyc (predicted)
YtfA	TF	Ecocyc (predicted)
YtfH	TF	Ecocyc (predicted)
Spf	sRNA	Ecocyc or RFAM
MicF	sRNA	Ecocyc or RFAM
DicF	sRNA	Ecocyc or RFAM
Tff	sRNA	Ecocyc or RFAM
RprA	sRNA	Ecocyc or RFAM
Tpr	sRNA	Ecocyc or RFAM
DsrA	sRNA	Ecocyc or RFAM
OxyS	sRNA	Ecocyc or RFAM
RyfA	sRNA	Ecocyc or RFAM
CyaR	sRNA	Ecocyc or RFAM
GadY	sRNA	Ecocyc or RFAM
GlmZ	sRNA	Ecocyc or RFAM
RyeB	sRNA	Ecocyc or RFAM
PsrO	sRNA	Ecocyc or RFAM
GcvB	sRNA	Ecocyc or RFAM
RyeA	sRNA	Ecocyc or RFAM
IsrA	sRNA	Ecocyc or RFAM
RttR	sRNA	Ecocyc or RFAM
RdlD	sRNA	Ecocyc or RFAM
SibA	sRNA	Ecocyc or RFAM
RyhA	sRNA	Ecocyc or RFAM
RdlB	sRNA	Ecocyc or RFAM

RdlA	sRNA	Ecocyc or RFAM
RybA	sRNA	Ecocyc or RFAM
OmrA	sRNA	Ecocyc or RFAM
PsrD	sRNA	Ecocyc or RFAM
SibC	sRNA	Ecocyc or RFAM
IsrC	sRNA	Ecocyc or RFAM
SokB	sRNA	Ecocyc or RFAM
RyhB	sRNA	Ecocyc or RFAM
RyjA	sRNA	Ecocyc or RFAM
OmrB	sRNA	Ecocyc or RFAM
RybB	sRNA	Ecocyc or RFAM
PsrN	sRNA	Ecocyc or RFAM
RdlC	sRNA	Ecocyc or RFAM
SgrS	sRNA	Ecocyc or RFAM
RydB	sRNA	Ecocyc or RFAM
SokC	sRNA	Ecocyc or RFAM
SibD	sRNA	Ecocyc or RFAM
SibB	sRNA	Ecocyc or RFAM
MicC	sRNA	Ecocyc or RFAM
GlmY	sRNA	Ecocyc or RFAM
SraD	sRNA	Ecocyc or RFAM
C0465	sRNA	Ecocyc or RFAM
C0719	sRNA	Ecocyc or RFAM
IS128	sRNA	Ecocyc or RFAM
SroB	sRNA	Ecocyc or RFAM
SroC	sRNA	Ecocyc or RFAM
SroD	sRNA	Ecocyc or RFAM
SroE	sRNA	Ecocyc or RFAM
SroH	sRNA	Ecocyc or RFAM
Istr2	sRNA	Ecocyc or RFAM
Tp2	sRNA	Ecocyc or RFAM
Tpke11	sRNA	Ecocyc or RFAM
Tpke70	sRNA	Ecocyc or RFAM
SymR	sRNA	Ecocyc or RFAM
SroG	sRNA	Ecocyc or RFAM
SroA	sRNA	Ecocyc or RFAM
SraA	sRNA	Ecocyc or RFAM
RyjB	sRNA	Ecocyc or RFAM
RyfD	sRNA	Ecocyc or RFAM
RyfB	sRNA	Ecocyc or RFAM
RydC	sRNA	Ecocyc or RFAM
OhsC	sRNA	Ecocyc or RFAM
IsrB	sRNA	Ecocyc or RFAM
C0664	sRNA	Ecocyc or RFAM
C0614	sRNA	Ecocyc or RFAM
C0362	sRNA	Ecocyc or RFAM
C0343	sRNA	Ecocyc or RFAM
C0299	sRNA	Ecocyc or RFAM
C0293	sRNA	Ecocyc or RFAM
C0067	sRNA	Ecocyc or RFAM

Table List of regulators

[a]Regulator ID: ID of regulators.

[b]Regulator type: type of regulator. A regulator can is defined as a TF or a small RNA.

[c]Source: an indication of the sources from which the respective regulator was derived: RegulonDB, predicted transcriptional factors from Ecocyc, sRNAs from Ecocyc, or RFAM).
