# Supplementary material for: Inferring the relation between transcriptional and posttranscriptional regulation from expression compendia
Source: BMC Microbiol. 2014 Jan 27;14:14. doi: 10.1186/1471-2180-14-14 (PMC3948049; doi:10.1186/1471-2180-14-14)
Supplement: Additional file 10: Table S8 — Description of our benchmark of known sRNA-target interactions. [file 1471-2180-14-14-S10.pdf]

**Additional file 10 - Table 8: Description of our benchmark of known sRNA-target interactions**

| [a]sRNA ID | [b]mRNA target ID | [c]Reference | [d]Ecocyc | [e]RegulonDB | [f]Storz |
|---|---|---|---|---|---|
| MicA | ompX | (Johansen et al. 2008) | x | x | x |
| MicA | htrG | (Gogol et al. 2011) | | | x |
| MicA | ompW | (Gogol et al. 2011) | | | x |
| MicA | fimB | (Gogol et al. 2011) | | | |
| ArcZ | rpoS | (Mandin and Gottesman 2010) | x | | x |
| ArcZ | arcB | (Mandin and Gottesman 2010) | x | | |
| CyaR | ompX | (De Lay and Gottesman 2009) | x | x | x |
| CyaR | yqaE | (De Lay and Gottesman 2009) | x | x | x |
| CyaR | nadE | (De Lay and Gottesman 2009) | x | x | x |
| CyaR | luxS | (De Lay and Gottesman 2009) | x | x | x |
| DicF | ftsZ | (Tetart et al. 1992) | x | x | |
| DsrA | hns | (Lease and Belfort 2000; Lease et al. 1998) | x | x | x |
| DsrA | rpoS | (Majdalani et al. 1998; Worhunsky et al. 2003) | x | x | x |
| GadY | gadX | (Opdyke  et al. 2004) | x | x | |
| GcvB | oppA | (Pulvermacher et al. 2009a; Urbanowski  et al. 2000;Sharma  et al. 2007) | x | x | x |
| GcvB | dppA | (Pulvermacher et al. 2009a; Urbanowski  et al. 2000;Sharma  et al. 2007) | x | x | x |
| GcvB | livJ | (Urbanowski et al. 2000;Sharma et al. 2007) | x | x | x |
| GcvB | sstT | (Pulvermacher et al. 2009c;Sharma et al. 2007) | x | x | x |
| GcvB | gltI | (Sharma et al. 2007; Urbanowski et al. 2000) | x | x | x |
| GcvB | cycA | (Pulvermacher et al. 2009b) | x | x | x |
| GcvB | argT | (Sharma et al. 2007) | x | x | x |
| GcvB | livK | (Pulvermacher et al. 2009b; Sharma et al. 2007) | x | x | x |
| GcvB | brnQ | (Sharma et al. 2009; Sharma et al. 2011) | | | x |
| GcvB | ndk | (Sharma et al. 2009; Sharma et al. 2011 | | | x |
| GcvB | ybdH | (Sharma et al. 2009; Sharma et al. 2011 | | | x |
| GcvB | ilvE | (Sharma et al. 2009; Sharma et al. 2011 | | | x |
| GcvB | tppB | (Sharma et al. 2011) | | | x |
| GcvB | ilvC | (Sharma et al. 2011) | | x | x |
| GcvB | serA | (Sharma et al. 2011) | | x | x |
| GcvB | iciA | (Sharma et al. 2011) | | | x |
| GcvB | yaeC | (Sharma et al. 2011) | | | x |
| GcvB | thrL | (Sharma et al. 2011) | | | x |

| | | | | | |
|---|---|---|---|---|---|
| GcvB | gdhA | (Sharma et al. 2011) | | | x |
| GcvB | lrp | (Sharma et al. 2011) | x | | x |
| GcvB | csgD | (Jørgensen et al. 2012) | x | | |
| GcvB | PhoP | (Coornaert et al. 2013) | | | |
| GlmY | glmZ | (Urban and Vogel 2007) | x | x | |
| GlmZ | glmS | (Kalamorz et al. 2007) | x | x | x |
| Istr2 | tisB | (Vogel et al. 2004) | x | | |
| Istr2 | tisA | (Vogel et al. 2004) | x | | |
| MicA | ompA | (Udekwu et al. 2005) | x | x | x |
| MicA | lamB | (Bossi and Figueroa-Bossi 2007) | | | x |
| MicA | tsx | (Gogol et al. 2011) | | | x |
| MicA | yfeK | (Gogol et al. 2011) | | | x |
| MicA | phoP | (Coornaert et al. 2010) | | | x |
| MicC | ompC | (Chen et al. 2004; Urban and Vogel 2007) | x | x | x |
| MicF | ompF | (Andersen and Delihas 1990; Andersen et al. 1987) | x | x | x |
| MicF | lrp | (Holmqvist et al. 2012) | x | | x |
| MicF | phoE | (Holmqvist et al. 2012) | x | | |
| MicF | cpxR | (Holmqvist et al. 2012) | x | | |
| MicM | ybfM | (Rasmussen et al. 2009) | x | x | x |
| MicM | dpiB | (Mandin and Gottesman 2009) | x | | x |
| OmrA | ompT | (Guillier and Gottesman 2006; Guillier and Gottesman 2008) | x | x | x |
| OmrA | fecA | (Guillier and Gottesman 2006) | x | x | x |
| OmrA | cirA | (Guillier and Gottesman 2006) | x | x | x |
| OmrA | fepA | (Guillier and Gottesman 2006) | x | x | x |
| OmrA | csgD | (Holmqvist et al. 2012) | x | x | x |
| OmrA | ompR | (Guillier and Gottesman 2008) | x | x | x |
| OmrB | ompT | (Guillier and Gottesman 2006; Guillier and Gottesman 2008) | x | x | x |
| OmrB | gntP | (Guillier and Gottesman 2006; Tjaden et al. 2006) | x | x | |
| OmrB | fecA | (Guillier and Gottesman 2006) | x | x | x |
| OmrB | cirA | (Guillier and Gottesman 2006) | x | x | x |
| OmrB | csgD | (Holmqvist et al. 2012) | x | x | x |
| OmrB | ompR | (Guillier and Gottesman 2008) | x | x | x |
| OxyS | fhlA | (Argaman and Altuvia 2000) | x | x | x |
| OxyS | rpoS | (Tjaden et al. 2006; Zhang et al. 1998) | x | x | |
| OxyS | ybaY | (Tjaden et al. 2006) | x | x | x |
| OxyS | yobF | (Tjaden et al. 2006) | x | | x |

| | | | | | |
|---|---|---|---|---|---|
| RprA | rpoS | (Lease and Woodson 2004; Majdalani et al. 2001) | x | x | x |
| RprA | csgD | (Mika et al. 2012) | x | | |
| RprA | ydaM | (Mika et al. 2012) | x | | |
| RybB | ompW | (Johansen et al. 2006; Papenfort et al. 2006) | x | x | x |
| RybB | ompC | (Johansen et al. 2006; Papenfort et al. 2006) | x | x | x |
| RybB | ompA | (Gogol et al. 2011) | | | |
| RybB | lamB | (Gogol et al. 2011) | | | |
| RybB | htrG | (Gogol et al. 2011) | | | |
| RybB | tsx | (Gogol et al. 2011) | | | |
| RybB | yfeK | (Gogol et al. 2011) | | | |
| RybB | rluD | (Gogol et al. 2011) | | | |
| RybB | hinT | (Gogol et al. 2011) | | | |
| RybB | fumC | (Gogol et al. 2011) | | | |
| RybB | ydeN | (Gogol et al. 2011) | | | |
| RybB | nmpC | (Gogol et al. 2011) | | | |
| RybB | yhiJ | (Gogol et al. 2011) | | | |
| RybB | rraB | (Gogol et al. 2011) | | | |
| RybB | ompF | (Gogol et al. 2011) | | | |
| RybB | ycfL | (Gogol et al. 2011) | | | |
| RybB | fadL | (Gogol et al. 2011) | | | |
| RybB | fimA | (Gogol et al. 2011) | | | |
| RybB | rbsB | (Gogol et al. 2011) | | | |
| RybB | asr | (Gogol et al. 2011) | | | |
| RybB | fiu | (Gogol et al. 2011) | | | x |
| RydC | yejA | (Antal et al. 2005) | x | x | |
| RyhB | fumA | (Masse and Gottesman 2002) | x | x | |
| RyhB | iscS | (Desnoyers et al. 2009) | x | x | x |
| RyhB | sdhC | (Masse and Gottesman 2002) | x | | x |
| RyhB | shiA | (Prevost et al. 2007) | x | x | x |
| RyhB | sodB | (Geissmann and Touati 2004) | | x | x |
| RyhB | uof | (Večerek et al. 2007) | x | x | |
| RyhB | fur | (Večerek et al. 2007) | | | x |
| RyhB | cysE | (Salvail et al. 2010) | x | x | x |
| SgrS | ptsG | (Kawamoto et al. 2006; Vanderpool and Gottesman 2004) | x | x | x |
| SgrS | manX | (Rice and Vanderpool 2011) | x | | x |
| SgrS | yigL | (Papenfort, 2013) | | | |

| | | | | | |
|---|---|---|---|---|---|
| SokB | mokB | (Faridani et al. 2006) | x | x | |
| SokB | hokB | (Faridani et al. 2006) | x | | |
| SokC | mokC | (Pedersen and Gerdes 1999) | x | x | |
| SokC | hokC | (Pedersen and Gerdes 1999) | x | | |
| Spf | galK | (Møller et al. 2002) | x | x | x |
| Spf | nanC | (Beisel and Storz 2011) | | | |
| Spf | xylF | (Beisel and Storz 2011) | | | |
| Spf | fucI | (Beisel and Storz 2011) | | | |
| Spf | fucK | (Beisel and Storz 2011) | | | |
| Spf | nanT | (Beisel and Storz 2011) | | | |
| Spf | gltA | (Beisel and Storz 2011) | | | |
| Spf | srlA | (Beisel and Storz 2011) | | | |
| Spf | sthA | (Beisel and Storz 2011) | | | |
| SymR | symE | (Kawano et al. 2007) | x | x | |

[a]**sRNA ID** : ID of the sRNA for which at least one target has been described previously. Those has "=" means the sRNA has more than one ID.
[b]**mRNA target ID** : ID of the target of corresponding sRNA from previous column
[c]**Reference** : paper in which corresponding sRNA-target interaction has been described (direct binding or strong evidence for a direct interaction).
[d]**Ecocyc** : "x" in the corresponding cell means the respective sRNA-target interaction has been documented in Ecocyc.
[e]**RegulonDB** : "x" in the corresponding cell means the respective sRNA-target interaction has been documented in RegulonDB.
[f]**Storz** : "x" in the corresponding cell indicates the respective sRNA-target has been described in Storz et al. 2011. sRNA-target interactions indicated in red are HFQ independent.

## References


Altuvia, S., D. Weinstein-Fischer, A. Zhang, L. Postow and G. Storz (1997). "A small, stable RNA induced by oxidative stress: role as a pleiotropic regulator and antimutator." Cell **90**(1): 43-53.

Andersen, J. and N. Delihas (1990). "micF RNA binds to the 5' end of ompF mRNA and to a protein from Escherichia coli." Biochemistry **29**(39): 9249-9256.

Andersen, J., N. Delihas, K. Ikenaka, P. J. Green, O. Pines, O. Ilercil and M. Inouye (1987). "The isolation and characterization of RNA coded by the micF gene in Escherichia coli." Nucleic Acids Res **15**(5): 2089-2101.

Antal, M., V. Bordeau, V. Douchin and B. Felden (2005). "A small bacterial RNA regulates a putative ABC transporter." J Biol Chem **280**(9): 7901-7908.

Argaman, L. and S. Altuvia (2000). "fhlA repression by OxyS RNA: kissing complex formation at two sites results in a stable antisense-target RNA complex." J Mol Biol **300**(5): 1101-1112.

Beisel, C. L., & Storz, G. (2011). "Discriminating tastes: Physiological contributions of the Hfq-binding small RNA Spot 42 to catabolite repression." RNA biology, 8(5), 766-770.

Bossi, L., & Figueroa- Bossi, N. (2007). "A small RNA downregulates LamB maltoporin in Salmonella." Mol Microbiol, 65(3), 799-810.

Chen, S., A. Zhang, L. B. Blyn and G. Storz (2004). "MicC, a second small-RNA regulator of Omp protein expression in *Escherichia coli*." J Bacteriol **186**(20): 6689-6697.

Coornaert, A., Lu, A., Mandin, P., Springer, M., Gottesman, S., & Guillier, M. (2010). "MicA sRNA links the PhoP regulon to cell envelope stress." Mol Microbiol, 76(2), 467-479.

Coornaert, A., Chiaruttini, C., Springer, M., & Guillier, M. (2013). "Post-transcriptional control of the *Escherichia coli* PhoQ-PhoP two-component system by multiple sRNAs involves a novel pairing region of GcvB". PLoS genetics, 9(1), e1003156.

De Lay, N. and S. Gottesman (2009). "The Crp-activated small noncoding regulatory RNA CyaR (RyeE) links nutritional status to group behavior." J Bacteriol **191**(2): 461-476.

Desnoyers, G., A. Morissette, K. Prevost and E. Masse (2009). "Small RNA-induced differential degradation of the polycistronic mRNA iscRSUA." EMBO J **28**(11): 1551-1561.

Faridani, O. R., A. Nikravesh, D. P. Pandey, K. Gerdes and L. Good (2006). "Competitive inhibition of natural antisense Sok-RNA interactions activates Hok-mediated cell killing in Escherichia coli." Nucleic Acids Res **34**(20): 5915-5922.

Geissmann, T. A. and D. Touati (2004). "Hfq, a new chaperoning role: binding to messenger RNA determines access for small RNA regulator." EMBO J **23**(2): 396-405.

Gogol, Emily B., Virgil A. Rhodius, Kai Papenfort, Jörg Vogel, and Carol A. Gross. "Small RNAs endow a transcriptional activator with essential repressor functions for single-tier control of a global stress regulon." Proceedings of the National Academy of Sciences 108, no. 31 (2011): 12875-12880.

Guillier, M. and S. Gottesman (2006). "Remodelling of the Escherichia coli outer membrane by two small regulatory RNAs." Mol Microbiol. **59**(1): 231-247.

Guillier, M. and S. Gottesman (2008). "The 5' end of two redundant sRNAs is involved in the regulation of multiple targets, including their own regulator." Nucleic Acids Res **36**(21): 6781-6794.

Holmqvist, E., Unoson, C., Reimegård, J., & Wagner, E. G. H. (2012). "A mixed double negative feedback loop between the sRNA MicF and the global regulator Lrp." Mol Microbiol 84, no. 3: 414-427.

Johansen, J., A. A. Rasmussen, M. Overgaard and P. Valentin-Hansen (2006). "Conserved small non-coding RNAs that belong to the sigmaE regulon: role in down-regulation of outer membrane proteins." J Mol Biol **364**(1): 1-8.

Jorgensen MG, Nielsen JS, Boysen A, Franch T, Moller-Jensen J, Valentin-Hansen P(2012)." Small regulatory RNAs control the multi-cellular adhesive lifestyle of Escherichia coli." Mol Microbiol, 84(1):36-50.

Kalamorz, F., B. Reichenbach, W. Marz, B. Rak and B. Gorke (2007). "Feedback control of glucosamine-6-phosphate synthase GlmS expression depends on the small RNA GlmZ and involves the novel protein YhbJ in Escherichia coli." Mol Microbiol **65**(6): 1518-1533.

Kawamoto, H., Y. Koide, T. Morita and H. Aiba (2006). "Base-pairing requirement for RNA silencing by a bacterial small RNA and acceleration of duplex formation by Hfq." Mol Microbiol **61**(4): 1013-1022.

Kawano, M., L. Aravind and G. Storz (2007). "An antisense RNA controls synthesis of an SOS-induced toxin evolved from an antitoxin." Mol Microbiol **64**(3): 738-754.

Kawano, M., S. Kanaya, T. Oshima, Y. Masuda, T. Ara and H. Mori (2002). "Distribution of repetitive sequences on the leading and lagging strands of the Escherichia coli genome: comparative study of Long Direct Repeat (LDR) sequences." DNA Res **9**(1): 1-10.

Kawano, M., T. Oshima, H. Kasai and H. Mori (2002). "Molecular characterization of long direct repeat (LDR) sequences expressing a stable mRNA encoding for a 35-amino-acid cell-killing peptide and a cis-encoded small antisense RNA in Escherichia coli." Mol Microbiol **45**(2): 333-349.

Lease, R. A. and M. Belfort (2000). "A trans-acting RNA as a control switch in Escherichia coli: DsrA modulates function by forming alternative structures." Proc Natl Acad Sci U S A **97**(18): 9919-9924.

Lease, R. A., M. E. Cusick and M. Belfort (1998). "Riboregulation in Escherichia coli: DsrA RNA acts by RNA:RNA interactions at multiple loci." Proc Natl Acad Sci U S A **95**(21): 12456-12461.

Lease, R. A. and S. A. Woodson (2004). "Cycling of the Sm-like protein Hfq on the DsrA small regulatory RNA." J Mol Biol **344**(5): 1211-1223.

Majdalani, N., S. Chen, J. Murrow, K. St John and S. Gottesman (2001). "Regulation of RpoS by a novel small RNA: the characterization of RprA." Mol Microbiol **39**(5): 1382-1394.

Majdalani, N., C. Cunning, D. Sledjeski, T. Elliott and S. Gottesman (1998). "DsrA RNA regulates translation of RpoS message by an anti-antisense mechanism, independent of its action as an antisilencer of transcription." Proc Natl Acad Sci U S A **95**(21): 12462-12467.

Mandin, P., & Gottesman, S. (2009). "Regulating the regulator: an RNA decoy acts as an OFF switch for the regulation of an sRNA." Genes & development,23(17), 1981-1985.

Mandin, P. and S. Gottesman (2010). "Integrating anaerobic/aerobic sensing and the general stress response through the ArcZ small RNA." EMBO J **29**(18): 3094-3107.

Masse, E. and S. Gottesman (2002). "A small RNA regulates the expression of genes involved in iron metabolism in *Escherichia coli*." Proc Natl Acad Sci U S A **99**(7): 4620-4625.

Mika, F., Busse, S., Possling, A., Berkholz, J., Tschowri, N., Sommerfeldt, N., Hengge, R. (2012). "Targeting of csgD by the small regulatory RNA RprA links stationary phase, biofilm formation and cell envelope stress in *Escherichia coli*." Mol Microbiol, 84(1), 51-65.

Møller, T., T. Franch, C. Udesen, K. Gerdes and P. Valentin-Hansen (2002). "Spot 42 RNA mediates discoordinate expression of the E. coli galactose operon." Genes Dev **16**(13): 1696-1706.

Opdyke, J. A., J. G. Kang and G. Storz (2004). "GadY, a Small-RNA Regulator of Acid Response Genes in Escherichia coli." J Bacteriol **186**(20): 6698-6705.

Papenfort, K., V. Pfeiffer, F. Mika, S. Lucchini, J. C. Hinton and J. Vogel (2006). "*SigmaE*-dependent small RNAs of *Salmonella* respond to membrane stress by accelerating global omp mRNA decay." Mol Microbiol **62**(6): 1674-1688.

Papenfort K, Sun Y, Miyakoshi M, Vanderpool CK, Vogel J.( 2013)." Small RNA-mediated activation of sugar phosphatase mRNA regulates glucose homeostasis. "Cell, 153(2):426-437.

Pedersen, K. and K. Gerdes (1999). "Multiple hok genes on the chromosome of Escherichia coli." Mol Microbiol **32**(5): 1090-1102.

Prevost, K., H. Salvail, G. Desnoyers, J. F. Jacques, E. Phaneuf and E. Masse (2007). "The small RNA RyhB activates the translation of shiA mRNA encoding a permease of shikimate, a compound involved in siderophore synthesis." Mol Microbiol **64**(5): 1260-1273.

Pulvermacher, S. C., L. T. Stauffer and G. V. Stauffer (2009). "Role of the Escherichia coli Hfq protein in GcvB regulation of oppA and dppA mRNAs." Microbiology **155**(Pt 1): 115-123.

Pulvermacher, S. C., L. T. Stauffer and G. V. Stauffer (2009). "Role of the sRNA GcvB in regulation of cycA in Escherichia coli." Microbiology **155**(Pt 1): 106-114.

Pulvermacher, S. C., L. T. Stauffer and G. V. Stauffer (2009). "The small RNA GcvB regulates sstT mRNA expression in Escherichia coli." J Bacteriol **191**(1): 238-248.

Rasmussen, A. A., J. Johansen, J. S. Nielsen, M. Overgaard, B. Kallipolitis and P. Valentin-Hansen (2009). "A conserved small RNA promotes silencing of the outer membrane protein YbfM." Mol Microbiol **72**(3): 566-577.

Rice, J. B., & Vanderpool, C. K. (2011). "The small RNA SgrS controls sugar–phosphate accumulation by regulating multiple PTS genes." Nucleic acids research, 39(9), 3806-3819

Salvail, H., Lanthier-Bourbonnais, P., Sobota, J. M., Caza, M., Benjamin, J. A. M., Mendieta, M. E. S., ... & Massé, E. (2010). "A small RNA promotes siderophore production through transcriptional and metabolic remodeling." Proceedings of the National Academy of Sciences, 107(34), 15223-15228.

Sharma, C. M., F. Darfeuille, T. H. Plantinga and J. Vogel (2007). "A small RNA regulates multiple ABC transporter mRNAs by targeting C/A-rich elements inside and upstream of ribosome-binding sites." Genes Dev **21**(21): 2804-2817.

Sharma, Cynthia Mira(2009). "Identification of small regulatory RNAs and their targets in bacteria.".

Sharma, C. M., Papenfort, K., Pernitzsch, S. R., Mollenkopf, H. J., Hinton, J. C., & Vogel, J. (2011). "Pervasive post- transcriptional control of genes involved in amino acid metabolism by the Hfq- dependent GcvB small RNA." Mol Microbiol, 81(5), 1144-1165.

Sledjeski, D. and S. Gottesman (1995). "A small RNA acts as an antisilencer of the H-NS-silenced rcsA gene of Escherichia coli." Proc Natl Acad Sci U S A **92**(6): 2003-2007.

Sledjeski, D. D., A. Gupta and S. Gottesman (1996). "The small RNA, DsrA, is essential for the low temperature expression of RpoS during exponential growth in Escherichia coli." EMBO J **15**(15): 3993-4000.

Storz G, Vogel J, Wassarman KM(2011), "Regulation by small RNAs in bacteria: expanding frontiers." Mol Cell, 43(6):880-891.

Tetart, F., R. Albigot, A. Conter, E. Mulder and J. P. Bouche (1992). "Involvement of FtsZ in coupling of nucleoid separation with septation." Mol Microbiol **6**(5): 621-627.

Tjaden, B., S. S. Goodwin, J. A. Opdyke, M. Guillier, D. X. Fu, S. Gottesman and G. Storz (2006). "Target prediction for small, noncoding RNAs in bacteria." Nucleic Acids Res **34**(9): 2791-2802.

Udekwu, K. I., F. Darfeuille, J. Vogel, J. Reimegard, E. Holmqvist and E. G. Wagner (2005). "Hfq-dependent regulation of OmpA synthesis is mediated by an antisense RNA." Genes Dev **19**(19): 2355-2366.

Urban, J. H. and J. Vogel (2007). "Translational control and target recognition by Escherichia coli small RNAs in vivo " Nucleic Acids Res. **35**(3): 1018-1037.

Urbanowski, M. L., L. T. Stauffer and G. V. Stauffer (2000). "The gcvB gene encodes a small untranslated RNA involved in expression of the dipeptide and oligopeptide transport systems in Escherichia coli." Mol Microbiol **37**(4): 856-868.

Vanderpool, C. K. and S. Gottesman (2004). "Involvement of a novel transcriptional activator and small RNA in post-transcriptional regulation of the glucose phosphoenolpyruvate phosphotransferase system." Mol Microbiol **54**(4): 1076-1089.

Večerek, B., Moll, I., & Bläsi, U. (2007). "Control of Fur synthesis by the non-coding RNA RyhB and iron-responsive decoding." The EMBO journal, 26(4), 965-975.

Vogel, J., L. Argaman, E. G. Wagner and S. Altuvia (2004). "The small RNA IstR inhibits synthesis of an SOS-induced toxic peptide." Curr Biol **14**(24): 2271-2276.

Vogel, J., & Luisi, B. F. (2011). "Hfq and its constellation of RNA". Nature Reviews Microbiology, 9(8), 578-589.

Worhunsky, D. J., K. Godek, S. Litsch and P. J. Schlax (2003). "Interactions of the non-coding RNA DsrA and RpoS mRNA with the 30 S ribosomal subunit." J Biol Chem **278**(18): 15815-15824.

Zhang, A., S. Altuvia, A. Tiwari, L. Argaman, R. Hengge-Aronis and G. Storz (1998). "The OxyS regulatory RNA represses rpoS translation and binds the Hfq (HF-I) protein." EMBO J **17**(20): 6061-6068.
